# Altered miRNA expression network in locus coeruleus of depressed suicide subjects

**DOI:** 10.1038/s41598-017-04300-9

**Published:** 2017-06-29

**Authors:** Bhaskar Roy, Qingzhong Wang, Miklos Palkovits, Gabor Faludi, Yogesh Dwivedi

**Affiliations:** 1 Department of Psychiatry and Behavioral Neurobiology, University of Alabama at Birmingham, Birmingham, Alabama 35294 USA; 2 Human Brain Tissue Bank and Laboratory, Semmelweis University, Budapest, H-1094 Hungary; 3 Department of Psychiatry, Semmelweis University, Budapest, H-1125 Hungary

## Abstract

Norepinephrine (NE) is produced primarily by neurons in the locus coeruleus (LC). Retrograde and ultrastructural examinations reveal that the core of the LC and its surrounding region receives afferent projections from several brain areas which provide multiple neurochemical inputs to the LC with changes in LC neuronal firing, making it a highly coordinated event. Although NE and mediated signaling systems have been studied in relation to suicide and psychiatric disorders that increase the risk of suicide including depression, less is known about the corresponding changes in molecular network within LC. In this study, we examined miRNA networks in the LC of depressed suicide completers and healthy controls. Expression array revealed differential regulation of 13 miRNAs. Interaction between altered miRNAs and target genes showed dense interconnected molecular network. Functional clustering of predicated target genes yielded stress induced disorders that collectively showed the complex nature of suicidal behavior. In addition, 25 miRNAs were pairwise correlated specifically in the depressed suicide group, but not in the control group. Altogether, our study revealed for the first time the involvement of LC based dysregulated miRNA network in disrupting cellular pathways associated with suicidal behavior.

## Introduction

Major depressive disorder (MDD) is one of the most debilitating mental disorders world-wide which can lead to significant morbidity and mortality^1, 2^. Although suicide is one of the most devastating consequences of major depression, the precise molecular mechanisms associated with suicidal behavior are yet to be clearly understood. Recent advancements in understanding the neurobiological complexities associated with suicidal behavior have undeniably accepted that aberrant information processing in pathways of participating neural circuits in susceptible brain areas may play a critical role in the pathogenesis of suicide^3^. Identification of epigenetic modifiers as potential regulators of gene function has gained increasing acceptance in explaining the inadvertent changes in cellular network as a consequence of dysregulated gene expression profile orchestrated by environmental stimuli^4, 5^. MicroRNAs (miRNAs) are one of the most important epigenetic modifiers that belong to non-coding RNA family with a precise epigenetic role to modulate the coding potential of transcribed mRNA pool based on characteristic sequence complementarity^6^. In humans so far, more than 2500 mature miRNAs have been annotated^7^ which can act as potential regulatory hub to control a wide array of complex gene network either through direct association or by indirect intermediates^8^. Despite the restricted size (~22 nucleotides) and limited potential to go through exon splicing for generating more structural variations, this small form factor exhibits a functional diversity in targeting diverse range of RNA molecules spanning from protein coding (mRNA) to long non-coding RNAs (lncRNA)^9, 10^. Since gene expression regulation by miRNAs can occur in a coordinated and cohesive fashion, miRNAs can regulate entire genetic circuitries and thereby play a critical role in maintaining biological homeostasis^11, 12^. Thus, any perturbations in the expression of miRNAs may result in the imbalance of homeostasis, which are often reflected as adaptive changes in the regulatory networks that can distinguish normal vs disease states. In this regard, in preclinical model of stress-induced behavioral depression, we have shown that in the prefrontal cortex not only a set of altered miRNAs could transduce wide-spread changes in underlying gene regulatory network implicated in neural plasticity and neural transmission, but they were also involved in causing phenotypic changes associated with depression^13^. Such coordinated changes in miRNA network were also found in the prefrontal cortex of depressed suicide individuals^14^.

As the origin point of noradrenergic neurons, locus coeruleus (LC) is one of the critical brain areas that is known to be involved in behaviors including attention and memory during cognitive tasks and stress response^15^. Pre-clinical studies show profound changes in LC related activities under stressful conditions due to lack of functional integration with hypothalamic-pituitary-adrenal (HPA) axis^16, 17^. In addition, aberrant responses of glutamate and gamma-aminobutyric acid (GABA) that provide direct input to LC, can elicit activity-dependent neuronal responses towards stressful stimuli^18^. The direct role of LC in depression and suicidal behavior comes from studies which demonstrate that supersensitive presynaptic α-2 adrenergic receptors can lead to reduced NE or serotonin, the neurotransmitters most implicated in depression and suicidal behavior^19, 20^. Altogether, these studies suggest that modulations in neurobiological functions associated with LC can cause serious consequences of discordant neurochemical output that can result in increased vulnerability to suicidal behavior^19, 21–23^.

To further understand the role of LC in suicidal behavior at the molecular level, we examined miRNA expression, miRNA regulatory network, and regulated gene expression in this brain area of depressed individuals who committed suicide. Our study provides empirical evidence that miRNAs are capable of altering potential gene regulatory networks and can yield functional clustering of predicated target genes associated with anxiety, hyperactive behavior, and stress-related disorders, which collectively show the complex nature of suicidal behavior. Furthermore, formation of specific coordinated regulatory network with a non-overlapping set of miRNAs identified in depressed-suicide subjects as compared to healthy group may be critical in induction of suicidal behavior.

## Results

### Subjects

The demographic characteristics of healthy non-psychiatric controls and suicide subjects are provided in Table 1. There were no significant differences in PMI (t = 1.36, df = 18, p = 0.19), brain pH (t = 0.46, df = 18, p = 0.65) or RIN^24^ (t = 0.81, df = 18, p = 0.43) between suicide subjects and healthy controls. Age was slightly, but significantly, higher in the control group compared with the suicide group (t = 3.37, df = 18, p = 0.003). There were 2 females and 9 males in control group and 3 females and 6 males in the suicide group.

**Table 1:** Demographic and clinical characteristics of non-psychiatric controls and suicide subjects.

Case #	Age	PMI	SEX	Brain pH	RIN	COD	Drug	Alcohol	Psychiatric Diagnosis
**Control**									
1	66	4.5	M	6.87	7.56	Cardiovascular-pulmonary insufficiency	None	None	Control
2	44	5.0	F	6.75	7.32	Myocardial infarction	None	None	Control
3	53	5.0	M	6.58	7.23	Pulmonary embolism	None	None	Control
4	60	5.0	M	6.88	7.19	Myocardial infarction	None	None	Control
5	52	2.5	M	6.98	7.41	Heart Failure	None	None	Control
6	72	5.5	M	6.89	7.28	Heart Failure	None	None	Control
7	65	1.0	M	6.88	7.06	Heart Failure	None	None	Control
8	74	3.0	M	6.98	7.34	Acute myocardial infarction	None	None	Control
9	89	1.5	F	6.57	7.8	Atherosclerosis cerebri	None	None	Control
10	80	5.5	M	6.92	7.05	Stroke	None	None	Control
11	81	5.0	M	6.77	7.22	Heart failure	None	None	Control
Mean ± SD	66.90 ± 13.88	3.95 ± 1.65	2 F/9 M	6.82 ± 0.14	7.31 ± 0.21				
**Suicide**									
1	42	3.0	F	6.67	7.36	Hanging	None	None	Depression
2	52	3.0	M	6.94	7.27	Hanging	None	None	Depression
3	35	2.0	M	7.01	7.44	Jump from height	None	None	Depression
4	39	12.0	F	6.88	7.16	Hanging	None	None	Depression
5	30	4.0	F	6.72	7.83	Hanging	None	None	Depression
6	71	1.0	M	6.69	7.43	Jump from height	None	None	Depression
7	66	10.0	M	6.86	7.44	Hanging	None	None	Depression
8	31	7.0	M	6.91	7.16	Hanging	None	None	Depression
9	41	10.0	M	6.45	7.43	Hanging	None	None	Depression
Mean ± SD	45.22 ± 14.78	5.77 ± 4.05	3F/6M	6.79 ± 0.17	7.39 ± 0.20				

### Transcriptome-wide changes in miRNAs in LC of suicide subjects

**Figure 1:**
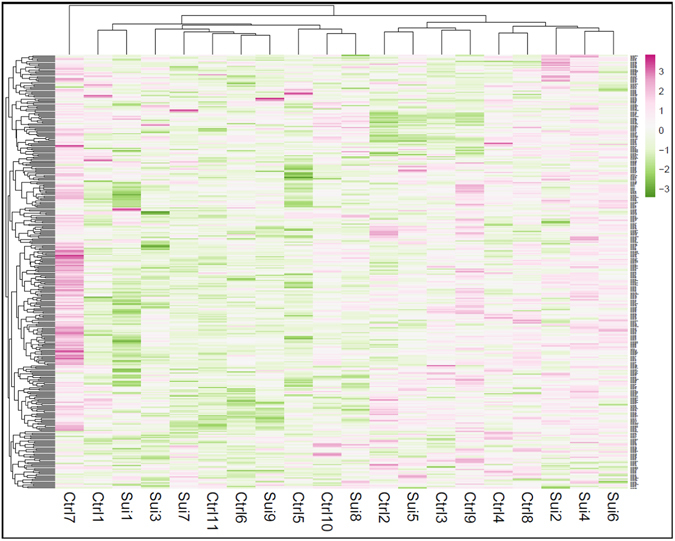
Hierarchical clustering of miRNAs based on median normalized expression data. The construction of dendrogram was based on hierarchical clustering of median normalized miRNA expression data (∆Ct values) in post-mortem locus coeruleus samples which includes both MDD-suicide and healthy control groups. The clustering was prepared following complete linkage method and Euclidean distance. The MDD-suicide group is represented as ‘Sui’ and control is represented as ‘Ctrl’ in this miRNA expression heat map.

The TLDA based miRNA profiler is comprised of two plates: A and B. As detailed in the methods section, each plate assays 377 miRNAs excluding 6 small RNA related genes incorporated in each plate as normalizer and a plant-specific ath-miR-159a as negative control. Initial sorting of miRNA genes was performed based on their Ct values. Genes showing Ct ≤ 35 were excluded from the analysis. Based on the median normalized ΔCT values, a hierarchical clustering of miRNAs was conducted across control and suicide groups following complete linkage and Euclidian distance method and is presented as a Heat Map (Fig. 1). When tested individually, a total of 13 miRNAs achieved statistical significance at p = 0.05 or better (Table 2). Perturbation analysis (SAM, Stanford University), which takes testing of multiple miRNAs into account, identified 13 of these miRNAs as significant at a very stringent false discovery rate of 1.52%. Of these, 8 miRNA belonged to plate A (miR-17-5p, miR-20b-5p, miR-106a-5p, miR-330-3p, miR-409-5p, miR-541-3p, miR-582-5p, miR-890), whereas 5 miRNAs were part of plate B (let-7g-3p, miR-99b-3p, miR-550-5p, miR-1179, miR-1197) (Table 2). Except miR-409-5p in plate A and let-7g-3p and miR-1197 in plate B, all the miRNAs were upregulated. Among upregulated miRNAs, miR-541-3p from plate A was the top ranking miRNA based on its maximum level of expression in the suicide group (fold change = 1.52). On the other hand, miR-550-5p from plate B had the highest expression upregulation (fold change = 1.57) in the suicide group. Among 3 downregulated miRNAs from plates A and B, miR-1197 showed the most change (fold change = 0.48), which was also the most significantly altered miRNA in the suicide group (p = 0.008) (Table 2). All the significantly altered miRNAs in MDD-suicide group are represented as scattered plots in Supplementary Fig. 1. When correlated with confounding variables, we did not find any significant effects of age, PMI or brain pH on the expression of above mentioned altered miRNAs except miR-890 which correlated positively with age in the control group (Supplementary Table 2a). No significant effect of gender was observed on any of the miRNAs except miR-1179, whose expression was significantly higher in the female group (Supplementary Fig. 2)

**Table 2:** significantly altered miRNAs in locus coeruleus of MDD-suicide subjects.

miRBase Acc. No.	TLDA Plate	miRNAs	Fold Change	p-value	Regulation	Chromosomal location	Strand	miRNA Seed (5′-3′)
MIMAT0000070	A	hsa-miR-17-5p	1.260715355	0.012752	↑	Chromosome 13	Sense	AAAGUGC
MIMAT0001413	A	hsa-miR-20b-5p	1.248312981	0.038056	↑	Chromosome X	Antisense	AAAGUGC
MIMAT0000103	A	hsa-miR-106a-5p	1.2312043	0.036205	↑	Chromosome X	Antisense	AAAGUGC
MIMAT0000751	A	hsa-miR-330-3p	1.403274751	0.010941	↑	Chromosome 19	Antisense	CAAAGCA
MIMAT0001638	A	hsa-miR-409-5p	0.780180155	0.039303	↓	Chromosome 14	Sense	GGUUACC
MIMAT0004920	A	hsa-miR-541-3p	1.520180479	0.024907	↑	Chromosome 14	Sense	GGUGGGC
MIMAT0003247	A	hsa-miR-582-5p	1.430205029	0.022995	↑	Chromosome 5	Antisense	UACAGUU
MIMAT0004912	A	hsa-miR-890	1.335886765	0.020283	↑	Chromosome X	Antisense	ACUUGGA
MIMAT0004584	B	hsa-let-7g-3p	0.56503572	0.021121	↓	Chromosome 3	Antisense	GAGGUAG
MIMAT0004678	B	hsa-miR-99b-3p	1.398655254	0.039436	↑	Chromosome 19	Sense	ACCCGUA
MIMAT0004800	B	hsa-miR-550-5p	1.576943392	0.041171	↑	Chromosome 7	Sense	GUGCCUG
MIMAT0005824	B	hsa-miR-1179	1.550548092	0.018529	↑	Chromosome 15	Sense	AGCAUUC
MIMAT0005955	B	hsa-miR-1197	0.4863239	0.00846	↓	Chromosome 14	Sense	AGGACAC

### Clustering of significantly dysregulated miRNAs based on their localization on chromosomes in LC of suicide subjects

The genomic localization of each significantly altered miRNA is shown in Table 2, which was curated following the recent release of miRbase (v.21) database (Manchester, UK). Table 2 also indicates the orientation of transcriptional unit for each individual miRNA which is denoted as either sense or antisense strand of duplex genomic DNA. miRNAs miR-20b-5p, miR-106a-5p and miR-890, which were found to be localized on chromosome X, showed a similar magnitude of increase (~1.25 fold) in LC of suicide subjects. Interestingly, the transcriptional unit for these 3 miRNAs was found to be localized on antisense strand. Apart from clustering on chromosome X, two more clustering groups were identified - one on chromosome 19 and another on chromosome 14. On chromosome 19, two miRNAs (miR-330-3p and miR-99b-3p) had their transcriptional units in opposite direction but they exhibited transcriptional upregulation with the similar fold change (~1.4 fold). On the other hand, three miRNAs (miR-409-5p, miR-541-3p and miR-1197) were located on chromosome 14 with the same directionality of transcription from sense strand. Of these, two miRNAs (miR-409-5p and miR-1197) showed changes in the same direction. These results suggest a coordinated regulation of miRNAs located on the same chromosome.

### Functional clustering of differentially regulated miRNAs and their co-expression analysis in LC of suicide subjects following *in silico* approaches

**Figure 2:**
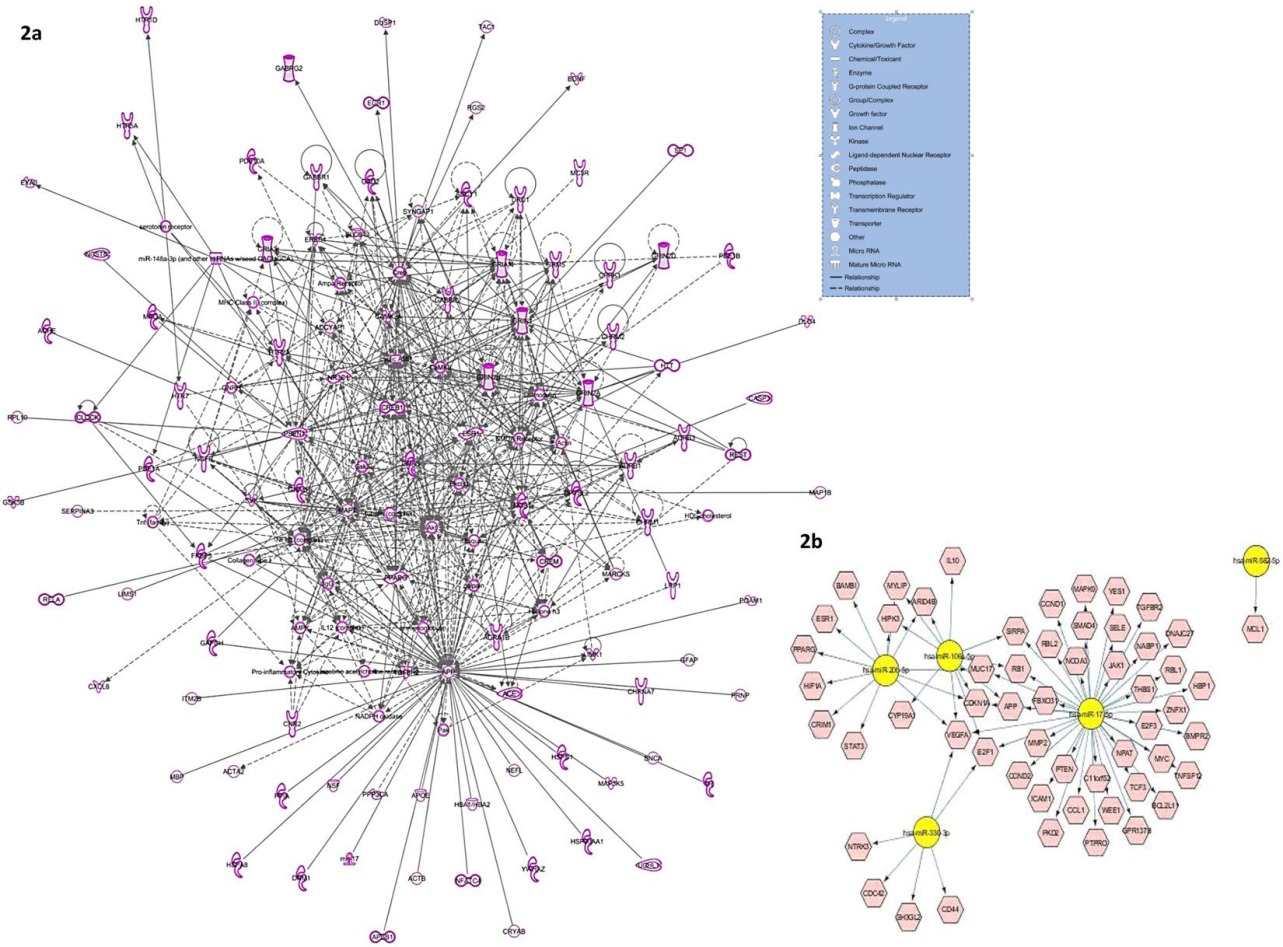
Integrated target gene network based on functional clustering of differentially regulated miRNAs. (**a**) The intense molecular crosstalk (represented as solid lines for direct relationship) is indicative of target enrichment of the differentially regulated miRNAs as found in MDD-suicide group compared to healthy control subjects. In the functional molecular network analysis, shapes of individual molecules are representative of their function and genes are represented as nodes. (**b**) Gene regulatory network of significantly upregulated miRNAs based on validated targets. Using CyTargetLinker plugin from Cytoscape software (version 3.0), functional gene network was constructed based on validated targets of upregulated miRNAs from MDD-suicide group.

As part of integrated miRNA-gene network study, relevance of the differentially regulated miRNAs was contemplated based on their target gene functionality. The computational selection of target genes (Supplementary Table 3) to interpret their global association with 13 differentially expressed miRNAs was performed using the algorithms of three different *in silico* tools: TargetScan, Ingenuity Expert Finding, and miRecords. The respective algorithms select target genes based on either prediction score which is determined by high to moderate confidence level of 3′UTR complementarity or experimentally validated records. Furthermore, functional mapping of these selected target genes help in creating an integrated gene network (Fig. 2a) which elaborates the regulatory role of these differentially expressed miRNAs on target gene expression and associated cellular pathways.

**Figure 3:**
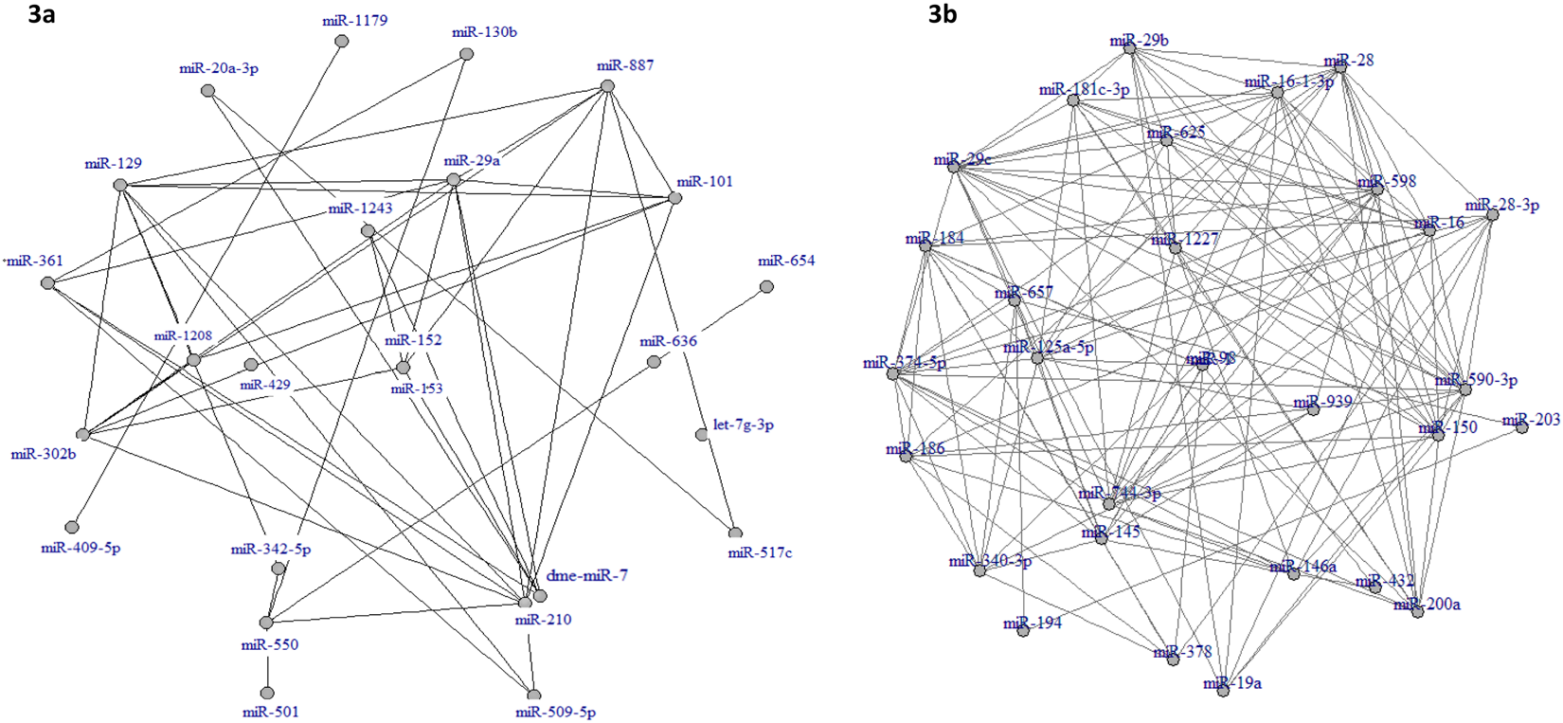
miRNA co-expression network analysis. (**a**) A core set of 25 miRNAs were selectively and highly co-expressed within the suicide group but not in the healthy group. (**b**) A core set of 30 microRNAs were strongly co-expressed and interconnected in the healthy control, but not in the suicide group.

Figure 3a displays network visualization for the core set of 25 miRNAs that were found to be pairwise correlated specifically with the suicide group, but not with the control group. Figure 3b reveals a set of 30 miRNAs, which were pairwise correlated specifically with the control group, but not with the suicide group. Interestingly, the miRNA co-expression network in the control group was very extensive where each miRNA was interconnected with 5–12 miRNAs within the network. On the other hand, in the suicide group, this network was not as dense and except miR-210, all the miRNAs correlated only with 3–4 other miRNAs within the network.

### Analysis of gene regulatory networks associated with altered miRNAs

Since miRNAs are primarily known for their role in causing repressive effects on target gene expression via direct interaction, a possible downstream effect on a set of target genes is expected that may cause an overall dysfunctional status of associated cellular pathways. In this regard, an attempt was made to closely identify the gene networks associated with the subset of miRNAs which were found to be significantly upregulated in the suicide group (Fig. 2b). Closer attention was paid to construct the network based on genes which have previously been validated as target of those miRNAs. Identification of NTRK3, VEGFA, E2Fs, MMP2 as regulated candidate genes indicates the authenticity of this functional miRNA regulatory network.

**Figure 4:**
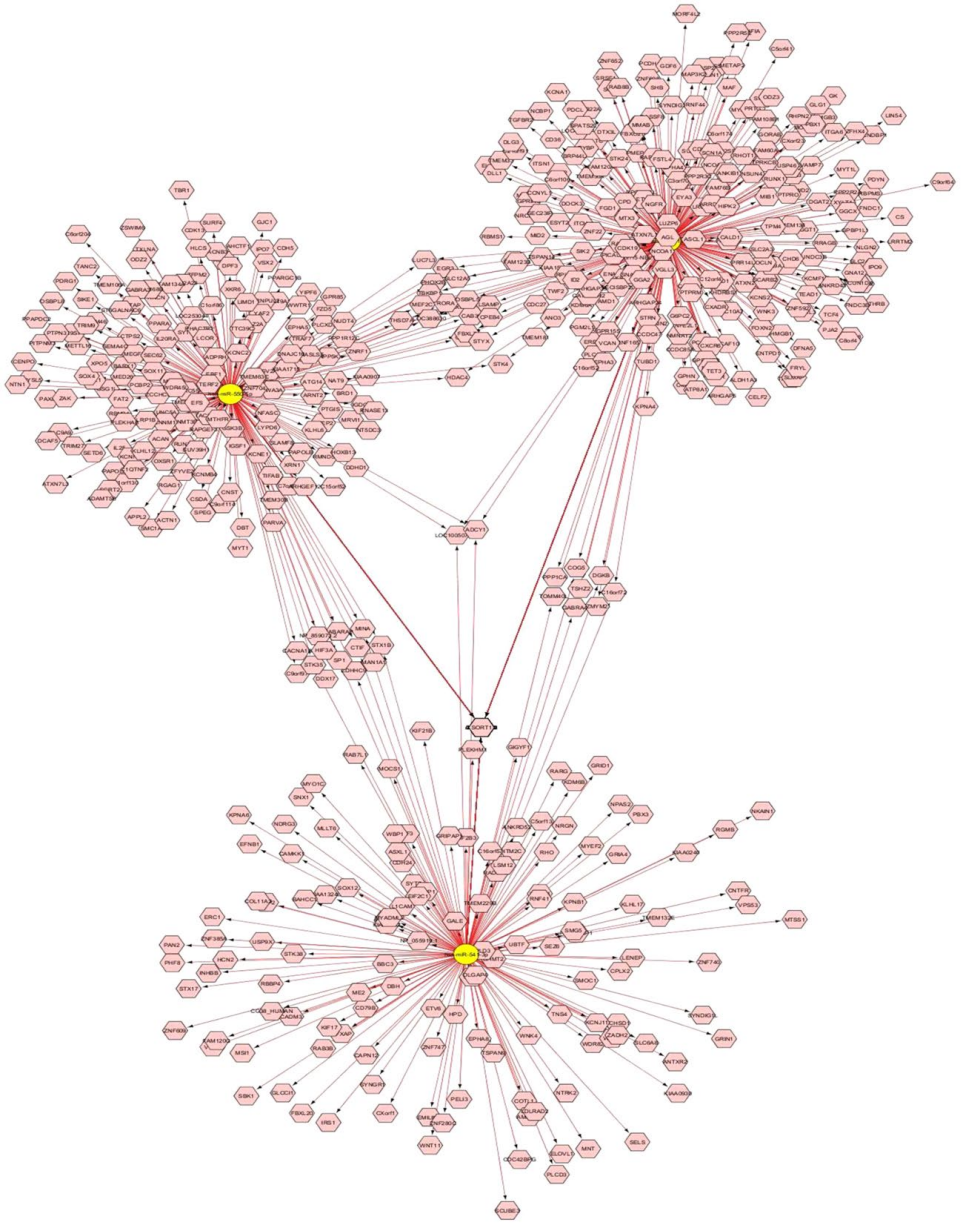
Gene regulatory network of top 3 upregulated miRNAs based on TargetScan target prediction algorithm. Construction of an integrated target gene regulatory network is shown, which was based on the TargetScan derived predicted target genes for top three significantly upregulated miRNAs (miR-541-3p, miR-550-5p and miR-1179). The selection of miRNAs was based on the magnitude of upregulated expression in the suicide group (≥1.5 cutoff in relative fold change).

As an additional measure to strengthen the gene regulatory network data and to have deeper insight on target gene regulation and their related functions, top three miRNAs (miR-541-3p, miR-550-5p and miR-1179) were chosen from upregulated miRNA list and an integrated target gene regulatory network (Fig. 4) was built based on the TargetScan-derived predicted target genes. The selection was based on the magnitude of upregulated expression in the suicide group (≥1.5 cutoff in relative fold change). Notably, each miRNA was found to create its own individual network with putative targets along with overlapping networks with the other two miRNAs where a number of genes were identified as shared targets receiving bimodal regulatory effect. Identification of critical genes (GRIA4, GRIN1, CAMKK, NGFR, NTRK2, SLC2A3, SLC6A8, SUV39H, METTL6, SLC12A5 and GSK3B) from this network for their well-documented role in neuropsychiatry and related epigenetics further indicates their anticipatory involvement in suicide pathology under the current study.

**Figure 5:**
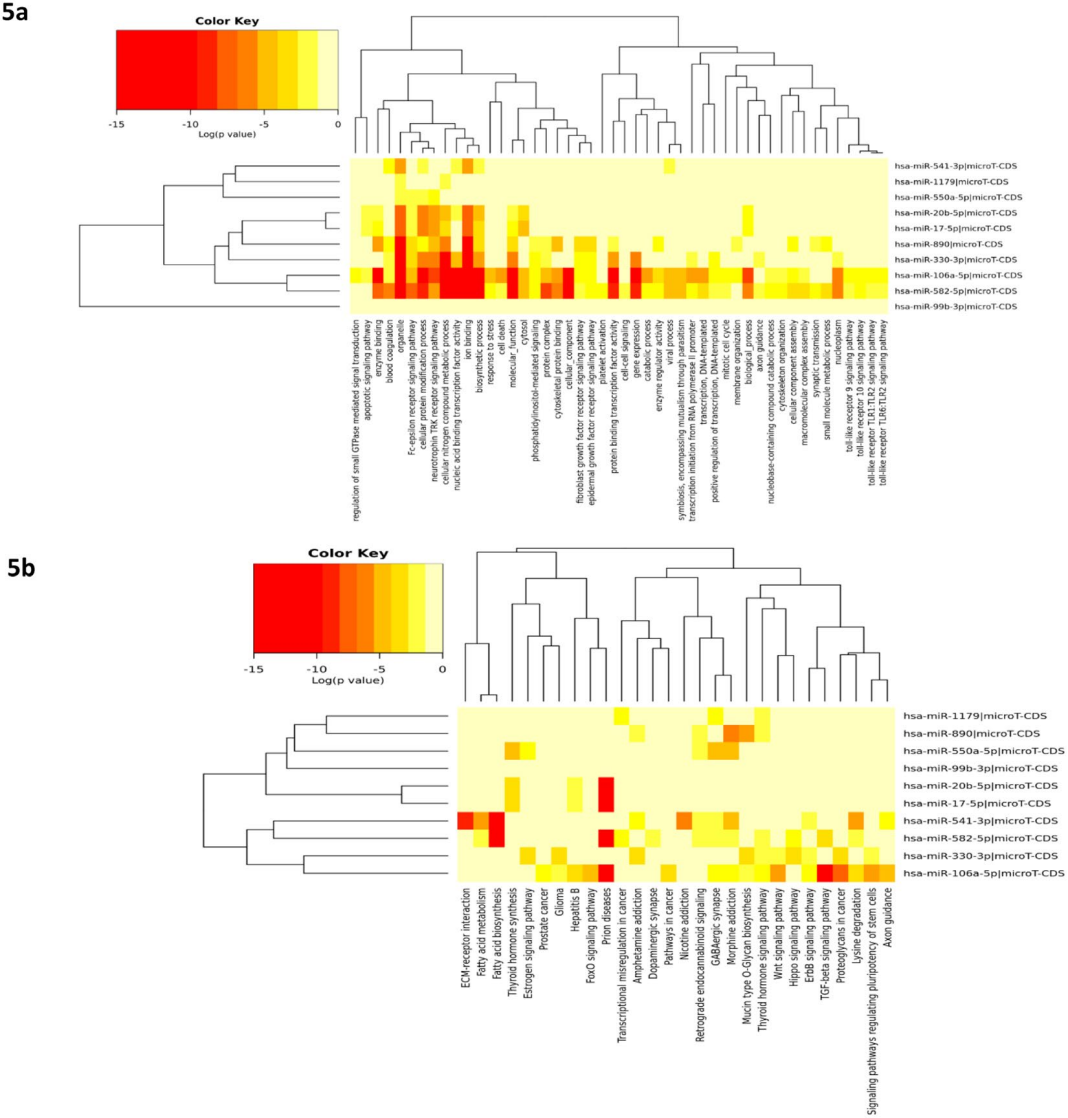
Hierarchical clustering based on predicted target genes of upregulated miRNAs (**a**) Ontology-based hierarchical clustering of genes identified as predicted targets of upregulated miRNAs. Using mirPath (version 3) from DIANA tools, 10 upregulated miRNAs in the suicide group was used to prepare a heat map based on the gene ontology of predicted target genes. A color-coded representation indicated the functional enrichment of genes as targets of significantly upregulated miRNAs from MDD-suicide group. (**b**) Cellular pathway based hierarchical clustering of predicted target genes as part of upregulated miRNAs. Kyoto Encyclopedia of Genes and Genomes (KEGG) based mapping of putative target genes in various cellular pathways as part of the deregulated miRNAs demonstrated the possible involvement of 10 up regulated miRNAs and their canonical target genes.

As part of the 10 upregulated miRNAs in the suicide group, a Heat Map was prepared based on the gene ontology of predicted target genes (Fig. 5a). A functional enrichment of genes was seen for TRK receptor signaling pathway and synaptic transmission as targets of miR-582-5p which ranked fourth in the list of 10 up regulated miRNAs. Target enrichment for the same TRK pathway and synaptic transmission was also observed for the other two top ranking miRNAs, e.g., miR-541-3p and miR-1179.

**Figure 6:**
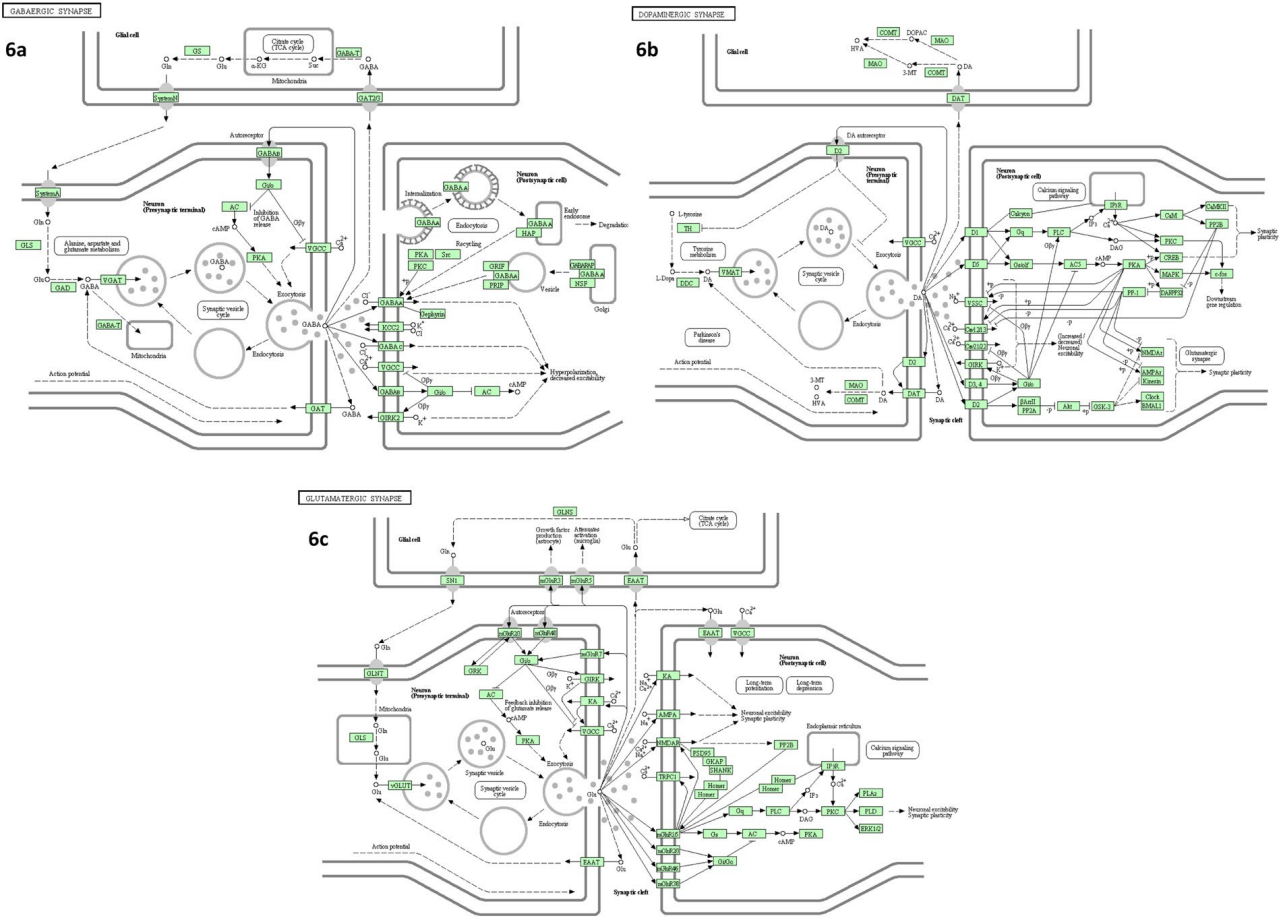
Mapping of predicted target genes in pathways related to various synaptic neurotransmission. (**a**) Mapping of genes were identified as predicted targets of upregulated miRNAs. Elaboration of canonical pathway related to GABAergic synapse function enriched with predicted target genes of upregulated miRNAs in the MDD-suicide group is shown. (**b**) Mapping of genes in dopaminergic synapse identified as predicted targets of upregulated miRNAs. Elaboration of canonical pathway related to dopaminergic synapse function enriched with predicted target genes of upregulated miRNAs in the MDD-suicide group is shown. (**c**) Mapping of genes in glutamatergic synapse identified as predicted targets of downregulated miRNAs. Elaboration of canonical pathway related to dopaminergic synapse function enriched with predicted target genes of downregulated miRNAs in the MDD-suicide group is shown. The pathway images are obtained from Kyoto Encyclopedia of Genes and Genomes (KEGG).

Abnormal cellular physiology is known to be the result of dysfunctional cellular pathways. Under the current study, similar observation was made in suicide subjects when putative target genes were mapped in various cellular pathways as part of the dysregulated miRNAs (Fig. 5b). Kyoto Encyclopedia of Genes and Genomes (KEGG) based pathway analysis^25–27^ demonstrated the possible involvement of 10 upregulated miRNAs and their canonical target genes (e.g. GABA_A_, GABA_B_, GABA_C_, VGCC, GAD, VGAT, GIRK2, PKC, CREB, CAMKII, PKA, GSK-3 and AKT) in impairing GABAergic and dopaminergic neurotransmission (Fig. 6a and b). Closer examination of the participating genes suggests functional enrichment of 50 genes as down regulated targets of 5 upregulated miRNAs in LC of suicide subjects with possible hindrance in GABAergic synaptic function (Supplementary Table 4). On the other hand, 31 target genes were identified as part of the upregulated miRNA network with compromised functional involvement in dopaminergic synapses (Supplementary Table 4). The putative target genes of 3 downregulated miRNAs in LC of suicide subjects and their possible functional mapping in participating cellular pathways identified the involvement of glutamatergic synapse (Fig. 6c). Total 26 genes (Supplementary Table 5) were found to be part of this affected neurotransmitter pathway as being target of miR-409-5p and let-7g-3p; a majority of them were known to be linked with neural plasticity (GRIA3, GRIK3, GRIA4, GRIN2B) and glutamate signaling including feedback inhibition of glutamate release (SLC38A2, SLC1A2, GRM4, GRM7, PRKACB).

As observed from miRNA-target gene relationship, a set of predicted genes with strong neuropsychiatric background was found to be the target of more than one miRNA. Notably, GRIA3 (miR-330-3p, miR-1179), CREB1 (miR-17-5, miR-582-5p), ELF1 (miR-330-3p, miR-582-5p), CHRM2 (miR-1197, miR-17-5p, miR-541-3p), GRIK3 (miR-1197, miR-541-3p), NTRK2 (miR-1197, miR-541-3p), NTRK3 (miR-17-5p, miR-541-3p), EGF (miR-890, miR-330-3p), CREM (miR-1197, miR-330-3p, miR-582-5p) and GRM5 (miR-330-3p, miR-582-5p) showed the presence of canonical sites at their 3′ UTR with seed matches for more than one miRNA from the list of differentially expressed miRNAs.

**Figure 7:**
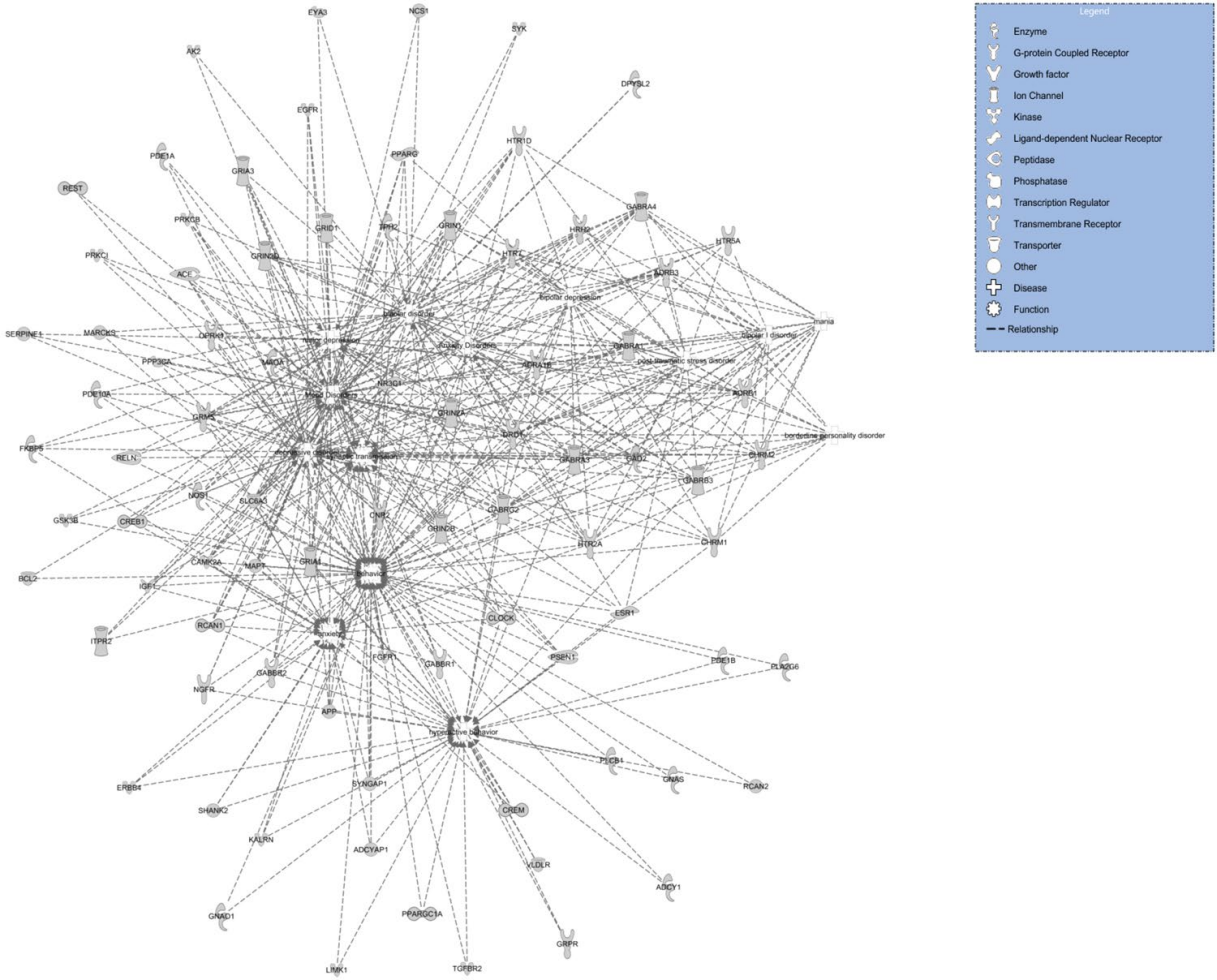
Enriched gene regulatory network of altered miRNAs and their targets with functional relationship to neuropsychiatric disorders. Overlapping target gene network affected by altered miRNAs were mapped. Representing hubs on the network were pathophysiological footprints of associated neuropsychiatric disorders. (p < 0.05, Fisher’s Exact Test).

Further attention was paid to create additional gene regulatory network based on the predicted targets of differentially altered miRNAs in the suicide group (Fig. 7). This time the integrated gene network was constructed with an emphasis to link their functional association with a few mental disabilities earlier found to be of high-risk factor for suicide. As can be seen in Fig. 7, an overlapping pattern of target gene regulation was prominent, which could be a combination of pathophysiological footprint of associated neuropsychiatric disorders as mapped on the network. The nodes in this network were represented as the genes with characteristic shapes that denote their function and the disorders were drawn as the hub in the network. Connections between hubs and nodes as well as between nodes are presented with broken line.

To further understand the relative involvement of identified target genes in disease physiology, functional clustering was considered, which resulted in identification of psychopathologies broadly related to mood disorders. Maximum gene set enrichment belonged to depressive disorder (64%), followed by anxiety (27%) and bipolar (9%) disorders (Supplementary Fig. 3a). In addition, ingenuity pathway based in silico analysis further dissected out anxiety related disorders based on its knowledgebase and retrieved a gene set enrichment for hyperactivity related disabilities (22%) as well as post-traumatic disorder (78%) (Supplementary Fig. 3b).

**Figure 8:**
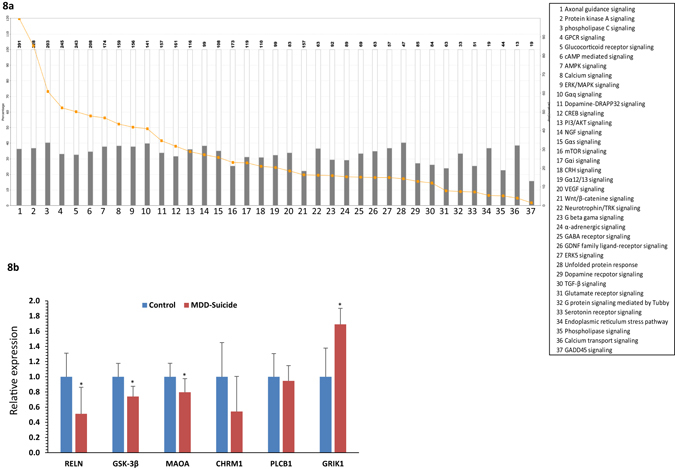
Relating functional disorders with dysregulated miRNAs based on predicted target genes and their validation. (**a**) Affected canonical pathways mediated by deregulated miRNA network. Canonical biological pathways associated with genes that were predicted to be targets of significantly altered miRNAs in MDD-suicide subjects are shown (p < 0.05, Fisher’s Exact Test). The ratio is calculated as the number of genes in a given pathway divided by the number of genes that make up the pathway. The p-value for a given process annotation is calculated by considering the number of focus genes that participate in that process and the total number of genes that are known to be associated with that process in the selected reference set. The more focus genes involved, the more likely the association is not due to a random chance. (**b**) Expression status of few selected genes identified as targets of altered miRNAs. Transcript levels of RELN, GSK-3β, MAOA, CHRM1, PLCB1 and GRIK1 were analyzed in locus coeruleus of MDD-suicide and healthy control subjects by qPCR using primers mentioned in the Methods section. GAPDH normalized relative expression level of RELN, GSK-3β, MAOA, CHRM1, PLCB1 and GRIK1 mRNA transcripts were analyzed in locus coeruleus of MDD-suicide as compared with the normal healthy control group. All data are the mean ± SEM (for RELN, GSK-3β and GRIK1, n = 10 in control and n = 9 in MDD-suicide group; for MAOA, n = 9/group). The level of significance was determined using independent-sample ‘t’ test. ‘*’ denotes significant difference between MDD-suicide and control groups (RELN p = 0.038; GSK-3β p = 0.009; MAOA p = 0.032; GRIK1 p = 0.046).

Besides disease related pathways, abnormalities in cellular pathways were also assessed based on the altered gene regulatory functions attributed by the targeting miRNAs. Following Fisher Exact Test with a p value threshold set at 0.05, the canonical pathway analysis module of IPA identified a list of pathways with relevance to neuropsychiatric disorders (Fig. 8a) such as protein kinase A signaling, phospholipase C signaling, glucocorticoid signaling, ERK/MAPK signaling, neurotrophin signaling, corticotropin releasing signaling, GABAergic signaling and glutamatergic signaling; all of them are known for their role in depression and suicidal behavior.

### Expression analysis of protein coding target genes of altered miRNAs in LC of suicide subjects

To unravel the functional importance of significantly altered miRNAs in the LC of suicide subjects, a set of predicted target genes were screened and short listed based on their relevance in depression and suicide (RELN, GSK-3β, MAOA, CHRM1, PLCB1 and GRIK1). Relative quantification of these selected genes exhibited a common trend of expression down regulation (Fig. 8b) in the LC of suicide subjects compared with matched healthy controls except GRIK1 gene. Almost 50% expression attenuation was seen for RELN gene (Fig. 8b) which was found to be statistically significant (p = 0.03). Likewise, GSK-3β exhibited a similar pattern of downregulation at transcript level (~30%) with very high statistical significance (p = 0.009) (Fig. 8b). Reduced level was also found for MAOA gene, which showed a ~30% reduction in transcript level with a strong statistical significance (p = 0.03) across the analyzed samples (Fig. 8b). Interestingly, no significant change was identified in expression status of CHRM1 and PLCB1 genes; however, a relatively high level of expression downregulation (~56%) was observed for CHRM1 in suicide group (Fig. 8b). On the other hand, ~80% expression upregulation was noted for GRIK1 gene with a strong significance level (p = 0.04). The changes in expression of these altered genes were not associated with age, PMI or brain pH except MAO and PLCB1 genes. MAOA was positively correlated with PMI whereas PLCB1 showed significant correlation with pH (Supplementary Table 2b). Gender did not show any significant effect on RELN, GSK-3β, MAOA expression except CHRM1, PLCB1 and GRIK1 where CHRM1 and PLCB1 were lower in the female group and GRIK1 was higher in the male group (Supplementary Fig. 4).

## Discussion

In the past, LC, a primary source of norepinephrine, has extensively been studied for its role in stress responsiveness^28, 29^. In addition, a considerable amount of attention has been paid to understand the role of LC with perspective of noradrenergic system in suicide, which suggests that dysfunction in central noradrenergic system and associated signaling may lie at the root of psychiatric disorders, including depression, that contribute to suicide^18, 21^. In fact, aberrant release of norepinephrine from the terminals of both afferent and efferent noradrenergic LC projections and subsequent adaptive upregulation of noradrenergic receptors has been reported in suicide subjects^21, 23^. These evidence demonstrate that LC may have high susceptibility to the pathological changes associated with suicide^30^. The present study was undertaken to further examine the role of LC specific miRNAs and associated gene regulatory networks to ascertain their molecular contribution in understanding the pathobiology of suicidal behavior.

For the first time, we not only present the evidence that the expression of miRNAs is substantially altered in LC of suicide subjects but miRNAs as well as target genes may form networks that can be crucial in underlying etiopathogenesis of suicide. We analyzed a total of 754 miRNAs, out of which, 367 miRNAs were further analyzed after normalization. We found that expression of a core group of 13 miRNAs was significantly altered in LC of suicide subjects compared with healthy control subjects. Of them 10 were upregulated (miR-17-5p, miR-20b-5p, miR-106a-5p, miR-330-3p, miR-541-3p, miR-582-5p, miR-890, miR-99b-3p, miR-550-5p, miR-1179) and 3 were downregulated (miR-409-5p, let-7g-3p, miR-1197). Construction of an integrated gene regulatory network based on predicted target genes of altered miRNAs showed a comprehensive association with neuropsychiatric disorders, which included major depression and anxiety, the two important risk factors associated with suicidal behavior. In addition, mapping of cellular pathways, affected by these altered miRNAs, indicated an overall change in cellular signaling that have been implicated in suicide neurobiology. Moreover, formation of a miRNA network, which appeared to be specific to the suicide but not the control group, was noted. Altogether, our study shows that LC associated changes in miRNA expression and molecular networks may play a critical role in the pathophysiology of suicide.

Involvement of region specific neural circuits in the etiopathogenesis of depression and associated suicidal behavior is well documented^31^. In a previous study, we analyzed expression of miRNAs in dorsolateral prefrontal cortex (dlPFC) of suicide subjects^14^. Contrary to LC, we observed a global downregulation of miRNAs in this brain area of suicide subjects compared with matched healthy controls. When examined individually, we found that the expression levels of 21 miRNAs were significantly lower. In addition, 16 additional miRNAs showed a large but non-significant decrease (<35%). Interestingly, alterations in these miRNAs were associated with a set of target genes that were part of functions related to repression of chromatin structure (DNMT3B, EZH2), activation of glucocorticoid receptor (NCOA2), transcription factors (SP1, SP3, SP4 and SOX4), and apoptotic regulatory proteins (BCL2). An overall elevated expression of these target genes was predicted due to downregulated expression of targeting miRNAs in the PFC of depressed-suicide group. This is in agreement with earlier reports in pre-clinical models of depression where profound repression of PFC activity have been reported including decreased PFC volume, dendritic retraction, and spine loss^32^. Moreover, chromatin compaction in PFC of depressed-suicide brain has also been reported which further supports induced activity of those genes found as targets of repressed miRNAs in PFC^33^. In the present study, a contrasting pattern of miRNA expression was observed in the LC of depressed suicide subjects. For example, the number of miRNAs affected in LC of suicide subjects was much lower than the PFC. In addition, none of the miRNAs, which showed alterations in the PFC, except for miR-20b-5p, appeared in the list of LC-associated miRNA changes in suicide subjects. Even the expression of miR-20b-5p was upregulated in LC in contrast to PFC, where its expression was downregulated. Moreover, the elevated levels of miRNAs was found to be responsible for the dysregulation of genes associated primarily with neurotransmitter systems such as glutamate and GABA^34^. Altogether, our results not only suggest a complex neuroanatomical nature of LC receiving various neurochemical inputs across various brain areas^35^ but also indicate that suicide-associated changes in miRNAs are brain region specific and that miRNAs or associated gene networks in specific brain areas may have distinct functions in determining disease pathophysiology.

Disease pathogenesis associated with psychiatric illnesses like suicidal behavior is a highly complex molecular process which involves a diverse array of dysregulation at both genetic and epigenetic levels^36, 37^. In order to have a deeper insight, a system level approach needs to be perused to explain the inherent nature of changes associated with this kind of molecular dysfunctionality^38^. MicroRNAs, as epigenetic modifiers, are capable of regulating large sets of genes through synergism^39^. This synergistic interaction between sets of miRNAs stems out from their same chromosomal localization and regulation by common cellular pathways^40^, which allows one to build a co-relational network which could be disease specific. This is supported by our data in which we identified pairs of miRNAs that were co-regulated in their expression across individuals within a single group (control or suicide). The pairwise correlations for all miRNAs were computed within the control group as well as within the suicide group separately. We were particularly interested in identifying miRNA pairs that showed no significant correlation in the control group, yet exhibited a significant positive correlation in the suicide group and vice-versa. This phenomenon allows to detect coordinated changes as the underlying driving force for expression (e.g., due to changes in transcription factors or miRNA processing) and likely to reflect the influence of shared factor(s) which regulate miRNAs in the brain of healthy control and suicide individuals and can influence the phenotypic outcome. More interestingly, we found that separate sets of miRNAs were pairwise correlated in the LC of suicide group, which was not present in the control group. On the other hand, a separate group of co-regulated miRNAs was pairwise correlated in the control group, which was absent in the suicide group. dentification of two independently regulated synergistic networks with different sets of pairwise correlated miRNAs in control vs MDD-suicide group indicates the likely involvement of a co-regulated target gene set, bearing a suicide specific signature. Furthermore, the mapping of a less intensely interacting miRNA network in the MDD-suicide group compared with control suggests a disrupted synergistic network due to maladaptive changes in underlying molecular circuitry. With a closer look of miRNA hubs in LC of suicide group, it appears that miR-129, 29a, 1243, 101 and 361 are involved exclusively in suicide phenotype and associated pathophysiology. In contrast, majority of the hubs constructing miRNAs (e.g. miR-1227, 625, 598, 590-3p, 150, 744-3p, 28, 145, 340-3p, 29c and 657) from the uniquely patterned co-expression network of control subjects did not appear to be part of any miRNA list in the suicide group. This suggests that not only is the expression of miRNAs altered, but it is reorganized in the LC of suicide subjects. We also noted that miRNAs formed a very cohesive network in the control group where miRNAs were heavily interconnected with each other within the network, but this interconnection was comparatively much less intense in the suicide group. Collectively, our data indicates a unique mode of regulation of a set of miRNAs in the MDD-suicide group, which is completely different from its counterpart in the control group and possibly leading towards a dysfunctional regulatory network with major implication in suicidal behavior. A similar phenomenon was found in our earlier rodent study, where we observed that resiliency or vulnerability to develop stress-induced behavioral depression may be linked to specific miRNA co-expression network in the frontal cortex^41^.

Studying chromosomal localization of miRNA clusters helps understand coordinated regulation of miRNA expression under specific pathophysiological conditions^42^. In this study, we observed that miRNAs, whose chromosomal localizations were in proximity (e.g., miR-20b-5p, miR-106a-5p, miR-890 on chromosome X, miR-330-3p, miR-99b-3p on chromosome 19, and miR-409-5p, miR-1197 on chromosome 14), had the same direction of changes and similar fold changes. This happened even in those miRNAs whose transcriptional units were in opposite directions (miR-330-3p and miR-99b-3p on chromosome 19). This notion signifies the evolutionary conservation pattern of gene regulation which may culminate into similar functional output^43^. This also has relevance with the perspective of disease pathophysiology in which LC may be regulating functional gene networks in a cohesive manner by orchestrating the coordinated transcriptional output of the altered miRNAs.

*In silico* prediction of target genes for the 13 altered miRNAs in the LC of suicide subjects identified many genes with a range of diverse cellular functions involved in pathways associated with neuropsychiatric disorders. Several of these genes were found to be predicted as targets of more than one miRNA from the altered list. This manifold regulation of single target gene by multiple miRNAs is a unique mechanism of miRNA-mediated gene regulation^44^ and can be interpreted as dynamic regulation in brain areas like LC which is known to receive a diverse set of neurochemical inputs ranging from excitatory glutamatergic to inhibitory GABAergic pathways^35^. In this regard, our identification of target genes such as GRIA1, GRIA3, GRIK3, GRIN2b and GAD2 linked with glutamatergic neurotransmission in LC of suicide subjects raises the possibility of miRNA-mediated dysfunctional glutamatergic system. This is further consolidated by our finding of about 80% upregulation of GRIK1 gene expression in depressed-suicide LC samples. This finding correlates well with the earlier report of impaired glutamatergic pathway in LC of depressed brain where significant up regulation was noted in the function of GRIA1, GRIK1, GRM1, and GRM5 genes in addition to genes with a functional role in presynaptic vesicular transport of glutamate (VGLUT2 or SLC17A6)^45^. Similar evidence has been reported in the postmortem brain studies of altered glutamatergic input to noradrenergic LC in depressed-suicide brain^46^. In addition to glutamatergic pathway, several target genes associated with GABAergic neurotransmission (GABARB3, GABARA4, GABARA3, GABARR1, GABARG2 and GAD2) were found in LC of suicide subjects^47^, which implicates a strong relationship between miRNA-mediated GABAergic deficits and LC based noradrenergic activity. Studies from postmortem brain samples of depressed subjects have also found similar impairments in GABAergic neurotransmission where altered expression of GABA receptor subtypes such as α3, γ2, β1/3, and ε have been reported^48^. In addition, signaling mechanisms associated with protein kinase A, phospholipase C, glucocorticoid, ERK/MAPK, neurotrophins, GSK3-β, MAO, and CRH, were also predicated to participate in LC-associated neuropathological mechanisms in suicide^18, 30, 49^. All these signaling components have previously been reported by us to play critical roles in mood disorders and suicide^50–56^. Interestingly, *RELN* gene also appeared in the predicted target list with high context score. *RELN*, which plays a role in regulating neural migration during brain development as well as in dendritic maturation and dendritic spine development and whose expression and function changes in depression^57^, was also found to be a prominent target gene in the LC of suicide subjects. We tested a few of these genes to examine whether they correlate with miRNA expression. We found that RELN, GSK-3β, and MAOA were inversely correlated with miR-1179, miR-550a-5p, miR-1197, miR-330a-3p, miR-541a-3p, miR-582-5p, suggesting that these target genes are regulated by their corresponding miRNAs.

Taken together, our present study for the first time provides evidence of a transcriptome-wide altered miRNA expression in LC of suicide subjects and characterizes associated changes in miRNA and gene regulatory networks. Our study has the limitation that the number of subjects is low in both control and suicide groups; however, we were highly selective in choosing the samples that were very well characterized, had no known neuropathology, and were devoid of confounding variables such as substance abuse, alcohol or antidepressants treatment. Even with these low sample numbers, the results appear to be quite robust where we could identify significant gene and miRNA networks associated with suicidal behavior. The study in suicide subjects was conducted in the context of depression, which suggests that miRNA and gene networks in depressed individuals may be critical in the development of suicidal behavior. To further understand whether this is a general phenomenon of suicide, it will be required to further dissect our findings from depression to suicide which will require addition of suicide cases with other psychiatric disorders.

In conclusion, our present study substantially contributes to our understanding of how miRNAs and gene networks are reorganized in LC of suicide subjects and serves as a foundation to further explore more mechanistic studies. One example would be to conduct promoter-wide miRNA methylome study to find out the possible regulatory mechanism behind the altered transcription of miRNAs.

## Methods

### Subjects and postmortem tissue

The study was performed in locus coeruleus (LC) obtained from 10 non-psychiatric controls (referred as normal controls) and 9 depressed suicide subjects. Family members signed written informed consents and the study was approved by the Institutional Review Board of the University of Alabama at Birmingham and all methods were performed in accordance with the relevant guidelines and regulations. Brain tissues were collected according to the Lenhossek Human Brain Program, in the Human Brain Tissue Bank, Semmelweis University, Budapest, Hungary. The major advantage of using samples from this brain bank is that postmortem interval (PMI) is very short and RNA integrity numbers of the tissues are excellent. Psychiatric diagnoses of the subjects were made by means of the psychological autopsy method using DSM-IV^58^. The demographic and clinical characteristics of the subjects are provided in Table 1. Examination of medical records of controls subjects showed the absence of a history of psychiatric illnesses. Causes of death in control subjects were acute cardiac failure or myocardial infarction. All suicide subjects had a diagnosis of major depression and died by hanging or jumping from height. All subjects died suddenly and had a very short agonal state. Toxicology and presence of antidepressants were examined in blood samples obtained from these subjects. None of the subjects had positive antidepressant toxicology or substance abuse or alcohol. Suicide subjects included in the study were not taking antidepressants at least 2 month prior to death.

LC was dissected using the following procedure: after removal from the skull, the brains were cut in six major pieces (four cortical lobes, basal ganglia-diencephalon and lower brain stem-cerebellum), rapidly frozen on dry ice, and stored at −70 °C until dissection. At the time of the dissection the brain samples were sliced into 1–1.5 mm thick coronal sections at a temperature of 0–10 °C. The LC was recognized on two sequent sections in the rostral part of the pontine tegmentum, at the two sides of the fourth ventricle. Special microdissection needles^53, 54^ with 1.0 mm inside diameter were used to collect 4 tissue pellets containing LC. None of the control and suicide subjects showed evidence of abnormal neuropathology.

### RNA isolation

Total RNA was isolated from LC using a modified protocol^41^ designed to optimize recovery of small RNAs. Glycoblue 20 μg (Ambion, Waltham, MA, USA) was added to the RNA precipitation step, which was allowed to proceed overnight at −20 °C. The RNA pellet was spun down at 20,000 × g for 25 min at 4 °C; rinsed with 80% ethanol in nuclease-free water; resuspended in RNAsecure (Ambion, Waltham, MA, USA); and treated with DNase I using DNA-free kit (Ambion, Waltham, MA, USA). The purity and integrity of RNAs were determined by measuring the optical density with an absorbance ratio of 260/280 (NanoDrop spectrophotometer, ThermoScientific, Waltham, MA, USA) and running the samples on agarose gel. RNA integrity numbers were also determined in all samples.

### TLDA-based miRNA expression analysis

Expression of miRNAs was determined as described earlier^13^. Reverse transcription (RT) was performed following the manufacturer’s protocol with the TaqMan MiRNA Reverse Transcription kit (Applied Biosystems, Foster City, CA, USA) and the multiplex RT for TaqMan MicroRNA Assays that consisted of eight predefined RT primer pools. For each RT pool, 100 ng of total RNA was used and the product was diluted 1:62.5 and 55 μl diluted product mixed with 55 μl of TaqMan Universal PCR Master Mix (Applied Biosystems, Foster City, CA, USA), no AmpErase® UNG. One hundred microliter of each mix was dispensed in the appropriate well in the TaqMan Human MicroRNA Array v3.0 (Taqman low density array (TLDA), Applied Biosystems) and run for 40 cycles on an ABI 7900HT RT-PCR machine (Applied Biosystems). miRNAs were assayed on two plates, A and B; A plates contained many of the canonical miRNA sequences in miRBase, whereas the B plate primarily contained minor or star (*) miRNA sequences arising from the opposite arm of the pre-miR hairpin precursor. A sample processed without RT showed no detectable miRNA values. Using samples run on duplicate plates to monitor inter-plate reliability, we observed that Ct values > 35 were less reliable, so Ct ≤ 35 was set as the threshold of detectability. Median values (miRNAs and small RNAs) of each replicate was determined and used for normalization. We also checked geometric means of endogenous genes provided within the TLDA plates (U6, RNU44 and RNU48). The geometric means of these endogenous RNAs, which is represented as Ct values, did not change between healthy controls and suicide subjects (Plate A: Control: 22.55 ± 0.68; Suicide: 21.49 ± 0.51,﻿ Plate B: Control: 22.38 ± 0.88; Suicide: 22.40 ± 0.65). Plant-specific ath-miR-159a was included in the TLDA plate as negative control, which did not show any expression in human LC derived RNA. Fold-differences in miRNA expression across groups were calculated following ΔΔCt method as described earlier^59^. Based on median normalization, dCt values were determined for each miRNA and hierarchical clustering was performed to create a gene expression heat map using ClustVis (BETA) software^60^.

### Target genes and network analysis

Statistically significant miRNAs were analyzed for their mRNA targets using Ingenuity Pathway Analysis Software (IPA; Qiagen, Valencia, CA, USA). Briefly, the miRNA-target modules in IPA were used to filter a list of significantly altered up or downregulated miRNAs to retrieve a population of target mRNAs. Two sets of target mRNAs were analyzed. In one, mRNA target gene set was prepared using TarBase, Ingenuity Expert Finding and miRecords (Qiagen, Redwood City, CA, USA) with experimental validation. In another, mRNA target gene set was prepared based on number of conserved targeting site and total context score prediction value with a high-to-moderate degree of 3ʹ untranslated region-binding specificity with the miRNA seed sequence using TargetScan (Cambridge, MA, USA). The short-listed target genes were further analyzed with IPA core analysis module for functional enrichment of target genes deciphering their role in canonical pathways, molecular networks, and disease pathways using Fisher Exact Test. P-value threshold was set at ≤ 0.05. The initial data output from canonical pathway was filtered by setting the criteria stringently to represent only a few selected pathways related to stress and psychiatric disorders. Further analysis on predicted and validated target genes of differentially expressed miRNAs were carried out using mirPath (version 3) from DIANA Tools^61^ and CyTargetLinker plugin from Cytoscape software (version 3.0). The purpose of these advanced analyses was to find out affected pathways in relation to pathophysiological state and linking with the gene ontology (GO) of the target genes based on their molecular functions.

### Co-expression analysis

To identify pairs of miRNAs significantly correlated in the suicide group, but not in the control groups, we conducted co-expression analysis. In this analysis, miRNAs were first filtered to include only those who showed Ct values ≤ 35 in the two groups. The Ct values for each miRNAs were normalized to the global mean Ct value of the individual sample to exclude the false positive correlations caused by differences among individuals in overall miRNA content. Next, all miRNAs were detected pairwise and the Pearson correlation coefficient was determined for each group (control and suicide) separately. Note that for n = 9 for suicide and n = 11 for control subjects, only correlations of r = 0.7 or greater were significantly different from 0 at p = 0.05. Finally, we selected pairs of miRNAs that met the following criteria: 1) they demonstrated a significant positive correlation in the suicide group (r-suicide > 0.7); 2) the pairs did not exhibit a significant negative correlation in the control group (0.7 > r-control > −0.7); 3) correlation coefficients in the suicide group were higher than in the control group (r-suicide minus r-control > 0.8). A similar analysis was conducted in the control group to identify pairwise correlation in the control but not in the suicide group. We utilized the igraph package in R^62^ to display pairwise co-expression relationships with networks where nodes represented miRNAs and edges represented connected pairs of miRNAs.

### Expression analysis of select target genes by qPCR

To examine whether altered miRNAs were associated with changes in the expression of predicted target genes, we randomly selected six genes that show putative binding sites for certain significantly altered miRNAs and have relevance in depression and anxiety related pathophysiology. mRNA levels of these genes were determined by qPCR using EvaGreen/SybrGreen-based reaction chemistry (EvaGreen qPCR Mastermix, Applied Biological Materials, Richmond, Canada) as discussed above. The primer sequences are listed in Supplementary Table 1. Human specific GAPDH primer was used as endogenous control. The results were calculated using ΔΔCt method and reported as fold change.

### Statistical analysis

A total of 754 miRNAs were measured in TLDA plate; however, after excluding miRNAs with Ct value > 35, a total of 355 miRNAs were further analyzed. The non-parametric Wilcoxon sign-rank test, 2-tailed was utilized when making lists of miRNAs whose mean expression levels differed significantly across the two groups (control and suicide), since this test is appropriate for miRNAs whether or not they follow a normal distribution^63^. The Bonferroni correction of statistical significance values was not appropriate in this study because it assumes that the expression of the vast majority of genes is independent of each other. This situation does not apply in the case of miRNAs, which form extensive cross-correlation networks –in some cases due to co-transcription from the same primary gene transcripts^64, 65^. Instead, we performed SAM analysis (Significance Analysis of Microarrays software, version 3.08, Stanford University, http://www-stat.stanford.edu/~tibs/SAM/), which estimated statistical significance by subjecting the data to multiple random permutations; parameters were set for two-class unpaired analysis, Wilcoxon statistic and 5000 permutations^14, 66^. The correlation between miRNAs and various genes with PMI, age, and brain pH, were determined by Pearson product-moment correlation analysis. An α level ≤ 0.05 was considered significant.

### Data availability statement

All data generated or analyzed during this study are included in this published article (and its Supplementary Information files).

## Electronic supplementary material

Supplementary Information
