# Supplementary material for: Altered miRNA expression network in locus coeruleus of depressed suicide subjects
Source: Sci Rep. 2017 Jun 29;7:4387. doi: 10.1038/s41598-017-04300-9 (PMC5491496; doi:10.1038/s41598-017-04300-9)
Supplement: Supplementary file 1 — Supplementary Information [file 41598_2017_4300_MOESM1_ESM.pdf]

**Altered miRNA expression network in locus coeruleus of depressed suicide subjects**

Bhaskar Roy, Ph.D.,[1] Qingzhong Wang, Ph.D.,[1] Miklos Palkovits, M.D., Ph.D.,[2] Gabor Faludi, M.D., Ph.D.,[3] Yogesh Dwivedi, Ph.D.[1*]

[1]Department of Psychiatry and Behavioral Neurobiology, University of Alabama at Birmingham, Birmingham, Alabama, 35294, USA

[2]Human Brain Tissue Bank and Laboratory, Semmelweis University, Budapest, H-1094, Hungary

[3]Department of Psychiatry, Semmelweis University, Budapest, H-1125, Hungary

| Supplementary Table 1: Primer sequences | | |
|---|---|---|
| **Oligo Name** | **Sequence (5'-3')** | **Assay** |
| hsa-RELN RT F | CCCTTCTCAAGACCGAGTGT | Gene Expression |
| hsa-RELN RT R | GATCACAACTTTCACGGACACA | Gene Expression |
| hsa-CHRM1 RT F | GGCACAGCCAGAAACTGAAC | Gene Expression |
| hsa-CHRM1 RT R | GCCTATGAAGCGAGGGGATT | Gene Expression |
| hsa-PLCB1 RT F | GCGTCATCGGACTCTCTCTT | Gene Expression |
| hsa-PLCB1 RT R | AGGCTAAGGGATGCTTCTCG | Gene Expression |
| hsa-GSK3β RT F | AGGTGGCTCTTTGTTTGCCT | Gene Expression |
| hsa-GSK3β RT R | TGCTCAACACCCCTTCCATC | Gene Expression |
| hsa-MAOA RT F | GTAACTGCCCTGGGGTTTGT | Gene Expression |
| hsa-MAOA RT R | AAAACCAGTGAGCAGAGAGCA | Gene Expression |
| hsa-GRIK1 RT F | ATGGGGCGGTTAGAGATGGA | Gene Expression |
| hsa-GRIK1 RT R | CTGTTTCTTACCAGGGCGGT | Gene Expression |

**Table 2a:** Effect of age, PMI, RIN and age on expression of differentially regulated miRNAs in human post-mortem brains of healthy control subjects

| | **hsa-miR-17** | | **hsa-miR-20b** | | **hsa-miR-106a** | | **hsa-miR-330** | | **hsa-miR-409-5p** | | **hsa-miR-541** | | **hsa-miR-582-5p** | |
|---|---|---|---|---|---|---|---|---|---|---|---|---|---|---|
| **Variable** | **r** | **p** | **r** | **p** | **r** | **p** | **r** | **p** | **r** | **p** | **r** | **p** | **r** | **p** |
| **Age** | 0.324 | 0.332 | 0.432 | 0.185 | 0.303 | 0.364 | 0.243 | 0.472 | 0.266 | 0.43 | -0.55 | 0.077 | -0.203 | 0.549 |
| **PMI** | -0.542 | 0.085 | -0.578 | 0.063 | -0.507 | 0.111 | -0.502 | 0.115 | -0.029 | 0.932 | -0.42 | 0.205 | -0.09 | 0.793 |
| **RIN** | -0.223 | 0.51 | -0.135 | 0.692 | -0.28 | 0.405 | -0.32 | 0.338 | -0.184 | 0.589 | -0.38 | 0.247 | 0.016 | 0.962 |
| **pH** | 0.08 | 0.816 | 0.017 | 0.96 | 0.042 | 0.903 | -0.233 | 0.491 | 0.501 | 0.117 | 0.34 | 0.306 | -0.043 | 0.901 |

| | **hsa-miR-890** | | **hsa-let-7g** | | **hsa-miR-99b** | | **hsa-miR-550** | | **hsa-miR-1179** | | **hsa-miR-1197** | |
|---|---|---|---|---|---|---|---|---|---|---|---|---|
| **Variable** | **r** | **p** | **r** | **p** | **r** | **p** | **r** | **p** | **r** | **p** | **r** | **p** |
| **Age** | .660* | **0.027** | -0.24 | 0.478 | 0.32 | 0.337 | 0.202 | 0.552 | 0.028 | 0.936 | 0.367 | 0.267 |
| **PMI** | -0.546 | 0.082 | -0.301 | 0.368 | -0.189 | 0.578 | -0.439 | 0.177 | -0.318 | 0.341 | -0.49 | 0.123 |
| **RIN** | 0.151 | 0.658 | -.622* | **0.041** | -0.331 | 0.32 | -0.121 | 0.723 | 0.502 | 0.115 | -0.17 | 0.613 |
| **pH** | -0.128 | 0.708 | 0.333 | 0.318 | 0.109 | 0.75 | -0.456 | 0.159 | -0.547 | 0.082 | -0.09 | 0.802 |

PMI: post-mortem interval; RIN: RNA Integrity Number

**Table 2b:** Effect of age, PMI, RIN and pH on expression of target genes in human post-mortem brain of healthy control subjects

| | **RELN** | | **GSK-3β** | | **MAOA** | | **CHRM1** | | **PLCB1** | | **GRIK1** | |
|---|---|---|---|---|---|---|---|---|---|---|---|---|
| **Variable** | **r** | **p** | **r** | **p** | **r** | **p** | **r** | **p** | **r** | **p** | **r** | **p** |
| **Age** | 0.43 | 0.186 | -0.129 | 0.723 | -0.17 | 0.662 | 0.191 | 0.574 | 0.287 | 0.392 | .034 | .925 |
| **PMI** | 0.23 | 0.497 | 0.592 | 0.071 | .752* | **0.019** | 0.063 | 0.854 | 0.377 | 0.253 | -.356 | .312 |
| **RIN** | -0.038 | 0.912 | -0.007 | 0.985 | -0.056 | 0.887 | -0.365 | 0.27 | -.609* | **0.047** | 0.675* | **.032** |
| **pH** | -0.262 | 0.436 | -0.217 | 0.548 | 0.046 | 0.907 | 0.575 | 0.064 | .645* | **0.032** | -.587 | .074 |

PMI: post-mortem interval; RIN: RNA integrity number

**Supplementary Table 3: Predicted Targets of differentially regulated miRNAs**

| ID | Symbol | Source | Confidence | Symbol | Pathway |
|---|---|---|---|---|---|
| MIMAT0005824 | miR-1179 (miRNAs w/seed AGCAUUC) | TargetScan Human | Moderate (predicted) | ADD3 | Gαs Signaling,Protein Kinase A Signaling |
| MIMAT0005824 | miR-1179 (miRNAs w/seed AGCAUUC) | TargetScan Human | Moderate (predicted) | AKAP9 | cAMP-mediated signaling,Cardiac β-adrenergic Signaling,Protein Kinase A Signaling |
| MIMAT0005824 | miR-1179 (miRNAs w/seed AGCAUUC) | TargetScan Human | Moderate (predicted) | AP2B1 | Clathrin-mediated Endocytosis Signaling,CTLA4 Signaling in Cytotoxic T Lymphocytes,GABA Receptor Signaling,Lipid Antigen Presentation by CD1,Virus Entry via Endocytic Pathways |
| MIMAT0005824 | miR-1179 (miRNAs w/seed AGCAUUC) | TargetScan Human | Moderate (predicted) | ATM | 14-3-3-mediated Signaling,3-phosphoinositide Biosynthesis,Actin Cytoskeleton Signaling,Acute Myeloid Leukemia Signaling,Aldosterone Signaling in Epithelial Cells,AMPK Signaling,Amyotrophic Lateral Sclerosis Signaling,Angiopoietin Signaling,Antiproliferative Role of Somatostatin Receptor 2,Aryl Hydrocarbon Receptor Signaling,ATM Signaling,Axonal Guidance Signaling,B Cell Receptor Signaling,Breast Cancer Regulation by Stathmin1,Cardiac Hypertrophy Signaling,CCR3 Signaling in Eosinophils,CD28 Signaling in T Helper Cells,CD40 Signaling,Cell Cycle: G1/S Checkpoint Regulation,Cell Cycle: G2/M DNA Damage Checkpoint Regulation,Ceramide Signaling,Chronic Myeloid Leukemia Signaling,Clathrin-mediated Endocytosis Signaling,CNTF Signaling,Colorectal Cancer Metastasis Signaling,CREB Signaling in Neurons,CTLA4 Signaling in Cytotoxic T Lymphocytes,CXCR4 Signaling,Cyclins and Cell Cycle Regulation,Dendritic Cell Maturation,DNA damage-induced 14-3-3σ Signaling,DNA Double-Strand Break Repair by Homologous Recombination,DNA Double-Strand Break Repair by Non-Homologous End Joining,Docosahexaenoic Acid (DHA) Signaling,EGF Signaling,EIF2 Signaling,Endometrial Cancer Signaling,Endothelin-1 Signaling,eNOS Signaling,Ephrin A Signaling,ErbB Signaling,ErbB2-ErbB3 Signaling,ErbB4 Signaling,ERK/MAPK Signaling,Erythropoietin Signaling,Estrogen-Dependent Breast Cancer Signaling,FAK Signaling,Fc Epsilon RI Signaling,FcγRIIB Signaling in B Lymphocytes,FGF Signaling,FLT3 Signaling in Hematopoietic Progenitor Cells,fMLP Signaling in Neutrophils,G-Protein Coupled Receptor Signaling,GADD45 Signaling,Gap Junction Signaling,GDNF Family Ligand-Receptor Interactions,Germ Cell-Sertoli Cell Junction Signaling,Glioblastoma Multiforme Signaling,Glioma Invasiveness |

| | | | | | |
|---|---|---|---|---|---|
| | | | | | Signaling,Glioma Signaling,Glucocorticoid Receptor Signaling,GM-CSF Signaling,Growth Hormone Signaling,Gα12/13 Signaling,Gαq Signaling,HER-2 Signaling in Breast Cancer,Hereditary Breast Cancer Signaling,HGF Signaling,HIF1α Signaling,HMGB1 Signaling,Human Embryonic Stem Cell Pluripotency,Huntington's Disease Signaling,Hypoxia Signaling in the Cardiovascular System,iCOS-iCOSL Signaling in T Helper Cells,IGF-1 Signaling,IL-12 Signaling and Production in Macrophages,IL-15 Signaling,IL-17 Signaling,IL-17A Signaling in Airway Cells,IL-2 Signaling,IL-3 Signaling,IL-4 Signaling,IL-6 Signaling,IL-8 Signaling,IL-9 Signaling,ILK Signaling,Insulin Receptor Signaling,Integrin Signaling,JAK/Stat Signaling,Leptin Signaling in Obesity,Leukocyte Extravasation Signaling,LPS-stimulated MAPK Signaling,Lymphotoxin β Receptor Signaling,Macropinocytosis Signaling,Melanocyte Development and Pigmentation Signaling,Melanoma Signaling,Molecular Mechanisms of Cancer,Mouse Embryonic Stem Cell Pluripotency,MSP-RON Signaling Pathway,mTOR Signaling,Myc Mediated Apoptosis Signaling,Natural Killer Cell Signaling,Neuropathic Pain Signaling In Dorsal Horn Neurons,Neurotrophin/TRK Signaling,NF-κB Activation by Viruses,NF-κB Signaling,NGF Signaling,Nitric Oxide Signaling in the Cardiovascular System,Non-Small Cell Lung Cancer Signaling,NRF2-mediated Oxidative Stress Response,Ovarian Cancer Signaling,P2Y Purigenic Receptor Signaling Pathway,p53 Signaling,p70S6K Signaling,PAK Signaling,Pancreatic Adenocarcinoma Signaling,Paxillin Signaling,PDGF Signaling,PEDF Signaling,phagosome formation,PKCθ Signaling in T Lymphocytes,Production of Nitric Oxide and Reactive Oxygen Species in Macrophages,Prolactin Signaling,Prostate Cancer Signaling,Rac Signaling,RANK Signaling in Osteoclasts,Reelin Signaling in Neurons,Regulation of eIF4 and p70S6K Signaling,Regulation of the Epithelial-Mesenchymal Transition Pathway,Relaxin Signaling,Renal Cell Carcinoma Signaling,Renin-Angiotensin Signaling,Role of BRCA1 in DNA Damage Response,Role of CHK Proteins in Cell Cycle Checkpoint Control,Role of IL-17A in Arthritis,Role of JAK1 and JAK3 in γc Cytokine Signaling,Role of Macrophages, Fibroblasts and Endothelial Cells in Rheumatoid Arthritis,Role of NANOG in Mammalian Embryonic Stem Cell Pluripotency,Role of NFAT in Cardiac Hypertrophy,Role of NFAT in Regulation of the Immune Response,Role of Osteoblasts, Osteoclasts and Chondrocytes in Rheumatoid Arthritis,Role of p14/p19ARF in Tumor Suppression,Role of Pattern Recognition Receptors in Recognition of Bacteria and Viruses,Role of PI3K/AKT Signaling in the Pathogenesis of Influenza,Role of Tissue Factor in Cancer,SAPK/JNK Signaling,Signaling by Rho Family GTPases,Small Cell Lung Cancer Signaling,Sphingosine-1-phosphate Signaling,Superpathway of Inositol Phosphate Compounds,Systemic Lupus Erythematosus Signaling,T Cell Receptor Signaling,Tec Kinase |

| | | | | | |
|---|---|---|---|---|---|
| | | | | | Signaling,Telomerase Signaling,Thrombin Signaling,Thrombopoietin Signaling,TR/RXR Activation,Type II Diabetes Mellitus Signaling,UVA-Induced MAPK Signaling,UVB-Induced MAPK Signaling,VEGF Family Ligand-Receptor Interactions,VEGF Signaling,Virus Entry via Endocytic Pathways,Xenobiotic Metabolism Signaling |
| MIMAT0005824 | miR-1179 (miRNAs w/seed AGCAUUC) | TargetScan Human | Moderate (predicted) | BMP3 | Axonal Guidance Signaling,Basal Cell Carcinoma Signaling,BMP signaling pathway,Factors Promoting Cardiogenesis in Vertebrates,Human Embryonic Stem Cell Pluripotency,Molecular Mechanisms of Cancer,Role of NANOG in Mammalian Embryonic Stem Cell Pluripotency,Role of Osteoblasts, Osteoclasts and Chondrocytes in Rheumatoid Arthritis |
| MIMAT0005824 | miR-1179 (miRNAs w/seed AGCAUUC) | TargetScan Human | Moderate (predicted) | CAB39 | AMPK Signaling |
| MIMAT0005824 | miR-1179 (miRNAs w/seed AGCAUUC) | TargetScan Human | Moderate (predicted) | CABLES1 | CDK5 Signaling |
| MIMAT0005824 | miR-1179 (miRNAs w/seed AGCAUUC) | TargetScan Human | High (predicted) | CANX | Antigen Presentation Pathway,Lipid Antigen Presentation by CD1,phagosome maturation,Unfolded protein response |
| MIMAT0005824 | miR-1179 (miRNAs w/seed AGCAUUC) | TargetScan Human | Moderate (predicted) | CDH19 | Gα12/13 Signaling,RhoGDI Signaling,Signaling by Rho Family GTPases |
| MIMAT0005824 | miR-1179 (miRNAs w/seed AGCAUUC) | TargetScan Human | Moderate (predicted) | ELF3 | ERK/MAPK Signaling,HGF Signaling,Telomerase Signaling |
| MIMAT0005824 | miR-1179 (miRNAs w/seed AGCAUUC) | TargetScan Human | Moderate (predicted) | GABRG2 | GABA Receptor Signaling |
| MIMAT0005824 | miR-1179 (miRNAs w/seed AGCAUUC) | TargetScan Human | Moderate (predicted) | GHR | Growth Hormone Signaling,NF-κB Signaling,PPARα/RXRα Activation,PTEN Signaling,Role of JAK2 in Hormone-like Cytokine Signaling,STAT3 Pathway |
| MIMAT0005824 | miR-1179 (miRNAs w/seed | TargetScan Human | Moderate (predicted) | GLI2 | Axonal Guidance Signaling,Basal Cell Carcinoma Signaling,Corticotropin |

| | | | | | |
|---|---|---|---|---|---|
| | AGCAUUC) | | | | Releasing Hormone Signaling,Sonic Hedgehog Signaling |
| MIMAT0005824 | miR-1179 (miRNAs w/seed AGCAUUC) | TargetScan Human | Moderate (predicted) | GRIA3 | Amyotrophic Lateral Sclerosis Signaling,Calcium Signaling,CREB Signaling in Neurons,Glutamate Receptor Signaling,Neuropathic Pain Signaling In Dorsal Horn Neurons,Synaptic Long Term Depression,Synaptic Long Term Potentiation |
| MIMAT0005824 | miR-1179 (miRNAs w/seed AGCAUUC) | TargetScan Human | Moderate (predicted) | GRPR | GPCR-Mediated Integration of Enteroendocrine Signaling Exemplified by an L Cell |
| MIMAT0005824 | miR-1179 (miRNAs w/seed AGCAUUC) | TargetScan Human | Moderate (predicted) | HHIP | Axonal Guidance Signaling,Basal Cell Carcinoma Signaling,Sonic Hedgehog Signaling |
| MIMAT0005824 | miR-1179 (miRNAs w/seed AGCAUUC) | TargetScan Human | Moderate (predicted) | ISL1 | Embryonic Stem Cell Differentiation into Cardiac Lineages,Transcriptional Regulatory Network in Embryonic Stem Cells |
| MIMAT0005824 | miR-1179 (miRNAs w/seed AGCAUUC) | TargetScan Human | Moderate (predicted) | ITGA1 | Agranulocyte Adhesion and Diapedesis,Agrin Interactions at Neuromuscular Junction,Caveolar-mediated Endocytosis Signaling,Granulocyte Adhesion and Diapedesis,Integrin Signaling,Leukocyte Extravasation Signaling,NF-κB Activation by Viruses,Paxillin Signaling,Reelin Signaling in Neurons,Virus Entry via Endocytic Pathways |
| MIMAT0005824 | miR-1179 (miRNAs w/seed AGCAUUC) | TargetScan Human | Moderate (predicted) | ITGA6 | Agranulocyte Adhesion and Diapedesis,Agrin Interactions at Neuromuscular Junction,Caveolar-mediated Endocytosis Signaling,CDK5 Signaling,Germ Cell-Sertoli Cell Junction Signaling,Granulocyte Adhesion and Diapedesis,Integrin Signaling,Leukocyte Extravasation Signaling,NF-κB Activation by Viruses,Paxillin Signaling,Reelin Signaling in Neurons,Role of Tissue Factor in Cancer,Virus Entry via Endocytic Pathways |
| MIMAT0005824 | miR-1179 (miRNAs w/seed AGCAUUC) | TargetScan Human | High (predicted) | KCNJ1 | Dopamine-DARPP32 Feedback in cAMP Signaling |
| MIMAT0005824 | miR-1179 (miRNAs w/seed AGCAUUC) | TargetScan Human | High (predicted) | KCNJ16 | Dopamine-DARPP32 Feedback in cAMP Signaling |
| MIMAT0005824 | miR-1179 (miRNAs w/seed | TargetScan Human | Moderate (predicted) | LEF1 | Acute Myeloid Leukemia Signaling,Basal Cell Carcinoma Signaling,Colorectal Cancer Metastasis Signaling,Endometrial Cancer Signaling,Epithelial Adherens |

| | | | | | |
|---|---|---|---|---|---|
| | AGCAUUC) | | | | Junction Signaling,Factors Promoting Cardiogenesis in Vertebrates,Glioblastoma Multiforme Signaling,Human Embryonic Stem Cell Pluripotency,ILK Signaling,Molecular Mechanisms of Cancer,Mouse Embryonic Stem Cell Pluripotency,Ovarian Cancer Signaling,Prostate Cancer Signaling,Protein Kinase A Signaling,Regulation of the Epithelial-Mesenchymal Transition Pathway,Role of Macrophages, Fibroblasts and Endothelial Cells in Rheumatoid Arthritis,Role of Osteoblasts, Osteoclasts and Chondrocytes in Rheumatoid Arthritis,Role of Wnt/GSK-3β Signaling in the Pathogenesis of Influenza,Thyroid Cancer Signaling,Wnt/β-catenin Signaling |
| MIMAT0005824 | miR-1179 (miRNAs w/seed AGCAUUC) | TargetScan Human | Moderate (predicted) | LTB4R | cAMP-mediated signaling,Eicosanoid Signaling,G-Protein Coupled Receptor Signaling,Gαi Signaling |
| MIMAT0005824 | miR-1179 (miRNAs w/seed AGCAUUC) | TargetScan Human | Moderate (predicted) | MEF2C | B Cell Receptor Signaling,Calcium Signaling,Cardiac Hypertrophy Signaling,Cardiomyocyte Differentiation via BMP Receptors,Cholecystokinin/Gastrin-mediated Signaling,Corticotropin Releasing Hormone Signaling,ERK5 Signaling,Factors Promoting Cardiogenesis in Vertebrates,Gα12/13 Signaling,p38 MAPK Signaling,Phospholipase C Signaling,PPARα/RXRα Activation,Role of NFAT in Cardiac Hypertrophy,Role of NFAT in Regulation of the Immune Response |
| MIMAT0005824 | miR-1179 (miRNAs w/seed AGCAUUC) | TargetScan Human | Moderate (predicted) | NRP2 | Axonal Guidance Signaling,RhoA Signaling,VEGF Family Ligand-Receptor Interactions |
| MIMAT0005824 | miR-1179 (miRNAs w/seed AGCAUUC) | TargetScan Human | Moderate (predicted) | OS9 | Unfolded protein response |
| MIMAT0005824 | miR-1179 (miRNAs w/seed AGCAUUC) | TargetScan Human | Moderate (predicted) | PIK3C2A | 14-3-3-mediated Signaling,3-phosphoinositide Biosynthesis,Actin Cytoskeleton Signaling,Acute Myeloid Leukemia Signaling,Aldosterone Signaling in Epithelial Cells,AMPK Signaling,Amyotrophic Lateral Sclerosis Signaling,Angiopoietin Signaling,Antiproliferative Role of Somatostatin Receptor 2,Axonal Guidance Signaling,B Cell Receptor Signaling,Breast Cancer Regulation by Stathmin1,Cardiac Hypertrophy Signaling,CCR3 Signaling in Eosinophils,CD28 Signaling in T Helper Cells,CD40 Signaling,Ceramide Signaling,Chronic Myeloid Leukemia Signaling,Clathrin-mediated Endocytosis Signaling,CNTF Signaling,Colorectal Cancer Metastasis Signaling,CREB Signaling in Neurons,CTLA4 Signaling in Cytotoxic T Lymphocytes,CXCR4 Signaling,Dendritic Cell Maturation,Docosahexaenoic Acid (DHA) |

| | | | | | |
|---|---|---|---|---|---|
| | | | | | Signaling,EGF Signaling,EIF2 Signaling,Endometrial Cancer Signaling,Endothelin-1 Signaling,eNOS Signaling,Ephrin A Signaling,ErbB Signaling,ErbB2-ErbB3 Signaling,ErbB4 Signaling,ERK/MAPK Signaling,Erythropoietin Signaling,Estrogen-Dependent Breast Cancer Signaling,FAK Signaling,Fc Epsilon RI Signaling,FcγRIIB Signaling in B Lymphocytes,FGF Signaling,FLT3 Signaling in Hematopoietic Progenitor Cells,fMLP Signaling in Neutrophils,G-Protein Coupled Receptor Signaling,Gap Junction Signaling,GDNF Family Ligand-Receptor Interactions,Germ Cell-Sertoli Cell Junction Signaling,Glioblastoma Multiforme Signaling,Glioma Invasiveness Signaling,Glioma Signaling,Glucocorticoid Receptor Signaling,GM-CSF Signaling,Growth Hormone Signaling,Gα12/13 Signaling,Gαq Signaling,HER-2 Signaling in Breast Cancer,Hereditary Breast Cancer Signaling,HGF Signaling,HIF1α Signaling,HMGB1 Signaling,Human Embryonic Stem Cell Pluripotency,Huntington's Disease Signaling,iCOS-iCOSL Signaling in T Helper Cells,IGF-1 Signaling,IL-12 Signaling and Production in Macrophages,IL-15 Signaling,IL-17 Signaling,IL-17A Signaling in Airway Cells,IL-2 Signaling,IL-3 Signaling,IL-4 Signaling,IL-6 Signaling,IL-8 Signaling,IL-9 Signaling,ILK Signaling,Insulin Receptor Signaling,Integrin Signaling,JAK/Stat Signaling,Leptin Signaling in Obesity,Leukocyte Extravasation Signaling,LPS-stimulated MAPK Signaling,Lymphotoxin β Receptor Signaling,Macropinocytosis Signaling,Melanocyte Development and Pigmentation Signaling,Melanoma Signaling,Molecular Mechanisms of Cancer,Mouse Embryonic Stem Cell Pluripotency,MSP-RON Signaling Pathway,mTOR Signaling,Myc Mediated Apoptosis Signaling,Natural Killer Cell Signaling,Neuropathic Pain Signaling In Dorsal Horn Neurons,Neurotrophin/TRK Signaling,NF-κB Activation by Viruses,NF-κB Signaling,NGF Signaling,Nitric Oxide Signaling in the Cardiovascular System,Non-Small Cell Lung Cancer Signaling,NRF2-mediated Oxidative Stress Response,Ovarian Cancer Signaling,P2Y Purigenic Receptor Signaling Pathway,p53 Signaling,p70S6K Signaling,PAK Signaling,Pancreatic Adenocarcinoma Signaling,Paxillin Signaling,PDGF Signaling,PEDF Signaling,phagosome formation,PKCθ Signaling in T Lymphocytes,Production of Nitric Oxide and Reactive Oxygen Species in Macrophages,Prolactin Signaling,Prostate Cancer Signaling,Rac Signaling,RANK Signaling in Osteoclasts,Reelin Signaling in Neurons,Regulation of eIF4 and p70S6K Signaling,Regulation of the Epithelial-Mesenchymal Transition Pathway,Relaxin Signaling,Renal Cell Carcinoma Signaling,Renin-Angiotensin Signaling,Role of IL-17A in Arthritis,Role of JAK1 and JAK3 in γc Cytokine Signaling,Role of Macrophages, Fibroblasts and Endothelial Cells in Rheumatoid Arthritis,Role of NANOG in Mammalian Embryonic Stem Cell Pluripotency,Role of NFAT in Cardiac Hypertrophy,Role of NFAT in Regulation |

| | | | | | |
|---|---|---|---|---|---|
| | | | | | of the Immune Response,Role of Osteoblasts, Osteoclasts and Chondrocytes in Rheumatoid Arthritis,Role of p14/p19ARF in Tumor Suppression,Role of Pattern Recognition Receptors in Recognition of Bacteria and Viruses,Role of PI3K/AKT Signaling in the Pathogenesis of Influenza,Role of Tissue Factor in Cancer,SAPK/JNK Signaling,Signaling by Rho Family GTPases,Small Cell Lung Cancer Signaling,Sphingosine-1-phosphate Signaling,Superpathway of Inositol Phosphate Compounds,Systemic Lupus Erythematosus Signaling,T Cell Receptor Signaling,Tec Kinase Signaling,Telomerase Signaling,Thrombin Signaling,Thrombopoietin Signaling,TR/RXR Activation,Type II Diabetes Mellitus Signaling,UVA-Induced MAPK Signaling,UVB-Induced MAPK Signaling,VEGF Family Ligand-Receptor Interactions,VEGF Signaling,Virus Entry via Endocytic Pathways,Xenobiotic Metabolism Signaling |
| MIMAT0005824 | miR-1179 (miRNAs w/seed AGCAUUC) | TargetScan Human | Moderate (predicted) | PIP4K2A | 3-phosphoinositide Biosynthesis,Actin Cytoskeleton Signaling,Aldosterone Signaling in Epithelial Cells,D-myo-inositol (1,4,5)-Trisphosphate Biosynthesis,D-myo-inositol-5-phosphate Metabolism,Rac Signaling,Regulation of Actin-based Motility by Rho,RhoA Signaling,RhoGDI Signaling,Signaling by Rho Family GTPases,Superpathway of Inositol Phosphate Compounds |
| MIMAT0005824 | miR-1179 (miRNAs w/seed AGCAUUC) | TargetScan Human | Moderate (predicted) | PLCG1 | 14-3-3-mediated Signaling,Aldosterone Signaling in Epithelial Cells,Antioxidant Action of Vitamin C,Apoptosis Signaling,Axonal Guidance Signaling,Calcium-induced T Lymphocyte Apoptosis,Cardiac Hypertrophy Signaling,CCR5 Signaling in Macrophages,CD28 Signaling in T Helper Cells,Cellular Effects of Sildenafil (Viagra),Chemokine Signaling,Corticotropin Releasing Hormone Signaling,CREB Signaling in Neurons,CTLA4 Signaling in Cytotoxic T Lymphocytes,D-myo-inositol (1,4,5)-Trisphosphate Biosynthesis,D-myo-inositol-5-phosphate Metabolism,Dendritic Cell Maturation,Dopamine-DARPP32 Feedback in cAMP Signaling,EGF Signaling,Endothelin-1 Signaling,eNOS Signaling,ErbB Signaling,ErbB4 Signaling,ERK/MAPK Signaling,Erythropoietin Signaling,FAK Signaling,Fc Epsilon RI Signaling,Fcγ Receptor-mediated Phagocytosis in Macrophages and Monocytes,FGF Signaling,G Beta Gamma Signaling,G Protein Signaling Mediated by Tubby,Gap Junction Signaling,GDNF Family Ligand-Receptor Interactions,Glioblastoma Multiforme Signaling,Glioma Signaling,GPCR-Mediated Integration of Enteroendocrine Signaling Exemplified by an L Cell,GPCR-Mediated Nutrient Sensing in Enteroendocrine Cells,Growth Hormone Signaling,Gαq Signaling,HER-2 Signaling in Breast Cancer,HGF Signaling,iCOS-iCOSL Signaling in T Helper Cells,IL-15 Signaling,Integrin Signaling,Leptin Signaling in Obesity,Leukocyte Extravasation Signaling,Macropinocytosis Signaling,Melanocyte Development and |

| | | | | | |
|---|---|---|---|---|---|
| | | | | | Pigmentation Signaling,Melatonin Signaling,Natural Killer Cell Signaling,Neuregulin Signaling,Neuropathic Pain Signaling In Dorsal Horn Neurons,Neurotrophin/TRK Signaling,NGF Signaling,Non-Small Cell Lung Cancer Signaling,P2Y Purigenic Receptor Signaling Pathway,p70S6K Signaling,PDGF Signaling,phagosome formation,Phospholipase C Signaling,Phospholipases,PI3K Signaling in B Lymphocytes,PKCθ Signaling in T Lymphocytes,PPARα/RXRα Activation,Production of Nitric Oxide and Reactive Oxygen Species in Macrophages,Prolactin Signaling,Protein Kinase A Signaling,Regulation of IL-2 Expression in Activated and Anergic T Lymphocytes,Renin-Angiotensin Signaling,Role of Macrophages, Fibroblasts and Endothelial Cells in Rheumatoid Arthritis,Role of NFAT in Cardiac Hypertrophy,Role of NFAT in Regulation of the Immune Response,Sperm Motility,Sphingosine-1-phosphate Signaling,Superpathway of Inositol Phosphate Compounds,Synaptic Long Term Depression,Synaptic Long Term Potentiation,Systemic Lupus Erythematosus Signaling,T Cell Receptor Signaling,Tec Kinase Signaling,Thrombin Signaling,Thrombopoietin Signaling,TREM1 Signaling,UVA-Induced MAPK Signaling,VEGF Family Ligand-Receptor Interactions,VEGF Signaling,Virus Entry via Endocytic Pathways,Wnt/Ca+ pathway,α-Adrenergic Signaling |
| MIMAT0005824 | miR-1179 (miRNAs w/seed AGCAUUC) | TargetScan Human | Moderate (predicted) | PPP1R12A | 3-phosphoinositide Biosynthesis,3-phosphoinositide Degradation,Actin Cytoskeleton Signaling,Actin Nucleation by ARP-WASP Complex,Breast Cancer Regulation by Stathmin1,Cardiac β-adrenergic Signaling,CCR3 Signaling in Eosinophils,Cdc42 Signaling,CDK5 Signaling,Cellular Effects of Sildenafil (Viagra),Chemokine Signaling,D-myo-inositol (1,4,5,6)-Tetrakisphosphate Biosynthesis,D-myo-inositol (3,4,5,6)-tetrakisphosphate Biosynthesis,D-myo-inositol-5-phosphate Metabolism,Dopamine Receptor Signaling,Dopamine-DARPP32 Feedback in cAMP Signaling,ERK/MAPK Signaling,HIPPO signaling,ILK Signaling,Insulin Receptor Signaling,Integrin Signaling,Phospholipase C Signaling,Production of Nitric Oxide and Reactive Oxygen Species in Macrophages,Protein Kinase A Signaling,Regulation of Actin-based Motility by Rho,RhoA Signaling,RhoGDI Signaling,Signaling by Rho Family GTPases,Superpathway of Inositol Phosphate Compounds,Synaptic Long Term Potentiation,Thrombin Signaling |
| MIMAT0005824 | miR-1179 (miRNAs w/seed AGCAUUC) | TargetScan Human | High (predicted) | PPP2R2A | AMPK Signaling,Breast Cancer Regulation by Stathmin1,Cardiac β-adrenergic Signaling,CDK5 Signaling,Cell Cycle Regulation by BTG Family Proteins,Ceramide Signaling,CTLA4 Signaling in Cytotoxic T Lymphocytes,Cyclins and Cell Cycle Regulation,Dopamine Receptor Signaling,Dopamine-DARPP32 Feedback in cAMP Signaling,ERK/MAPK Signaling,HIPPO signaling,ILK Signaling,Mitotic Roles of Polo-Like |

| | | | | | |
|---|---|---|---|---|---|
| | | | | | Kinase,mTOR Signaling,p70S6K Signaling,PI3K/AKT Signaling,Production of Nitric Oxide and Reactive Oxygen Species in Macrophages,Regulation of eIF4 and p70S6K Signaling,Role of CHK Proteins in Cell Cycle Checkpoint Control,Synaptic Long Term Depression,Telomerase Signaling,Tight Junction Signaling,Wnt/β-catenin Signaling,Xenobiotic Metabolism Signaling |
| MIMAT0005824 | miR-1179 (miRNAs w/seed AGCAUUC) | TargetScan Human | Moderate (predicted) | PPP3R2 | Axonal Guidance Signaling,B Cell Receptor Signaling,Calcium Signaling,Calcium-induced T Lymphocyte Apoptosis,cAMP-mediated signaling,Cardiac Hypertrophy Signaling,CD28 Signaling in T Helper Cells,Clathrin-mediated Endocytosis Signaling,Dopamine-DARPP32 Feedback in cAMP Signaling,fMLP Signaling in Neutrophils,Gap Junction Signaling,Glucocorticoid Receptor Signaling,GM-CSF Signaling,Gαq Signaling,iCOS-iCOSL Signaling in T Helper Cells,IL-3 Signaling,Netrin Signaling,nNOS Signaling in Neurons,Nur77 Signaling in T Lymphocytes,Phospholipase C Signaling,PI3K Signaling in B Lymphocytes,PKCθ Signaling in T Lymphocytes,Protein Kinase A Signaling,RANK Signaling in Osteoclasts,Regulation of IL-2 Expression in Activated and Anergic T Lymphocytes,Role of Macrophages, Fibroblasts and Endothelial Cells in Rheumatoid Arthritis,Role of NFAT in Cardiac Hypertrophy,Role of NFAT in Regulation of the Immune Response,Role of Osteoblasts, Osteoclasts and Chondrocytes in Rheumatoid Arthritis,Synaptic Long Term Potentiation,T Cell Receptor Signaling |
| MIMAT0005824 | miR-1179 (miRNAs w/seed AGCAUUC) | TargetScan Human | Moderate (predicted) | PRKAB1 | AMPK Signaling,eNOS Signaling,Glucocorticoid Receptor Signaling,mTOR Signaling,PPARα/RXRα Activation,Type II Diabetes Mellitus Signaling |
| MIMAT0005824 | miR-1179 (miRNAs w/seed AGCAUUC) | TargetScan Human | Moderate (predicted) | PRKCB | 14-3-3-mediated Signaling,Aldosterone Signaling in Epithelial Cells,Androgen Signaling,Axonal Guidance Signaling,B Cell Receptor Signaling,Breast Cancer Regulation by Stathmin1,Calcium-induced T Lymphocyte Apoptosis,CCR3 Signaling in Eosinophils,CCR5 Signaling in Macrophages,Chemokine Signaling,Cholecystokinin/Gastrin-mediated Signaling,Corticotropin Releasing Hormone Signaling,CREB Signaling in Neurons,CXCR4 Signaling,Dopamine-DARPP32 Feedback in cAMP Signaling,Endothelin-1 Signaling,eNOS Signaling,ErbB Signaling,ErbB4 Signaling,ERK/MAPK Signaling,Erythropoietin Signaling,Factors Promoting Cardiogenesis in Vertebrates,Fc Epsilon RI Signaling,Fcγ Receptor-mediated Phagocytosis in Macrophages and Monocytes,fMLP Signaling in Neutrophils,G Beta Gamma Signaling,G-Protein Coupled Receptor Signaling,Gap Junction Signaling,Glioma Signaling,GM-CSF Signaling,GNRH Signaling,GPCR-Mediated Nutrient Sensing in Enteroendocrine Cells,Growth Hormone Signaling,Gαq Signaling,Hepatic |

| | | | | | |
|---|---|---|---|---|---|
| | | | | | Cholestasis,HER-2 Signaling in Breast Cancer,HGF Signaling,Huntington's Disease Signaling,IL-12 Signaling and Production in Macrophages,IL-3 Signaling,IL-8 Signaling,Leukocyte Extravasation Signaling,LPS-stimulated MAPK Signaling,Macropinocytosis Signaling,Mechanisms of Viral Exit from Host Cells,Melatonin Signaling,Molecular Mechanisms of Cancer,mTOR Signaling,Natural Killer Cell Signaling,Neuregulin Signaling,Neuropathic Pain Signaling In Dorsal Horn Neurons,NF-κB Activation by Viruses,NF-κB Signaling,Nitric Oxide Signaling in the Cardiovascular System,nNOS Signaling in Neurons,NRF2-mediated Oxidative Stress Response,P2Y Purigenic Receptor Signaling Pathway,p70S6K Signaling,PDGF Signaling,phagosome formation,Phospholipase C Signaling,PI3K Signaling in B Lymphocytes,PPARα/RXRα Activation,Production of Nitric Oxide and Reactive Oxygen Species in Macrophages,Prolactin Signaling,Protein Kinase A Signaling,RAR Activation,Renin-Angiotensin Signaling,Role of Macrophages, Fibroblasts and Endothelial Cells in Rheumatoid Arthritis,Role of NFAT in Cardiac Hypertrophy,Role of Pattern Recognition Receptors in Recognition of Bacteria and Viruses,Sperm Motility,Synaptic Long Term Depression,Synaptic Long Term Potentiation,Tec Kinase Signaling,Thrombin Signaling,Thrombopoietin Signaling,Type II Diabetes Mellitus Signaling,UVB-Induced MAPK Signaling,UVC-Induced MAPK Signaling,VDR/RXR Activation,VEGF Family Ligand-Receptor Interactions,VEGF Signaling,Virus Entry via Endocytic Pathways,Xenobiotic Metabolism Signaling,α-Adrenergic Signaling |
| MIMAT0005824 | miR-1179 (miRNAs w/seed AGCAUUC) | TargetScan Human | Moderate (predicted) | PTCH1 | Axonal Guidance Signaling,Basal Cell Carcinoma Signaling,Corticotropin Releasing Hormone Signaling,Molecular Mechanisms of Cancer,Protein Kinase A Signaling,Sonic Hedgehog Signaling |
| MIMAT0005824 | miR-1179 (miRNAs w/seed AGCAUUC) | TargetScan Human | Moderate (predicted) | PTGER3 | cAMP-mediated signaling,Colorectal Cancer Metastasis Signaling,Eicosanoid Signaling,G-Protein Coupled Receptor Signaling,Gαi Signaling |
| MIMAT0005824 | miR-1179 (miRNAs w/seed AGCAUUC) | TargetScan Human | Moderate (predicted) | PTPN22 | 3-phosphoinositide Biosynthesis,3-phosphoinositide Degradation,CTLA4 Signaling in Cytotoxic T Lymphocytes,D-myo-inositol (1,4,5,6)-Tetrakisphosphate Biosynthesis,D-myo-inositol (3,4,5,6)-tetrakisphosphate Biosynthesis,D-myo-inositol-5-phosphate Metabolism,Protein Kinase A Signaling,Superpathway of Inositol Phosphate Compounds |
| MIMAT0005824 | miR-1179 (miRNAs w/seed | TargetScan Human | Moderate (predicted) | PTPRB | Protein Kinase A Signaling |

| | | | | | |
|---|---|---|---|---|---|
| | AGCAUUC) | | | | |
| MIMAT0005824 | miR-1179 (miRNAs w/seed AGCAUUC) | TargetScan Human | Moderate (predicted) | PTPRM | 3-phosphoinositide Biosynthesis,3-phosphoinositide Degradation,D-myo-inositol (1,4,5,6)-Tetrakisphosphate Biosynthesis,D-myo-inositol (3,4,5,6)-tetrakisphosphate Biosynthesis,D-myo-inositol-5-phosphate Metabolism,Epithelial Adherens Junction Signaling,Protein Kinase A Signaling,Superpathway of Inositol Phosphate Compounds |
| MIMAT0005824 | miR-1179 (miRNAs w/seed AGCAUUC) | TargetScan Human | Moderate (predicted) | RASA1 | 3-phosphoinositide Biosynthesis,3-phosphoinositide Degradation,Angiopoietin Signaling,Axonal Guidance Signaling,Cdc42 Signaling,D-myo-inositol (1,4,5,6)-Tetrakisphosphate Biosynthesis,D-myo-inositol (3,4,5,6)-tetrakisphosphate Biosynthesis,D-myo-inositol-5-phosphate Metabolism,EGF Signaling,Ephrin Receptor Signaling,G-Protein Coupled Receptor Signaling,GDNF Family Ligand-Receptor Interactions,Gα12/13 Signaling,Huntington's Disease Signaling,IGF-1 Signaling,Molecular Mechanisms of Cancer,PDGF Signaling,Superpathway of Inositol Phosphate Compounds,T Cell Receptor Signaling |
| MIMAT0005824 | miR-1179 (miRNAs w/seed AGCAUUC) | TargetScan Human | Moderate (predicted) | RELN | Reelin Signaling in Neurons |
| MIMAT0005824 | miR-1179 (miRNAs w/seed AGCAUUC) | TargetScan Human | Moderate (predicted) | RHOT1 | Actin Nucleation by ARP-WASP Complex,Cardiac Hypertrophy Signaling,Cholecystokinin/Gastrin-mediated Signaling,Colorectal Cancer Metastasis Signaling,CXCR4 Signaling,Germ Cell-Sertoli Cell Junction Signaling,Glioblastoma Multiforme Signaling,Glioma Invasiveness Signaling,Gαq Signaling,HMGB1 Signaling,IL-8 Signaling,ILK Signaling,Integrin Signaling,Molecular Mechanisms of Cancer,mTOR Signaling,phagosome formation,Phospholipase C Signaling,Production of Nitric Oxide and Reactive Oxygen Species in Macrophages,Regulation of Actin-based Motility by Rho,RhoGDI Signaling,Semaphorin Signaling in Neurons,Signaling by Rho Family GTPases,Sphingosine-1-phosphate Signaling,Tec Kinase Signaling,Thrombin Signaling |
| MIMAT0005824 | miR-1179 (miRNAs w/seed AGCAUUC) | TargetScan Human | Moderate (predicted) | SH3GLB1 | Clathrin-mediated Endocytosis Signaling,Mechanisms of Viral Exit from Host Cells,Role of Oct4 in Mammalian Embryonic Stem Cell Pluripotency |
| MIMAT0005824 | miR-1179 (miRNAs w/seed AGCAUUC) | TargetScan Human | Moderate (predicted) | SLIT2 | Axonal Guidance Signaling |

| | | | | | |
|---|---|---|---|---|---|
| MIMAT0005824 | miR-1179 (miRNAs w/seed AGCAUUC) | TargetScan Human | Moderate (predicted) | SMAD4 | Antiproliferative Role of TOB in T Cell Signaling,BMP signaling pathway,Cardiomyocyte Differentiation via BMP Receptors,Cell Cycle: G1/S Checkpoint Regulation,Chronic Myeloid Leukemia Signaling,Colorectal Cancer Metastasis Signaling,Factors Promoting Cardiogenesis in Vertebrates,Glucocorticoid Receptor Signaling,Hepatic Fibrosis / Hepatic Stellate Cell Activation,HIPPO signaling,Human Embryonic Stem Cell Pluripotency,Molecular Mechanisms of Cancer,Mouse Embryonic Stem Cell Pluripotency,Pancreatic Adenocarcinoma Signaling,PPARα/RXRα Activation,Protein Kinase A Signaling,RAR Activation,Regulation of IL-2 Expression in Activated and Anergic T Lymphocytes,Regulation of the Epithelial-Mesenchymal Transition Pathway,Role of NANOG in Mammalian Embryonic Stem Cell Pluripotency,Role of Osteoblasts, Osteoclasts and Chondrocytes in Rheumatoid Arthritis,TGF-β Signaling |
| MIMAT0005824 | miR-1179 (miRNAs w/seed AGCAUUC) | TargetScan Human | Moderate (predicted) | SYVN1 | Unfolded protein response |
| MIMAT0005824 | miR-1179 (miRNAs w/seed AGCAUUC) | TargetScan Human | Moderate (predicted) | TAF1B | Assembly of RNA Polymerase I Complex |
| MIMAT0005824 | miR-1179 (miRNAs w/seed AGCAUUC) | TargetScan Human | Moderate (predicted) | TAF4B | Assembly of RNA Polymerase II Complex,Estrogen Receptor Signaling,Glucocorticoid Receptor Signaling |
| MIMAT0005824 | miR-1179 (miRNAs w/seed AGCAUUC) | TargetScan Human | High (predicted) | TGFBR2 | Antiproliferative Role of TOB in T Cell Signaling,Cardiac Hypertrophy Signaling,Chronic Myeloid Leukemia Signaling,Colorectal Cancer Metastasis Signaling,Epithelial Adherens Junction Signaling,Factors Promoting Cardiogenesis in Vertebrates,Germ Cell-Sertoli Cell Junction Signaling,Glucocorticoid Receptor Signaling,Hepatic Fibrosis / Hepatic Stellate Cell Activation,Human Embryonic Stem Cell Pluripotency,Inhibition of Angiogenesis by TSP1,Molecular Mechanisms of Cancer,NF-κB Signaling,p38 MAPK Signaling,Pancreatic Adenocarcinoma Signaling,PPARα/RXRα Activation,Protein Kinase A Signaling,PTEN Signaling,Regulation of IL-2 Expression in Activated and Anergic T Lymphocytes,Regulation of the Epithelial-Mesenchymal Transition Pathway,Role of NFAT in Cardiac Hypertrophy,STAT3 Pathway,T Helper Cell Differentiation,TGF-β Signaling,Tight Junction Signaling,Wnt/β-catenin Signaling |
| MIMAT0005824 | miR-1179 | TargetScan Human | Moderate (predicted) | TRPC6 | Calcium Signaling |

| | | | | | |
|---|---|---|---|---|---|
| | (miRNAs w/seed AGCAUUC) | | | | |
| MIMAT0005824 | miR-1179 (miRNAs w/seed AGCAUUC) | TargetScan Human | Moderate (predicted) | TUBA1A | 14-3-3-mediated Signaling,Axonal Guidance Signaling,Breast Cancer Regulation by Stathmin1,Epithelial Adherens Junction Signaling,Gap Junction Signaling,Germ Cell-Sertoli Cell Junction Signaling,phagosome maturation,Remodeling of Epithelial Adherens Junctions,Sertoli Cell-Sertoli Cell Junction Signaling |
| MIMAT0005824 | miR-1179 (miRNAs w/seed AGCAUUC) | TargetScan Human | Moderate (predicted) | UBXN4 | Unfolded protein response |
| MIMAT0005824 | miR-1179 (miRNAs w/seed AGCAUUC) | TargetScan Human | Moderate (predicted) | VIP | Circadian Rhythm Signaling,GPCR-Mediated Integration of Enteroendocrine Signaling Exemplified by an L Cell |
| MIMAT0005955 | miR-1197 (and other miRNAs w/seed AGGACAC) | TargetScan Human | Moderate (predicted) | ADCY1 | Breast Cancer Regulation by Stathmin1,cAMP-mediated signaling,Cardiac Hypertrophy Signaling,Cardiac β-adrenergic Signaling,CDK5 Signaling,Cellular Effects of Sildenafil (Viagra),Colorectal Cancer Metastasis Signaling,Corticotropin Releasing Hormone Signaling,CREB Signaling in Neurons,CXCR4 Signaling,Dopamine Receptor Signaling,Dopamine-DARPP32 Feedback in cAMP Signaling,Endothelin-1 Signaling,eNOS Signaling,G Beta Gamma Signaling,G-Protein Coupled Receptor Signaling,GABA Receptor Signaling,Gap Junction Signaling,GNRH Signaling,GPCR-Mediated Integration of Enteroendocrine Signaling Exemplified by an L Cell,GPCR-Mediated Nutrient Sensing in Enteroendocrine Cells,Gustation Pathway,Gαi Signaling,Gαs Signaling,Hepatic Cholestasis,IL-1 Signaling,Leptin Signaling in Obesity,Melanocyte Development and Pigmentation Signaling,Molecular Mechanisms of Cancer,P2Y Purigenic Receptor Signaling Pathway,Phospholipase C Signaling,PPARα/RXRα Activation,Protein Kinase A Signaling,RAR Activation,Relaxin Signaling,Renin-Angiotensin Signaling,Role of NFAT in Cardiac Hypertrophy,Serotonin Receptor Signaling,Sphingosine-1-phosphate Signaling,Synaptic Long Term Potentiation,Thrombin Signaling,α-Adrenergic Signaling |
| MIMAT0005955 | miR-1197 (and other miRNAs w/seed AGGACAC) | TargetScan Human | High (predicted) | ADD3 | Gαs Signaling,Protein Kinase A Signaling |

| | | | | | |
|---|---|---|---|---|---|
| MIMAT0005955 | miR-1197 (and other miRNAs w/seed AGGACAC) | TargetScan Human | Moderate (predicted) | AK2 | AMPK Signaling |
| MIMAT0005955 | miR-1197 (and other miRNAs w/seed AGGACAC) | TargetScan Human | Moderate (predicted) | AKAP5 | Calcium Signaling,cAMP-mediated signaling,Cardiac β-adrenergic Signaling,Protein Kinase A Signaling,Role of NFAT in Cardiac Hypertrophy,Role of NFAT in Regulation of the Immune Response |
| MIMAT0005955 | miR-1197 (and other miRNAs w/seed AGGACAC) | TargetScan Human | Moderate (predicted) | ANAPC13 | Mitotic Roles of Polo-Like Kinase,Protein Kinase A Signaling |
| MIMAT0005955 | miR-1197 (and other miRNAs w/seed AGGACAC) | TargetScan Human | Moderate (predicted) | ARID2 | AMPK Signaling,Glucocorticoid Receptor Signaling,Hereditary Breast Cancer Signaling,RAR Activation,Role of BRCA1 in DNA Damage Response |
| MIMAT0005955 | miR-1197 (and other miRNAs w/seed AGGACAC) | TargetScan Human | Moderate (predicted) | BRF1 | Assembly of RNA Polymerase III Complex |
| MIMAT0005955 | miR-1197 (and other miRNAs w/seed AGGACAC) | TargetScan Human | Moderate (predicted) | CAB39 | AMPK Signaling |
| MIMAT0005955 | miR-1197 (and other miRNAs w/seed AGGACAC) | TargetScan Human | Moderate (predicted) | CAMKK2 | AMPK Signaling,Calcium Signaling,Dopamine-DARPP32 Feedback in cAMP Signaling |
| MIMAT0005955 | miR-1197 (and other miRNAs w/seed AGGACAC) | TargetScan Human | Moderate (predicted) | CAPN5 | Amyloid Processing,Amyotrophic Lateral Sclerosis Signaling,Apoptosis Signaling,FAK Signaling,Huntington's Disease Signaling,Integrin Signaling,nNOS Signaling in Neurons,Regulation of Cellular Mechanics by Calpain Protease |
| MIMAT0005955 | miR-1197 (and other miRNAs | TargetScan Human | High (predicted) | CBL | 14-3-3-mediated Signaling,Clathrin-mediated Endocytosis Signaling,Ephrin B Signaling,Erythropoietin Signaling,Fcγ Receptor-mediated Phagocytosis in |

| | | | | | |
|---|---|---|---|---|---|
| | w/seed AGGACAC) | | | | Macrophages and Monocytes,FLT3 Signaling in Hematopoietic Progenitor Cells,Insulin Receptor Signaling,Molecular Mechanisms of Cancer,PI3K Signaling in B Lymphocytes,Protein Ubiquitination Pathway,PTEN Signaling,RANK Signaling in Osteoclasts,Role of Osteoblasts, Osteoclasts and Chondrocytes in Rheumatoid Arthritis,Systemic Lupus Erythematosus Signaling,T Cell Receptor Signaling |
| MIMAT0005955 | miR-1197 (and other miRNAs w/seed AGGACAC) | TargetScan Human | Moderate (predicted) | CD79B | Altered T Cell and B Cell Signaling in Rheumatoid Arthritis,B Cell Development,B Cell Receptor Signaling,FcγRIIB Signaling in B Lymphocytes,p70S6K Signaling,Phospholipase C Signaling,PI3K Signaling in B Lymphocytes,Role of NFAT in Regulation of the Immune Response,Systemic Lupus Erythematosus Signaling |
| MIMAT0005955 | miR-1197 (and other miRNAs w/seed AGGACAC) | TargetScan Human | High (predicted) | CDC25B | 3-phosphoinositide Biosynthesis,3-phosphoinositide Degradation,Cell Cycle: G2/M DNA Damage Checkpoint Regulation,D-myo-inositol (1,4,5,6)-Tetrakisphosphate Biosynthesis,D-myo-inositol (3,4,5,6)-tetrakisphosphate Biosynthesis,D-myo-inositol-5-phosphate Metabolism,Mitotic Roles of Polo-Like Kinase,Molecular Mechanisms of Cancer,p38 MAPK Signaling,Protein Kinase A Signaling,Superpathway of Inositol Phosphate Compounds |
| MIMAT0005955 | miR-1197 (and other miRNAs w/seed AGGACAC) | TargetScan Human | Moderate (predicted) | CHRM1 | cAMP-mediated signaling,G-Protein Coupled Receptor Signaling,Gαq Signaling,Gαs Signaling,Nitric Oxide Signaling in the Cardiovascular System |
| MIMAT0005955 | miR-1197 (and other miRNAs w/seed AGGACAC) | TargetScan Human | Moderate (predicted) | CHRM2 | cAMP-mediated signaling,G-Protein Coupled Receptor Signaling,GPCR-Mediated Integration of Enteroendocrine Signaling Exemplified by an L Cell,Gαi Signaling |
| MIMAT0005955 | miR-1197 (and other miRNAs w/seed AGGACAC) | TargetScan Human | Moderate (predicted) | COMT | Dopamine Degradation,Dopamine Receptor Signaling,L-DOPA Degradation,Noradrenaline and Adrenaline Degradation |
| MIMAT0005955 | miR-1197 (and other miRNAs w/seed AGGACAC) | TargetScan Human | High (predicted) | COPS5 | Axonal Guidance Signaling,HIF1α Signaling,Hypoxia Signaling in the Cardiovascular System |
| MIMAT0005955 | miR-1197 (and other miRNAs | TargetScan Human | High (predicted),Moderate | CREM | cAMP-mediated signaling,Cholecystokinin/Gastrin-mediated Signaling,Dopamine-DARPP32 Feedback in cAMP Signaling,Protein Kinase A |

| | | | | | |
|---|---|---|---|---|---|
| | w/seed AGGACAC) | | (predicted) | | Signaling,Systemic Lupus Erythematosus Signaling |
| MIMAT0005955 | miR-1197 (and other miRNAs w/seed AGGACAC) | TargetScan Human | Moderate (predicted) | DCX | Reelin Signaling in Neurons |
| MIMAT0005955 | miR-1197 (and other miRNAs w/seed AGGACAC) | TargetScan Human | Moderate (predicted) | EPHB4 | Axonal Guidance Signaling,Ephrin B Signaling,Ephrin Receptor Signaling |
| MIMAT0005955 | miR-1197 (and other miRNAs w/seed AGGACAC) | TargetScan Human | Moderate (predicted) | ERBB2 | Agrin Interactions at Neuromuscular Junction,Axonal Guidance Signaling,Bladder Cancer Signaling,Endometrial Cancer Signaling,ErbB Signaling,ErbB2-ErbB3 Signaling,HER-2 Signaling in Breast Cancer,Neuregulin Signaling,Non-Small Cell Lung Cancer Signaling,Pancreatic Adenocarcinoma Signaling |
| MIMAT0005955 | miR-1197 (and other miRNAs w/seed AGGACAC) | TargetScan Human | High (predicted) | FCER1G | Allograft Rejection Signaling,Altered T Cell and B Cell Signaling in Rheumatoid Arthritis,Autoimmune Thyroid Disease Signaling,Calcium-induced T Lymphocyte Apoptosis,CCR5 Signaling in Macrophages,CD28 Signaling in T Helper Cells,Cdc42 Signaling,Communication between Innate and Adaptive Immune Cells,CTLA4 Signaling in Cytotoxic T Lymphocytes,Cytotoxic T Lymphocyte-mediated Apoptosis of Target Cells,Dendritic Cell Maturation,Fc Epsilon RI Signaling,Graft-versus-Host Disease Signaling,Hematopoiesis from Pluripotent Stem Cells,iCOS-iCOSL Signaling in T Helper Cells,Lipid Antigen Presentation by CD1,Natural Killer Cell Signaling,NF-κB Signaling,Nur77 Signaling in T Lymphocytes,OX40 Signaling Pathway,phagosome formation,Phospholipase C Signaling,PKCθ Signaling in T Lymphocytes,Role of NFAT in Regulation of the Immune Response,SAPK/JNK Signaling,Systemic Lupus Erythematosus Signaling,T Helper Cell Differentiation,Tec Kinase Signaling,Type I Diabetes Mellitus Signaling |
| MIMAT0005955 | miR-1197 (and other miRNAs w/seed AGGACAC) | TargetScan Human | Moderate (predicted) | FRS2 | FGF Signaling,GDNF Family Ligand-Receptor Interactions,Neurotrophin/TRK Signaling,Regulation of the Epithelial-Mesenchymal Transition Pathway |
| MIMAT0005955 | miR-1197 (and other miRNAs | TargetScan Human | Moderate (predicted) | GABRG2 | GABA Receptor Signaling |

| | | | | | |
|---|---|---|---|---|---|
| | w/seed AGGACAC) | | | | |
| MIMAT0005955 | miR-1197 (and other miRNAs w/seed AGGACAC) | TargetScan Human | Moderate (predicted) | GNA13 | Actin Cytoskeleton Signaling,Androgen Signaling,Axonal Guidance Signaling,Breast Cancer Regulation by Stathmin1,Cardiac Hypertrophy Signaling,Cholecystokinin/Gastrin-mediated Signaling,CREB Signaling in Neurons,CXCR4 Signaling,Endothelin-1 Signaling,Ephrin B Signaling,Ephrin Receptor Signaling,ERK5 Signaling,G Beta Gamma Signaling,Gα12/13 Signaling,IL-1 Signaling,IL-8 Signaling,Molecular Mechanisms of Cancer,Phospholipase C Signaling,Protein Kinase A Signaling,Relaxin Signaling,RhoA Signaling,RhoGDI Signaling,Role of NFAT in Regulation of the Immune Response,Role of Tissue Factor in Cancer,SAPK/JNK Signaling,Signaling by Rho Family GTPases,Sphingosine-1-phosphate Signaling,Synaptic Long Term Depression,Tec Kinase Signaling,Thrombin Signaling |
| MIMAT0005955 | miR-1197 (and other miRNAs w/seed AGGACAC) | TargetScan Human | Moderate (predicted) | GNRHR | GNRH Signaling |
| MIMAT0005955 | miR-1197 (and other miRNAs w/seed AGGACAC) | TargetScan Human | Moderate (predicted) | GRIK3 | Amyotrophic Lateral Sclerosis Signaling,CREB Signaling in Neurons,Glutamate Receptor Signaling |
| MIMAT0005955 | miR-1197 (and other miRNAs w/seed AGGACAC) | TargetScan Human | Moderate (predicted) | GRIP1 | Glutamate Receptor Signaling,RhoGDI Signaling,Xenobiotic Metabolism Signaling |
| MIMAT0005955 | miR-1197 (and other miRNAs w/seed AGGACAC) | TargetScan Human | Moderate (predicted) | GUCY2C | Antiproliferative Role of Somatostatin Receptor 2,Cellular Effects of Sildenafil (Viagra),Corticotropin Releasing Hormone Signaling,Endothelin-1 Signaling,Gap Junction Signaling,Nitric Oxide Signaling in the Cardiovascular System,Phototransduction Pathway,Relaxin Signaling,Synaptic Long Term Depression |
| MIMAT0005955 | miR-1197 (and other miRNAs w/seed AGGACAC) | TargetScan Human | Moderate (predicted) | H1F0 | Granzyme A Signaling,Protein Kinase A Signaling |

| | | | | | |
|---|---|---|---|---|---|
| MIMAT0005955 | miR-1197 (and other miRNAs w/seed AGGACAC) | TargetScan Human | Moderate (predicted) | HDAC5 | Adipogenesis pathway,Calcium Signaling,Cell Cycle: G1/S Checkpoint Regulation,Chronic Myeloid Leukemia Signaling,Cyclins and Cell Cycle Regulation,Hereditary Breast Cancer Signaling,Huntington's Disease Signaling,Phospholipase C Signaling,Role of NFAT in Cardiac Hypertrophy,Telomerase Signaling,Xenobiotic Metabolism Signaling |
| MIMAT0005955 | miR-1197 (and other miRNAs w/seed AGGACAC) | TargetScan Human | Moderate (predicted) | HMGB1 | Glucocorticoid Receptor Signaling,HMGB1 Signaling |
| MIMAT0005955 | miR-1197 (and other miRNAs w/seed AGGACAC) | TargetScan Human | Moderate (predicted) | HTR1D | cAMP-mediated signaling,G-Protein Coupled Receptor Signaling,Gαi Signaling,Serotonin Receptor Signaling |
| MIMAT0005955 | miR-1197 (and other miRNAs w/seed AGGACAC) | TargetScan Human | Moderate (predicted) | ITGA3 | Actin Cytoskeleton Signaling,Actin Nucleation by ARP-WASP Complex,Agranulocyte Adhesion and Diapedesis,Agrin Interactions at Neuromuscular Junction,Axonal Guidance Signaling,Caveolar-mediated Endocytosis Signaling,Cdc42 Signaling,CDK5 Signaling,Ephrin Receptor Signaling,ERK/MAPK Signaling,FAK Signaling,Germ Cell-Sertoli Cell Junction Signaling,Granulocyte Adhesion and Diapedesis,HGF Signaling,Integrin Signaling,Leukocyte Extravasation Signaling,Molecular Mechanisms of Cancer,Neuregulin Signaling,NF-κB Activation by Viruses,PAK Signaling,Paxillin Signaling,phagosome formation,Phospholipase C Signaling,PI3K/AKT Signaling,PTEN Signaling,Rac Signaling,Reelin Signaling in Neurons,Regulation of Actin-based Motility by Rho,Regulation of Cellular Mechanics by Calpain Protease,Regulation of eIF4 and p70S6K Signaling,RhoGDI Signaling,Role of Osteoblasts, Osteoclasts and Chondrocytes in Rheumatoid Arthritis,Role of Tissue Factor in Cancer,Sertoli Cell-Sertoli Cell Junction Signaling,Signaling by Rho Family GTPases,Tec Kinase Signaling,Virus Entry via Endocytic Pathways |
| MIMAT0005955 | miR-1197 (and other miRNAs w/seed AGGACAC) | TargetScan Human | Moderate (predicted) | ITGB3 | Agrin Interactions at Neuromuscular Junction,Caveolar-mediated Endocytosis Signaling,Clathrin-mediated Endocytosis Signaling,Glioma Invasiveness Signaling,Granulocyte Adhesion and Diapedesis,HER-2 Signaling in Breast Cancer,IL-8 Signaling,ILK Signaling,Integrin Signaling,Leukocyte Extravasation Signaling,Macropinocytosis Signaling,NF-κB Activation by Viruses,P2Y Purigenic Receptor Signaling Pathway,Paxillin Signaling,Reelin Signaling in Neurons,Role of Osteoblasts, Osteoclasts and Chondrocytes in Rheumatoid Arthritis,Role of Tissue Factor in Cancer,Virus Entry via Endocytic Pathways |

| | | | | | |
|---|---|---|---|---|---|
| MIMAT0005955 | miR-1197 (and other miRNAs w/seed AGGACAC) | TargetScan Human | Moderate (predicted) | ITPR1 | Aldosterone Signaling in Epithelial Cells,Breast Cancer Regulation by Stathmin1,Calcium Signaling,Calcium-induced T Lymphocyte Apoptosis,CCR3 Signaling in Eosinophils,CD28 Signaling in T Helper Cells,Cellular Effects of Sildenafil (Viagra),Cholecystokinin/Gastrin-mediated Signaling,Corticotropin Releasing Hormone Signaling,CREB Signaling in Neurons,CXCR4 Signaling,Dopamine-DARPP32 Feedback in cAMP Signaling,EGF Signaling,Endothelin-1 Signaling,eNOS Signaling,FGF Signaling,fMLP Signaling in Neutrophils,Gap Junction Signaling,GDNF Family Ligand-Receptor Interactions,Glioblastoma Multiforme Signaling,GNRH Signaling,GPCR-Mediated Integration of Enteroendocrine Signaling Exemplified by an L Cell,GPCR-Mediated Nutrient Sensing in Enteroendocrine Cells,Gustation Pathway,Gαq Signaling,Huntington's Disease Signaling,iCOS-iCOSL Signaling in T Helper Cells,Neuropathic Pain Signaling In Dorsal Horn Neurons,Nitric Oxide Signaling in the Cardiovascular System,Non-Small Cell Lung Cancer Signaling,Phospholipase C Signaling,PI3K Signaling in B Lymphocytes,Protein Kinase A Signaling,Renin-Angiotensin Signaling,Role of NFAT in Cardiac Hypertrophy,Role of NFAT in Regulation of the Immune Response,Sperm Motility,Synaptic Long Term Depression,Synaptic Long Term Potentiation,Thrombin Signaling,α-Adrenergic Signaling |
| MIMAT0005955 | miR-1197 (and other miRNAs w/seed AGGACAC) | TargetScan Human | Moderate (predicted) | JAK2 | Acute Phase Response Signaling,Antioxidant Action of Vitamin C,CNTF Signaling,Colorectal Cancer Metastasis Signaling,CTLA4 Signaling in Cytotoxic T Lymphocytes,Dendritic Cell Maturation,Ephrin Receptor Signaling,Erythropoietin Signaling,G Protein Signaling Mediated by Tubby,Glucocorticoid Receptor Signaling,GM-CSF Signaling,Growth Hormone Signaling,IGF-1 Signaling,IL-15 Production,IL-15 Signaling,IL-17 Signaling,IL-17A Signaling in Airway Cells,IL-3 Signaling,IL-4 Signaling,IL-6 Signaling,iNOS Signaling,Insulin Receptor Signaling,Interferon Signaling,JAK/Stat Signaling,Leptin Signaling in Obesity,Molecular Mechanisms of Cancer,Mouse Embryonic Stem Cell Pluripotency,MSP-RON Signaling Pathway,Oncostatin M Signaling,Pancreatic Adenocarcinoma Signaling,PDGF Signaling,PI3K/AKT Signaling,PPARα/RXRα Activation,Production of Nitric Oxide and Reactive Oxygen Species in Macrophages,Prolactin Signaling,RAR Activation,Regulation of the Epithelial-Mesenchymal Transition Pathway,Renin-Angiotensin Signaling,Role of JAK family kinases in IL-6-type Cytokine Signaling,Role of JAK1 and JAK3 in γc Cytokine Signaling,Role of JAK1, JAK2 and TYK2 in Interferon Signaling,Role of JAK2 in Hormone-like Cytokine Signaling,Role of Macrophages, Fibroblasts and Endothelial Cells in Rheumatoid Arthritis,Role of NANOG in Mammalian Embryonic Stem Cell Pluripotency,Role of Tissue Factor in Cancer,STAT3 Pathway,Tec Kinase Signaling,Thrombopoietin |

| | | | | | |
|---|---|---|---|---|---|
| | | | | | Signaling,TREM1 Signaling,Type I Diabetes Mellitus Signaling |
| MIMAT0005955 | miR-1197 (and other miRNAs w/seed AGGACAC) | TargetScan Human | Moderate (predicted) | JAK3 | CD40 Signaling,Colorectal Cancer Metastasis Signaling,ErbB2-ErbB3 Signaling,Glucocorticoid Receptor Signaling,IL-15 Production,IL-15 Signaling,IL-17A Signaling in Airway Cells,IL-2 Signaling,IL-4 Signaling,IL-9 Signaling,iNOS Signaling,JAK/Stat Signaling,Molecular Mechanisms of Cancer,Mouse Embryonic Stem Cell Pluripotency,Oncostatin M Signaling,Pancreatic Adenocarcinoma Signaling,PDGF Signaling,PI3K/AKT Signaling,Primary Immunodeficiency Signaling,Production of Nitric Oxide and Reactive Oxygen Species in Macrophages,Regulation of the Epithelial-Mesenchymal Transition Pathway,Role of JAK1 and JAK3 in γc Cytokine Signaling,Role of NANOG in Mammalian Embryonic Stem Cell Pluripotency,Tec Kinase Signaling |
| MIMAT0005955 | miR-1197 (and other miRNAs w/seed AGGACAC) | TargetScan Human | Moderate (predicted) | KCNJ1 | Dopamine-DARPP32 Feedback in cAMP Signaling |
| MIMAT0005955 | miR-1197 (and other miRNAs w/seed AGGACAC) | TargetScan Human | Moderate (predicted) | KCNJ8 | Dopamine-DARPP32 Feedback in cAMP Signaling |
| MIMAT0005955 | miR-1197 (and other miRNAs w/seed AGGACAC) | TargetScan Human | Moderate (predicted) | KDELR1 | Protein Kinase A Signaling |
| MIMAT0005955 | miR-1197 (and other miRNAs w/seed AGGACAC) | TargetScan Human | Moderate (predicted) | MAOA | Dopamine Degradation,Dopamine Receptor Signaling,LPS/IL-1 Mediated Inhibition of RXR Function,Melatonin Degradation II,Mitochondrial Dysfunction,Noradrenaline and Adrenaline Degradation,Phenylalanine Degradation IV (Mammalian, via Side Chain),Putrescine Degradation III,Serotonin Degradation,Serotonin Receptor Signaling,Superpathway of Melatonin Degradation,Tryptophan Degradation X (Mammalian, via Tryptamine),Xenobiotic Metabolism Signaling |
| MIMAT0005955 | miR-1197 (and other miRNAs w/seed AGGACAC) | TargetScan Human | Moderate (predicted) | MAP3K1 | Acute Phase Response Signaling,April Mediated Signaling,B Cell Activating Factor Signaling,B Cell Receptor Signaling,Cardiac Hypertrophy Signaling,CD27 Signaling in Lymphocytes,CD28 Signaling in T Helper Cells,Ceramide Signaling,EGF Signaling,FGF Signaling,Germ Cell-Sertoli Cell Junction |

| | | | | | |
|---|---|---|---|---|---|
| | | | | | Signaling,Glucocorticoid Receptor Signaling,GNRH Signaling,Gα12/13 Signaling,HGF Signaling,IL-1 Signaling,LPS/IL-1 Mediated Inhibition of RXR Function,NF-κB Activation by Viruses,NF-κB Signaling,NGF Signaling,NRF2-mediated Oxidative Stress Response,PDGF Signaling,PKCθ Signaling in T Lymphocytes,Production of Nitric Oxide and Reactive Oxygen Species in Macrophages,Protein Kinase A Signaling,Rac Signaling,RANK Signaling in Osteoclasts,RAR Activation,Regulation of IL-2 Expression in Activated and Anergic T Lymphocytes,Renin-Angiotensin Signaling,Role of NFAT in Cardiac Hypertrophy,SAPK/JNK Signaling,Sertoli Cell-Sertoli Cell Junction Signaling,T Cell Receptor Signaling,TNFR1 Signaling,TNFR2 Signaling,Toll-like Receptor Signaling,Type II Diabetes Mellitus Signaling,Xenobiotic Metabolism Signaling |
| MIMAT0005955 | miR-1197 (and other miRNAs w/seed AGGACAC) | TargetScan Human | Moderate (predicted) | MAP3K3 | B Cell Receptor Signaling,Cardiac Hypertrophy Signaling,CD27 Signaling in Lymphocytes,ERK5 Signaling,Germ Cell-Sertoli Cell Junction Signaling,GNRH Signaling,HGF Signaling,NF-κB Signaling,NGF Signaling,Nur77 Signaling in T Lymphocytes,PKCθ Signaling in T Lymphocytes,Production of Nitric Oxide and Reactive Oxygen Species in Macrophages,RANK Signaling in Osteoclasts,SAPK/JNK Signaling,Sertoli Cell-Sertoli Cell Junction Signaling,Xenobiotic Metabolism Signaling |
| MIMAT0005955 | miR-1197 (and other miRNAs w/seed AGGACAC) | TargetScan Human | Moderate (predicted) | MAP3K8 | B Cell Receptor Signaling,Cardiac Hypertrophy Signaling,CD27 Signaling in Lymphocytes,ERK5 Signaling,G-Protein Coupled Receptor Signaling,Germ Cell-Sertoli Cell Junction Signaling,GNRH Signaling,HGF Signaling,IL-12 Signaling and Production in Macrophages,NF-κB Signaling,NGF Signaling,PI3K/AKT Signaling,PKCθ Signaling in T Lymphocytes,Production of Nitric Oxide and Reactive Oxygen Species in Macrophages,Pyridoxal 5'-phosphate Salvage Pathway,RANK Signaling in Osteoclasts,Salvage Pathways of Pyrimidine Ribonucleotides,Sertoli Cell-Sertoli Cell Junction Signaling,Xenobiotic Metabolism Signaling |
| MIMAT0005955 | miR-1197 (and other miRNAs w/seed AGGACAC) | TargetScan Human | High (predicted) | MAP3K9 | B Cell Receptor Signaling,Cardiac Hypertrophy Signaling,CD27 Signaling in Lymphocytes,Germ Cell-Sertoli Cell Junction Signaling,GNRH Signaling,HGF Signaling,NGF Signaling,PKCθ Signaling in T Lymphocytes,Production of Nitric Oxide and Reactive Oxygen Species in Macrophages,Pyridoxal 5'-phosphate Salvage Pathway,RANK Signaling in Osteoclasts,Reelin Signaling in Neurons,Salvage Pathways of Pyrimidine Ribonucleotides,SAPK/JNK Signaling,Sertoli Cell-Sertoli Cell Junction Signaling,Signaling by Rho Family GTPases,STAT3 Pathway,Xenobiotic Metabolism Signaling |
| MIMAT0005955 | miR-1197 (and other miRNAs | TargetScan Human | Moderate (predicted) | MEF2D | Calcium Signaling,Calcium-induced T Lymphocyte Apoptosis,Cardiac Hypertrophy Signaling,Cholecystokinin/Gastrin-mediated |

| | | | | | |
|---|---|---|---|---|---|
| | w/seed AGGACAC) | | | | Signaling,Corticotropin Releasing Hormone Signaling,ERK5 Signaling,Gα12/13 Signaling,Nur77 Signaling in T Lymphocytes,p38 MAPK Signaling,Phospholipase C Signaling,Role of NFAT in Cardiac Hypertrophy,Role of NFAT in Regulation of the Immune Response |
| MIMAT0005955 | miR-1197 (and other miRNAs w/seed AGGACAC) | TargetScan Human | High (predicted) | MMP8 | Agranulocyte Adhesion and Diapedesis,Airway Pathology in Chronic Obstructive Pulmonary Disease,Axonal Guidance Signaling,Bladder Cancer Signaling,Colorectal Cancer Metastasis Signaling,Granulocyte Adhesion and Diapedesis,HIF1α Signaling,Inhibition of Matrix Metalloproteases,Leukocyte Extravasation Signaling,Role of Osteoblasts, Osteoclasts and Chondrocytes in Rheumatoid Arthritis |
| MIMAT0005955 | miR-1197 (and other miRNAs w/seed AGGACAC) | TargetScan Human | Moderate (predicted) | MYH14 | Actin Cytoskeleton Signaling,Agranulocyte Adhesion and Diapedesis,Calcium Signaling,Cellular Effects of Sildenafil (Viagra),Epithelial Adherens Junction Signaling,Hepatic Fibrosis / Hepatic Stellate Cell Activation,ILK Signaling,Tight Junction Signaling |
| MIMAT0005955 | miR-1197 (and other miRNAs w/seed AGGACAC) | TargetScan Human | Moderate (predicted) | NFATC4 | Adipogenesis pathway,April Mediated Signaling,Axonal Guidance Signaling,B Cell Activating Factor Signaling,B Cell Receptor Signaling,Calcium Signaling,Cardiac Hypertrophy Signaling,CD28 Signaling in T Helper Cells,fMLP Signaling in Neutrophils,Glucocorticoid Receptor Signaling,Gαq Signaling,iCOS-iCOSL Signaling in T Helper Cells,IL-4 Signaling,Netrin Signaling,Phospholipase C Signaling,PI3K Signaling in B Lymphocytes,PKCθ Signaling in T Lymphocytes,Protein Kinase A Signaling,Regulation of IL-2 Expression in Activated and Anergic T Lymphocytes,Role of Macrophages, Fibroblasts and Endothelial Cells in Rheumatoid Arthritis,Role of NFAT in Cardiac Hypertrophy,Role of NFAT in Regulation of the Immune Response,Role of Osteoblasts, Osteoclasts and Chondrocytes in Rheumatoid Arthritis,Systemic Lupus Erythematosus Signaling,T Cell Receptor Signaling,Wnt/Ca+ pathway |
| MIMAT0005955 | miR-1197 (and other miRNAs w/seed AGGACAC) | TargetScan Human | Moderate (predicted) | NOS1 | Amyotrophic Lateral Sclerosis Signaling,Antiproliferative Role of Somatostatin Receptor 2,Citrulline-Nitric Oxide Cycle,Corticotropin Releasing Hormone Signaling,Dopamine-DARPP32 Feedback in cAMP Signaling,Endothelin-1 Signaling,HIF1α Signaling,nNOS Signaling in Neurons,nNOS Signaling in Skeletal Muscle Cells,phagosome maturation,Sertoli Cell-Sertoli Cell Junction Signaling,Small Cell Lung Cancer Signaling,Superpathway of Citrulline Metabolism,Synaptic Long Term Depression |
| MIMAT0005955 | miR-1197 (and other miRNAs | TargetScan Human | Moderate (predicted) | NPR3 | cAMP-mediated signaling,G-Protein Coupled Receptor Signaling,Gαi Signaling |

| | | | | | |
|---|---|---|---|---|---|
| | w/seed AGGACAC) | | | | |
| MIMAT0005955 | miR-1197 (and other miRNAs w/seed AGGACAC) | TargetScan Human | Moderate (predicted) | NTRK2 | Axonal Guidance Signaling,CDK5 Signaling,Human Embryonic Stem Cell Pluripotency,Neuropathic Pain Signaling In Dorsal Horn Neurons,Neurotrophin/TRK Signaling,NF-κB Signaling,PTEN Signaling,STAT3 Pathway,Thyroid Cancer Signaling |
| MIMAT0005955 | miR-1197 (and other miRNAs w/seed AGGACAC) | TargetScan Human | Moderate (predicted) | OPRK1 | cAMP-mediated signaling,G-Protein Coupled Receptor Signaling,Gαi Signaling,Gαs Signaling |
| MIMAT0005955 | miR-1197 (and other miRNAs w/seed AGGACAC) | TargetScan Human | Moderate (predicted) | PAK6 | Actin Cytoskeleton Signaling,Agrin Interactions at Neuromuscular Junction,Angiopoietin Signaling,Axonal Guidance Signaling,CCR3 Signaling in Eosinophils,CXCR4 Signaling,Ephrin Receptor Signaling,ErbB Signaling,ERK/MAPK Signaling,FAK Signaling,Germ Cell-Sertoli Cell Junction Signaling,GNRH Signaling,Integrin Signaling,Molecular Mechanisms of Cancer,Natural Killer Cell Signaling,PAK Signaling,Paxillin Signaling,Rac Signaling,Regulation of Actin-based Motility by Rho,Renal Cell Carcinoma Signaling,Renin-Angiotensin Signaling,RhoGDI Signaling,Semaphorin Signaling in Neurons,Signaling by Rho Family GTPases,Tec Kinase Signaling,TNFR1 Signaling |
| MIMAT0005955 | miR-1197 (and other miRNAs w/seed AGGACAC) | TargetScan Human | Moderate (predicted) | PAX6 | Transcriptional Regulatory Network in Embryonic Stem Cells |
| MIMAT0005955 | miR-1197 (and other miRNAs w/seed AGGACAC) | TargetScan Human | Moderate (predicted) | PDE6G | cAMP-mediated signaling,Cardiac β-adrenergic Signaling,G-Protein Coupled Receptor Signaling,Gustation Pathway,Phototransduction Pathway,Protein Kinase A Signaling,Relaxin Signaling,tRNA Splicing |
| MIMAT0005955 | miR-1197 (and other miRNAs w/seed AGGACAC) | TargetScan Human | Moderate (predicted) | PDE7A | cAMP-mediated signaling,Cardiac β-adrenergic Signaling,G-Protein Coupled Receptor Signaling,Gustation Pathway,Protein Kinase A Signaling,Relaxin Signaling,tRNA Splicing |
| MIMAT0005955 | miR-1197 (and other miRNAs w/seed | TargetScan Human | Moderate (predicted) | PFKFB1 | AMPK Signaling |

| | | | | | |
|---|---|---|---|---|---|
| | AGGACAC) | | | | |
| MIMAT0005955 | miR-1197 (and other miRNAs w/seed AGGACAC) | TargetScan Human | Moderate (predicted) | PGP | Protein Kinase A Signaling |
| MIMAT0005955 | miR-1197 (and other miRNAs w/seed AGGACAC) | TargetScan Human | Moderate (predicted) | PIK3R5 | 14-3-3-mediated Signaling,3-phosphoinositide Biosynthesis,Actin Cytoskeleton Signaling,Acute Myeloid Leukemia Signaling,Aldosterone Signaling in Epithelial Cells,AMPK Signaling,Amyotrophic Lateral Sclerosis Signaling,Angiopoietin Signaling,Antiproliferative Role of Somatostatin Receptor 2,Axonal Guidance Signaling,B Cell Receptor Signaling,Breast Cancer Regulation by Stathmin1,Cardiac Hypertrophy Signaling,CCR3 Signaling in Eosinophils,CD28 Signaling in T Helper Cells,CD40 Signaling,Ceramide Signaling,Chronic Myeloid Leukemia Signaling,Clathrin-mediated Endocytosis Signaling,CNTF Signaling,Colorectal Cancer Metastasis Signaling,CREB Signaling in Neurons,CTLA4 Signaling in Cytotoxic T Lymphocytes,CXCR4 Signaling,Dendritic Cell Maturation,Docosahexaenoic Acid (DHA) Signaling,EGF Signaling,EIF2 Signaling,Endometrial Cancer Signaling,Endothelin-1 Signaling,eNOS Signaling,Ephrin A Signaling,ErbB Signaling,ErbB2-ErbB3 Signaling,ErbB4 Signaling,ERK/MAPK Signaling,Erythropoietin Signaling,Estrogen-Dependent Breast Cancer Signaling,FAK Signaling,Fc Epsilon RI Signaling,FcγRIIB Signaling in B Lymphocytes,FGF Signaling,FLT3 Signaling in Hematopoietic Progenitor Cells,fMLP Signaling in Neutrophils,G-Protein Coupled Receptor Signaling,Gap Junction Signaling,GDNF Family Ligand-Receptor Interactions,Germ Cell-Sertoli Cell Junction Signaling,Glioblastoma Multiforme Signaling,Glioma Invasiveness Signaling,Glioma Signaling,Glucocorticoid Receptor Signaling,GM-CSF Signaling,Growth Hormone Signaling,Gα12/13 Signaling,Gαq Signaling,HER-2 Signaling in Breast Cancer,Hereditary Breast Cancer Signaling,HGF Signaling,HIF1α Signaling,HMGB1 Signaling,Human Embryonic Stem Cell Pluripotency,Huntington's Disease Signaling,iCOS-iCOSL Signaling in T Helper Cells,IGF-1 Signaling,IL-12 Signaling and Production in Macrophages,IL-15 Signaling,IL-17 Signaling,IL-17A Signaling in Airway Cells,IL-2 Signaling,IL-3 Signaling,IL-4 Signaling,IL-6 Signaling,IL-8 Signaling,IL-9 Signaling,ILK Signaling,Insulin Receptor Signaling,Integrin Signaling,JAK/Stat Signaling,Leptin Signaling in Obesity,Leukocyte Extravasation Signaling,LPS-stimulated MAPK Signaling,Lymphotoxin β Receptor Signaling,Macropinocytosis Signaling,Melanocyte Development and Pigmentation Signaling,Melanoma Signaling,Molecular Mechanisms of Cancer,Mouse Embryonic Stem Cell Pluripotency,MSP-RON Signaling |

| | | | | | |
|---|---|---|---|---|---|
| | | | | | Pathway,mTOR Signaling,Myc Mediated Apoptosis Signaling,Natural Killer Cell Signaling,Neuropathic Pain Signaling In Dorsal Horn Neurons,Neurotrophin/TRK Signaling,NF-κB Activation by Viruses,NF-κB Signaling,NGF Signaling,Nitric Oxide Signaling in the Cardiovascular System,Non-Small Cell Lung Cancer Signaling,NRF2-mediated Oxidative Stress Response,Ovarian Cancer Signaling,P2Y Purigenic Receptor Signaling Pathway,p53 Signaling,p70S6K Signaling,PAK Signaling,Pancreatic Adenocarcinoma Signaling,Paxillin Signaling,PDGF Signaling,PEDF Signaling,phagosome formation,PKCθ Signaling in T Lymphocytes,Production of Nitric Oxide and Reactive Oxygen Species in Macrophages,Prolactin Signaling,Prostate Cancer Signaling,PTEN Signaling,Rac Signaling,RANK Signaling in Osteoclasts,Reelin Signaling in Neurons,Regulation of eIF4 and p70S6K Signaling,Regulation of the Epithelial-Mesenchymal Transition Pathway,Relaxin Signaling,Renal Cell Carcinoma Signaling,Renin-Angiotensin Signaling,Role of IL-17A in Arthritis,Role of JAK1 and JAK3 in γc Cytokine Signaling,Role of Macrophages, Fibroblasts and Endothelial Cells in Rheumatoid Arthritis,Role of NANOG in Mammalian Embryonic Stem Cell Pluripotency,Role of NFAT in Cardiac Hypertrophy,Role of NFAT in Regulation of the Immune Response,Role of Osteoblasts, Osteoclasts and Chondrocytes in Rheumatoid Arthritis,Role of p14/p19ARF in Tumor Suppression,Role of Pattern Recognition Receptors in Recognition of Bacteria and Viruses,Role of PI3K/AKT Signaling in the Pathogenesis of Influenza,Role of Tissue Factor in Cancer,SAPK/JNK Signaling,Signaling by Rho Family GTPases,Small Cell Lung Cancer Signaling,Sphingosine-1-phosphate Signaling,Superpathway of Inositol Phosphate Compounds,Systemic Lupus Erythematosus Signaling,T Cell Receptor Signaling,Tec Kinase Signaling,Telomerase Signaling,Thrombin Signaling,Thrombopoietin Signaling,TR/RXR Activation,Type II Diabetes Mellitus Signaling,UVA-Induced MAPK Signaling,UVB-Induced MAPK Signaling,VEGF Family Ligand-Receptor Interactions,VEGF Signaling,Virus Entry via Endocytic Pathways,Xenobiotic Metabolism Signaling |
| MIMAT0005955 | miR-1197 (and other miRNAs w/seed AGGACAC) | TargetScan Human | Moderate (predicted) | PLA2G2D | Antioxidant Action of Vitamin C,Atherosclerosis Signaling,CCR3 Signaling in Eosinophils,Eicosanoid Signaling,Endothelin-1 Signaling,ERK/MAPK Signaling,Fc Epsilon RI Signaling,MIF Regulation of Innate Immunity,MIF-mediated Glucocorticoid Regulation,p38 MAPK Signaling,Phospholipase C Signaling,Phospholipases,Role of MAPK Signaling in the Pathogenesis of Influenza,Sperm Motility,Synaptic Long Term Depression,VEGF Family Ligand-Receptor Interactions |
| MIMAT0005955 | miR-1197 (and other miRNAs | TargetScan Human | Moderate (predicted) | PPARGC1A | AMPK Signaling,Estrogen Receptor Signaling,FXR/RXR Activation,LPS/IL-1 Mediated Inhibition of RXR Function,PPAR Signaling,PPARα/RXRα |

| | | | | | |
|---|---|---|---|---|---|
| | w/seed AGGACAC) | | | | Activation,PXR/RXR Activation,RAR Activation,TR/RXR Activation,Xenobiotic Metabolism Signaling |
| MIMAT0005955 | miR-1197 (and other miRNAs w/seed AGGACAC) | TargetScan Human | Moderate (predicted) | PPM1A | AMPK Signaling |
| MIMAT0005955 | miR-1197 (and other miRNAs w/seed AGGACAC) | TargetScan Human | Moderate (predicted) | PPP1R14C | Breast Cancer Regulation by Stathmin1,Cardiac β-adrenergic Signaling,CDK5 Signaling,Dopamine Receptor Signaling,Dopamine-DARPP32 Feedback in cAMP Signaling,ERK/MAPK Signaling,HIPPO signaling,Insulin Receptor Signaling,Production of Nitric Oxide and Reactive Oxygen Species in Macrophages,Protein Kinase A Signaling,Synaptic Long Term Potentiation |
| MIMAT0005955 | miR-1197 (and other miRNAs w/seed AGGACAC) | TargetScan Human | Moderate (predicted) | PPP1R3D | Breast Cancer Regulation by Stathmin1,Cardiac β-adrenergic Signaling,CDK5 Signaling,Dopamine Receptor Signaling,Dopamine-DARPP32 Feedback in cAMP Signaling,ERK/MAPK Signaling,HIPPO signaling,Insulin Receptor Signaling,Production of Nitric Oxide and Reactive Oxygen Species in Macrophages,Protein Kinase A Signaling,Synaptic Long Term Potentiation |
| MIMAT0005955 | miR-1197 (and other miRNAs w/seed AGGACAC) | TargetScan Human | Moderate (predicted) | PPP2R5C | AMPK Signaling,Breast Cancer Regulation by Stathmin1,Cardiac β-adrenergic Signaling,CDK5 Signaling,Cell Cycle Regulation by BTG Family Proteins,Ceramide Signaling,CTLA4 Signaling in Cytotoxic T Lymphocytes,Cyclins and Cell Cycle Regulation,Dopamine Receptor Signaling,Dopamine-DARPP32 Feedback in cAMP Signaling,ERK/MAPK Signaling,HIPPO signaling,ILK Signaling,Mitotic Roles of Polo-Like Kinase,mTOR Signaling,p70S6K Signaling,PI3K/AKT Signaling,Production of Nitric Oxide and Reactive Oxygen Species in Macrophages,Regulation of eIF4 and p70S6K Signaling,Role of CHK Proteins in Cell Cycle Checkpoint Control,Synaptic Long Term Depression,Telomerase Signaling,Tight Junction Signaling,Wnt/β-catenin Signaling,Xenobiotic Metabolism Signaling |
| MIMAT0005955 | miR-1197 (and other miRNAs w/seed AGGACAC) | TargetScan Human | Moderate (predicted) | PRKAG2 | AMPK Signaling,Amyloid Processing,Androgen Signaling,Axonal Guidance Signaling,BMP signaling pathway,Breast Cancer Regulation by Stathmin1,Calcium Signaling,Cardiac Hypertrophy Signaling,Cardiac β-adrenergic Signaling,CDK5 Signaling,Cellular Effects of Sildenafil (Viagra),Colorectal Cancer Metastasis Signaling,Corticotropin Releasing Hormone Signaling,CREB Signaling in Neurons,Dopamine Receptor Signaling,Dopamine-DARPP32 Feedback in cAMP Signaling,eNOS Signaling,ERK/MAPK Signaling,G Beta Gamma Signaling,G-Protein Coupled Receptor Signaling,Gap Junction Signaling,Glucocorticoid Receptor |

| | | | | | |
|---|---|---|---|---|---|
| | | | | | Signaling,GNRH Signaling,GPCR-Mediated Integration of Enteroendocrine Signaling Exemplified by an L Cell,GPCR-Mediated Nutrient Sensing in Enteroendocrine Cells,Gustation Pathway,Gαi Signaling,Gαs Signaling,Hepatic Cholestasis,IGF-1 Signaling,IL-1 Signaling,Insulin Receptor Signaling,Leptin Signaling in Obesity,Melanocyte Development and Pigmentation Signaling,Melatonin Signaling,Molecular Mechanisms of Cancer,mTOR Signaling,Netrin Signaling,Neuropathic Pain Signaling In Dorsal Horn Neurons,Neuroprotective Role of THOP1 in Alzheimer's Disease,Nitric Oxide Signaling in the Cardiovascular System,Ovarian Cancer Signaling,P2Y Purigenic Receptor Signaling Pathway,Phototransduction Pathway,PPARα/RXRα Activation,Protein Kinase A Signaling,PXR/RXR Activation,RAR Activation,Relaxin Signaling,Renin-Angiotensin Signaling,Role of NFAT in Cardiac Hypertrophy,Sertoli Cell-Sertoli Cell Junction Signaling,Sonic Hedgehog Signaling,Sperm Motility,Synaptic Long Term Potentiation,Tight Junction Signaling,Type II Diabetes Mellitus Signaling,α-Adrenergic Signaling |
| MIMAT0005955 | miR-1197 (and other miRNAs w/seed AGGACAC) | TargetScan Human | Moderate (predicted) | PTPRK | Protein Kinase A Signaling |
| MIMAT0005955 | miR-1197 (and other miRNAs w/seed AGGACAC) | TargetScan Human | Moderate (predicted) | PTPRO | 3-phosphoinositide Biosynthesis,3-phosphoinositide Degradation,D-myo-inositol (1,4,5,6)-Tetrakisphosphate Biosynthesis,D-myo-inositol (3,4,5,6)-tetrakisphosphate Biosynthesis,D-myo-inositol-5-phosphate Metabolism,Protein Kinase A Signaling,Superpathway of Inositol Phosphate Compounds |
| MIMAT0005955 | miR-1197 (and other miRNAs w/seed AGGACAC) | TargetScan Human | Moderate (predicted) | RAPGEF3 | cAMP-mediated signaling,ERK/MAPK Signaling,G-Protein Coupled Receptor Signaling,Gαs Signaling,Leukocyte Extravasation Signaling,Molecular Mechanisms of Cancer,Phospholipase C Signaling,Synaptic Long Term Potentiation |
| MIMAT0005955 | miR-1197 (and other miRNAs w/seed AGGACAC) | TargetScan Human | High (predicted) | RB1 | Antiproliferative Role of TOB in T Cell Signaling,Aryl Hydrocarbon Receptor Signaling,Bladder Cancer Signaling,Cell Cycle Regulation by BTG Family Proteins,Cell Cycle: G1/S Checkpoint Regulation,Chronic Myeloid Leukemia Signaling,Cyclins and Cell Cycle Regulation,Estrogen-mediated S-phase Entry,Glioblastoma Multiforme Signaling,Glioma Signaling,Hereditary Breast Cancer Signaling,Melanoma Signaling,Molecular Mechanisms of Cancer,Non-Small Cell Lung Cancer Signaling,Ovarian Cancer Signaling,p53 Signaling,Pancreatic Adenocarcinoma Signaling,Prostate Cancer Signaling,Regulation of Cellular Mechanics by Calpain Protease,Role of BRCA1 |

| | | | | | |
|---|---|---|---|---|---|
| | | | | | in DNA Damage Response,Role of Oct4 in Mammalian Embryonic Stem Cell Pluripotency,Role of p14/p19ARF in Tumor Suppression,Small Cell Lung Cancer Signaling,Telomerase Signaling |
| MIMAT0005955 | miR-1197 (and other miRNAs w/seed AGGACAC) | TargetScan Human | Moderate (predicted) | RCAN2 | Calcium Signaling,Role of NFAT in Cardiac Hypertrophy,Role of NFAT in Regulation of the Immune Response,TR/RXR Activation |
| MIMAT0005955 | miR-1197 (and other miRNAs w/seed AGGACAC) | TargetScan Human | Moderate (predicted) | RORC | Melatonin Signaling,T Helper Cell Differentiation |
| MIMAT0005955 | miR-1197 (and other miRNAs w/seed AGGACAC) | TargetScan Human | Moderate (predicted) | S1PR1 | cAMP-mediated signaling,Ceramide Signaling,G-Protein Coupled Receptor Signaling,Gαi Signaling,Human Embryonic Stem Cell Pluripotency,Sphingosine-1-phosphate Signaling |
| MIMAT0005955 | miR-1197 (and other miRNAs w/seed AGGACAC) | TargetScan Human | Moderate (predicted) | SCNN1B | Aldosterone Signaling in Epithelial Cells,Gustation Pathway,Insulin Receptor Signaling |
| MIMAT0005955 | miR-1197 (and other miRNAs w/seed AGGACAC) | TargetScan Human | Moderate (predicted) | SCNN1G | Aldosterone Signaling in Epithelial Cells,Gustation Pathway,Insulin Receptor Signaling |
| MIMAT0005955 | miR-1197 (and other miRNAs w/seed AGGACAC) | TargetScan Human | Moderate (predicted) | SLC2A1 | AMPK Signaling,Antioxidant Action of Vitamin C,HIF1α Signaling,Renal Cell Carcinoma Signaling,TR/RXR Activation,Vitamin-C Transport |
| MIMAT0005955 | miR-1197 (and other miRNAs w/seed AGGACAC) | TargetScan Human | High (predicted) | SLC6A3 | Dopamine Receptor Signaling |
| MIMAT0005955 | miR-1197 (and other miRNAs w/seed | TargetScan Human | Moderate (predicted) | SMAD3 | Adipogenesis pathway,Androgen Signaling,Antiproliferative Role of TOB in T Cell Signaling,Cell Cycle: G1/S Checkpoint Regulation,Chronic Myeloid Leukemia Signaling,Colorectal Cancer Metastasis Signaling,Glucocorticoid |

| | | | | | |
|---|---|---|---|---|---|
| | AGGACAC) | | | | Receptor Signaling,Hepatic Fibrosis / Hepatic Stellate Cell Activation,HIPPO signaling,Human Embryonic Stem Cell Pluripotency,Molecular Mechanisms of Cancer,Pancreatic Adenocarcinoma Signaling,PPARα/RXRα Activation,Protein Kinase A Signaling,RAR Activation,Regulation of IL-2 Expression in Activated and Anergic T Lymphocytes,Regulation of the Epithelial-Mesenchymal Transition Pathway,TGF-β Signaling |
| MIMAT0005955 | miR-1197 (and other miRNAs w/seed AGGACAC) | TargetScan Human | Moderate (predicted) | SOCS5 | Acute Phase Response Signaling,Growth Hormone Signaling,IGF-1 Signaling,JAK/Stat Signaling,Prolactin Signaling,Role of JAK2 in Hormone-like Cytokine Signaling,STAT3 Pathway,Type I Diabetes Mellitus Signaling,Type II Diabetes Mellitus Signaling |
| MIMAT0005955 | miR-1197 (and other miRNAs w/seed AGGACAC) | TargetScan Human | Moderate (predicted) | SPR | Dopamine Receptor Signaling,Serotonin Receptor Signaling,Tetrahydrobiopterin Biosynthesis I,Tetrahydrobiopterin Biosynthesis II |
| MIMAT0005955 | miR-1197 (and other miRNAs w/seed AGGACAC) | TargetScan Human | Moderate (predicted) | SRGAP1 | Axonal Guidance Signaling |
| MIMAT0005955 | miR-1197 (and other miRNAs w/seed AGGACAC) | TargetScan Human | Moderate (predicted) | TPH2 | Serotonin and Melatonin Biosynthesis,Serotonin Receptor Signaling |
| MIMAT0005955 | miR-1197 (and other miRNAs w/seed AGGACAC) | TargetScan Human | Moderate (predicted) | TPM1 | Calcium Signaling |
| MIMAT0005955 | miR-1197 (and other miRNAs w/seed AGGACAC) | TargetScan Human | High (predicted) | TSC22D3 | Glucocorticoid Receptor Signaling |
| MIMAT0005955 | miR-1197 (and other miRNAs w/seed AGGACAC) | TargetScan Human | Moderate (predicted) | ZFHX3 | Transcriptional Regulatory Network in Embryonic Stem Cells |

| | | | | | |
|---|---|---|---|---|---|
| MIMAT0000070 | miR-17-5p (and other miRNAs w/seed AAAGUGC) | TargetScan Human | High (predicted) | ADAM9 | Axonal Guidance Signaling |
| MIMAT0000070 | miR-17-5p (and other miRNAs w/seed AAAGUGC) | TargetScan Human | High (predicted) | ADAMTS5 | Axonal Guidance Signaling,Role of Osteoblasts, Osteoclasts and Chondrocytes in Rheumatoid Arthritis |
| MIMAT0000070 | miR-17-5p (and other miRNAs w/seed AAAGUGC) | TargetScan Human | High (predicted) | ADRA1B | AMPK Signaling,Cardiac Hypertrophy Signaling,G-Protein Coupled Receptor Signaling,Gαq Signaling |
| MIMAT0000070 | miR-17-5p (and other miRNAs w/seed AAAGUGC) | TargetScan Human | High (predicted) | AHNAK | Phospholipase C Signaling |
| MIMAT0000070 | miR-17-5p (and other miRNAs w/seed AAAGUGC) | TargetScan Human | High (predicted) | AK4 | AMPK Signaling,Pyrimidine Deoxyribonucleotides De Novo Biosynthesis I,Pyrimidine Ribonucleotides De Novo Biosynthesis,Pyrimidine Ribonucleotides Interconversion,Salvage Pathways of Pyrimidine Ribonucleotides |
| MIMAT0000070 | miR-17-5p (and other miRNAs w/seed AAAGUGC) | TargetScan Human | High (predicted) | AKAP11 | cAMP-mediated signaling,Cardiac β-adrenergic Signaling,Protein Kinase A Signaling |
| MIMAT0000070 | miR-17-5p (and other miRNAs w/seed AAAGUGC) | TargetScan Human | High (predicted) | AKAP13 | cAMP-mediated signaling,Cardiac β-adrenergic Signaling,Protein Kinase A Signaling |
| MIMAT0000070 | miR-17-5p (and other miRNAs w/seed AAAGUGC) | TargetScan Human | High (predicted) | AKT3 | 14-3-3-mediated Signaling,Acute Myeloid Leukemia Signaling,Acute Phase Response Signaling,AMPK Signaling,Amyloid Processing,Amyotrophic Lateral Sclerosis Signaling,Angiopoietin Signaling,Axonal Guidance Signaling,B Cell Receptor Signaling,CD28 Signaling in T Helper Cells,Ceramide Signaling,Chronic Myeloid Leukemia Signaling,Colorectal Cancer Metastasis Signaling,CREB Signaling in Neurons,CTLA4 Signaling in Cytotoxic T Lymphocytes,CXCR4 Signaling,Dendritic Cell Maturation,DNA damage- |

| | | | | | |
|---|---|---|---|---|---|
| | | | | | induced 14-3-3σ Signaling,Docosahexaenoic Acid (DHA) Signaling,EGF Signaling,EIF2 Signaling,Endometrial Cancer Signaling,eNOS Signaling,Ephrin Receptor Signaling,Epithelial Adherens Junction Signaling,Erythropoietin Signaling,Estrogen-Dependent Breast Cancer Signaling,FAK Signaling,Fc Epsilon RI Signaling,Fcγ Receptor-mediated Phagocytosis in Macrophages and Monocytes,FGF Signaling,FLT3 Signaling in Hematopoietic Progenitor Cells,FXR/RXR Activation,G Beta Gamma Signaling,G-Protein Coupled Receptor Signaling,Gap Junction Signaling,Glioblastoma Multiforme Signaling,Glioma Signaling,Glucocorticoid Receptor Signaling,GM-CSF Signaling,Gα12/13 Signaling,Gαq Signaling,HER-2 Signaling in Breast Cancer,Hereditary Breast Cancer Signaling,HGF Signaling,HIF1α Signaling,HMGB1 Signaling,Human Embryonic Stem Cell Pluripotency,Huntington's Disease Signaling,iCOS-iCOSL Signaling in T Helper Cells,IGF-1 Signaling,IL-12 Signaling and Production in Macrophages,IL-15 Signaling,IL-17 Signaling,IL-17A Signaling in Airway Cells,IL-2 Signaling,IL-22 Signaling,IL-3 Signaling,IL-4 Signaling,IL-6 Signaling,IL-8 Signaling,ILK Signaling,Inhibition of Angiogenesis by TSP1,Insulin Receptor Signaling,Integrin Signaling,JAK/Stat Signaling,Leptin Signaling in Obesity,Lymphotoxin β Receptor Signaling,Melanoma Signaling,Molecular Mechanisms of Cancer,Mouse Embryonic Stem Cell Pluripotency,mTOR Signaling,Myc Mediated Apoptosis Signaling,Natural Killer Cell Signaling,Neuregulin Signaling,NF-κB Activation by Viruses,NF-κB Signaling,NGF Signaling,Nitric Oxide Signaling in the Cardiovascular System,Non-Small Cell Lung Cancer Signaling,Ovarian Cancer Signaling,P2Y Purigenic Receptor Signaling Pathway,p53 Signaling,p70S6K Signaling,Pancreatic Adenocarcinoma Signaling,PEDF Signaling,PI3K Signaling in B Lymphocytes,PI3K/AKT Signaling,Production of Nitric Oxide and Reactive Oxygen Species in Macrophages,Prostate Cancer Signaling,PTEN Signaling,PXR/RXR Activation,RANK Signaling in Osteoclasts,RAR Activation,Regulation of eIF4 and p70S6K Signaling,Regulation of the Epithelial-Mesenchymal Transition Pathway,Relaxin Signaling,Renal Cell Carcinoma Signaling,Role of Macrophages, Fibroblasts and Endothelial Cells in Rheumatoid Arthritis,Role of MAPK Signaling in the Pathogenesis of Influenza,Role of NANOG in Mammalian Embryonic Stem Cell Pluripotency,Role of NFAT in Cardiac Hypertrophy,Role of NFAT in Regulation of the Immune Response,Role of Osteoblasts, Osteoclasts and Chondrocytes in Rheumatoid Arthritis,Role of PI3K/AKT Signaling in the Pathogenesis of Influenza,Role of Tissue Factor in Cancer,Sertoli Cell-Sertoli Cell Junction Signaling,Small Cell Lung Cancer Signaling,Sphingosine-1-phosphate Signaling,Systemic Lupus Erythematosus Signaling,Telomerase Signaling,Thrombin Signaling,Tight Junction Signaling,TR/RXR |

| | | | | | |
|---|---|---|---|---|---|
| | | | | | Activation,TREM1 Signaling,Type II Diabetes Mellitus Signaling,VEGF Family Ligand-Receptor Interactions,VEGF Signaling,Wnt/β-catenin Signaling |
| MIMAT0000070 | miR-17-5p (and other miRNAs w/seed AAAGUGC) | TargetScan Human | High (predicted) | AP2B1 | Clathrin-mediated Endocytosis Signaling,CTLA4 Signaling in Cytotoxic T Lymphocytes,GABA Receptor Signaling,Lipid Antigen Presentation by CD1,Virus Entry via Endocytic Pathways |
| MIMAT0000070 | miR-17-5p (and other miRNAs w/seed AAAGUGC) | Ingenuity Expert Findings,TargetScan Human,miRecords | Experimentally Observed,High (predicted) | APP | Amyloid Processing,Docosahexaenoic Acid (DHA) Signaling,Mitochondrial Dysfunction,Neuroprotective Role of THOP1 in Alzheimer's Disease,Reelin Signaling in Neurons |
| MIMAT0000070 | miR-17-5p (and other miRNAs w/seed AAAGUGC) | TargetScan Human | High (predicted) | ARHGAP1 | Leukocyte Extravasation Signaling,RhoA Signaling,RhoGDI Signaling,Semaphorin Signaling in Neurons |
| MIMAT0000070 | miR-17-5p (and other miRNAs w/seed AAAGUGC) | TargetScan Human | High (predicted) | ARHGEF10 | Breast Cancer Regulation by Stathmin1,Molecular Mechanisms of Cancer,Phospholipase C Signaling,Reelin Signaling in Neurons,RhoGDI Signaling,Signaling by Rho Family GTPases,Thrombin Signaling |
| MIMAT0000070 | miR-17-5p (and other miRNAs w/seed AAAGUGC) | TargetScan Human | High (predicted) | ARHGEF11 | Axonal Guidance Signaling,Breast Cancer Regulation by Stathmin1,CXCR4 Signaling,Molecular Mechanisms of Cancer,Phospholipase C Signaling,Reelin Signaling in Neurons,RhoA Signaling,RhoGDI Signaling,Signaling by Rho Family GTPases,Thrombin Signaling |
| MIMAT0000070 | miR-17-5p (and other miRNAs w/seed AAAGUGC) | TargetScan Human | High (predicted) | ARHGEF18 | Breast Cancer Regulation by Stathmin1,Molecular Mechanisms of Cancer,Phospholipase C Signaling,RhoGDI Signaling,Signaling by Rho Family GTPases |
| MIMAT0000070 | miR-17-5p (and other miRNAs w/seed AAAGUGC) | TargetScan Human | High (predicted) | ARHGEF3 | Breast Cancer Regulation by Stathmin1,Molecular Mechanisms of Cancer,Phospholipase C Signaling,Reelin Signaling in Neurons,RhoGDI Signaling,Signaling by Rho Family GTPases,Thrombin Signaling |
| MIMAT0000070 | miR-17-5p (and other miRNAs w/seed | TargetScan Human | High (predicted) | ARHGEF7 | Actin Cytoskeleton Signaling,Agrin Interactions at Neuromuscular Junction,Axonal Guidance Signaling,Breast Cancer Regulation by Stathmin1,FAK Signaling,Integrin Signaling,Molecular Mechanisms of Cancer,PAK Signaling,Paxillin Signaling,Phospholipase C Signaling,RhoGDI |

| | | | | | |
|---|---|---|---|---|---|
| | AAAGUGC) | | | | Signaling,Signaling by Rho Family GTPases |
| MIMAT0000070 | miR-17-5p (and other miRNAs w/seed AAAGUGC) | TargetScan Human | High (predicted) | ATP2B2 | Calcium Signaling,Calcium Transport I |
| MIMAT0000070 | miR-17-5p (and other miRNAs w/seed AAAGUGC) | miRecords | Experimentally Observed | BCL2 | Amyotrophic Lateral Sclerosis Signaling,Apoptosis Signaling,autophagy,Ceramide Signaling,Cytotoxic T Lymphocyte-mediated Apoptosis of Target Cells,Death Receptor Signaling,Docosahexaenoic Acid (DHA) Signaling,Glucocorticoid Receptor Signaling,Hepatic Fibrosis / Hepatic Stellate Cell Activation,IL-15 Signaling,IL-8 Signaling,Induction of Apoptosis by HIV1,Interferon Signaling,Melanocyte Development and Pigmentation Signaling,Mitochondrial Dysfunction,Molecular Mechanisms of Cancer,Myc Mediated Apoptosis Signaling,Nur77 Signaling in T Lymphocytes,Ovarian Cancer Signaling,OX40 Signaling Pathway,p53 Signaling,Pancreatic Adenocarcinoma Signaling,PEDF Signaling,PI3K/AKT Signaling,Prostate Cancer Signaling,PTEN Signaling,Role of MAPK Signaling in the Pathogenesis of Influenza,Role of Osteoblasts, Osteoclasts and Chondrocytes in Rheumatoid Arthritis,Small Cell Lung Cancer Signaling,STAT3 Pathway,TGF-β Signaling,Type I Diabetes Mellitus Signaling,Unfolded protein response,VEGF Signaling |
| MIMAT0000070 | miR-17-5p (and other miRNAs w/seed AAAGUGC) | TargetScan Human | High (predicted) | BHLHE41 | Circadian Rhythm Signaling |
| MIMAT0000070 | miR-17-5p (and other miRNAs w/seed AAAGUGC) | TargetScan Human | High (predicted) | BMP2 | Adipogenesis pathway,Axonal Guidance Signaling,Basal Cell Carcinoma Signaling,BMP signaling pathway,Cardiomyocyte Differentiation via BMP Receptors,Factors Promoting Cardiogenesis in Vertebrates,Human Embryonic Stem Cell Pluripotency,ILK Signaling,Molecular Mechanisms of Cancer,NF-κB Signaling,RAR Activation,Retinoate Biosynthesis I,Role of NANOG in Mammalian Embryonic Stem Cell Pluripotency,Role of Osteoblasts, Osteoclasts and Chondrocytes in Rheumatoid Arthritis,TGF-β Signaling |
| MIMAT0000070 | miR-17-5p (and other miRNAs w/seed AAAGUGC) | TargetScan Human,miRecords | Experimentally Observed,High (predicted) | BMPR2 | Adipogenesis pathway,BMP signaling pathway,Cardiomyocyte Differentiation via BMP Receptors,Epithelial Adherens Junction Signaling,Factors Promoting Cardiogenesis in Vertebrates,Human Embryonic Stem Cell Pluripotency,Molecular Mechanisms of Cancer,Mouse Embryonic Stem Cell Pluripotency,NF-κB Signaling,PPARα/RXRα Activation,PTEN Signaling,Role of |

| | | | | | |
|---|---|---|---|---|---|
| | | | | | NANOG in Mammalian Embryonic Stem Cell Pluripotency,Role of Osteoblasts, Osteoclasts and Chondrocytes in Rheumatoid Arthritis,STAT3 Pathway,TGF-β Signaling,Wnt/β-catenin Signaling |
| MIMAT0000070 | miR-17-5p (and other miRNAs w/seed AAAGUGC) | TargetScan Human | High (predicted) | CABLES1 | CDK5 Signaling |
| MIMAT0000070 | miR-17-5p (and other miRNAs w/seed AAAGUGC) | TargetScan Human | High (predicted) | CASP7 | Amyotrophic Lateral Sclerosis Signaling,Apoptosis Signaling,Cytotoxic T Lymphocyte-mediated Apoptosis of Target Cells,Death Receptor Signaling,Endoplasmic Reticulum Stress Pathway,Endothelin-1 Signaling,Huntington's Disease Signaling,Molecular Mechanisms of Cancer,PEDF Signaling,Sphingosine-1-phosphate Signaling,TNFR1 Signaling,Tumoricidal Function of Hepatic Natural Killer Cells,TWEAK Signaling |
| MIMAT0000070 | miR-17-5p (and other miRNAs w/seed AAAGUGC) | TargetScan Human,miRecords | Experimentally Observed,High (predicted) | CCND1 | Acute Myeloid Leukemia Signaling,AMPK Signaling,Androgen Signaling,Aryl Hydrocarbon Receptor Signaling,Bladder Cancer Signaling,Cell Cycle Regulation by BTG Family Proteins,Cell Cycle: G1/S Checkpoint Regulation,Chronic Myeloid Leukemia Signaling,Colorectal Cancer Metastasis Signaling,Cyclins and Cell Cycle Regulation,Endometrial Cancer Signaling,ErbB2-ErbB3 Signaling,Estrogen-Dependent Breast Cancer Signaling,Estrogen-mediated S-phase Entry,GADD45 Signaling,Glioblastoma Multiforme Signaling,Glioma Signaling,GM-CSF Signaling,HER-2 Signaling in Breast Cancer,Hereditary Breast Cancer Signaling,HGF Signaling,IL-8 Signaling,ILK Signaling,Melanoma Signaling,Molecular Mechanisms of Cancer,Non-Small Cell Lung Cancer Signaling,Ovarian Cancer Signaling,p53 Signaling,Pancreatic Adenocarcinoma Signaling,PI3K/AKT Signaling,Prostate Cancer Signaling,PTEN Signaling,Regulation of Cellular Mechanics by Calpain Protease,Role of Macrophages, Fibroblasts and Endothelial Cells in Rheumatoid Arthritis,Small Cell Lung Cancer Signaling,Thyroid Cancer Signaling,Wnt/β-catenin Signaling |
| MIMAT0000070 | miR-17-5p (and other miRNAs w/seed AAAGUGC) | TargetScan Human | High (predicted) | CDC23 | Mitotic Roles of Polo-Like Kinase,Protein Kinase A Signaling,Protein Ubiquitination Pathway |
| MIMAT0000070 | miR-17-5p (and other miRNAs w/seed | TargetScan Human | High (predicted) | CDC25A | 3-phosphoinositide Biosynthesis,3-phosphoinositide Degradation,ATM Signaling,Cell Cycle: G1/S Checkpoint Regulation,Cyclins and Cell Cycle Regulation,D-myo-inositol (1,4,5,6)-Tetrakisphosphate Biosynthesis,D-myo- |

| | | | | | |
|---|---|---|---|---|---|
| | AAAGUGC) | | | | inositol (3,4,5,6)-tetrakisphosphate Biosynthesis,D-myo-inositol-5-phosphate Metabolism,Estrogen-mediated S-phase Entry,Mitotic Roles of Polo-Like Kinase,Molecular Mechanisms of Cancer,Protein Kinase A Signaling,Role of CHK Proteins in Cell Cycle Checkpoint Control,STAT3 Pathway,Superpathway of Inositol Phosphate Compounds |
| MIMAT0000070 | miR-17-5p (and other miRNAs w/seed AAAGUGC) | Ingenuity Expert Findings,TarBase,TargetScan Human,miRecords | Experimentally Observed,High (predicted) | CDKN1A | AMPK Signaling,Antiproliferative Role of Somatostatin Receptor 2,Aryl Hydrocarbon Receptor Signaling,ATM Signaling,Bladder Cancer Signaling,Breast Cancer Regulation by Stathmin1,Cell Cycle: G1/S Checkpoint Regulation,Cell Cycle: G2/M DNA Damage Checkpoint Regulation,Chronic Myeloid Leukemia Signaling,Cyclins and Cell Cycle Regulation,Estrogen-mediated S-phase Entry,GADD45 Signaling,Glioblastoma Multiforme Signaling,Glioma Signaling,Glucocorticoid Receptor Signaling,HER-2 Signaling in Breast Cancer,Hereditary Breast Cancer Signaling,HGF Signaling,JAK/Stat Signaling,Melanoma Signaling,Molecular Mechanisms of Cancer,p53 Signaling,Pancreatic Adenocarcinoma Signaling,PI3K/AKT Signaling,Prostate Cancer Signaling,PTEN Signaling,Role of BRCA1 in DNA Damage Response,Role of CHK Proteins in Cell Cycle Checkpoint Control,STAT3 Pathway,Telomerase Signaling,VDR/RXR Activation |
| MIMAT0000070 | miR-17-5p (and other miRNAs w/seed AAAGUGC) | TargetScan Human | High (predicted) | CFL2 | Actin Cytoskeleton Signaling,Axonal Guidance Signaling,B Cell Receptor Signaling,CCR3 Signaling in Eosinophils,Cdc42 Signaling,Ephrin A Signaling,Ephrin B Signaling,Ephrin Receptor Signaling,Germ Cell-Sertoli Cell Junction Signaling,ILK Signaling,PAK Signaling,Rac Signaling,RhoA Signaling,RhoGDI Signaling,Role of Tissue Factor in Cancer,Semaphorin Signaling in Neurons,Signaling by Rho Family GTPases |
| MIMAT0000070 | miR-17-5p (and other miRNAs w/seed AAAGUGC) | TargetScan Human | High (predicted) | CHRM2 | cAMP-mediated signaling,G-Protein Coupled Receptor Signaling,GPCR-Mediated Integration of Enteroendocrine Signaling Exemplified by an L Cell,Gαi Signaling |
| MIMAT0000070 | miR-17-5p (and other miRNAs w/seed AAAGUGC) | TargetScan Human | High (predicted) | CLOCK | Adipogenesis pathway,Circadian Rhythm Signaling,PPARα/RXRα Activation |
| MIMAT0000070 | miR-17-5p (and other miRNAs w/seed AAAGUGC) | TargetScan Human | High (predicted) | CNGB3 | cAMP-mediated signaling,eNOS Signaling,Gαs Signaling,Phototransduction Pathway,Protein Kinase A Signaling,Regulation of Cellular Mechanics by Calpain Protease,Sperm Motility |

| | | | | | |
|---|---|---|---|---|---|
| MIMAT0000070 | miR-17-5p (and other miRNAs w/seed AAAGUGC) | Ingenuity Expert Findings,TargetScan Human | Experimentally Observed,High (predicted) | CREB1 | AMPK Signaling,ATM Signaling,B Cell Receptor Signaling,BMP signaling pathway,Calcium Signaling,cAMP-mediated signaling,Cardiac Hypertrophy Signaling,Circadian Rhythm Signaling,Corticotropin Releasing Hormone Signaling,CREB Signaling in Neurons,Dendritic Cell Maturation,Dopamine-DARPP32 Feedback in cAMP Signaling,Ephrin Receptor Signaling,ERK/MAPK Signaling,ERK5 Signaling,Estrogen-Dependent Breast Cancer Signaling,FGF Signaling,FLT3 Signaling in Hematopoietic Progenitor Cells,G-Protein Coupled Receptor Signaling,GDNF Family Ligand-Receptor Interactions,Glucocorticoid Receptor Signaling,GNRH Signaling,Gαs Signaling,Huntington's Disease Signaling,Hypoxia Signaling in the Cardiovascular System,ILK Signaling,LPS-stimulated MAPK Signaling,Melanocyte Development and Pigmentation Signaling,Neuropathic Pain Signaling In Dorsal Horn Neurons,Neuroprotective Role of THOP1 in Alzheimer's Disease,Neurotrophin/TRK Signaling,NGF Signaling,P2Y Purigenic Receptor Signaling Pathway,p38 MAPK Signaling,Phospholipase C Signaling,PI3K Signaling in B Lymphocytes,Prostate Cancer Signaling,Protein Kinase A Signaling,Relaxin Signaling,Role of IL-17F in Allergic Inflammatory Airway Diseases,Role of Macrophages, Fibroblasts and Endothelial Cells in Rheumatoid Arthritis,Role of Pattern Recognition Receptors in Recognition of Bacteria and Viruses,Synaptic Long Term Potentiation,Thrombin Signaling,Wnt/Ca+ pathway |
| MIMAT0000070 | miR-17-5p (and other miRNAs w/seed AAAGUGC) | TargetScan Human | High (predicted) | CREB5 | AMPK Signaling,ATM Signaling,B Cell Receptor Signaling,Calcium Signaling,cAMP-mediated signaling,Circadian Rhythm Signaling,Corticotropin Releasing Hormone Signaling,CREB Signaling in Neurons,Dendritic Cell Maturation,Dopamine-DARPP32 Feedback in cAMP Signaling,Ephrin Receptor Signaling,ERK/MAPK Signaling,ERK5 Signaling,Estrogen-Dependent Breast Cancer Signaling,FGF Signaling,FLT3 Signaling in Hematopoietic Progenitor Cells,G-Protein Coupled Receptor Signaling,GNRH Signaling,Gαs Signaling,Huntington's Disease Signaling,Hypoxia Signaling in the Cardiovascular System,ILK Signaling,Melanocyte Development and Pigmentation Signaling,Neurotrophin/TRK Signaling,NGF Signaling,P2Y Purigenic Receptor Signaling Pathway,p38 MAPK Signaling,Phospholipase C Signaling,Prostate Cancer Signaling,Protein Kinase A Signaling,Role of IL-17F in Allergic Inflammatory Airway Diseases,Role of Macrophages, Fibroblasts and Endothelial Cells in Rheumatoid Arthritis,Synaptic Long Term Potentiation,Wnt/Ca+ pathway |
| MIMAT0000070 | miR-17-5p (and other miRNAs w/seed | TargetScan Human | High (predicted) | CRK | Actin Cytoskeleton Signaling,Angiopoietin Signaling,Axonal Guidance Signaling,Chronic Myeloid Leukemia Signaling,CXCR4 Signaling,Ephrin Receptor Signaling,Epithelial Adherens Junction Signaling,ERK/MAPK Signaling,FAK Signaling,Fcγ Receptor-mediated Phagocytosis in Macrophages |

| | | | | | |
|---|---|---|---|---|---|
| | AAAGUGC) | | | | and Monocytes,FGF Signaling,Insulin Receptor Signaling,Integrin Signaling,Leukocyte Extravasation Signaling,Melanocyte Development and Pigmentation Signaling,Molecular Mechanisms of Cancer,Neuregulin Signaling,NGF Signaling,Paxillin Signaling,PDGF Signaling,Renal Cell Carcinoma Signaling,Role of PI3K/AKT Signaling in the Pathogenesis of Influenza,SAPK/JNK Signaling |
| MIMAT0000070 | miR-17-5p (and other miRNAs w/seed AAAGUGC) | TargetScan Human | High (predicted) | CRY2 | Circadian Rhythm Signaling |
| MIMAT0000070 | miR-17-5p (and other miRNAs w/seed AAAGUGC) | TargetScan Human | High (predicted) | CSNK1G1 | Dopamine-DARPP32 Feedback in cAMP Signaling,Gap Junction Signaling,Role of NFAT in Regulation of the Immune Response,Role of Wnt/GSK-3β Signaling in the Pathogenesis of Influenza,Wnt/β-catenin Signaling |
| MIMAT0000070 | miR-17-5p (and other miRNAs w/seed AAAGUGC) | TargetScan Human,miRecords | Experimentally Observed,High (predicted) | CXCL8 | Agranulocyte Adhesion and Diapedesis,Airway Pathology in Chronic Obstructive Pulmonary Disease,Atherosclerosis Signaling,Bladder Cancer Signaling,Communication between Innate and Adaptive Immune Cells,Glucocorticoid Receptor Signaling,Granulocyte Adhesion and Diapedesis,Hematopoiesis from Pluripotent Stem Cells,Hepatic Cholestasis,Hepatic Fibrosis / Hepatic Stellate Cell Activation,HMGB1 Signaling,IL-15 Signaling,IL-17 Signaling,IL-17A Signaling in Gastric Cells,IL-6 Signaling,IL-8 Signaling,Role of Cytokines in Mediating Communication between Immune Cells,Role of Hypercytokinemia/hyperchemokinemia in the Pathogenesis of Influenza,Role of IL-17A in Arthritis,Role of IL-17A in Psoriasis,Role of IL-17F in Allergic Inflammatory Airway Diseases,Role of Macrophages, Fibroblasts and Endothelial Cells in Rheumatoid Arthritis,Role of Pattern Recognition Receptors in Recognition of Bacteria and Viruses,Role of Tissue Factor in Cancer,TREM1 Signaling |
| MIMAT0000070 | miR-17-5p (and other miRNAs w/seed AAAGUGC) | TargetScan Human | High (predicted) | DNAJB9 | Aldosterone Signaling in Epithelial Cells,NRF2-mediated Oxidative Stress Response,Protein Ubiquitination Pathway,Unfolded protein response |
| MIMAT0000070 | miR-17-5p (and other miRNAs w/seed AAAGUGC) | TargetScan Human | High (predicted) | DNM2 | Caveolar-mediated Endocytosis Signaling,Clathrin-mediated Endocytosis Signaling,eNOS Signaling,G Beta Gamma Signaling,GNRH Signaling,Huntington's Disease Signaling,Remodeling of Epithelial Adherens Junctions,Virus Entry via Endocytic Pathways |

| | | | | | |
|---|---|---|---|---|---|
| MIMAT0000070 | miR-17-5p (and other miRNAs w/seed AAAGUGC) | TargetScan Human | High (predicted) | DOK6 | GDNF Family Ligand-Receptor Interactions |
| MIMAT0000070 | miR-17-5p (and other miRNAs w/seed AAAGUGC) | TargetScan Human | High (predicted) | DPYSL2 | Axonal Guidance Signaling,Semaphorin Signaling in Neurons,Thymine Degradation,Uracil Degradation II (Reductive) |
| MIMAT0000070 | miR-17-5p (and other miRNAs w/seed AAAGUGC) | TargetScan Human | High (predicted) | DPYSL5 | Axonal Guidance Signaling,Semaphorin Signaling in Neurons |
| MIMAT0000070 | miR-17-5p (and other miRNAs w/seed AAAGUGC) | TargetScan Human | High (predicted) | DRD1 | cAMP-mediated signaling,CDK5 Signaling,Dopamine Receptor Signaling,Dopamine-DARPP32 Feedback in cAMP Signaling,G-Protein Coupled Receptor Signaling,Gap Junction Signaling,Gαs Signaling |
| MIMAT0000070 | miR-17-5p (and other miRNAs w/seed AAAGUGC) | TargetScan Human | Moderate (predicted) | DUSP18 | Protein Kinase A Signaling |
| MIMAT0000070 | miR-17-5p (and other miRNAs w/seed AAAGUGC) | TargetScan Human | High (predicted) | DUSP2 | 3-phosphoinositide Biosynthesis,3-phosphoinositide Degradation,D-myo-inositol (1,4,5,6)-Tetrakisphosphate Biosynthesis,D-myo-inositol (3,4,5,6)-tetrakisphosphate Biosynthesis,D-myo-inositol-5-phosphate Metabolism,ERK/MAPK Signaling,Protein Kinase A Signaling,Superpathway of Inositol Phosphate Compounds |
| MIMAT0000070 | miR-17-5p (and other miRNAs w/seed AAAGUGC) | TargetScan Human | High (predicted) | DUSP8 | 3-phosphoinositide Biosynthesis,3-phosphoinositide Degradation,D-myo-inositol (1,4,5,6)-Tetrakisphosphate Biosynthesis,D-myo-inositol (3,4,5,6)-tetrakisphosphate Biosynthesis,D-myo-inositol-5-phosphate Metabolism,Protein Kinase A Signaling,SAPK/JNK Signaling,Superpathway of Inositol Phosphate Compounds |
| MIMAT0000070 | miR-17-5p (and other miRNAs w/seed AAAGUGC) | TargetScan Human | High (predicted) | EFNB1 | Axonal Guidance Signaling,Ephrin B Signaling,Ephrin Receptor Signaling,PCP pathway |

| | | | | | |
|---|---|---|---|---|---|
| MIMAT0000070 | miR-17-5p (and other miRNAs w/seed AAAGUGC) | TargetScan Human | High (predicted) | EFNB2 | Axonal Guidance Signaling,Ephrin B Signaling,Ephrin Receptor Signaling |
| MIMAT0000070 | miR-17-5p (and other miRNAs w/seed AAAGUGC) | TargetScan Human | High (predicted) | ELK3 | ERK/MAPK Signaling,HGF Signaling,Telomerase Signaling |
| MIMAT0000070 | miR-17-5p (and other miRNAs w/seed AAAGUGC) | TargetScan Human | High (predicted) | EPHA4 | Axonal Guidance Signaling,Cholecystokinin/Gastrin-mediated Signaling,Ephrin A Signaling,Ephrin Receptor Signaling |
| MIMAT0000070 | miR-17-5p (and other miRNAs w/seed AAAGUGC) | TargetScan Human | High (predicted) | EPHA5 | Axonal Guidance Signaling,Ephrin A Signaling,Ephrin Receptor Signaling |
| MIMAT0000070 | miR-17-5p (and other miRNAs w/seed AAAGUGC) | TargetScan Human | High (predicted) | EPHA7 | Axonal Guidance Signaling,Ephrin A Signaling,Ephrin Receptor Signaling |
| MIMAT0000070 | miR-17-5p (and other miRNAs w/seed AAAGUGC) | TargetScan Human | High (predicted) | EPHB4 | Axonal Guidance Signaling,Ephrin B Signaling,Ephrin Receptor Signaling |
| MIMAT0000070 | miR-17-5p (and other miRNAs w/seed AAAGUGC) | TargetScan Human | High (predicted) | ERBB3 | Agrin Interactions at Neuromuscular Junction,ErbB Signaling,ErbB2-ErbB3 Signaling,HER-2 Signaling in Breast Cancer,Neuregulin Signaling |
| MIMAT0000070 | miR-17-5p (and other miRNAs w/seed AAAGUGC) | TargetScan Human | High (predicted) | EREG | ErbB Signaling,Neuregulin Signaling |
| MIMAT0000070 | miR-17-5p (and other miRNAs | TargetScan Human | High (predicted) | ERO1B | Unfolded protein response |

| | | | | | |
|---|---|---|---|---|---|
| | w/seed AAAGUGC) | | | | |
| MIMAT0000070 | miR-17-5p (and other miRNAs w/seed AAAGUGC) | TargetScan Human,miRecords | Experimentally Observed,Moderate (predicted) | ESR1 | Aryl Hydrocarbon Receptor Signaling,eNOS Signaling,ERK/MAPK Signaling,Estrogen Receptor Signaling,Estrogen-Dependent Breast Cancer Signaling,Estrogen-mediated S-phase Entry,Glucocorticoid Receptor Signaling,Hepatic Cholestasis,RhoGDI Signaling |
| MIMAT0000070 | miR-17-5p (and other miRNAs w/seed AAAGUGC) | TargetScan Human | High (predicted) | F2R | Actin Cytoskeleton Signaling,Clathrin-mediated Endocytosis Signaling,Coagulation System,Glioma Invasiveness Signaling,Gα12/13 Signaling,p70S6K Signaling,Thrombin Signaling |
| MIMAT0000070 | miR-17-5p (and other miRNAs w/seed AAAGUGC) | TargetScan Human | Moderate (predicted) | F2RL3 | Gα12/13 Signaling,p70S6K Signaling,Thrombin Signaling |
| MIMAT0000070 | miR-17-5p (and other miRNAs w/seed AAAGUGC) | TargetScan Human | High (predicted) | FGF4 | Actin Cytoskeleton Signaling,Bladder Cancer Signaling,Clathrin-mediated Endocytosis Signaling,FGF Signaling,Human Embryonic Stem Cell Pluripotency,Regulation of the Epithelial-Mesenchymal Transition Pathway,Role of Oct4 in Mammalian Embryonic Stem Cell Pluripotency |
| MIMAT0000070 | miR-17-5p (and other miRNAs w/seed AAAGUGC) | TargetScan Human | Moderate (predicted) | FKBP5 | Glucocorticoid Receptor Signaling,NRF2-mediated Oxidative Stress Response |
| MIMAT0000070 | miR-17-5p (and other miRNAs w/seed AAAGUGC) | TargetScan Human | High (predicted) | FLT1 | eNOS Signaling,Hepatic Fibrosis / Hepatic Stellate Cell Activation,IL-8 Signaling,NF-κB Signaling,Nitric Oxide Signaling in the Cardiovascular System,PTEN Signaling,STAT3 Pathway,VEGF Family Ligand-Receptor Interactions,VEGF Signaling |
| MIMAT0000070 | miR-17-5p (and other miRNAs w/seed AAAGUGC) | TargetScan Human | High (predicted) | FOXA1 | FXR/RXR Activation,Role of Oct4 in Mammalian Embryonic Stem Cell Pluripotency |
| MIMAT0000070 | miR-17-5p (and other miRNAs w/seed | TargetScan Human | High (predicted) | FRS2 | FGF Signaling,GDNF Family Ligand-Receptor Interactions,Neurotrophin/TRK Signaling,Regulation of the Epithelial-Mesenchymal Transition Pathway |

| | | | | | |
|---|---|---|---|---|---|
| | AAAGUGC) | | | | |
| MIMAT0000070 | miR-17-5p (and other miRNAs w/seed AAAGUGC) | TargetScan Human | High (predicted) | FZD3 | Adipogenesis pathway,Axonal Guidance Signaling,Basal Cell Carcinoma Signaling,Colorectal Cancer Metastasis Signaling,Factors Promoting Cardiogenesis in Vertebrates,Glioblastoma Multiforme Signaling,Human Embryonic Stem Cell Pluripotency,Molecular Mechanisms of Cancer,Mouse Embryonic Stem Cell Pluripotency,Ovarian Cancer Signaling,PCP pathway,Regulation of the Epithelial-Mesenchymal Transition Pathway,Role of Macrophages, Fibroblasts and Endothelial Cells in Rheumatoid Arthritis,Role of NANOG in Mammalian Embryonic Stem Cell Pluripotency,Role of Osteoblasts, Osteoclasts and Chondrocytes in Rheumatoid Arthritis,Role of Wnt/GSK-3β Signaling in the Pathogenesis of Influenza,Wnt/Ca+ pathway,Wnt/β-catenin Signaling |
| MIMAT0000070 | miR-17-5p (and other miRNAs w/seed AAAGUGC) | TargetScan Human | High (predicted) | FZD4 | Adipogenesis pathway,Axonal Guidance Signaling,Basal Cell Carcinoma Signaling,Colorectal Cancer Metastasis Signaling,Factors Promoting Cardiogenesis in Vertebrates,Glioblastoma Multiforme Signaling,Human Embryonic Stem Cell Pluripotency,Molecular Mechanisms of Cancer,Mouse Embryonic Stem Cell Pluripotency,Ovarian Cancer Signaling,PCP pathway,Regulation of the Epithelial-Mesenchymal Transition Pathway,Role of Macrophages, Fibroblasts and Endothelial Cells in Rheumatoid Arthritis,Role of NANOG in Mammalian Embryonic Stem Cell Pluripotency,Role of Osteoblasts, Osteoclasts and Chondrocytes in Rheumatoid Arthritis,Role of Wnt/GSK-3β Signaling in the Pathogenesis of Influenza,Wnt/Ca+ pathway,Wnt/β-catenin Signaling |
| MIMAT0000070 | miR-17-5p (and other miRNAs w/seed AAAGUGC) | TargetScan Human | High (predicted) | FZD7 | Adipogenesis pathway,Axonal Guidance Signaling,Basal Cell Carcinoma Signaling,Colorectal Cancer Metastasis Signaling,Factors Promoting Cardiogenesis in Vertebrates,Glioblastoma Multiforme Signaling,Human Embryonic Stem Cell Pluripotency,Molecular Mechanisms of Cancer,Mouse Embryonic Stem Cell Pluripotency,Ovarian Cancer Signaling,PCP pathway,Regulation of the Epithelial-Mesenchymal Transition Pathway,Role of Macrophages, Fibroblasts and Endothelial Cells in Rheumatoid Arthritis,Role of NANOG in Mammalian Embryonic Stem Cell Pluripotency,Role of Osteoblasts, Osteoclasts and Chondrocytes in Rheumatoid Arthritis,Role of Wnt/GSK-3β Signaling in the Pathogenesis of Influenza,Wnt/Ca+ pathway,Wnt/β-catenin Signaling |
| MIMAT0000070 | miR-17-5p (and other miRNAs w/seed | TargetScan Human | High (predicted) | GAB1 | B Cell Receptor Signaling,Endothelin-1 Signaling,ERK5 Signaling,Fc Epsilon RI Signaling,FGF Signaling,GDNF Family Ligand-Receptor Interactions,HGF Signaling,Insulin Receptor Signaling,Molecular Mechanisms of |

| | | | | | |
|---|---|---|---|---|---|
| | AAAGUGC) | | | | Cancer,Neurotrophin/TRK Signaling,NGF Signaling,PI3K/AKT Signaling,Regulation of the Epithelial-Mesenchymal Transition Pathway,Renal Cell Carcinoma Signaling,Role of NANOG in Mammalian Embryonic Stem Cell Pluripotency,SAPK/JNK Signaling |
| MIMAT0000070 | miR-17-5p (and other miRNAs w/seed AAAGUGC) | TargetScan Human | High (predicted) | GABBR1 | cAMP-mediated signaling,G-Protein Coupled Receptor Signaling,GABA Receptor Signaling,Gαi Signaling |
| MIMAT0000070 | miR-17-5p (and other miRNAs w/seed AAAGUGC) | TargetScan Human | High (predicted) | GABBR2 | cAMP-mediated signaling,G-Protein Coupled Receptor Signaling,GABA Receptor Signaling,Gαi Signaling |
| MIMAT0000070 | miR-17-5p (and other miRNAs w/seed AAAGUGC) | TargetScan Human | High (predicted) | GABPB1 | Agrin Interactions at Neuromuscular Junction |
| MIMAT0000070 | miR-17-5p (and other miRNAs w/seed AAAGUGC) | TargetScan Human | Moderate (predicted) | GABRA1 | GABA Receptor Signaling |
| MIMAT0000070 | miR-17-5p (and other miRNAs w/seed AAAGUGC) | TargetScan Human | Moderate (predicted) | GNB4 | Androgen Signaling,Antiproliferative Role of Somatostatin Receptor 2,Axonal Guidance Signaling,Breast Cancer Regulation by Stathmin1,Cardiac Hypertrophy Signaling,Cardiac β-adrenergic Signaling,CCR3 Signaling in Eosinophils,CCR5 Signaling in Macrophages,Colorectal Cancer Metastasis Signaling,CREB Signaling in Neurons,CXCR4 Signaling,Ephrin B Signaling,Ephrin Receptor Signaling,fMLP Signaling in Neutrophils,G Beta Gamma Signaling,G Protein Signaling Mediated by Tubby,Gαi Signaling,Gαq Signaling,Gαs Signaling,Huntington's Disease Signaling,IL-1 Signaling,IL-8 Signaling,P2Y Purigenic Receptor Signaling Pathway,Phospholipase C Signaling,Protein Kinase A Signaling,Relaxin Signaling,RhoGDI Signaling,Role of NFAT in Cardiac Hypertrophy,Role of NFAT in Regulation of the Immune Response,Signaling by Rho Family GTPases,Tec Kinase Signaling,Thrombin Signaling,α-Adrenergic Signaling |
| MIMAT0000070 | miR-17-5p (and other miRNAs | TargetScan Human | High (predicted) | GNB5 | Androgen Signaling,Antiproliferative Role of Somatostatin Receptor 2,Axonal Guidance Signaling,Breast Cancer Regulation by Stathmin1,Cardiac |

| | | | | | |
|---|---|---|---|---|---|
| | w/seed AAAGUGC) | | | | Hypertrophy Signaling,Cardiac β-adrenergic Signaling,CCR3 Signaling in Eosinophils,CCR5 Signaling in Macrophages,Colorectal Cancer Metastasis Signaling,CREB Signaling in Neurons,CXCR4 Signaling,Ephrin B Signaling,Ephrin Receptor Signaling,fMLP Signaling in Neutrophils,G Beta Gamma Signaling,G Protein Signaling Mediated by Tubby,Gαi Signaling,Gαq Signaling,Gαs Signaling,Huntington's Disease Signaling,IL-1 Signaling,IL-8 Signaling,P2Y Purigenic Receptor Signaling Pathway,Phospholipase C Signaling,Phototransduction Pathway,Protein Kinase A Signaling,Relaxin Signaling,RhoGDI Signaling,Role of NFAT in Cardiac Hypertrophy,Role of NFAT in Regulation of the Immune Response,Signaling by Rho Family GTPases,Tec Kinase Signaling,Thrombin Signaling,α-Adrenergic Signaling |
| MIMAT0000070 | miR-17-5p (and other miRNAs w/seed AAAGUGC) | TargetScan Human | High (predicted) | GUCY1A3 | Antiproliferative Role of Somatostatin Receptor 2,Cellular Effects of Sildenafil (Viagra),Corticotropin Releasing Hormone Signaling,Dopamine-DARPP32 Feedback in cAMP Signaling,Endothelin-1 Signaling,eNOS Signaling,Gap Junction Signaling,Inhibition of Angiogenesis by TSP1,Nitric Oxide Signaling in the Cardiovascular System,Phototransduction Pathway,Relaxin Signaling,Sertoli Cell-Sertoli Cell Junction Signaling,Sperm Motility,Synaptic Long Term Depression |
| MIMAT0000070 | miR-17-5p (and other miRNAs w/seed AAAGUGC) | TargetScan Human | High (predicted) | HDAC4 | Adipogenesis pathway,Calcium Signaling,Cell Cycle: G1/S Checkpoint Regulation,Chronic Myeloid Leukemia Signaling,Cyclins and Cell Cycle Regulation,Hereditary Breast Cancer Signaling,Huntington's Disease Signaling,Phospholipase C Signaling,Role of NFAT in Cardiac Hypertrophy,Telomerase Signaling,Xenobiotic Metabolism Signaling |
| MIMAT0000070 | miR-17-5p (and other miRNAs w/seed AAAGUGC) | TargetScan Human | High (predicted) | HIF1A | Adipogenesis pathway,HIF1α Signaling,Hypoxia Signaling in the Cardiovascular System,ILK Signaling,Molecular Mechanisms of Cancer,mTOR Signaling,p53 Signaling,Regulation of the Epithelial-Mesenchymal Transition Pathway,Renal Cell Carcinoma Signaling,TR/RXR Activation,VEGF Signaling |
| MIMAT0000070 | miR-17-5p (and other miRNAs w/seed AAAGUGC) | TargetScan Human | High (predicted) | HSPA6 | Aldosterone Signaling in Epithelial Cells,eNOS Signaling,Glucocorticoid Receptor Signaling,Huntington's Disease Signaling,Protein Ubiquitination Pathway,Unfolded protein response |
| MIMAT0000070 | miR-17-5p (and other miRNAs w/seed AAAGUGC) | TargetScan Human | High (predicted) | HSPA8 | Aldosterone Signaling in Epithelial Cells,Clathrin-mediated Endocytosis Signaling,eNOS Signaling,Glucocorticoid Receptor Signaling,Huntington's Disease Signaling,Protein Ubiquitination Pathway,Unfolded protein response |

| | | | | | |
|---|---|---|---|---|---|
| MIMAT0000070 | miR-17-5p (and other miRNAs w/seed AAAGUGC) | TargetScan Human | High (predicted) | IGF2BP1 | Role of Oct4 in Mammalian Embryonic Stem Cell Pluripotency |
| MIMAT0000070 | miR-17-5p (and other miRNAs w/seed AAAGUGC) | TargetScan Human | High (predicted) | IL6ST | Acute Phase Response Signaling,CNTF Signaling,Colorectal Cancer Metastasis Signaling,ERK5 Signaling,IL-6 Signaling,Mouse Embryonic Stem Cell Pluripotency,Oncostatin M Signaling,Role of JAK family kinases in IL-6-type Cytokine Signaling,Role of Macrophages, Fibroblasts and Endothelial Cells in Rheumatoid Arthritis,Role of NANOG in Mammalian Embryonic Stem Cell Pluripotency,Role of NFAT in Cardiac Hypertrophy,T Helper Cell Differentiation |
| MIMAT0000070 | miR-17-5p (and other miRNAs w/seed AAAGUGC) | TargetScan Human | High (predicted) | ITGA4 | Actin Cytoskeleton Signaling,Actin Nucleation by ARP-WASP Complex,Agranulocyte Adhesion and Diapedesis,Agrin Interactions at Neuromuscular Junction,Atherosclerosis Signaling,Axonal Guidance Signaling,Caveolar-mediated Endocytosis Signaling,Cdc42 Signaling,Ephrin Receptor Signaling,ERK/MAPK Signaling,FAK Signaling,Granulocyte Adhesion and Diapedesis,HGF Signaling,Integrin Signaling,Leukocyte Extravasation Signaling,Molecular Mechanisms of Cancer,Neuregulin Signaling,NF-κB Activation by Viruses,PAK Signaling,Paxillin Signaling,phagosome formation,Phospholipase C Signaling,PI3K/AKT Signaling,PTEN Signaling,Rac Signaling,Reelin Signaling in Neurons,Regulation of Actin-based Motility by Rho,Regulation of Cellular Mechanics by Calpain Protease,Regulation of eIF4 and p70S6K Signaling,RhoGDI Signaling,Sertoli Cell-Sertoli Cell Junction Signaling,Signaling by Rho Family GTPases,Tec Kinase Signaling,Virus Entry via Endocytic Pathways |
| MIMAT0000070 | miR-17-5p (and other miRNAs w/seed AAAGUGC) | TargetScan Human | High (predicted) | ITPR2 | Aldosterone Signaling in Epithelial Cells,Breast Cancer Regulation by Stathmin1,Calcium Signaling,Calcium-induced T Lymphocyte Apoptosis,CCR3 Signaling in Eosinophils,CD28 Signaling in T Helper Cells,Cellular Effects of Sildenafil (Viagra),Cholecystokinin/Gastrin-mediated Signaling,Corticotropin Releasing Hormone Signaling,CREB Signaling in Neurons,CXCR4 Signaling,Dopamine-DARPP32 Feedback in cAMP Signaling,EGF Signaling,Endothelin-1 Signaling,eNOS Signaling,fMLP Signaling in Neutrophils,Gap Junction Signaling,GDNF Family Ligand-Receptor Interactions,Glioblastoma Multiforme Signaling,GNRH Signaling,GPCR-Mediated Integration of Enteroendocrine Signaling Exemplified by an L Cell,GPCR-Mediated Nutrient Sensing in Enteroendocrine Cells,Gustation Pathway,Gαq Signaling,iCOS-iCOSL Signaling in T Helper Cells,Neuropathic Pain Signaling In Dorsal Horn Neurons,Nitric Oxide Signaling in the |

| | | | | | |
|---|---|---|---|---|---|
| | | | | | Cardiovascular System,Non-Small Cell Lung Cancer Signaling,Phospholipase C Signaling,PI3K Signaling in B Lymphocytes,Protein Kinase A Signaling,Renin-Angiotensin Signaling,Role of NFAT in Cardiac Hypertrophy,Role of NFAT in Regulation of the Immune Response,Sperm Motility,Synaptic Long Term Depression,Synaptic Long Term Potentiation,Thrombin Signaling,α-Adrenergic Signaling |
| MIMAT0000070 | miR-17-5p (and other miRNAs w/seed AAAGUGC) | TargetScan Human | Moderate (predicted) | ITSN2 | Ephrin B Signaling |
| MIMAT0000070 | miR-17-5p (and other miRNAs w/seed AAAGUGC) | TargetScan Human,miRecords | Experimentally Observed,High (predicted) | JAK1 | CNTF Signaling,Colorectal Cancer Metastasis Signaling,EGF Signaling,Glucocorticoid Receptor Signaling,IGF-1 Signaling,IL-10 Signaling,IL-15 Production,IL-15 Signaling,IL-17 Signaling,IL-17A Signaling in Airway Cells,IL-2 Signaling,IL-22 Signaling,IL-3 Signaling,IL-4 Signaling,IL-9 Signaling,iNOS Signaling,Insulin Receptor Signaling,Interferon Signaling,JAK/Stat Signaling,Molecular Mechanisms of Cancer,Mouse Embryonic Stem Cell Pluripotency,Oncostatin M Signaling,p70S6K Signaling,Pancreatic Adenocarcinoma Signaling,PDGF Signaling,PI3K/AKT Signaling,Production of Nitric Oxide and Reactive Oxygen Species in Macrophages,Regulation of the Epithelial-Mesenchymal Transition Pathway,Role of JAK family kinases in IL-6-type Cytokine Signaling,Role of JAK1 and JAK3 in γc Cytokine Signaling,Role of JAK1, JAK2 and TYK2 in Interferon Signaling,Role of JAK2 in Hormone-like Cytokine Signaling,Role of NANOG in Mammalian Embryonic Stem Cell Pluripotency,Tec Kinase Signaling,Type I Diabetes Mellitus Signaling |
| MIMAT0000070 | miR-17-5p (and other miRNAs w/seed AAAGUGC) | TargetScan Human | High (predicted) | KAT2B | AMPK Signaling,Androgen Signaling,Cell Cycle: G2/M DNA Damage Checkpoint Regulation,Estrogen Receptor Signaling,Glucocorticoid Receptor Signaling,HMGB1 Signaling,p53 Signaling,RAR Activation |
| MIMAT0000070 | miR-17-5p (and other miRNAs w/seed AAAGUGC) | TargetScan Human | High (predicted) | KCNJ10 | Dopamine-DARPP32 Feedback in cAMP Signaling |
| MIMAT0000070 | miR-17-5p (and other miRNAs w/seed | TargetScan Human | High (predicted) | KCNJ8 | Dopamine-DARPP32 Feedback in cAMP Signaling |

| | | | | | |
|---|---|---|---|---|---|
| | AAAGUGC) | | | | |
| MIMAT0000070 | miR-17-5p (and other miRNAs w/seed AAAGUGC) | TargetScan Human | High (predicted) | LIF | ERK5 Signaling,Hematopoiesis from Pluripotent Stem Cells,Hepatic Cholestasis,HMGB1 Signaling,Mouse Embryonic Stem Cell Pluripotency,Role of NANOG in Mammalian Embryonic Stem Cell Pluripotency,Role of NFAT in Cardiac Hypertrophy,Role of Pattern Recognition Receptors in Recognition of Bacteria and Viruses |
| MIMAT0000070 | miR-17-5p (and other miRNAs w/seed AAAGUGC) | TargetScan Human | High (predicted) | LIMK1 | Actin Cytoskeleton Signaling,Axonal Guidance Signaling,Breast Cancer Regulation by Stathmin1,CCR3 Signaling in Eosinophils,Cdc42 Signaling,Chemokine Signaling,Death Receptor Signaling,Ephrin A Signaling,Ephrin B Signaling,Ephrin Receptor Signaling,Germ Cell-Sertoli Cell Junction Signaling,IL-8 Signaling,PAK Signaling,Pyridoxal 5'-phosphate Salvage Pathway,Rac Signaling,Regulation of Actin-based Motility by Rho,RhoA Signaling,RhoGDI Signaling,Role of Tissue Factor in Cancer,Salvage Pathways of Pyrimidine Ribonucleotides,Semaphorin Signaling in Neurons,Signaling by Rho Family GTPases |
| MIMAT0000070 | miR-17-5p (and other miRNAs w/seed AAAGUGC) | TargetScan Human | High (predicted) | LRP8 | Reelin Signaling in Neurons |
| MIMAT0000070 | miR-17-5p (and other miRNAs w/seed AAAGUGC) | TargetScan Human | High (predicted) | MAP3K1 | Acute Phase Response Signaling,April Mediated Signaling,B Cell Activating Factor Signaling,B Cell Receptor Signaling,Cardiac Hypertrophy Signaling,CD27 Signaling in Lymphocytes,CD28 Signaling in T Helper Cells,Ceramide Signaling,EGF Signaling,FGF Signaling,Germ Cell-Sertoli Cell Junction Signaling,Glucocorticoid Receptor Signaling,GNRH Signaling,Gα12/13 Signaling,HGF Signaling,IL-1 Signaling,LPS/IL-1 Mediated Inhibition of RXR Function,NF-κB Activation by Viruses,NF-κB Signaling,NGF Signaling,NRF2-mediated Oxidative Stress Response,PDGF Signaling,PKCθ Signaling in T Lymphocytes,Production of Nitric Oxide and Reactive Oxygen Species in Macrophages,Protein Kinase A Signaling,Rac Signaling,RANK Signaling in Osteoclasts,RAR Activation,Regulation of IL-2 Expression in Activated and Anergic T Lymphocytes,Renin-Angiotensin Signaling,Role of NFAT in Cardiac Hypertrophy,SAPK/JNK Signaling,Sertoli Cell-Sertoli Cell Junction Signaling,T Cell Receptor Signaling,TNFR1 Signaling,TNFR2 Signaling,Toll-like Receptor Signaling,Type II Diabetes Mellitus Signaling,Xenobiotic Metabolism Signaling |
| MIMAT0000070 | miR-17-5p (and other miRNAs | TargetScan Human | High (predicted) | MAP3K11 | B Cell Receptor Signaling,Cardiac Hypertrophy Signaling,CD27 Signaling in Lymphocytes,Cdc42 Signaling,Germ Cell-Sertoli Cell Junction Signaling,GNRH |

| | | | | | |
|---|---|---|---|---|---|
| | w/seed AAAGUGC) | | | | Signaling,HGF Signaling,IL-15 Production,Integrin Signaling,NGF Signaling,PKCθ Signaling in T Lymphocytes,Production of Nitric Oxide and Reactive Oxygen Species in Macrophages,Rac Signaling,RANK Signaling in Osteoclasts,Reelin Signaling in Neurons,SAPK/JNK Signaling,Sertoli Cell-Sertoli Cell Junction Signaling,Signaling by Rho Family GTPases,Sperm Motility,STAT3 Pathway,Xenobiotic Metabolism Signaling |
| MIMAT0000070 | miR-17-5p (and other miRNAs w/seed AAAGUGC) | TargetScan Human,miRecords | Experimentally Observed,High (predicted) | MAP3K12 | B Cell Receptor Signaling,Cardiac Hypertrophy Signaling,CD27 Signaling in Lymphocytes,Germ Cell-Sertoli Cell Junction Signaling,GNRH Signaling,HGF Signaling,NGF Signaling,PKCθ Signaling in T Lymphocytes,Production of Nitric Oxide and Reactive Oxygen Species in Macrophages,RANK Signaling in Osteoclasts,SAPK/JNK Signaling,Sertoli Cell-Sertoli Cell Junction Signaling,Signaling by Rho Family GTPases,STAT3 Pathway,Xenobiotic Metabolism Signaling |
| MIMAT0000070 | miR-17-5p (and other miRNAs w/seed AAAGUGC) | TargetScan Human | High (predicted) | MAP3K13 | B Cell Receptor Signaling,Cardiac Hypertrophy Signaling,CD27 Signaling in Lymphocytes,Germ Cell-Sertoli Cell Junction Signaling,GNRH Signaling,HGF Signaling,NGF Signaling,PKCθ Signaling in T Lymphocytes,Production of Nitric Oxide and Reactive Oxygen Species in Macrophages,RANK Signaling in Osteoclasts,SAPK/JNK Signaling,Sertoli Cell-Sertoli Cell Junction Signaling,Xenobiotic Metabolism Signaling |
| MIMAT0000070 | miR-17-5p (and other miRNAs w/seed AAAGUGC) | TargetScan Human | High (predicted) | MAP3K14 | 4-1BB Signaling in T Lymphocytes,Acute Phase Response Signaling,Altered T Cell and B Cell Signaling in Rheumatoid Arthritis,Apoptosis Signaling,April Mediated Signaling,B Cell Activating Factor Signaling,B Cell Receptor Signaling,Cardiac Hypertrophy Signaling,CD27 Signaling in Lymphocytes,CD40 Signaling,Death Receptor Signaling,Dendritic Cell Maturation,Ephrin Receptor Signaling,Germ Cell-Sertoli Cell Junction Signaling,Glucocorticoid Receptor Signaling,GNRH Signaling,Hepatic Cholestasis,HGF Signaling,IL-1 Signaling,IL-10 Signaling,IL-17 Signaling,IL-6 Signaling,Induction of Apoptosis by HIV1,LPS-stimulated MAPK Signaling,Lymphotoxin β Receptor Signaling,NF-κB Activation by Viruses,NF-κB Signaling,NGF Signaling,PKCθ Signaling in T Lymphocytes,PPAR Signaling,PPARα/RXRα Activation,Production of Nitric Oxide and Reactive Oxygen Species in Macrophages,RANK Signaling in Osteoclasts,Role of Macrophages, Fibroblasts and Endothelial Cells in Rheumatoid Arthritis,Role of Osteoblasts, Osteoclasts and Chondrocytes in Rheumatoid Arthritis,Sertoli Cell-Sertoli Cell Junction Signaling,TNFR1 Signaling,TNFR2 Signaling,Toll-like Receptor Signaling,TWEAK Signaling,Type I Diabetes Mellitus Signaling,Type II Diabetes Mellitus Signaling,Xenobiotic Metabolism Signaling |

| | | | | | |
|---|---|---|---|---|---|
| MIMAT0000070 | miR-17-5p (and other miRNAs w/seed AAAGUGC) | TargetScan Human | High (predicted) | MAP3K2 | B Cell Receptor Signaling,Cardiac Hypertrophy Signaling,Caveolar-mediated Endocytosis Signaling,CD27 Signaling in Lymphocytes,ERK5 Signaling,Gap Junction Signaling,Germ Cell-Sertoli Cell Junction Signaling,GNRH Signaling,HGF Signaling,NGF Signaling,Nur77 Signaling in T Lymphocytes,PKCθ Signaling in T Lymphocytes,Production of Nitric Oxide and Reactive Oxygen Species in Macrophages,RANK Signaling in Osteoclasts,SAPK/JNK Signaling,Sertoli Cell-Sertoli Cell Junction Signaling,Xenobiotic Metabolism Signaling |
| MIMAT0000070 | miR-17-5p (and other miRNAs w/seed AAAGUGC) | TargetScan Human | High (predicted) | MAP3K3 | B Cell Receptor Signaling,Cardiac Hypertrophy Signaling,CD27 Signaling in Lymphocytes,ERK5 Signaling,Germ Cell-Sertoli Cell Junction Signaling,GNRH Signaling,HGF Signaling,NF-κB Signaling,NGF Signaling,Nur77 Signaling in T Lymphocytes,PKCθ Signaling in T Lymphocytes,Production of Nitric Oxide and Reactive Oxygen Species in Macrophages,RANK Signaling in Osteoclasts,SAPK/JNK Signaling,Sertoli Cell-Sertoli Cell Junction Signaling,Xenobiotic Metabolism Signaling |
| MIMAT0000070 | miR-17-5p (and other miRNAs w/seed AAAGUGC) | TargetScan Human | High (predicted) | MAP3K5 | 14-3-3-mediated Signaling,4-1BB Signaling in T Lymphocytes,Acute Phase Response Signaling,Apoptosis Signaling,B Cell Receptor Signaling,Cardiac Hypertrophy Signaling,CD27 Signaling in Lymphocytes,Death Receptor Signaling,Endoplasmic Reticulum Stress Pathway,FGF Signaling,Germ Cell-Sertoli Cell Junction Signaling,GNRH Signaling,Gα12/13 Signaling,HER-2 Signaling in Breast Cancer,HGF Signaling,Induction of Apoptosis by HIV1,LPS-stimulated MAPK Signaling,Molecular Mechanisms of Cancer,Neurotrophin/TRK Signaling,NGF Signaling,NRF2-mediated Oxidative Stress Response,p38 MAPK Signaling,PI3K/AKT Signaling,PKCθ Signaling in T Lymphocytes,Production of Nitric Oxide and Reactive Oxygen Species in Macrophages,RANK Signaling in Osteoclasts,RAR Activation,Role of MAPK Signaling in the Pathogenesis of Influenza,Role of Osteoblasts, Osteoclasts and Chondrocytes in Rheumatoid Arthritis,SAPK/JNK Signaling,Sertoli Cell-Sertoli Cell Junction Signaling,Type I Diabetes Mellitus Signaling,Type II Diabetes Mellitus Signaling,Unfolded protein response,Xenobiotic Metabolism Signaling |
| MIMAT0000070 | miR-17-5p (and other miRNAs w/seed AAAGUGC) | TargetScan Human | High (predicted) | MAP3K8 | B Cell Receptor Signaling,Cardiac Hypertrophy Signaling,CD27 Signaling in Lymphocytes,ERK5 Signaling,G-Protein Coupled Receptor Signaling,Germ Cell-Sertoli Cell Junction Signaling,GNRH Signaling,HGF Signaling,IL-12 Signaling and Production in Macrophages,NF-κB Signaling,NGF Signaling,PI3K/AKT Signaling,PKCθ Signaling in T Lymphocytes,Production of Nitric Oxide and Reactive Oxygen Species in Macrophages,Pyridoxal 5'-phosphate Salvage Pathway,RANK Signaling in Osteoclasts,Salvage Pathways of Pyrimidine |

| | | | | | |
|---|---|---|---|---|---|
| | | | | | Ribonucleotides,Sertoli Cell-Sertoli Cell Junction Signaling,Xenobiotic Metabolism Signaling |
| MIMAT0000070 | miR-17-5p (and other miRNAs w/seed AAAGUGC) | TargetScan Human | High (predicted) | MAP3K9 | B Cell Receptor Signaling,Cardiac Hypertrophy Signaling,CD27 Signaling in Lymphocytes,Germ Cell-Sertoli Cell Junction Signaling,GNRH Signaling,HGF Signaling,NGF Signaling,PKCθ Signaling in T Lymphocytes,Production of Nitric Oxide and Reactive Oxygen Species in Macrophages,Pyridoxal 5'-phosphate Salvage Pathway,RANK Signaling in Osteoclasts,Reelin Signaling in Neurons,Salvage Pathways of Pyrimidine Ribonucleotides,SAPK/JNK Signaling,Sertoli Cell-Sertoli Cell Junction Signaling,Signaling by Rho Family GTPases,STAT3 Pathway,Xenobiotic Metabolism Signaling |
| MIMAT0000070 | miR-17-5p (and other miRNAs w/seed AAAGUGC) | TargetScan Human | High (predicted) | MAPK1 | 14-3-3-mediated Signaling,4-1BB Signaling in T Lymphocytes,Actin Cytoskeleton Signaling,Acute Myeloid Leukemia Signaling,Acute Phase Response Signaling,Agrin Interactions at Neuromuscular Junction,Aldosterone Signaling in Epithelial Cells,AMPK Signaling,Amyloid Processing,Androgen Signaling,Antioxidant Action of Vitamin C,Antiproliferative Role of Somatostatin Receptor 2,Antiproliferative Role of TOB in T Cell Signaling,Apoptosis Signaling,April Mediated Signaling,Aryl Hydrocarbon Receptor Signaling,Axonal Guidance Signaling,B Cell Activating Factor Signaling,B Cell Receptor Signaling,Bladder Cancer Signaling,BMP signaling pathway,Breast Cancer Regulation by Stathmin1,Calcium Signaling,cAMP-mediated signaling,Cardiac Hypertrophy Signaling,CCR3 Signaling in Eosinophils,CCR5 Signaling in Macrophages,CD40 Signaling,Cdc42 Signaling,CDK5 Signaling,Chemokine Signaling,Cholecystokinin/Gastrin-mediated Signaling,Chronic Myeloid Leukemia Signaling,CNTF Signaling,Colorectal Cancer Metastasis Signaling,Corticotropin Releasing Hormone Signaling,CREB Signaling in Neurons,CXCR4 Signaling,Dendritic Cell Maturation,EGF Signaling,EIF2 Signaling,Endometrial Cancer Signaling,Endothelin-1 Signaling,Ephrin B Signaling,Ephrin Receptor Signaling,ErbB Signaling,ErbB2-ErbB3 Signaling,ErbB4 Signaling,ERK/MAPK Signaling,Erythropoietin Signaling,Estrogen Receptor Signaling,Estrogen-Dependent Breast Cancer Signaling,FAK Signaling,Fc Epsilon RI Signaling,Fcγ Receptor-mediated Phagocytosis in Macrophages and Monocytes,FGF Signaling,FLT3 Signaling in Hematopoietic Progenitor Cells,fMLP Signaling in Neutrophils,G Beta Gamma Signaling,G-Protein Coupled Receptor Signaling,Gap Junction Signaling,GDNF Family Ligand-Receptor Interactions,Germ Cell-Sertoli Cell Junction Signaling,Glioblastoma Multiforme Signaling,Glioma Invasiveness Signaling,Glioma Signaling,Glucocorticoid Receptor Signaling,GM-CSF Signaling,GNRH Signaling,Growth Hormone Signaling,Gα12/13 Signaling,Gαi Signaling,Gαq |

| | | | | | |
|---|---|---|---|---|---|
| | | | | | Signaling,Gαs Signaling,HGF Signaling,HIF1α Signaling,HMGB1 Signaling,Huntington's Disease Signaling,IGF-1 Signaling,IL-1 Signaling,IL-10 Signaling,IL-12 Signaling and Production in Macrophages,IL-15 Signaling,IL-17 Signaling,IL-17A Signaling in Airway Cells,IL-17A Signaling in Fibroblasts,IL-17A Signaling in Gastric Cells,IL-2 Signaling,IL-22 Signaling,IL-3 Signaling,IL-6 Signaling,IL-8 Signaling,ILK Signaling,Inhibition of Angiogenesis by TSP1,iNOS Signaling,Insulin Receptor Signaling,Integrin Signaling,JAK/Stat Signaling,Leptin Signaling in Obesity,Leukocyte Extravasation Signaling,LPS-stimulated MAPK Signaling,Lymphotoxin β Receptor Signaling,Melanocyte Development and Pigmentation Signaling,Melanoma Signaling,Melatonin Signaling,MIF Regulation of Innate Immunity,MIF-mediated Glucocorticoid Regulation,Molecular Mechanisms of Cancer,Mouse Embryonic Stem Cell Pluripotency,mTOR Signaling,Natural Killer Cell Signaling,Neuregulin Signaling,Neuropathic Pain Signaling In Dorsal Horn Neurons,Neurotrophin/TRK Signaling,NF-κB Activation by Viruses,NGF Signaling,Nitric Oxide Signaling in the Cardiovascular System,Non-Small Cell Lung Cancer Signaling,NRF2-mediated Oxidative Stress Response,Oncostatin M Signaling,Ovarian Cancer Signaling,P2Y Purigenic Receptor Signaling Pathway,p70S6K Signaling,PAK Signaling,Pancreatic Adenocarcinoma Signaling,Parkinson's Signaling,Paxillin Signaling,PDGF Signaling,PEDF Signaling,Phospholipase C Signaling,PI3K Signaling in B Lymphocytes,PI3K/AKT Signaling,PKCθ Signaling in T Lymphocytes,PPAR Signaling,PPARα/RXRα Activation,Production of Nitric Oxide and Reactive Oxygen Species in Macrophages,Prolactin Signaling,Prostate Cancer Signaling,Protein Kinase A Signaling,PTEN Signaling,Pyridoxal 5'-phosphate Salvage Pathway,Rac Signaling,RANK Signaling in Osteoclasts,RAR Activation,Regulation of Cellular Mechanics by Calpain Protease,Regulation of eIF4 and p70S6K Signaling,Regulation of IL-2 Expression in Activated and Anergic T Lymphocytes,Regulation of the Epithelial-Mesenchymal Transition Pathway,Relaxin Signaling,Renal Cell Carcinoma Signaling,Renin-Angiotensin Signaling,Role of IL-17A in Arthritis,Role of IL-17F in Allergic Inflammatory Airway Diseases,Role of JAK family kinases in IL-6-type Cytokine Signaling,Role of JAK1 and JAK3 in γc Cytokine Signaling,Role of Macrophages, Fibroblasts and Endothelial Cells in Rheumatoid Arthritis,Role of MAPK Signaling in the Pathogenesis of Influenza,Role of NANOG in Mammalian Embryonic Stem Cell Pluripotency,Role of NFAT in Cardiac Hypertrophy,Role of NFAT in Regulation of the Immune Response,Role of Osteoblasts, Osteoclasts and Chondrocytes in Rheumatoid Arthritis,Role of Pattern Recognition Receptors in Recognition of Bacteria and Viruses,Role of PI3K/AKT Signaling in the Pathogenesis of Influenza,Role of Tissue Factor in Cancer,Salvage Pathways of Pyrimidine Ribonucleotides,Semaphorin |

| | | | | | |
|---|---|---|---|---|---|
| | | | | | Signaling in Neurons,Sertoli Cell-Sertoli Cell Junction Signaling,Signaling by Rho Family GTPases,Sphingosine-1-phosphate Signaling,STAT3 Pathway,Synaptic Long Term Depression,Synaptic Long Term Potentiation,Systemic Lupus Erythematosus Signaling,T Cell Receptor Signaling,Telomerase Signaling,TGF-β Signaling,Thrombin Signaling,Thrombopoietin Signaling,Thyroid Cancer Signaling,Toll-like Receptor Signaling,TREM1 Signaling,Type I Diabetes Mellitus Signaling,Type II Diabetes Mellitus Signaling,UVA-Induced MAPK Signaling,UVB-Induced MAPK Signaling,UVC-Induced MAPK Signaling,VEGF Family Ligand-Receptor Interactions,VEGF Signaling,Xenobiotic Metabolism Signaling,α-Adrenergic Signaling |
| MIMAT0000070 | miR-17-5p (and other miRNAs w/seed AAAGUGC) | TargetScan Human | High (predicted) | MAPK4 | CDK5 Signaling,Endothelin-1 Signaling,HIF1α Signaling |
| MIMAT0000070 | miR-17-5p (and other miRNAs w/seed AAAGUGC) | TargetScan Human | High (predicted) | MAPK9 | 14-3-3-mediated Signaling,4-1BB Signaling in T Lymphocytes,Activation of IRF by Cytosolic Pattern Recognition Receptors,Acute Phase Response Signaling,Agrin Interactions at Neuromuscular Junction,Antioxidant Action of Vitamin C,April Mediated Signaling,ATM Signaling,B Cell Activating Factor Signaling,B Cell Receptor Signaling,BMP signaling pathway,Cardiac Hypertrophy Signaling,CCR5 Signaling in Macrophages,CD27 Signaling in Lymphocytes,CD28 Signaling in T Helper Cells,CD40 Signaling,Cdc42 Signaling,CDK5 Signaling,Cholecystokinin/Gastrin-mediated Signaling,Colorectal Cancer Metastasis Signaling,CXCR4 Signaling,Dendritic Cell Maturation,Endothelin-1 Signaling,ErbB Signaling,Fc Epsilon RI Signaling,FcγRIIB Signaling in B Lymphocytes,FXR/RXR Activation,GDNF Family Ligand-Receptor Interactions,Germ Cell-Sertoli Cell Junction Signaling,Glucocorticoid Receptor Signaling,GNRH Signaling,Gα12/13 Signaling,Hepatic Cholestasis,HGF Signaling,HIF1α Signaling,HMGB1 Signaling,Huntington's Disease Signaling,IL-1 Signaling,IL-12 Signaling and Production in Macrophages,IL-17 Signaling,IL-17A Signaling in Airway Cells,IL-17A Signaling in Gastric Cells,IL-22 Signaling,IL-6 Signaling,IL-8 Signaling,ILK Signaling,Induction of Apoptosis by HIV1,Inhibition of Angiogenesis by TSP1,Leukocyte Extravasation Signaling,LPS-stimulated MAPK Signaling,LPS/IL-1 Mediated Inhibition of RXR Function,MIF Regulation of Innate Immunity,Mitochondrial Dysfunction,Molecular Mechanisms of Cancer,Myc Mediated Apoptosis Signaling,NGF Signaling,NRF2-mediated Oxidative Stress Response,OX40 Signaling Pathway,PAK Signaling,Pancreatic Adenocarcinoma Signaling,Paxillin Signaling,PCP pathway,Production of Nitric |

| | | | | | |
|---|---|---|---|---|---|
| | | | | | Oxide and Reactive Oxygen Species in Macrophages,Pyridoxal 5'-phosphate Salvage Pathway,RANK Signaling in Osteoclasts,RAR Activation,Reelin Signaling in Neurons,Regulation of IL-2 Expression in Activated and Anergic T Lymphocytes,Renin-Angiotensin Signaling,Role of IL-17A in Arthritis,Role of JAK family kinases in IL-6-type Cytokine Signaling,Role of Macrophages, Fibroblasts and Endothelial Cells in Rheumatoid Arthritis,Role of MAPK Signaling in the Pathogenesis of Influenza,Role of NFAT in Cardiac Hypertrophy,Role of Osteoblasts, Osteoclasts and Chondrocytes in Rheumatoid Arthritis,Role of Pattern Recognition Receptors in Recognition of Bacteria and Viruses,Salvage Pathways of Pyrimidine Ribonucleotides,SAPK/JNK Signaling,Sertoli Cell-Sertoli Cell Junction Signaling,Signaling by Rho Family GTPases,STAT3 Pathway,Tec Kinase Signaling,TGF-β Signaling,Type I Diabetes Mellitus Signaling,Type II Diabetes Mellitus Signaling,UVA-Induced MAPK Signaling,UVB-Induced MAPK Signaling,UVC-Induced MAPK Signaling,Xenobiotic Metabolism Signaling |
| MIMAT0000070 | miR-17-5p (and other miRNAs w/seed AAAGUGC) | TargetScan Human | High (predicted) | MBTPS1 | Endoplasmic Reticulum Stress Pathway,Unfolded protein response |
| MIMAT0000070 | miR-17-5p (and other miRNAs w/seed AAAGUGC) | TargetScan Human | High (predicted) | MCL1 | Apoptosis Signaling,IL-6 Signaling,PI3K/AKT Signaling |
| MIMAT0000070 | miR-17-5p (and other miRNAs w/seed AAAGUGC) | miRecords | Experimentally Observed | MEF2D | Calcium Signaling,Calcium-induced T Lymphocyte Apoptosis,Cardiac Hypertrophy Signaling,Cholecystokinin/Gastrin-mediated Signaling,Corticotropin Releasing Hormone Signaling,ERK5 Signaling,Gα12/13 Signaling,Nur77 Signaling in T Lymphocytes,p38 MAPK Signaling,Phospholipase C Signaling,Role of NFAT in Cardiac Hypertrophy,Role of NFAT in Regulation of the Immune Response |
| MIMAT0000070 | miR-17-5p (and other miRNAs w/seed AAAGUGC) | TargetScan Human | High (predicted) | MKNK2 | ERK/MAPK Signaling,p38 MAPK Signaling |
| MIMAT0000070 | miR-17-5p (and other miRNAs w/seed | TargetScan Human | High (predicted) | MMP2 | Agranulocyte Adhesion and Diapedesis,Airway Pathology in Chronic Obstructive Pulmonary Disease,Axonal Guidance Signaling,Bladder Cancer Signaling,Colorectal Cancer Metastasis Signaling,Glioma Invasiveness |

| | | | | | |
|---|---|---|---|---|---|
| | AAAGUGC) | | | | Signaling,Granulocyte Adhesion and Diapedesis,Hepatic Fibrosis / Hepatic Stellate Cell Activation,HER-2 Signaling in Breast Cancer,HIF1α Signaling,IL-8 Signaling,Inhibition of Matrix Metalloproteases,Leukocyte Extravasation Signaling,Ovarian Cancer Signaling,Regulation of the Epithelial-Mesenchymal Transition Pathway |
| MIMAT0000070 | miR-17-5p (and other miRNAs w/seed AAAGUGC) | TargetScan Human | High (predicted) | MTMR3 | 3-phosphoinositide Degradation,Protein Kinase A Signaling |
| MIMAT0000070 | miR-17-5p (and other miRNAs w/seed AAAGUGC) | TargetScan Human | High (predicted) | MYCN | ERK/MAPK Signaling |
| MIMAT0000070 | miR-17-5p (and other miRNAs w/seed AAAGUGC) | TargetScan Human | High (predicted) | MYF5 | Transcriptional Regulatory Network in Embryonic Stem Cells |
| MIMAT0000070 | miR-17-5p (and other miRNAs w/seed AAAGUGC) | TargetScan Human | High (predicted) | NAPEPLD | Antioxidant Action of Vitamin C,Choline Biosynthesis III,Endothelin-1 Signaling,Fcγ Receptor-mediated Phagocytosis in Macrophages and Monocytes,Gαq Signaling,IL-8 Signaling,mTOR Signaling,Pancreatic Adenocarcinoma Signaling,Phospholipase C Signaling,Phospholipases |
| MIMAT0000070 | miR-17-5p (and other miRNAs w/seed AAAGUGC) | TarBase,TargetScan Human,miRecords | Experimentally Observed,High (predicted) | NCOA3 | Aryl Hydrocarbon Receptor Signaling,Estrogen Receptor Signaling,Glucocorticoid Receptor Signaling,PPARα/RXRα Activation,Role of Wnt/GSK-3β Signaling in the Pathogenesis of Influenza,TR/RXR Activation,VDR/RXR Activation |
| MIMAT0000070 | miR-17-5p (and other miRNAs w/seed AAAGUGC) | TargetScan Human | High (predicted) | NCOR1 | Estrogen Receptor Signaling,Glucocorticoid Receptor Signaling,Huntington's Disease Signaling,LXR/RXR Activation,PPAR Signaling,PPARα/RXRα Activation,RAR Activation,TR/RXR Activation,VDR/RXR Activation |
| MIMAT0000070 | miR-17-5p (and other miRNAs w/seed AAAGUGC) | TargetScan Human | High (predicted) | NDEL1 | Reelin Signaling in Neurons |

| | | | | | |
|---|---|---|---|---|---|
| MIMAT0000070 | miR-17-5p (and other miRNAs w/seed AAAGUGC) | TargetScan Human | High (predicted) | NEUROG1 | Transcriptional Regulatory Network in Embryonic Stem Cells |
| MIMAT0000070 | miR-17-5p (and other miRNAs w/seed AAAGUGC) | TargetScan Human | High (predicted) | NFAT5 | April Mediated Signaling,Axonal Guidance Signaling,B Cell Activating Factor Signaling,B Cell Receptor Signaling,Calcium Signaling,CD28 Signaling in T Helper Cells,fMLP Signaling in Neutrophils,Glucocorticoid Receptor Signaling,iCOS-iCOSL Signaling in T Helper Cells,IL-4 Signaling,Netrin Signaling,Phospholipase C Signaling,PI3K Signaling in B Lymphocytes,PKCθ Signaling in T Lymphocytes,Protein Kinase A Signaling,Regulation of IL-2 Expression in Activated and Anergic T Lymphocytes,Role of Macrophages, Fibroblasts and Endothelial Cells in Rheumatoid Arthritis,Role of NFAT in Regulation of the Immune Response,Role of Osteoblasts, Osteoclasts and Chondrocytes in Rheumatoid Arthritis,Systemic Lupus Erythematosus Signaling,T Cell Receptor Signaling,Wnt/Ca+ pathway |
| MIMAT0000070 | miR-17-5p (and other miRNAs w/seed AAAGUGC) | TargetScan Human | High (predicted) | NRP2 | Axonal Guidance Signaling,RhoA Signaling,VEGF Family Ligand-Receptor Interactions |
| MIMAT0000070 | miR-17-5p (and other miRNAs w/seed AAAGUGC) | TargetScan Human | High (predicted) | NTN1 | Axonal Guidance Signaling,Netrin Signaling,Protein Kinase A Signaling |
| MIMAT0000070 | miR-17-5p (and other miRNAs w/seed AAAGUGC) | TargetScan Human | High (predicted) | NTRK3 | Axonal Guidance Signaling,Human Embryonic Stem Cell Pluripotency,Neurotrophin/TRK Signaling,NF-κB Signaling,PTEN Signaling,STAT3 Pathway,Thyroid Cancer Signaling |
| MIMAT0000070 | miR-17-5p (and other miRNAs w/seed AAAGUGC) | TargetScan Human | High (predicted) | OCRL | 1D-myo-inositol Hexakisphosphate Biosynthesis II (Mammalian),3-phosphoinositide Degradation,B Cell Receptor Signaling,D-myo-inositol (1,3,4)-trisphosphate Biosynthesis,D-myo-inositol (1,4,5)-trisphosphate Degradation,Fc Epsilon RI Signaling,IL-4 Signaling,Insulin Receptor Signaling,Natural Killer Cell Signaling,PDGF Signaling,PI3K/AKT Signaling,PTEN Signaling,Superpathway of D-myo-inositol (1,4,5)-trisphosphate Metabolism,Superpathway of Inositol Phosphate Compounds |
| MIMAT0000070 | miR-17-5p (and | TargetScan Human | High (predicted) | P2RX4 | Gustation Pathway |

| | | | | | |
|---|---|---|---|---|---|
| | other miRNAs w/seed AAAGUGC) | | | | |
| MIMAT0000070 | miR-17-5p (and other miRNAs w/seed AAAGUGC) | TargetScan Human | High (predicted) | PAFAH1B1 | Reelin Signaling in Neurons |
| MIMAT0000070 | miR-17-5p (and other miRNAs w/seed AAAGUGC) | TargetScan Human | High (predicted) | PAFAH1B2 | Reelin Signaling in Neurons |
| MIMAT0000070 | miR-17-5p (and other miRNAs w/seed AAAGUGC) | TargetScan Human,miRecords | Experimentally Observed,High (predicted) | PAK7 | Actin Cytoskeleton Signaling,Agrin Interactions at Neuromuscular Junction,Angiopoietin Signaling,Axonal Guidance Signaling,CCR3 Signaling in Eosinophils,CXCR4 Signaling,Ephrin Receptor Signaling,ErbB Signaling,ERK/MAPK Signaling,FAK Signaling,Germ Cell-Sertoli Cell Junction Signaling,GNRH Signaling,Integrin Signaling,Molecular Mechanisms of Cancer,Natural Killer Cell Signaling,PAK Signaling,Paxillin Signaling,Pyridoxal 5'-phosphate Salvage Pathway,Rac Signaling,Regulation of Actin-based Motility by Rho,Renal Cell Carcinoma Signaling,Renin-Angiotensin Signaling,RhoGDI Signaling,Salvage Pathways of Pyrimidine Ribonucleotides,Semaphorin Signaling in Neurons,Signaling by Rho Family GTPases,Tec Kinase Signaling,TNFR1 Signaling |
| MIMAT0000070 | miR-17-5p (and other miRNAs w/seed AAAGUGC) | TargetScan Human | Moderate (predicted) | PDE1B | cAMP-mediated signaling,Cardiac β-adrenergic Signaling,Cellular Effects of Sildenafil (Viagra),G-Protein Coupled Receptor Signaling,Gustation Pathway,Nitric Oxide Signaling in the Cardiovascular System,Protein Kinase A Signaling,Relaxin Signaling,Sperm Motility,tRNA Splicing |
| MIMAT0000070 | miR-17-5p (and other miRNAs w/seed AAAGUGC) | TargetScan Human | High (predicted) | PDE3B | cAMP-mediated signaling,Cardiac β-adrenergic Signaling,Cellular Effects of Sildenafil (Viagra),G-Protein Coupled Receptor Signaling,Gustation Pathway,Insulin Receptor Signaling,Leptin Signaling in Obesity,Nitric Oxide Signaling in the Cardiovascular System,Protein Kinase A Signaling,Relaxin Signaling,TR/RXR Activation,tRNA Splicing |
| MIMAT0000070 | miR-17-5p (and other miRNAs w/seed AAAGUGC) | TargetScan Human | High (predicted) | PDGFRA | Glioblastoma Multiforme Signaling,Glioma Signaling,Hepatic Fibrosis / Hepatic Stellate Cell Activation,Human Embryonic Stem Cell Pluripotency,NF-κB Signaling,PAK Signaling,PDGF Signaling,PPAR Signaling,PTEN Signaling,Sphingosine-1-phosphate Signaling,STAT3 Pathway |

| | | | | | |
|---|---|---|---|---|---|
| MIMAT0000070 | miR-17-5p (and other miRNAs w/seed AAAGUGC) | TargetScan Human | High (predicted) | PFKFB3 | AMPK Signaling |
| MIMAT0000070 | miR-17-5p (and other miRNAs w/seed AAAGUGC) | TargetScan Human | High (predicted) | PFKP | AMPK Signaling,Glycolysis I,TR/RXR Activation |
| MIMAT0000070 | miR-17-5p (and other miRNAs w/seed AAAGUGC) | TargetScan Human | High (predicted) | PFN2 | Actin Cytoskeleton Signaling,Axonal Guidance Signaling,PCP pathway,Regulation of Actin-based Motility by Rho,RhoA Signaling |
| MIMAT0000070 | miR-17-5p (and other miRNAs w/seed AAAGUGC) | TargetScan Human | High (predicted) | PGP | Protein Kinase A Signaling |
| MIMAT0000070 | miR-17-5p (and other miRNAs w/seed AAAGUGC) | TargetScan Human | High (predicted) | PHC3 | Role of Oct4 in Mammalian Embryonic Stem Cell Pluripotency |
| MIMAT0000070 | miR-17-5p (and other miRNAs w/seed AAAGUGC) | TargetScan Human | High (predicted) | PIK3R1 | 14-3-3-mediated Signaling,3-phosphoinositide Biosynthesis,Actin Cytoskeleton Signaling,Acute Myeloid Leukemia Signaling,Acute Phase Response Signaling,Aldosterone Signaling in Epithelial Cells,AMPK Signaling,Amyotrophic Lateral Sclerosis Signaling,Angiopoietin Signaling,Antiproliferative Role of Somatostatin Receptor 2,Axonal Guidance Signaling,B Cell Receptor Signaling,Breast Cancer Regulation by Stathmin1,Cardiac Hypertrophy Signaling,CCR3 Signaling in Eosinophils,CD28 Signaling in T Helper Cells,CD40 Signaling,Ceramide Signaling,Chronic Myeloid Leukemia Signaling,Clathrin-mediated Endocytosis Signaling,CNTF Signaling,Colorectal Cancer Metastasis Signaling,CREB Signaling in Neurons,CTLA4 Signaling in Cytotoxic T Lymphocytes,CXCR4 Signaling,Dendritic Cell Maturation,Docosahexaenoic Acid (DHA) Signaling,EGF Signaling,EIF2 Signaling,Endometrial Cancer Signaling,Endothelin-1 Signaling,eNOS Signaling,Ephrin A Signaling,ErbB Signaling,ErbB2-ErbB3 Signaling,ErbB4 Signaling,ERK/MAPK Signaling,Erythropoietin Signaling,Estrogen-Dependent Breast Cancer |

| | | | | | |
|---|---|---|---|---|---|
| | | | | | Signaling,FAK Signaling,Fc Epsilon RI Signaling,Fcγ Receptor-mediated Phagocytosis in Macrophages and Monocytes,FcγRIIB Signaling in B Lymphocytes,FGF Signaling,FLT3 Signaling in Hematopoietic Progenitor Cells,fMLP Signaling in Neutrophils,G-Protein Coupled Receptor Signaling,Gap Junction Signaling,GDNF Family Ligand-Receptor Interactions,Germ Cell-Sertoli Cell Junction Signaling,Glioblastoma Multiforme Signaling,Glioma Invasiveness Signaling,Glioma Signaling,Glucocorticoid Receptor Signaling,GM-CSF Signaling,Growth Hormone Signaling,Gα12/13 Signaling,Gαq Signaling,HER-2 Signaling in Breast Cancer,Hereditary Breast Cancer Signaling,HGF Signaling,HIF1α Signaling,HMGB1 Signaling,Human Embryonic Stem Cell Pluripotency,Huntington's Disease Signaling,iCOS-iCOSL Signaling in T Helper Cells,IGF-1 Signaling,IL-12 Signaling and Production in Macrophages,IL-15 Signaling,IL-17 Signaling,IL-17A Signaling in Airway Cells,IL-2 Signaling,IL-3 Signaling,IL-4 Signaling,IL-6 Signaling,IL-8 Signaling,IL-9 Signaling,ILK Signaling,Insulin Receptor Signaling,Integrin Signaling,JAK/Stat Signaling,Leptin Signaling in Obesity,Leukocyte Extravasation Signaling,LPS-stimulated MAPK Signaling,Lymphotoxin β Receptor Signaling,Macropinocytosis Signaling,Melanocyte Development and Pigmentation Signaling,Melanoma Signaling,Molecular Mechanisms of Cancer,Mouse Embryonic Stem Cell Pluripotency,MSP-RON Signaling Pathway,mTOR Signaling,Myc Mediated Apoptosis Signaling,Natural Killer Cell Signaling,Neuregulin Signaling,Neuropathic Pain Signaling In Dorsal Horn Neurons,Neurotrophin/TRK Signaling,NF-κB Activation by Viruses,NF-κB Signaling,NGF Signaling,Nitric Oxide Signaling in the Cardiovascular System,Non-Small Cell Lung Cancer Signaling,NRF2-mediated Oxidative Stress Response,Ovarian Cancer Signaling,P2Y Purigenic Receptor Signaling Pathway,p53 Signaling,p70S6K Signaling,PAK Signaling,Pancreatic Adenocarcinoma Signaling,Paxillin Signaling,PDGF Signaling,PEDF Signaling,phagosome formation,PI3K Signaling in B Lymphocytes,PI3K/AKT Signaling,PKCθ Signaling in T Lymphocytes,Production of Nitric Oxide and Reactive Oxygen Species in Macrophages,Prolactin Signaling,Prostate Cancer Signaling,PTEN Signaling,Rac Signaling,RANK Signaling in Osteoclasts,RAR Activation,Reelin Signaling in Neurons,Regulation of eIF4 and p70S6K Signaling,Regulation of the Epithelial-Mesenchymal Transition Pathway,Relaxin Signaling,Renal Cell Carcinoma Signaling,Renin-Angiotensin Signaling,Role of IL-17A in Arthritis,Role of JAK1 and JAK3 in γc Cytokine Signaling,Role of Macrophages, Fibroblasts and Endothelial Cells in Rheumatoid Arthritis,Role of NANOG in Mammalian Embryonic Stem Cell Pluripotency,Role of NFAT in Cardiac Hypertrophy,Role of NFAT in Regulation of the Immune Response,Role of Osteoblasts, Osteoclasts and Chondrocytes in Rheumatoid Arthritis,Role of p14/p19ARF in Tumor Suppression,Role of |

| | | | | | |
|---|---|---|---|---|---|
| | | | | | Pattern Recognition Receptors in Recognition of Bacteria and Viruses,Role of PI3K/AKT Signaling in the Pathogenesis of Influenza,Role of Tissue Factor in Cancer,SAPK/JNK Signaling,Signaling by Rho Family GTPases,Small Cell Lung Cancer Signaling,Sphingosine-1-phosphate Signaling,Superpathway of Inositol Phosphate Compounds,Systemic Lupus Erythematosus Signaling,T Cell Receptor Signaling,Tec Kinase Signaling,Telomerase Signaling,Thrombin Signaling,Thrombopoietin Signaling,TR/RXR Activation,Type II Diabetes Mellitus Signaling,UVA-Induced MAPK Signaling,UVB-Induced MAPK Signaling,VEGF Family Ligand-Receptor Interactions,VEGF Signaling,Virus Entry via Endocytic Pathways,Xenobiotic Metabolism Signaling |
| MIMAT0000070 | miR-17-5p (and other miRNAs w/seed AAAGUGC) | TargetScan Human | High (predicted) | PIKFYVE | 3-phosphoinositide Biosynthesis,Actin Cytoskeleton Signaling,Aldosterone Signaling in Epithelial Cells,D-myo-inositol (1,4,5)-Trisphosphate Biosynthesis,phagosome maturation,Rac Signaling,Regulation of Actin-based Motility by Rho,RhoA Signaling,RhoGDI Signaling,Signaling by Rho Family GTPases,Superpathway of Inositol Phosphate Compounds |
| MIMAT0000070 | miR-17-5p (and other miRNAs w/seed AAAGUGC) | TargetScan Human | High (predicted) | PIP4K2A | 3-phosphoinositide Biosynthesis,Actin Cytoskeleton Signaling,Aldosterone Signaling in Epithelial Cells,D-myo-inositol (1,4,5)-Trisphosphate Biosynthesis,D-myo-inositol-5-phosphate Metabolism,Rac Signaling,Regulation of Actin-based Motility by Rho,RhoA Signaling,RhoGDI Signaling,Signaling by Rho Family GTPases,Superpathway of Inositol Phosphate Compounds |
| MIMAT0000070 | miR-17-5p (and other miRNAs w/seed AAAGUGC) | TargetScan Human | High (predicted) | PIP4K2C | 3-phosphoinositide Biosynthesis,Actin Cytoskeleton Signaling,Aldosterone Signaling in Epithelial Cells,D-myo-inositol (1,4,5)-Trisphosphate Biosynthesis,D-myo-inositol-5-phosphate Metabolism,Rac Signaling,Regulation of Actin-based Motility by Rho,RhoA Signaling,RhoGDI Signaling,Signaling by Rho Family GTPases,Superpathway of Inositol Phosphate Compounds |
| MIMAT0000070 | miR-17-5p (and other miRNAs w/seed AAAGUGC) | TargetScan Human | High (predicted) | PKIA | cAMP-mediated signaling,Cardiac β-adrenergic Signaling |
| MIMAT0000070 | miR-17-5p (and other miRNAs w/seed AAAGUGC) | TargetScan Human | High (predicted) | PLA2G6 | Antioxidant Action of Vitamin C,Atherosclerosis Signaling,CCR3 Signaling in Eosinophils,Eicosanoid Signaling,Endothelin-1 Signaling,ERK/MAPK Signaling,Fc Epsilon RI Signaling,Fcγ Receptor-mediated Phagocytosis in Macrophages and Monocytes,MIF Regulation of Innate Immunity,MIF-mediated Glucocorticoid Regulation,p38 MAPK Signaling,Phospholipase C |

| | | | | | |
|---|---|---|---|---|---|
| | | | | | Signaling,Phospholipases,Role of MAPK Signaling in the Pathogenesis of Influenza,Sperm Motility,Synaptic Long Term Depression,VEGF Family Ligand-Receptor Interactions |
| MIMAT0000070 | miR-17-5p (and other miRNAs w/seed AAAGUGC) | TargetScan Human | High (predicted) | PLCB1 | 14-3-3-mediated Signaling,Aldosterone Signaling in Epithelial Cells,Antioxidant Action of Vitamin C,Axonal Guidance Signaling,Breast Cancer Regulation by Stathmin1,Cardiac Hypertrophy Signaling,CCR3 Signaling in Eosinophils,Cellular Effects of Sildenafil (Viagra),Chemokine Signaling,Cholecystokinin/Gastrin-mediated Signaling,CREB Signaling in Neurons,CXCR4 Signaling,D-myo-inositol (1,4,5)-Trisphosphate Biosynthesis,D-myo-inositol-5-phosphate Metabolism,Dendritic Cell Maturation,Dopamine-DARPP32 Feedback in cAMP Signaling,Endothelin-1 Signaling,fMLP Signaling in Neutrophils,G Protein Signaling Mediated by Tubby,G-Protein Coupled Receptor Signaling,Gap Junction Signaling,Glioblastoma Multiforme Signaling,GNRH Signaling,GPCR-Mediated Integration of Enteroendocrine Signaling Exemplified by an L Cell,GPCR-Mediated Nutrient Sensing in Enteroendocrine Cells,Gαq Signaling,Huntington's Disease Signaling,Leptin Signaling in Obesity,Melatonin Signaling,Molecular Mechanisms of Cancer,Neuropathic Pain Signaling In Dorsal Horn Neurons,P2Y Purigenic Receptor Signaling Pathway,p70S6K Signaling,phagosome formation,Phospholipase C Signaling,Phospholipases,PI3K Signaling in B Lymphocytes,PPARα/RXRα Activation,Protein Kinase A Signaling,Role of Macrophages, Fibroblasts and Endothelial Cells in Rheumatoid Arthritis,Role of NFAT in Cardiac Hypertrophy,Role of NFAT in Regulation of the Immune Response,Role of Tissue Factor in Cancer,Sperm Motility,Sphingosine-1-phosphate Signaling,Superpathway of Inositol Phosphate Compounds,Synaptic Long Term Depression,Synaptic Long Term Potentiation,Thrombin Signaling,UVA-Induced MAPK Signaling,Wnt/Ca+ pathway |
| MIMAT0000070 | miR-17-5p (and other miRNAs w/seed AAAGUGC) | TargetScan Human | High (predicted) | PLXNA1 | Axonal Guidance Signaling,RhoA Signaling,Semaphorin Signaling in Neurons |
| MIMAT0000070 | miR-17-5p (and other miRNAs w/seed AAAGUGC) | TargetScan Human | High (predicted) | POU2F1 | Glucocorticoid Receptor Signaling,PKCθ Signaling in T Lymphocytes,Role of BRCA1 in DNA Damage Response |

| | | | | | |
|---|---|---|---|---|---|
| MIMAT0000070 | miR-17-5p (and other miRNAs w/seed AAAGUGC) | Ingenuity Expert Findings | Experimentally Observed | PPARG | Adipogenesis pathway,ERK/MAPK Signaling,FXR/RXR Activation,IL-12 Signaling and Production in Macrophages,PEDF Signaling,Polyamine Regulation in Colon Cancer,PPAR Signaling,Thyroid Cancer Signaling,Type II Diabetes Mellitus Signaling,Unfolded protein response |
| MIMAT0000070 | miR-17-5p (and other miRNAs w/seed AAAGUGC) | TargetScan Human | High (predicted) | PPM1A | AMPK Signaling |
| MIMAT0000070 | miR-17-5p (and other miRNAs w/seed AAAGUGC) | TargetScan Human | High (predicted) | PPP2CA | AMPK Signaling,Breast Cancer Regulation by Stathmin1,Cardiac β-adrenergic Signaling,CDK5 Signaling,Cell Cycle Regulation by BTG Family Proteins,Ceramide Signaling,CTLA4 Signaling in Cytotoxic T Lymphocytes,Cyclins and Cell Cycle Regulation,Dopamine Receptor Signaling,Dopamine-DARPP32 Feedback in cAMP Signaling,ERK/MAPK Signaling,HIPPO signaling,ILK Signaling,Mitotic Roles of Polo-Like Kinase,mTOR Signaling,p70S6K Signaling,PI3K/AKT Signaling,Production of Nitric Oxide and Reactive Oxygen Species in Macrophages,Regulation of eIF4 and p70S6K Signaling,Role of CHK Proteins in Cell Cycle Checkpoint Control,Synaptic Long Term Depression,Telomerase Signaling,Tight Junction Signaling,Wnt/β-catenin Signaling,Xenobiotic Metabolism Signaling |
| MIMAT0000070 | miR-17-5p (and other miRNAs w/seed AAAGUGC) | TargetScan Human | Moderate (predicted) | PPP2R1B | AMPK Signaling,Breast Cancer Regulation by Stathmin1,Cardiac β-adrenergic Signaling,CDK5 Signaling,Cell Cycle Regulation by BTG Family Proteins,Ceramide Signaling,CTLA4 Signaling in Cytotoxic T Lymphocytes,Cyclins and Cell Cycle Regulation,Dopamine Receptor Signaling,Dopamine-DARPP32 Feedback in cAMP Signaling,ERK/MAPK Signaling,HIPPO signaling,ILK Signaling,Mitotic Roles of Polo-Like Kinase,mTOR Signaling,p70S6K Signaling,PI3K/AKT Signaling,Production of Nitric Oxide and Reactive Oxygen Species in Macrophages,Regulation of eIF4 and p70S6K Signaling,Role of CHK Proteins in Cell Cycle Checkpoint Control,Synaptic Long Term Depression,Telomerase Signaling,Tight Junction Signaling,Wnt/β-catenin Signaling,Xenobiotic Metabolism Signaling |
| MIMAT0000070 | miR-17-5p (and other miRNAs w/seed AAAGUGC) | TargetScan Human | High (predicted) | PPP2R2A | AMPK Signaling,Breast Cancer Regulation by Stathmin1,Cardiac β-adrenergic Signaling,CDK5 Signaling,Cell Cycle Regulation by BTG Family Proteins,Ceramide Signaling,CTLA4 Signaling in Cytotoxic T Lymphocytes,Cyclins and Cell Cycle Regulation,Dopamine Receptor Signaling,Dopamine-DARPP32 Feedback in cAMP Signaling,ERK/MAPK Signaling,HIPPO signaling,ILK Signaling,Mitotic Roles of Polo-Like Kinase,mTOR Signaling,p70S6K Signaling,PI3K/AKT Signaling,Production of |

| | | | | | |
|---|---|---|---|---|---|
| | | | | | Nitric Oxide and Reactive Oxygen Species in Macrophages,Regulation of eIF4 and p70S6K Signaling,Role of CHK Proteins in Cell Cycle Checkpoint Control,Synaptic Long Term Depression,Telomerase Signaling,Tight Junction Signaling,Wnt/β-catenin Signaling,Xenobiotic Metabolism Signaling |
| MIMAT0000070 | miR-17-5p (and other miRNAs w/seed AAAGUGC) | TargetScan Human | High (predicted) | PPP2R3A | 3-phosphoinositide Biosynthesis,3-phosphoinositide Degradation,AMPK Signaling,Breast Cancer Regulation by Stathmin1,Cardiac β-adrenergic Signaling,CDK5 Signaling,Cell Cycle Regulation by BTG Family Proteins,Ceramide Signaling,CTLA4 Signaling in Cytotoxic T Lymphocytes,Cyclins and Cell Cycle Regulation,D-myo-inositol (1,4,5,6)-Tetrakisphosphate Biosynthesis,D-myo-inositol (3,4,5,6)-tetrakisphosphate Biosynthesis,D-myo-inositol-5-phosphate Metabolism,Dopamine Receptor Signaling,Dopamine-DARPP32 Feedback in cAMP Signaling,ERK/MAPK Signaling,HIPPO signaling,ILK Signaling,Mitotic Roles of Polo-Like Kinase,mTOR Signaling,p70S6K Signaling,PI3K/AKT Signaling,Production of Nitric Oxide and Reactive Oxygen Species in Macrophages,Regulation of eIF4 and p70S6K Signaling,Role of CHK Proteins in Cell Cycle Checkpoint Control,Superpathway of Inositol Phosphate Compounds,Synaptic Long Term Depression,Telomerase Signaling,Tight Junction Signaling,Wnt/β-catenin Signaling,Xenobiotic Metabolism Signaling |
| MIMAT0000070 | miR-17-5p (and other miRNAs w/seed AAAGUGC) | TargetScan Human | High (predicted) | PPP3R1 | Axonal Guidance Signaling,B Cell Receptor Signaling,Calcium Signaling,Calcium-induced T Lymphocyte Apoptosis,cAMP-mediated signaling,Cardiac Hypertrophy Signaling,CD28 Signaling in T Helper Cells,Clathrin-mediated Endocytosis Signaling,Dopamine-DARPP32 Feedback in cAMP Signaling,fMLP Signaling in Neutrophils,Gap Junction Signaling,Glucocorticoid Receptor Signaling,GM-CSF Signaling,Gαq Signaling,iCOS-iCOSL Signaling in T Helper Cells,IL-3 Signaling,Netrin Signaling,nNOS Signaling in Neurons,Nur77 Signaling in T Lymphocytes,Phospholipase C Signaling,PI3K Signaling in B Lymphocytes,PKCθ Signaling in T Lymphocytes,Protein Kinase A Signaling,RANK Signaling in Osteoclasts,Regulation of IL-2 Expression in Activated and Anergic T Lymphocytes,Role of Macrophages, Fibroblasts and Endothelial Cells in Rheumatoid Arthritis,Role of NFAT in Cardiac Hypertrophy,Role of NFAT in Regulation of the Immune Response,Role of Osteoblasts, Osteoclasts and Chondrocytes in Rheumatoid Arthritis,Synaptic Long Term Potentiation,T Cell Receptor Signaling |
| MIMAT0000070 | miR-17-5p (and other miRNAs w/seed | TargetScan Human | High (predicted) | PRKACB | AMPK Signaling,Amyloid Processing,Androgen Signaling,Axonal Guidance Signaling,BMP signaling pathway,Breast Cancer Regulation by Stathmin1,Calcium Signaling,cAMP-mediated signaling,Cardiac Hypertrophy |

| | | | | | |
|---|---|---|---|---|---|
| | AAAGUGC) | | | | Signaling,Cardiac β-adrenergic Signaling,CDK5 Signaling,Cellular Effects of Sildenafil (Viagra),Colorectal Cancer Metastasis Signaling,Corticotropin Releasing Hormone Signaling,CREB Signaling in Neurons,Dopamine Receptor Signaling,Dopamine-DARPP32 Feedback in cAMP Signaling,eNOS Signaling,ERK/MAPK Signaling,G Beta Gamma Signaling,G-Protein Coupled Receptor Signaling,Gap Junction Signaling,Glucocorticoid Receptor Signaling,GNRH Signaling,GPCR-Mediated Integration of Enteroendocrine Signaling Exemplified by an L Cell,GPCR-Mediated Nutrient Sensing in Enteroendocrine Cells,Gustation Pathway,Gαi Signaling,Gαs Signaling,Hepatic Cholestasis,IGF-1 Signaling,IL-1 Signaling,Insulin Receptor Signaling,Leptin Signaling in Obesity,Melanocyte Development and Pigmentation Signaling,Melatonin Signaling,Molecular Mechanisms of Cancer,Netrin Signaling,Neuropathic Pain Signaling In Dorsal Horn Neurons,Neuroprotective Role of THOP1 in Alzheimer's Disease,NF-κB Signaling,Nitric Oxide Signaling in the Cardiovascular System,Ovarian Cancer Signaling,P2Y Purigenic Receptor Signaling Pathway,Phototransduction Pathway,PPARα/RXRα Activation,Protein Kinase A Signaling,PXR/RXR Activation,RAR Activation,Relaxin Signaling,Renin-Angiotensin Signaling,Role of NFAT in Cardiac Hypertrophy,Sertoli Cell-Sertoli Cell Junction Signaling,Sonic Hedgehog Signaling,Sperm Motility,Synaptic Long Term Potentiation,Tight Junction Signaling,α-Adrenergic Signaling |
| MIMAT0000070 | miR-17-5p (and other miRNAs w/seed AAAGUGC) | TargetScan Human | High (predicted) | PSEN1 | Amyloid Processing,ErbB4 Signaling,Mitochondrial Dysfunction,Molecular Mechanisms of Cancer,Neuregulin Signaling,Notch Signaling,Regulation of the Epithelial-Mesenchymal Transition Pathway |
| MIMAT0000070 | miR-17-5p (and other miRNAs w/seed AAAGUGC) | Ingenuity Expert Findings,TargetScan Human | Experimentally Observed,High (predicted) | PTEN | 3-phosphoinositide Biosynthesis,3-phosphoinositide Degradation,B Cell Receptor Signaling,D-myo-inositol (1,3,4)-trisphosphate Biosynthesis,D-myo-inositol (1,4,5,6)-Tetrakisphosphate Biosynthesis,D-myo-inositol (3,4,5,6)-tetrakisphosphate Biosynthesis,D-myo-inositol-5-phosphate Metabolism,Endometrial Cancer Signaling,Epithelial Adherens Junction Signaling,ErbB2-ErbB3 Signaling,FAK Signaling,Fcγ Receptor-mediated Phagocytosis in Macrophages and Monocytes,Glioblastoma Multiforme Signaling,Glioma Signaling,Hereditary Breast Cancer Signaling,Hypoxia Signaling in the Cardiovascular System,iCOS-iCOSL Signaling in T Helper Cells,IL-17A Signaling in Airway Cells,ILK Signaling,Insulin Receptor Signaling,Integrin Signaling,Melanoma Signaling,Neuregulin Signaling,Ovarian Cancer Signaling,p53 Signaling,PI3K Signaling in B Lymphocytes,PI3K/AKT Signaling,Prostate Cancer Signaling,Protein Kinase A Signaling,PTEN Signaling,RAR Activation,Role of Tissue Factor in Cancer,Sertoli Cell-Sertoli |

| | | | | | |
|---|---|---|---|---|---|
| | | | | | Cell Junction Signaling,Small Cell Lung Cancer Signaling,Superpathway of D-myo-inositol (1,4,5)-trisphosphate Metabolism,Superpathway of Inositol Phosphate Compounds,Tight Junction Signaling |
| MIMAT0000070 | miR-17-5p (and other miRNAs w/seed AAAGUGC) | TargetScan Human | High (predicted) | PTGDR | cAMP-mediated signaling,Eicosanoid Signaling,G-Protein Coupled Receptor Signaling,Gαs Signaling |
| MIMAT0000070 | miR-17-5p (and other miRNAs w/seed AAAGUGC) | TargetScan Human | High (predicted) | PTGER3 | cAMP-mediated signaling,Colorectal Cancer Metastasis Signaling,Eicosanoid Signaling,G-Protein Coupled Receptor Signaling,Gαi Signaling |
| MIMAT0000070 | miR-17-5p (and other miRNAs w/seed AAAGUGC) | TargetScan Human | Moderate (predicted) | PTH | Dopamine Receptor Signaling,Role of Osteoblasts, Osteoclasts and Chondrocytes in Rheumatoid Arthritis,VDR/RXR Activation |
| MIMAT0000070 | miR-17-5p (and other miRNAs w/seed AAAGUGC) | TargetScan Human | High (predicted) | PTPDC1 | Protein Kinase A Signaling |
| MIMAT0000070 | miR-17-5p (and other miRNAs w/seed AAAGUGC) | TargetScan Human | High (predicted) | PTPN21 | Protein Kinase A Signaling |
| MIMAT0000070 | miR-17-5p (and other miRNAs w/seed AAAGUGC) | TargetScan Human | High (predicted) | PTPN3 | Protein Kinase A Signaling |
| MIMAT0000070 | miR-17-5p (and other miRNAs w/seed AAAGUGC) | TargetScan Human | High (predicted) | PTPN4 | Protein Kinase A Signaling |
| MIMAT0000070 | miR-17-5p (and other miRNAs w/seed | TargetScan Human | High (predicted) | PTPRD | Protein Kinase A Signaling |

| | | | | | |
|---|---|---|---|---|---|
| | AAAGUGC) | | | | |
| MIMAT0000070 | miR-17-5p (and other miRNAs w/seed AAAGUGC) | TargetScan Human | High (predicted) | PTPRJ | 3-phosphoinositide Biosynthesis,3-phosphoinositide Degradation,D-myo-inositol (1,4,5,6)-Tetrakisphosphate Biosynthesis,D-myo-inositol (3,4,5,6)-tetrakisphosphate Biosynthesis,D-myo-inositol-5-phosphate Metabolism,Protein Kinase A Signaling,Superpathway of Inositol Phosphate Compounds |
| MIMAT0000070 | miR-17-5p (and other miRNAs w/seed AAAGUGC) | TargetScan Human | High (predicted) | PTPRO | 3-phosphoinositide Biosynthesis,3-phosphoinositide Degradation,D-myo-inositol (1,4,5,6)-Tetrakisphosphate Biosynthesis,D-myo-inositol (3,4,5,6)-tetrakisphosphate Biosynthesis,D-myo-inositol-5-phosphate Metabolism,Protein Kinase A Signaling,Superpathway of Inositol Phosphate Compounds |
| MIMAT0000070 | miR-17-5p (and other miRNAs w/seed AAAGUGC) | TargetScan Human | Moderate (predicted) | PTPRT | Oleate Biosynthesis II (Animals),Protein Kinase A Signaling |
| MIMAT0000070 | miR-17-5p (and other miRNAs w/seed AAAGUGC) | TargetScan Human | High (predicted) | RAPGEF1 | Ephrin Receptor Signaling,Epithelial Adherens Junction Signaling,ERK/MAPK Signaling,HGF Signaling,IL-3 Signaling,Insulin Receptor Signaling,Integrin Signaling,Molecular Mechanisms of Cancer,Renal Cell Carcinoma Signaling |
| MIMAT0000070 | miR-17-5p (and other miRNAs w/seed AAAGUGC) | TargetScan Human | High (predicted) | RAPGEF2 | Gαs Signaling,RhoA Signaling |
| MIMAT0000070 | miR-17-5p (and other miRNAs w/seed AAAGUGC) | TargetScan Human | High (predicted) | RAPGEF4 | cAMP-mediated signaling,ERK/MAPK Signaling,G-Protein Coupled Receptor Signaling,GPCR-Mediated Nutrient Sensing in Enteroendocrine Cells,Gαs Signaling,Leukocyte Extravasation Signaling |
| MIMAT0000070 | miR-17-5p (and other miRNAs w/seed AAAGUGC) | TargetScan Human | High (predicted) | RASD1 | nNOS Signaling in Neurons |
| MIMAT0000070 | miR-17-5p (and other miRNAs w/seed | Ingenuity Expert Findings,TarBase,TargetSca | Experimentally Observed,High | RB1 | Antiproliferative Role of TOB in T Cell Signaling,Aryl Hydrocarbon Receptor Signaling,Bladder Cancer Signaling,Cell Cycle Regulation by BTG Family Proteins,Cell Cycle: G1/S Checkpoint Regulation,Chronic Myeloid Leukemia |

| | | | | | |
|---|---|---|---|---|---|
| | AAAGUGC) | n Human,miRecords | (predicted) | | Signaling,Cyclins and Cell Cycle Regulation,Estrogen-mediated S-phase Entry,Glioblastoma Multiforme Signaling,Glioma Signaling,Hereditary Breast Cancer Signaling,Melanoma Signaling,Molecular Mechanisms of Cancer,Non-Small Cell Lung Cancer Signaling,Ovarian Cancer Signaling,p53 Signaling,Pancreatic Adenocarcinoma Signaling,Prostate Cancer Signaling,Regulation of Cellular Mechanics by Calpain Protease,Role of BRCA1 in DNA Damage Response,Role of Oct4 in Mammalian Embryonic Stem Cell Pluripotency,Role of p14/p19ARF in Tumor Suppression,Small Cell Lung Cancer Signaling,Telomerase Signaling |
| MIMAT0000070 | miR-17-5p (and other miRNAs w/seed AAAGUGC) | TargetScan Human | High (predicted) | REST | Huntington's Disease Signaling,Role of Oct4 in Mammalian Embryonic Stem Cell Pluripotency,Transcriptional Regulatory Network in Embryonic Stem Cells |
| MIMAT0000070 | miR-17-5p (and other miRNAs w/seed AAAGUGC) | TargetScan Human | High (predicted) | RHO | Phototransduction Pathway,Protein Kinase A Signaling |
| MIMAT0000070 | miR-17-5p (and other miRNAs w/seed AAAGUGC) | TargetScan Human | High (predicted) | RHOC | Actin Nucleation by ARP-WASP Complex,Cardiac Hypertrophy Signaling,Cholecystokinin/Gastrin-mediated Signaling,Colorectal Cancer Metastasis Signaling,CXCR4 Signaling,Germ Cell-Sertoli Cell Junction Signaling,Glioblastoma Multiforme Signaling,Glioma Invasiveness Signaling,Gαq Signaling,HMGB1 Signaling,IL-8 Signaling,ILK Signaling,Integrin Signaling,Molecular Mechanisms of Cancer,mTOR Signaling,phagosome formation,Phospholipase C Signaling,Production of Nitric Oxide and Reactive Oxygen Species in Macrophages,Regulation of Actin-based Motility by Rho,RhoGDI Signaling,Semaphorin Signaling in Neurons,Signaling by Rho Family GTPases,Sphingosine-1-phosphate Signaling,Tec Kinase Signaling,Thrombin Signaling |
| MIMAT0000070 | miR-17-5p (and other miRNAs w/seed AAAGUGC) | TargetScan Human | High (predicted) | RND3 | Actin Nucleation by ARP-WASP Complex,Cardiac Hypertrophy Signaling,Cholecystokinin/Gastrin-mediated Signaling,Colorectal Cancer Metastasis Signaling,CXCR4 Signaling,Germ Cell-Sertoli Cell Junction Signaling,Glioblastoma Multiforme Signaling,Glioma Invasiveness Signaling,Gαq Signaling,HMGB1 Signaling,IL-8 Signaling,ILK Signaling,Integrin Signaling,Molecular Mechanisms of Cancer,mTOR Signaling,phagosome formation,Phospholipase C Signaling,Production of Nitric Oxide and Reactive Oxygen Species in Macrophages,Regulation of Actin-based Motility by Rho,RhoA Signaling,RhoGDI Signaling,Semaphorin Signaling in |

| | | | | | |
|---|---|---|---|---|---|
| | | | | | Neurons,Signaling by Rho Family GTPases,Sphingosine-1-phosphate Signaling,Tec Kinase Signaling,Thrombin Signaling |
| MIMAT0000070 | miR-17-5p (and other miRNAs w/seed AAAGUGC) | TargetScan Human | High (predicted) | RORA | Melatonin Signaling |
| MIMAT0000070 | miR-17-5p (and other miRNAs w/seed AAAGUGC) | TargetScan Human | High (predicted) | RORC | Melatonin Signaling,T Helper Cell Differentiation |
| MIMAT0000070 | miR-17-5p (and other miRNAs w/seed AAAGUGC) | TargetScan Human | High (predicted) | RPS6KA1 | 14-3-3-mediated Signaling,Adipogenesis pathway,Antiproliferative Role of TOB in T Cell Signaling,Apoptosis Signaling,cAMP-mediated signaling,Cardiac Hypertrophy Signaling,Cell Cycle: G2/M DNA Damage Checkpoint Regulation,CNTF Signaling,CREB Signaling in Neurons,ERK/MAPK Signaling,ERK5 Signaling,FLT3 Signaling in Hematopoietic Progenitor Cells,G-Protein Coupled Receptor Signaling,Growth Hormone Signaling,Melanocyte Development and Pigmentation Signaling,mTOR Signaling,Neurotrophin/TRK Signaling,NGF Signaling,p38 MAPK Signaling,Role of IL-17A in Arthritis,Role of IL-17F in Allergic Inflammatory Airway Diseases,Role of Tissue Factor in Cancer,Synaptic Long Term Potentiation,UVA-Induced MAPK Signaling |
| MIMAT0000070 | miR-17-5p (and other miRNAs w/seed AAAGUGC) | TargetScan Human | High (predicted) | RPS6KA2 | CNTF Signaling,ERK5 Signaling,FLT3 Signaling in Hematopoietic Progenitor Cells,Growth Hormone Signaling,Melanocyte Development and Pigmentation Signaling,MSP-RON Signaling Pathway,mTOR Signaling,NGF Signaling,p38 MAPK Signaling,Role of IL-17F in Allergic Inflammatory Airway Diseases,Role of Tissue Factor in Cancer,UVA-Induced MAPK Signaling |
| MIMAT0000070 | miR-17-5p (and other miRNAs w/seed AAAGUGC) | TargetScan Human | High (predicted) | RPS6KA3 | CNTF Signaling,ERK5 Signaling,FLT3 Signaling in Hematopoietic Progenitor Cells,Growth Hormone Signaling,Melanocyte Development and Pigmentation Signaling,mTOR Signaling,NGF Signaling,p38 MAPK Signaling,Phospholipase C Signaling,Role of IL-17F in Allergic Inflammatory Airway Diseases,Role of Tissue Factor in Cancer,UVA-Induced MAPK Signaling,UVB-Induced MAPK Signaling |
| MIMAT0000070 | miR-17-5p (and other miRNAs w/seed AAAGUGC) | TargetScan Human | High (predicted) | RPS6KA4 | CNTF Signaling,ERK/MAPK Signaling,ERK5 Signaling,FLT3 Signaling in Hematopoietic Progenitor Cells,Growth Hormone Signaling,ILK Signaling,Melanocyte Development and Pigmentation Signaling,mTOR Signaling,NGF Signaling,p38 MAPK Signaling,Role of IL-17F in Allergic |

| | | | | | |
|---|---|---|---|---|---|
| | | | | | Inflammatory Airway Diseases,Role of Tissue Factor in Cancer,UVA-Induced MAPK Signaling |
| MIMAT0000070 | miR-17-5p (and other miRNAs w/seed AAAGUGC) | TargetScan Human | High (predicted) | RPS6KA5 | Bladder Cancer Signaling,CNTF Signaling,ERK/MAPK Signaling,ERK5 Signaling,FGF Signaling,FLT3 Signaling in Hematopoietic Progenitor Cells,Growth Hormone Signaling,ILK Signaling,Melanocyte Development and Pigmentation Signaling,mTOR Signaling,NGF Signaling,p38 MAPK Signaling,Role of IL-17F in Allergic Inflammatory Airway Diseases,Role of Tissue Factor in Cancer,UVA-Induced MAPK Signaling,UVB-Induced MAPK Signaling |
| MIMAT0000070 | miR-17-5p (and other miRNAs w/seed AAAGUGC) | TargetScan Human | High (predicted) | RPS6KA6 | CNTF Signaling,ERK5 Signaling,FLT3 Signaling in Hematopoietic Progenitor Cells,Growth Hormone Signaling,Melanocyte Development and Pigmentation Signaling,mTOR Signaling,NGF Signaling,p38 MAPK Signaling,Role of IL-17F in Allergic Inflammatory Airway Diseases,Role of Tissue Factor in Cancer,UVA-Induced MAPK Signaling |
| MIMAT0000070 | miR-17-5p (and other miRNAs w/seed AAAGUGC) | TargetScan Human,miRecords | Experimentally Observed,High (predicted) | S1PR1 | cAMP-mediated signaling,Ceramide Signaling,G-Protein Coupled Receptor Signaling,Gαi Signaling,Human Embryonic Stem Cell Pluripotency,Sphingosine-1-phosphate Signaling |
| MIMAT0000070 | miR-17-5p (and other miRNAs w/seed AAAGUGC) | TargetScan Human | Moderate (predicted) | SDC2 | Axonal Guidance Signaling,Ephrin Receptor Signaling,Granulocyte Adhesion and Diapedesis,Inhibition of Angiogenesis by TSP1,Inhibition of Matrix Metalloproteases,PCP pathway |
| MIMAT0000070 | miR-17-5p (and other miRNAs w/seed AAAGUGC) | TargetScan Human | High (predicted) | SEMA4B | Axonal Guidance Signaling |
| MIMAT0000070 | miR-17-5p (and other miRNAs w/seed AAAGUGC) | TargetScan Human | High (predicted) | SEMA4G | Axonal Guidance Signaling |
| MIMAT0000070 | miR-17-5p (and other miRNAs w/seed AAAGUGC) | TargetScan Human | High (predicted) | SEMA5A | Axonal Guidance Signaling |

| | | | | | |
|---|---|---|---|---|---|
| MIMAT0000070 | miR-17-5p (and other miRNAs w/seed AAAGUGC) | TargetScan Human | High (predicted) | SEMA7A | Axonal Guidance Signaling,Semaphorin Signaling in Neurons |
| MIMAT0000070 | miR-17-5p (and other miRNAs w/seed AAAGUGC) | TargetScan Human | High (predicted) | SHANK2 | Axonal Guidance Signaling |
| MIMAT0000070 | miR-17-5p (and other miRNAs w/seed AAAGUGC) | TargetScan Human | High (predicted) | SIRPA | 3-phosphoinositide Biosynthesis,3-phosphoinositide Degradation,D-myo-inositol (1,4,5,6)-Tetrakisphosphate Biosynthesis,D-myo-inositol (3,4,5,6)-tetrakisphosphate Biosynthesis,D-myo-inositol-5-phosphate Metabolism,Production of Nitric Oxide and Reactive Oxygen Species in Macrophages,Protein Kinase A Signaling,Role of JAK2 in Hormone-like Cytokine Signaling,Superpathway of Inositol Phosphate Compounds |
| MIMAT0000070 | miR-17-5p (and other miRNAs w/seed AAAGUGC) | TargetScan Human | High (predicted) | SLC17A7 | Glutamate Receptor Signaling |
| MIMAT0000070 | miR-17-5p (and other miRNAs w/seed AAAGUGC) | TargetScan Human | High (predicted) | SLC1A2 | Amyotrophic Lateral Sclerosis Signaling,Glutamate Receptor Signaling |
| MIMAT0000070 | miR-17-5p (and other miRNAs w/seed AAAGUGC) | TargetScan Human | High (predicted) | SLC2A4 | Adipogenesis pathway,AMPK Signaling,Antioxidant Action of Vitamin C,Growth Hormone Signaling,HIF1α Signaling,Hypoxia Signaling in the Cardiovascular System,Insulin Receptor Signaling,Melatonin Signaling,Type II Diabetes Mellitus Signaling,Vitamin-C Transport |
| MIMAT0000070 | miR-17-5p (and other miRNAs w/seed AAAGUGC) | TargetScan Human | High (predicted) | SMAD4 | Antiproliferative Role of TOB in T Cell Signaling,BMP signaling pathway,Cardiomyocyte Differentiation via BMP Receptors,Cell Cycle: G1/S Checkpoint Regulation,Chronic Myeloid Leukemia Signaling,Colorectal Cancer Metastasis Signaling,Factors Promoting Cardiogenesis in Vertebrates,Glucocorticoid Receptor Signaling,Hepatic Fibrosis / Hepatic Stellate Cell Activation,HIPPO signaling,Human Embryonic Stem Cell Pluripotency,Molecular Mechanisms of Cancer,Mouse Embryonic Stem Cell Pluripotency,Pancreatic Adenocarcinoma Signaling,PPARα/RXRα Activation,Protein Kinase A Signaling,RAR Activation,Regulation of IL-2 |

| | | | | | |
|---|---|---|---|---|---|
| | | | | | Expression in Activated and Anergic T Lymphocytes,Regulation of the Epithelial-Mesenchymal Transition Pathway,Role of NANOG in Mammalian Embryonic Stem Cell Pluripotency,Role of Osteoblasts, Osteoclasts and Chondrocytes in Rheumatoid Arthritis,TGF-β Signaling |
| MIMAT0000070 | miR-17-5p (and other miRNAs w/seed AAAGUGC) | TargetScan Human | High (predicted) | SMAD5 | Adipogenesis pathway,BMP signaling pathway,Factors Promoting Cardiogenesis in Vertebrates,HIPPO signaling,Human Embryonic Stem Cell Pluripotency,Molecular Mechanisms of Cancer,Mouse Embryonic Stem Cell Pluripotency,RAR Activation,Role of NANOG in Mammalian Embryonic Stem Cell Pluripotency,Role of Osteoblasts, Osteoclasts and Chondrocytes in Rheumatoid Arthritis,TGF-β Signaling |
| MIMAT0000070 | miR-17-5p (and other miRNAs w/seed AAAGUGC) | TargetScan Human | High (predicted) | SOCS6 | Acute Phase Response Signaling,Growth Hormone Signaling,IGF-1 Signaling,JAK/Stat Signaling,Prolactin Signaling,Role of JAK2 in Hormone-like Cytokine Signaling,STAT3 Pathway,Type I Diabetes Mellitus Signaling,Type II Diabetes Mellitus Signaling |
| MIMAT0000070 | miR-17-5p (and other miRNAs w/seed AAAGUGC) | TargetScan Human | High (predicted) | SOCS7 | Acute Phase Response Signaling,Growth Hormone Signaling,IGF-1 Signaling,JAK/Stat Signaling,Prolactin Signaling,Role of JAK2 in Hormone-like Cytokine Signaling,STAT3 Pathway,Type I Diabetes Mellitus Signaling,Type II Diabetes Mellitus Signaling |
| MIMAT0000070 | miR-17-5p (and other miRNAs w/seed AAAGUGC) | TargetScan Human | High (predicted) | SOS1 | Actin Cytoskeleton Signaling,Actin Nucleation by ARP-WASP Complex,Acute Myeloid Leukemia Signaling,Acute Phase Response Signaling,Aldosterone Signaling in Epithelial Cells,Angiopoietin Signaling,Axonal Guidance Signaling,B Cell Receptor Signaling,BMP signaling pathway,Breast Cancer Regulation by Stathmin1,Cardiac Hypertrophy Signaling,Cholecystokinin/Gastrin-mediated Signaling,Chronic Myeloid Leukemia Signaling,CNTF Signaling,Colorectal Cancer Metastasis Signaling,CREB Signaling in Neurons,EGF Signaling,EIF2 Signaling,Endometrial Cancer Signaling,Endothelin-1 Signaling,Ephrin Receptor Signaling,ErbB Signaling,ErbB2-ErbB3 Signaling,ErbB4 Signaling,ERK/MAPK Signaling,Erythropoietin Signaling,Estrogen Receptor Signaling,FAK Signaling,Fc Epsilon RI Signaling,FcγRIIB Signaling in B Lymphocytes,FGF Signaling,FLT3 Signaling in Hematopoietic Progenitor Cells,G Beta Gamma Signaling,G-Protein Coupled Receptor Signaling,Gap Junction Signaling,GDNF Family Ligand-Receptor Interactions,Glioblastoma Multiforme Signaling,Glioma Signaling,Glucocorticoid Receptor Signaling,GM-CSF Signaling,GNRH Signaling,Gαi Signaling,HER-2 Signaling in Breast Cancer,HGF Signaling,Huntington's Disease Signaling,IGF-1 Signaling,IL-2 Signaling,IL-3 Signaling,IL-4 Signaling,IL-6 Signaling,Insulin Receptor Signaling,Integrin |

| | | | | | |
|---|---|---|---|---|---|
| | | | | | Signaling,JAK/Stat Signaling,Melanocyte Development and Pigmentation Signaling,Molecular Mechanisms of Cancer,Mouse Embryonic Stem Cell Pluripotency,Myc Mediated Apoptosis Signaling,Natural Killer Cell Signaling,Neuregulin Signaling,Neurotrophin/TRK Signaling,NGF Signaling,Non-Small Cell Lung Cancer Signaling,Oncostatin M Signaling,p70S6K Signaling,PAK Signaling,Paxillin Signaling,PDGF Signaling,Phospholipase C Signaling,PI3K/AKT Signaling,PKCθ Signaling in T Lymphocytes,PPAR Signaling,PPARα/RXRα Activation,Prolactin Signaling,Prostate Cancer Signaling,PTEN Signaling,Regulation of eIF4 and p70S6K Signaling,Regulation of IL-2 Expression in Activated and Anergic T Lymphocytes,Regulation of the Epithelial-Mesenchymal Transition Pathway,Renal Cell Carcinoma Signaling,Renin-Angiotensin Signaling,Role of NANOG in Mammalian Embryonic Stem Cell Pluripotency,Role of NFAT in Cardiac Hypertrophy,Role of NFAT in Regulation of the Immune Response,SAPK/JNK Signaling,Systemic Lupus Erythematosus Signaling,T Cell Receptor Signaling,Telomerase Signaling,TGF-β Signaling,Thrombin Signaling,Thrombopoietin Signaling,VEGF Family Ligand-Receptor Interactions,VEGF Signaling |
| MIMAT0000070 | miR-17-5p (and other miRNAs w/seed AAAGUGC) | TargetScan Human | High (predicted) | SRGAP1 | Axonal Guidance Signaling |
| MIMAT0000070 | miR-17-5p (and other miRNAs w/seed AAAGUGC) | TargetScan Human | High (predicted) | SRGAP3 | Axonal Guidance Signaling |
| MIMAT0000070 | miR-17-5p (and other miRNAs w/seed AAAGUGC) | Ingenuity Expert Findings,TargetScan Human,miRecords | Experimentally Observed,High (predicted) | STAT3 | Acute Myeloid Leukemia Signaling,Acute Phase Response Signaling,cAMP-mediated signaling,CD40 Signaling,CNTF Signaling,Colorectal Cancer Metastasis Signaling,EGF Signaling,Ephrin Receptor Signaling,ErbB2-ErbB3 Signaling,ERK/MAPK Signaling,FGF Signaling,FLT3 Signaling in Hematopoietic Progenitor Cells,G-Protein Coupled Receptor Signaling,Glucocorticoid Receptor Signaling,GM-CSF Signaling,Growth Hormone Signaling,Gαi Signaling,HGF Signaling,IGF-1 Signaling,IL-10 Signaling,IL-15 Signaling,IL-17A Signaling in Airway Cells,IL-22 Signaling,IL-3 Signaling,IL-6 Signaling,IL-9 Signaling,JAK/Stat Signaling,Leptin Signaling in Obesity,Mouse Embryonic Stem Cell Pluripotency,Oncostatin M Signaling,Pancreatic Adenocarcinoma Signaling,PDGF Signaling,Prolactin Signaling,Regulation of the Epithelial-Mesenchymal Transition Pathway,Renin-Angiotensin Signaling,Role of JAK |

| | | | | | |
|---|---|---|---|---|---|
| | | | | | family kinases in IL-6-type Cytokine Signaling,Role of JAK1 and JAK3 in γc Cytokine Signaling,Role of JAK1, JAK2 and TYK2 in Interferon Signaling,Role of JAK2 in Hormone-like Cytokine Signaling,Role of Macrophages, Fibroblasts and Endothelial Cells in Rheumatoid Arthritis,Role of NANOG in Mammalian Embryonic Stem Cell Pluripotency,STAT3 Pathway,T Helper Cell Differentiation,Tec Kinase Signaling,Thrombopoietin Signaling,Transcriptional Regulatory Network in Embryonic Stem Cells,TREM1 Signaling |
| MIMAT0000070 | miR-17-5p (and other miRNAs w/seed AAAGUGC) | TargetScan Human | High (predicted) | STK11 | 14-3-3-mediated Signaling,AMPK Signaling,mTOR Signaling |
| MIMAT0000070 | miR-17-5p (and other miRNAs w/seed AAAGUGC) | TargetScan Human | High (predicted) | TAOK3 | Endoplasmic Reticulum Stress Pathway |
| MIMAT0000070 | miR-17-5p (and other miRNAs w/seed AAAGUGC) | TargetScan Human | High (predicted) | TCF4 | Acute Myeloid Leukemia Signaling,Acute Phase Response Signaling,Basal Cell Carcinoma Signaling,Colorectal Cancer Metastasis Signaling,Epithelial Adherens Junction Signaling,Factors Promoting Cardiogenesis in Vertebrates,Human Embryonic Stem Cell Pluripotency,Molecular Mechanisms of Cancer,Mouse Embryonic Stem Cell Pluripotency,Ovarian Cancer Signaling,PEDF Signaling,Polyamine Regulation in Colon Cancer,Protein Kinase A Signaling,Regulation of the Epithelial-Mesenchymal Transition Pathway,Role of Macrophages, Fibroblasts and Endothelial Cells in Rheumatoid Arthritis,Role of Osteoblasts, Osteoclasts and Chondrocytes in Rheumatoid Arthritis,Role of Wnt/GSK-3β Signaling in the Pathogenesis of Influenza,Thyroid Cancer Signaling,Wnt/β-catenin Signaling |
| MIMAT0000070 | miR-17-5p (and other miRNAs w/seed AAAGUGC) | TargetScan Human | High (predicted) | TCF7L1 | Acute Myeloid Leukemia Signaling,Basal Cell Carcinoma Signaling,Colorectal Cancer Metastasis Signaling,Epithelial Adherens Junction Signaling,Factors Promoting Cardiogenesis in Vertebrates,Human Embryonic Stem Cell Pluripotency,Mouse Embryonic Stem Cell Pluripotency,Ovarian Cancer Signaling,Protein Kinase A Signaling,Regulation of the Epithelial-Mesenchymal Transition Pathway,Role of Macrophages, Fibroblasts and Endothelial Cells in Rheumatoid Arthritis,Role of NANOG in Mammalian Embryonic Stem Cell Pluripotency,Role of Osteoblasts, Osteoclasts and Chondrocytes in Rheumatoid Arthritis,Role of Wnt/GSK-3β Signaling in the Pathogenesis of Influenza,Thyroid Cancer Signaling,Transcriptional Regulatory Network in |

| | | | | | |
|---|---|---|---|---|---|
| | | | | | Embryonic Stem Cells,Wnt/β-catenin Signaling |
| MIMAT0000070 | miR-17-5p (and other miRNAs w/seed AAAGUGC) | Ingenuity Expert Findings,TarBase,TargetScan Human | Experimentally Observed,High (predicted) | TGFBR2 | Antiproliferative Role of TOB in T Cell Signaling,Cardiac Hypertrophy Signaling,Chronic Myeloid Leukemia Signaling,Colorectal Cancer Metastasis Signaling,Epithelial Adherens Junction Signaling,Factors Promoting Cardiogenesis in Vertebrates,Germ Cell-Sertoli Cell Junction Signaling,Glucocorticoid Receptor Signaling,Hepatic Fibrosis / Hepatic Stellate Cell Activation,Human Embryonic Stem Cell Pluripotency,Inhibition of Angiogenesis by TSP1,Molecular Mechanisms of Cancer,NF-κB Signaling,p38 MAPK Signaling,Pancreatic Adenocarcinoma Signaling,PPARα/RXRα Activation,Protein Kinase A Signaling,PTEN Signaling,Regulation of IL-2 Expression in Activated and Anergic T Lymphocytes,Regulation of the Epithelial-Mesenchymal Transition Pathway,Role of NFAT in Cardiac Hypertrophy,STAT3 Pathway,T Helper Cell Differentiation,TGF-β Signaling,Tight Junction Signaling,Wnt/β-catenin Signaling |
| MIMAT0000070 | miR-17-5p (and other miRNAs w/seed AAAGUGC) | TargetScan Human | Moderate (predicted) | TGM2 | Aryl Hydrocarbon Receptor Signaling,Huntington's Disease Signaling,Phospholipase C Signaling |
| MIMAT0000070 | miR-17-5p (and other miRNAs w/seed AAAGUGC) | Ingenuity Expert Findings | Experimentally Observed | TP63 | Calcium Signaling,p53 Signaling |
| MIMAT0000070 | miR-17-5p (and other miRNAs w/seed AAAGUGC) | TargetScan Human | Moderate (predicted) | TRAF4 | Lymphotoxin β Receptor Signaling,NGF Signaling,Role of Macrophages, Fibroblasts and Endothelial Cells in Rheumatoid Arthritis,Small Cell Lung Cancer Signaling,Toll-like Receptor Signaling |
| MIMAT0000070 | miR-17-5p (and other miRNAs w/seed AAAGUGC) | TargetScan Human | Moderate (predicted) | TRDN | Calcium Signaling |
| MIMAT0000070 | miR-17-5p (and other miRNAs w/seed AAAGUGC) | TargetScan Human | High (predicted) | TRPV6 | Calcium Signaling,VDR/RXR Activation |

| | | | | | |
|---|---|---|---|---|---|
| MIMAT0000070 | miR-17-5p (and other miRNAs w/seed AAAGUGC) | TargetScan Human | High (predicted) | TSG101 | Breast Cancer Regulation by Stathmin1,Clathrin-mediated Endocytosis Signaling,Glucocorticoid Receptor Signaling,Mechanisms of Viral Exit from Host Cells,phagosome maturation |
| MIMAT0000070 | miR-17-5p (and other miRNAs w/seed AAAGUGC) | TargetScan Human | High (predicted) | UBASH3B | Protein Kinase A Signaling |
| MIMAT0000070 | miR-17-5p (and other miRNAs w/seed AAAGUGC) | TargetScan Human | High (predicted) | UBC | Clathrin-mediated Endocytosis Signaling,GABA Receptor Signaling,Hereditary Breast Cancer Signaling,Huntington's Disease Signaling,Protein Ubiquitination Pathway,Renal Cell Carcinoma Signaling,Toll-like Receptor Signaling,Wnt/β-catenin Signaling |
| MIMAT0000070 | miR-17-5p (and other miRNAs w/seed AAAGUGC) | TargetScan Human | High (predicted) | ULK1 | AMPK Signaling,autophagy,mTOR Signaling |
| MIMAT0000070 | miR-17-5p (and other miRNAs w/seed AAAGUGC) | TargetScan Human | High (predicted) | VASP | Actin Nucleation by ARP-WASP Complex,Axonal Guidance Signaling,Fcγ Receptor-mediated Phagocytosis in Macrophages and Monocytes,IL-8 Signaling,Integrin Signaling,Leukocyte Extravasation Signaling,Protein Kinase A Signaling,Tight Junction Signaling |
| MIMAT0000070 | miR-17-5p (and other miRNAs w/seed AAAGUGC) | TargetScan Human | High (predicted) | VAV2 | Actin Cytoskeleton Signaling,B Cell Receptor Signaling,Cdc42 Signaling,Ephrin A Signaling,Ephrin B Signaling,Epithelial Adherens Junction Signaling,Fc Epsilon RI Signaling,Fcγ Receptor-mediated Phagocytosis in Macrophages and Monocytes,Gα12/13 Signaling,Leukocyte Extravasation Signaling,Natural Killer Cell Signaling,PI3K Signaling in B Lymphocytes,PKCθ Signaling in T Lymphocytes,Regulation of IL-2 Expression in Activated and Anergic T Lymphocytes,T Cell Receptor Signaling,Tec Kinase Signaling |
| MIMAT0000070 | miR-17-5p (and other miRNAs w/seed AAAGUGC) | TargetScan Human | High (predicted) | VCL | Actin Cytoskeleton Signaling,Epithelial Adherens Junction Signaling,FAK Signaling,Germ Cell-Sertoli Cell Junction Signaling,ILK Signaling,Integrin Signaling,Leukocyte Extravasation Signaling,Paxillin Signaling,Regulation of Cellular Mechanics by Calpain Protease,Remodeling of Epithelial Adherens Junctions,Sertoli Cell-Sertoli Cell Junction Signaling,Tight Junction Signaling,VEGF Signaling |
| MIMAT0000070 | miR-17-5p (and other miRNAs | TargetScan | Experimentally Observed,High | VEGFA | Amyotrophic Lateral Sclerosis Signaling,Axonal Guidance Signaling,Bladder Cancer Signaling,Clathrin-mediated Endocytosis Signaling,Colorectal Cancer |

| | | | | | |
|---|---|---|---|---|---|
| | w/seed AAAGUGC) | Human,miRecords | (predicted) | | Metastasis Signaling,Corticotropin Releasing Hormone Signaling,eNOS Signaling,Ephrin Receptor Signaling,Hepatic Fibrosis / Hepatic Stellate Cell Activation,HIF1α Signaling,Hypoxia Signaling in the Cardiovascular System,IL-6 Signaling,IL-8 Signaling,ILK Signaling,Inhibition of Angiogenesis by TSP1,mTOR Signaling,Nitric Oxide Signaling in the Cardiovascular System,Ovarian Cancer Signaling,Pancreatic Adenocarcinoma Signaling,RAR Activation,Relaxin Signaling,Renal Cell Carcinoma Signaling,Role of Macrophages, Fibroblasts and Endothelial Cells in Rheumatoid Arthritis,Role of Tissue Factor in Cancer,VEGF Family Ligand-Receptor Interactions,VEGF Signaling |
| MIMAT0000070 | miR-17-5p (and other miRNAs w/seed AAAGUGC) | TargetScan Human | High (predicted) | VLDLR | FXR/RXR Activation,Reelin Signaling in Neurons |
| MIMAT0000070 | miR-17-5p (and other miRNAs w/seed AAAGUGC) | TargetScan Human | High (predicted) | YES1 | Epithelial Adherens Junction Signaling,Fcγ Receptor-mediated Phagocytosis in Macrophages and Monocytes,Reelin Signaling in Neurons,Role of Tissue Factor in Cancer,Tec Kinase Signaling |
| MIMAT0000751 | miR-330-3p (and other miRNAs w/seed CAAAGCA) | TargetScan Human | Moderate (predicted) | ACTC1 | Actin Cytoskeleton Signaling,Agranulocyte Adhesion and Diapedesis,Agrin Interactions at Neuromuscular Junction,Calcium Signaling,Caveolar-mediated Endocytosis Signaling,Cellular Effects of Sildenafil (Viagra),Clathrin-mediated Endocytosis Signaling,Crosstalk between Dendritic Cells and Natural Killer Cells,Death Receptor Signaling,Epithelial Adherens Junction Signaling,FAK Signaling,Fcγ Receptor-mediated Phagocytosis in Macrophages and Monocytes,Gap Junction Signaling,Germ Cell-Sertoli Cell Junction Signaling,ILK Signaling,Integrin Signaling,Leukocyte Extravasation Signaling,Mechanisms of Viral Exit from Host Cells,MSP-RON Signaling Pathway,NRF2-mediated Oxidative Stress Response,Paxillin Signaling,Regulation of Actin-based Motility by Rho,Remodeling of Epithelial Adherens Junctions,RhoA Signaling,RhoGDI Signaling,Sertoli Cell-Sertoli Cell Junction Signaling,Signaling by Rho Family GTPases,Tec Kinase Signaling,Tight Junction Signaling,VEGF Signaling,Virus Entry via Endocytic Pathways |
| MIMAT0000751 | miR-330-3p (and other miRNAs w/seed CAAAGCA) | TargetScan Human | Moderate (predicted) | ACTG1 | Actin Cytoskeleton Signaling,Agranulocyte Adhesion and Diapedesis,Agrin Interactions at Neuromuscular Junction,Caveolar-mediated Endocytosis Signaling,Cellular Effects of Sildenafil (Viagra),Clathrin-mediated Endocytosis Signaling,Crosstalk between Dendritic Cells and Natural Killer Cells,Death Receptor Signaling,Epithelial Adherens Junction Signaling,FAK Signaling,Fcγ |

| | | | | | |
|---|---|---|---|---|---|
| | | | | | Receptor-mediated Phagocytosis in Macrophages and Monocytes,Gap Junction Signaling,Germ Cell-Sertoli Cell Junction Signaling,ILK Signaling,Integrin Signaling,Leukocyte Extravasation Signaling,Mechanisms of Viral Exit from Host Cells,MSP-RON Signaling Pathway,NRF2-mediated Oxidative Stress Response,Paxillin Signaling,Remodeling of Epithelial Adherens Junctions,RhoA Signaling,RhoGDI Signaling,Sertoli Cell-Sertoli Cell Junction Signaling,Signaling by Rho Family GTPases,Tec Kinase Signaling,Tight Junction Signaling,VEGF Signaling,Virus Entry via Endocytic Pathways |
| MIMAT0000751 | miR-330-3p (and other miRNAs w/seed CAAAGCA) | TargetScan Human | Moderate (predicted) | ADAM10 | Axonal Guidance Signaling,Ephrin A Signaling,Ephrin Receptor Signaling,Inhibition of Matrix Metalloproteases |
| MIMAT0000751 | miR-330-3p (and other miRNAs w/seed CAAAGCA) | TargetScan Human | Moderate (predicted) | ADAMTS5 | Axonal Guidance Signaling,Role of Osteoblasts, Osteoclasts and Chondrocytes in Rheumatoid Arthritis |
| MIMAT0000751 | miR-330-3p (and other miRNAs w/seed CAAAGCA) | TargetScan Human | Moderate (predicted) | ADAMTS9 | Axonal Guidance Signaling |
| MIMAT0000751 | miR-330-3p (and other miRNAs w/seed CAAAGCA) | TargetScan Human | Moderate (predicted) | ADD3 | Gαs Signaling,Protein Kinase A Signaling |
| MIMAT0000751 | miR-330-3p (and other miRNAs w/seed CAAAGCA) | TargetScan Human | High (predicted) | AGTR2 | cAMP-mediated signaling,G-Protein Coupled Receptor Signaling,Gαi Signaling,Renin-Angiotensin Signaling |
| MIMAT0000751 | miR-330-3p (and other miRNAs w/seed CAAAGCA) | TargetScan Human | Moderate (predicted) | AK7 | AMPK Signaling,Pyrimidine Deoxyribonucleotides De Novo Biosynthesis I,Pyrimidine Ribonucleotides De Novo Biosynthesis,Pyrimidine Ribonucleotides Interconversion,Salvage Pathways of Pyrimidine Ribonucleotides |
| MIMAT0000751 | miR-330-3p (and other miRNAs w/seed | TargetScan Human | Moderate (predicted) | ASIC2 | Aldosterone Signaling in Epithelial Cells,Gustation Pathway,Insulin Receptor Signaling |

| | | | | | |
|---|---|---|---|---|---|
| | CAAAGCA) | | | | |
| MIMAT0000751 | miR-330-3p (and other miRNAs w/seed CAAAGCA) | TargetScan Human | High (predicted) | ATP2C1 | Calcium Signaling,Calcium Transport I |
| MIMAT0000751 | miR-330-3p (and other miRNAs w/seed CAAAGCA) | TargetScan Human | High (predicted) | BMI1 | Cell Cycle: G1/S Checkpoint Regulation,Role of Oct4 in Mammalian Embryonic Stem Cell Pluripotency |
| MIMAT0000751 | miR-330-3p (and other miRNAs w/seed CAAAGCA) | TargetScan Human | Moderate (predicted) | BMPR2 | Adipogenesis pathway,BMP signaling pathway,Cardiomyocyte Differentiation via BMP Receptors,Epithelial Adherens Junction Signaling,Factors Promoting Cardiogenesis in Vertebrates,Human Embryonic Stem Cell Pluripotency,Molecular Mechanisms of Cancer,Mouse Embryonic Stem Cell Pluripotency,NF-κB Signaling,PPARα/RXRα Activation,PTEN Signaling,Role of NANOG in Mammalian Embryonic Stem Cell Pluripotency,Role of Osteoblasts, Osteoclasts and Chondrocytes in Rheumatoid Arthritis,STAT3 Pathway,TGF-β Signaling,Wnt/β-catenin Signaling |
| MIMAT0000751 | miR-330-3p (and other miRNAs w/seed CAAAGCA) | TargetScan Human | Moderate (predicted) | CALCR | G-Protein Coupled Receptor Signaling,Gαq Signaling,Role of Osteoblasts, Osteoclasts and Chondrocytes in Rheumatoid Arthritis |
| MIMAT0000751 | miR-330-3p (and other miRNAs w/seed CAAAGCA) | TargetScan Human | Moderate (predicted) | CAMK4 | Androgen Signaling,B Cell Receptor Signaling,BMP signaling pathway,Breast Cancer Regulation by Stathmin1,Calcium Signaling,Calcium-induced T Lymphocyte Apoptosis,cAMP-mediated signaling,Cardiac Hypertrophy Signaling,CCR3 Signaling in Eosinophils,CCR5 Signaling in Macrophages,CD28 Signaling in T Helper Cells,Cellular Effects of Sildenafil (Viagra),Chemokine Signaling,Corticotropin Releasing Hormone Signaling,CREB Signaling in Neurons,Dopamine-DARPP32 Feedback in cAMP Signaling,eNOS Signaling,fMLP Signaling in Neutrophils,G-Protein Coupled Receptor Signaling,Glioma Signaling,Glutamate Receptor Signaling,Gαq Signaling,iCOS-iCOSL Signaling in T Helper Cells,iNOS Signaling,Melatonin Signaling,Neuropathic Pain Signaling In Dorsal Horn Neurons,Nitric Oxide Signaling in the Cardiovascular System,nNOS Signaling in Neurons,nNOS Signaling in Skeletal Muscle Cells,Nur77 Signaling in T Lymphocytes,Phospholipase C Signaling,PI3K Signaling in B Lymphocytes,Protein Kinase A Signaling,RANK Signaling in |

| | | | | | |
|---|---|---|---|---|---|
| | | | | | Osteoclasts,Regulation of IL-2 Expression in Activated and Anergic T Lymphocytes,Role of Macrophages, Fibroblasts and Endothelial Cells in Rheumatoid Arthritis,Role of NFAT in Cardiac Hypertrophy,Role of NFAT in Regulation of the Immune Response,Role of Osteoblasts, Osteoclasts and Chondrocytes in Rheumatoid Arthritis,Sperm Motility,Synaptic Long Term Potentiation,Systemic Lupus Erythematosus Signaling,T Cell Receptor Signaling,Thrombin Signaling,TR/RXR Activation,Xenobiotic Metabolism Signaling,α-Adrenergic Signaling |
| MIMAT0000751 | miR-330-3p (and other miRNAs w/seed CAAAGCA) | TargetScan Human | Moderate (predicted) | CDKN1B | 14-3-3-mediated Signaling,Antiproliferative Role of Somatostatin Receptor 2,Antiproliferative Role of TOB in T Cell Signaling,Aryl Hydrocarbon Receptor Signaling,Breast Cancer Regulation by Stathmin1,Cell Cycle: G1/S Checkpoint Regulation,Chronic Myeloid Leukemia Signaling,Cyclins and Cell Cycle Regulation,ErbB2-ErbB3 Signaling,Estrogen-mediated S-phase Entry,Glioblastoma Multiforme Signaling,HER-2 Signaling in Breast Cancer,Molecular Mechanisms of Cancer,Neuregulin Signaling,Pancreatic Adenocarcinoma Signaling,PI3K/AKT Signaling,Prostate Cancer Signaling,PTEN Signaling,Regulation of Cellular Mechanics by Calpain Protease,Small Cell Lung Cancer Signaling,VDR/RXR Activation |
| MIMAT0000751 | miR-330-3p (and other miRNAs w/seed CAAAGCA) | TargetScan Human | Moderate (predicted) | CREM | cAMP-mediated signaling,Cholecystokinin/Gastrin-mediated Signaling,Dopamine-DARPP32 Feedback in cAMP Signaling,Protein Kinase A Signaling,Systemic Lupus Erythematosus Signaling |
| MIMAT0000751 | miR-330-3p (and other miRNAs w/seed CAAAGCA) | TargetScan Human | Moderate (predicted) | CRK | Actin Cytoskeleton Signaling,Angiopoietin Signaling,Axonal Guidance Signaling,Chronic Myeloid Leukemia Signaling,CXCR4 Signaling,Ephrin Receptor Signaling,Epithelial Adherens Junction Signaling,ERK/MAPK Signaling,FAK Signaling,Fcγ Receptor-mediated Phagocytosis in Macrophages and Monocytes,FGF Signaling,Insulin Receptor Signaling,Integrin Signaling,Leukocyte Extravasation Signaling,Melanocyte Development and Pigmentation Signaling,Molecular Mechanisms of Cancer,Neuregulin Signaling,NGF Signaling,Paxillin Signaling,PDGF Signaling,Renal Cell Carcinoma Signaling,Role of PI3K/AKT Signaling in the Pathogenesis of Influenza,SAPK/JNK Signaling |
| MIMAT0000751 | miR-330-3p (and other miRNAs w/seed CAAAGCA) | TargetScan Human | Moderate (predicted) | CSNK1G3 | Dopamine-DARPP32 Feedback in cAMP Signaling,Gap Junction Signaling,Role of NFAT in Regulation of the Immune Response,Role of Wnt/GSK-3β Signaling in the Pathogenesis of Influenza,Wnt/β-catenin Signaling |

| | | | | | |
|---|---|---|---|---|---|
| MIMAT0000751 | miR-330-3p (and other miRNAs w/seed CAAAGCA) | TargetScan Human | Moderate (predicted) | CTNNB1 | Basal Cell Carcinoma Signaling,Colorectal Cancer Metastasis Signaling,Endometrial Cancer Signaling,Ephrin B Signaling,Epithelial Adherens Junction Signaling,Factors Promoting Cardiogenesis in Vertebrates,Gap Junction Signaling,Germ Cell-Sertoli Cell Junction Signaling,Glioblastoma Multiforme Signaling,Gα12/13 Signaling,Human Embryonic Stem Cell Pluripotency,ILK Signaling,Leukocyte Extravasation Signaling,Molecular Mechanisms of Cancer,Mouse Embryonic Stem Cell Pluripotency,Ovarian Cancer Signaling,p53 Signaling,PI3K/AKT Signaling,Polyamine Regulation in Colon Cancer,Prostate Cancer Signaling,Protein Kinase A Signaling,Regulation of the Epithelial-Mesenchymal Transition Pathway,Remodeling of Epithelial Adherens Junctions,Role of Macrophages, Fibroblasts and Endothelial Cells in Rheumatoid Arthritis,Role of NANOG in Mammalian Embryonic Stem Cell Pluripotency,Role of Osteoblasts, Osteoclasts and Chondrocytes in Rheumatoid Arthritis,Role of Wnt/GSK-3β Signaling in the Pathogenesis of Influenza,Sertoli Cell-Sertoli Cell Junction Signaling,Thyroid Cancer Signaling,Tight Junction Signaling,Wnt/β-catenin Signaling |
| MIMAT0000751 | miR-330-3p (and other miRNAs w/seed CAAAGCA) | TargetScan Human | Moderate (predicted) | DNM1L | Clathrin-mediated Endocytosis Signaling,GNRH Signaling,Huntington's Disease Signaling,Remodeling of Epithelial Adherens Junctions |
| MIMAT0000751 | miR-330-3p (and other miRNAs w/seed CAAAGCA) | TargetScan Human | Moderate (predicted) | DNM3 | Clathrin-mediated Endocytosis Signaling,GNRH Signaling,Huntington's Disease Signaling,Remodeling of Epithelial Adherens Junctions |
| MIMAT0000751 | miR-330-3p (and other miRNAs w/seed CAAAGCA) | TargetScan Human | Moderate (predicted) | DPYSL3 | Semaphorin Signaling in Neurons |
| MIMAT0000751 | miR-330-3p (and other miRNAs w/seed CAAAGCA) | TargetScan Human | Moderate (predicted) | EDEM1 | Unfolded protein response |
| MIMAT0000751 | miR-330-3p (and other miRNAs w/seed CAAAGCA) | TargetScan Human | Moderate (predicted) | EGF | Actin Cytoskeleton Signaling,Axonal Guidance Signaling,Bladder Cancer Signaling,Caveolar-mediated Endocytosis Signaling,Clathrin-mediated Endocytosis Signaling,Colorectal Cancer Metastasis Signaling,EGF Signaling,Ephrin Receptor Signaling,Epithelial Adherens Junction |

| | | | | | |
|---|---|---|---|---|---|
| | | | | | Signaling,ErbB Signaling,ERK5 Signaling,FAK Signaling,Gap Junction Signaling,Glioblastoma Multiforme Signaling,Glioma Signaling,GNRH Signaling,Hepatic Fibrosis / Hepatic Stellate Cell Activation,HER-2 Signaling in Breast Cancer,Huntington's Disease Signaling,IL-8 Signaling,Macropinocytosis Signaling,Neuregulin Signaling,NF-κB Signaling,Non-Small Cell Lung Cancer Signaling,Ovarian Cancer Signaling,Pancreatic Adenocarcinoma Signaling,Regulation of Cellular Mechanics by Calpain Protease,Regulation of the Epithelial-Mesenchymal Transition Pathway,Telomerase Signaling,Thrombin Signaling |
| MIMAT0000751 | miR-330-3p (and other miRNAs w/seed CAAAGCA) | TargetScan Human | Moderate (predicted) | ELAVL1 | AMPK Signaling,VEGF Signaling |
| MIMAT0000751 | miR-330-3p (and other miRNAs w/seed CAAAGCA) | TargetScan Human | Moderate (predicted) | ELF1 | ERK/MAPK Signaling,HGF Signaling,Telomerase Signaling |
| MIMAT0000751 | miR-330-3p (and other miRNAs w/seed CAAAGCA) | TargetScan Human | Moderate (predicted) | EPM2A | Protein Kinase A Signaling |
| MIMAT0000751 | miR-330-3p (and other miRNAs w/seed CAAAGCA) | TargetScan Human | Moderate (predicted) | ERBB4 | Agrin Interactions at Neuromuscular Junction,ErbB Signaling,ErbB4 Signaling,Neuregulin Signaling |
| MIMAT0000751 | miR-330-3p (and other miRNAs w/seed CAAAGCA) | TargetScan Human | Moderate (predicted) | FGFR1 | Adipogenesis pathway,Epithelial Adherens Junction Signaling,FGF Signaling,Hepatic Fibrosis / Hepatic Stellate Cell Activation,Human Embryonic Stem Cell Pluripotency,NF-κB Signaling,PTEN Signaling,Regulation of the Epithelial-Mesenchymal Transition Pathway,STAT3 Pathway |
| MIMAT0000751 | miR-330-3p (and other miRNAs w/seed CAAAGCA) | TargetScan Human | Moderate (predicted) | FOXO1 | 14-3-3-mediated Signaling,Adipogenesis pathway,AMPK Signaling,Angiopoietin Signaling,B Cell Receptor Signaling,Cell Cycle: G1/S Checkpoint Regulation,Docosahexaenoic Acid (DHA) Signaling,ErbB Signaling,ErbB2-ErbB3 Signaling,FXR/RXR Activation,Glioblastoma Multiforme Signaling,HER-2 Signaling in Breast Cancer,Human Embryonic Stem Cell Pluripotency,IGF-1 Signaling,IL-3 Signaling,Insulin Receptor Signaling,Leptin |

| | | | | | |
|---|---|---|---|---|---|
| | | | | | Signaling in Obesity,Molecular Mechanisms of Cancer,PI3K/AKT Signaling,Prostate Cancer Signaling,PTEN Signaling,PXR/RXR Activation,Role of Osteoblasts, Osteoclasts and Chondrocytes in Rheumatoid Arthritis,VDR/RXR Activation,VEGF Signaling |
| MIMAT0000751 | miR-330-3p (and other miRNAs w/seed CAAAGCA) | TargetScan Human | Moderate (predicted) | FRK | IL-15 Production,Reelin Signaling in Neurons,Role of Tissue Factor in Cancer,Sperm Motility,Tec Kinase Signaling |
| MIMAT0000751 | miR-330-3p (and other miRNAs w/seed CAAAGCA) | TargetScan Human | Moderate (predicted) | FZD3 | Adipogenesis pathway,Axonal Guidance Signaling,Basal Cell Carcinoma Signaling,Colorectal Cancer Metastasis Signaling,Factors Promoting Cardiogenesis in Vertebrates,Glioblastoma Multiforme Signaling,Human Embryonic Stem Cell Pluripotency,Molecular Mechanisms of Cancer,Mouse Embryonic Stem Cell Pluripotency,Ovarian Cancer Signaling,PCP pathway,Regulation of the Epithelial-Mesenchymal Transition Pathway,Role of Macrophages, Fibroblasts and Endothelial Cells in Rheumatoid Arthritis,Role of NANOG in Mammalian Embryonic Stem Cell Pluripotency,Role of Osteoblasts, Osteoclasts and Chondrocytes in Rheumatoid Arthritis,Role of Wnt/GSK-3β Signaling in the Pathogenesis of Influenza,Wnt/Ca+ pathway,Wnt/β-catenin Signaling |
| MIMAT0000751 | miR-330-3p (and other miRNAs w/seed CAAAGCA) | TargetScan Human | Moderate (predicted) | GCG | GPCR-Mediated Integration of Enteroendocrine Signaling Exemplified by an L Cell,GPCR-Mediated Nutrient Sensing in Enteroendocrine Cells,Hepatic Cholestasis |
| MIMAT0000751 | miR-330-3p (and other miRNAs w/seed CAAAGCA) | TargetScan Human | Moderate (predicted) | GNA13 | Actin Cytoskeleton Signaling,Androgen Signaling,Axonal Guidance Signaling,Breast Cancer Regulation by Stathmin1,Cardiac Hypertrophy Signaling,Cholecystokinin/Gastrin-mediated Signaling,CREB Signaling in Neurons,CXCR4 Signaling,Endothelin-1 Signaling,Ephrin B Signaling,Ephrin Receptor Signaling,ERK5 Signaling,G Beta Gamma Signaling,Gα12/13 Signaling,IL-1 Signaling,IL-8 Signaling,Molecular Mechanisms of Cancer,Phospholipase C Signaling,Protein Kinase A Signaling,Relaxin Signaling,RhoA Signaling,RhoGDI Signaling,Role of NFAT in Regulation of the Immune Response,Role of Tissue Factor in Cancer,SAPK/JNK Signaling,Signaling by Rho Family GTPases,Sphingosine-1-phosphate Signaling,Synaptic Long Term Depression,Tec Kinase Signaling,Thrombin Signaling |
| MIMAT0000751 | miR-330-3p (and | TargetScan Human | Moderate (predicted) | GPHN | GABA Receptor Signaling,Molybdenum Cofactor Biosynthesis |

| | | | | | |
|---|---|---|---|---|---|
| | other miRNAs w/seed CAAAGCA) | | | | |
| MIMAT0000751 | miR-330-3p (and other miRNAs w/seed CAAAGCA) | TargetScan Human | Moderate (predicted) | GPR37 | Cellular Effects of Sildenafil (Viagra),GABA Receptor Signaling,Neuropathic Pain Signaling In Dorsal Horn Neurons,Parkinson's Signaling |
| MIMAT0000751 | miR-330-3p (and other miRNAs w/seed CAAAGCA) | TargetScan Human | Moderate (predicted) | GRIA3 | Amyotrophic Lateral Sclerosis Signaling,Calcium Signaling,CREB Signaling in Neurons,Glutamate Receptor Signaling,Neuropathic Pain Signaling In Dorsal Horn Neurons,Synaptic Long Term Depression,Synaptic Long Term Potentiation |
| MIMAT0000751 | miR-330-3p (and other miRNAs w/seed CAAAGCA) | TargetScan Human | Moderate (predicted) | GRIN2B | Amyotrophic Lateral Sclerosis Signaling,Calcium Signaling,Circadian Rhythm Signaling,CREB Signaling in Neurons,Dopamine-DARPP32 Feedback in cAMP Signaling,Ephrin Receptor Signaling,Glutamate Receptor Signaling,Huntington's Disease Signaling,Neuropathic Pain Signaling In Dorsal Horn Neurons,nNOS Signaling in Neurons,Synaptic Long Term Potentiation |
| MIMAT0000751 | miR-330-3p (and other miRNAs w/seed CAAAGCA) | TargetScan Human | Moderate (predicted) | GRM5 | CREB Signaling in Neurons,G-Protein Coupled Receptor Signaling,Glutamate Receptor Signaling,Gαq Signaling,Huntington's Disease Signaling,Neuropathic Pain Signaling In Dorsal Horn Neurons,Synaptic Long Term Depression,Synaptic Long Term Potentiation |
| MIMAT0000751 | miR-330-3p (and other miRNAs w/seed CAAAGCA) | TargetScan Human | Moderate (predicted) | GTF2H1 | Adipogenesis pathway,Androgen Signaling,Assembly of RNA Polymerase II Complex,Estrogen Receptor Signaling,Glucocorticoid Receptor Signaling,Nucleotide Excision Repair Pathway,RAR Activation |
| MIMAT0000751 | miR-330-3p (and other miRNAs w/seed CAAAGCA) | TargetScan Human | Moderate (predicted) | GTF2H5 | Adipogenesis pathway,Androgen Signaling,Assembly of RNA Polymerase II Complex,Estrogen Receptor Signaling,Glucocorticoid Receptor Signaling,Nucleotide Excision Repair Pathway,RAR Activation |
| MIMAT0000751 | miR-330-3p (and other miRNAs w/seed CAAAGCA) | TargetScan Human | Moderate (predicted) | GUCY1A3 | Antiproliferative Role of Somatostatin Receptor 2,Cellular Effects of Sildenafil (Viagra),Corticotropin Releasing Hormone Signaling,Dopamine-DARPP32 Feedback in cAMP Signaling,Endothelin-1 Signaling,eNOS Signaling,Gap Junction Signaling,Inhibition of Angiogenesis by TSP1,Nitric Oxide Signaling in the Cardiovascular System,Phototransduction Pathway,Relaxin Signaling,Sertoli Cell-Sertoli Cell Junction Signaling,Sperm Motility,Synaptic |

| | | | | | |
|---|---|---|---|---|---|
| | | | | | Long Term Depression |
| MIMAT0000751 | miR-330-3p (and other miRNAs w/seed CAAAGCA) | TargetScan Human | Moderate (predicted) | HSPH1 | Aldosterone Signaling in Epithelial Cells,Protein Ubiquitination Pathway,Unfolded protein response |
| MIMAT0000751 | miR-330-3p (and other miRNAs w/seed CAAAGCA) | TargetScan Human | Moderate (predicted) | IGF2BP1 | Role of Oct4 in Mammalian Embryonic Stem Cell Pluripotency |
| MIMAT0000751 | miR-330-3p (and other miRNAs w/seed CAAAGCA) | TargetScan Human | Moderate (predicted) | KAT2B | AMPK Signaling,Androgen Signaling,Cell Cycle: G2/M DNA Damage Checkpoint Regulation,Estrogen Receptor Signaling,Glucocorticoid Receptor Signaling,HMGB1 Signaling,p53 Signaling,RAR Activation |
| MIMAT0000751 | miR-330-3p (and other miRNAs w/seed CAAAGCA) | TargetScan Human | Moderate (predicted) | KCNJ6 | Dopamine-DARPP32 Feedback in cAMP Signaling,G Beta Gamma Signaling |
| MIMAT0000751 | miR-330-3p (and other miRNAs w/seed CAAAGCA) | TargetScan Human | Moderate (predicted) | MAP2K5 | Acute Myeloid Leukemia Signaling,CD27 Signaling in Lymphocytes,CD40 Signaling,Cholecystokinin/Gastrin-mediated Signaling,ERK5 Signaling,Gap Junction Signaling,HMGB1 Signaling,Melatonin Signaling,Neurotrophin/TRK Signaling,NRF2-mediated Oxidative Stress Response,Nur77 Signaling in T Lymphocytes,Regulation of the Epithelial-Mesenchymal Transition Pathway,Xenobiotic Metabolism Signaling |
| MIMAT0000751 | miR-330-3p (and other miRNAs w/seed CAAAGCA) | TargetScan Human | Moderate (predicted) | MEF2A | Calcium Signaling,Cardiac Hypertrophy Signaling,Cholecystokinin/Gastrin-mediated Signaling,Corticotropin Releasing Hormone Signaling,ERK5 Signaling,Gα12/13 Signaling,p38 MAPK Signaling,Phospholipase C Signaling,Role of NFAT in Cardiac Hypertrophy,Role of NFAT in Regulation of the Immune Response,Role of Oct4 in Mammalian Embryonic Stem Cell Pluripotency |
| MIMAT0000751 | miR-330-3p (and other miRNAs w/seed CAAAGCA) | TargetScan Human | Moderate (predicted) | MYH3 | Actin Cytoskeleton Signaling,Agranulocyte Adhesion and Diapedesis,Calcium Signaling,Cellular Effects of Sildenafil (Viagra),Epithelial Adherens Junction Signaling,Hepatic Fibrosis / Hepatic Stellate Cell Activation,ILK Signaling,Tight Junction Signaling |

| | | | | | |
|---|---|---|---|---|---|
| MIMAT0000751 | miR-330-3p (and other miRNAs w/seed CAAAGCA) | TargetScan Human | Moderate (predicted) | NRIP1 | Aryl Hydrocarbon Receptor Signaling,Estrogen Receptor Signaling,Glucocorticoid Receptor Signaling,PPAR Signaling,RAR Activation,Xenobiotic Metabolism Signaling |
| MIMAT0000751 | miR-330-3p (and other miRNAs w/seed CAAAGCA) | TargetScan Human | Moderate (predicted) | PCK1 | Estrogen Receptor Signaling,Glucocorticoid Receptor Signaling,TR/RXR Activation |
| MIMAT0000751 | miR-330-3p (and other miRNAs w/seed CAAAGCA) | TargetScan Human | Moderate (predicted) | PDE10A | cAMP-mediated signaling,Cardiac β-adrenergic Signaling,G-Protein Coupled Receptor Signaling,Gustation Pathway,Protein Kinase A Signaling,Relaxin Signaling,tRNA Splicing |
| MIMAT0000751 | miR-330-3p (and other miRNAs w/seed CAAAGCA) | TargetScan Human | Moderate (predicted) | PDE1A | cAMP-mediated signaling,Cardiac β-adrenergic Signaling,Cellular Effects of Sildenafil (Viagra),G-Protein Coupled Receptor Signaling,Gustation Pathway,Nitric Oxide Signaling in the Cardiovascular System,Protein Kinase A Signaling,Relaxin Signaling,Sperm Motility,tRNA Splicing |
| MIMAT0000751 | miR-330-3p (and other miRNAs w/seed CAAAGCA) | TargetScan Human | Moderate (predicted) | PDE5A | cAMP-mediated signaling,Cardiac β-adrenergic Signaling,Cellular Effects of Sildenafil (Viagra),G-Protein Coupled Receptor Signaling,Gustation Pathway,Nitric Oxide Signaling in the Cardiovascular System,Protein Kinase A Signaling,Relaxin Signaling,tRNA Splicing |
| MIMAT0000751 | miR-330-3p (and other miRNAs w/seed CAAAGCA) | TargetScan Human | Moderate (predicted) | PDGFD | Actin Cytoskeleton Signaling,Atherosclerosis Signaling,Axonal Guidance Signaling,Clathrin-mediated Endocytosis Signaling,Ephrin Receptor Signaling,Glioblastoma Multiforme Signaling,Glioma Signaling,Hepatic Fibrosis / Hepatic Stellate Cell Activation,Human Embryonic Stem Cell Pluripotency,Macropinocytosis Signaling,PAK Signaling,PDGF Signaling,PPAR Signaling,Regulation of the Epithelial-Mesenchymal Transition Pathway,Role of Macrophages, Fibroblasts and Endothelial Cells in Rheumatoid Arthritis,Sphingosine-1-phosphate Signaling |
| MIMAT0000751 | miR-330-3p (and other miRNAs w/seed CAAAGCA) | TargetScan Human | Moderate (predicted) | PHKB | Protein Kinase A Signaling,α-Adrenergic Signaling |
| MIMAT0000751 | miR-330-3p (and other miRNAs w/seed | TargetScan Human | Moderate (predicted) | PIK3R3 | 14-3-3-mediated Signaling,3-phosphoinositide Biosynthesis,Actin Cytoskeleton Signaling,Acute Myeloid Leukemia Signaling,Acute Phase Response Signaling,Aldosterone Signaling in Epithelial Cells,AMPK |

| | | | | | |
|---|---|---|---|---|---|
| | CAAAGCA) | | | | Signaling,Amyotrophic Lateral Sclerosis Signaling,Angiopoietin Signaling,Antiproliferative Role of Somatostatin Receptor 2,Axonal Guidance Signaling,B Cell Receptor Signaling,Breast Cancer Regulation by Stathmin1,Cardiac Hypertrophy Signaling,CCR3 Signaling in Eosinophils,CD28 Signaling in T Helper Cells,CD40 Signaling,Ceramide Signaling,Chronic Myeloid Leukemia Signaling,Clathrin-mediated Endocytosis Signaling,CNTF Signaling,Colorectal Cancer Metastasis Signaling,CREB Signaling in Neurons,CTLA4 Signaling in Cytotoxic T Lymphocytes,CXCR4 Signaling,Dendritic Cell Maturation,Docosahexaenoic Acid (DHA) Signaling,EGF Signaling,EIF2 Signaling,Endometrial Cancer Signaling,Endothelin-1 Signaling,eNOS Signaling,Ephrin A Signaling,ErbB Signaling,ErbB2-ErbB3 Signaling,ErbB4 Signaling,ERK/MAPK Signaling,Erythropoietin Signaling,Estrogen-Dependent Breast Cancer Signaling,FAK Signaling,Fc Epsilon RI Signaling,Fcγ Receptor-mediated Phagocytosis in Macrophages and Monocytes,FcγRIIB Signaling in B Lymphocytes,FGF Signaling,FLT3 Signaling in Hematopoietic Progenitor Cells,fMLP Signaling in Neutrophils,G-Protein Coupled Receptor Signaling,Gap Junction Signaling,GDNF Family Ligand-Receptor Interactions,Germ Cell-Sertoli Cell Junction Signaling,Glioblastoma Multiforme Signaling,Glioma Invasiveness Signaling,Glioma Signaling,Glucocorticoid Receptor Signaling,GM-CSF Signaling,Growth Hormone Signaling,Gα12/13 Signaling,Gαq Signaling,HER-2 Signaling in Breast Cancer,Hereditary Breast Cancer Signaling,HGF Signaling,HIF1α Signaling,HMGB1 Signaling,Human Embryonic Stem Cell Pluripotency,Huntington's Disease Signaling,iCOS-iCOSL Signaling in T Helper Cells,IGF-1 Signaling,IL-12 Signaling and Production in Macrophages,IL-15 Signaling,IL-17 Signaling,IL-17A Signaling in Airway Cells,IL-2 Signaling,IL-3 Signaling,IL-4 Signaling,IL-6 Signaling,IL-8 Signaling,IL-9 Signaling,ILK Signaling,Insulin Receptor Signaling,Integrin Signaling,JAK/Stat Signaling,Leptin Signaling in Obesity,Leukocyte Extravasation Signaling,LPS-stimulated MAPK Signaling,Lymphotoxin β Receptor Signaling,Macropinocytosis Signaling,Melanocyte Development and Pigmentation Signaling,Melanoma Signaling,Molecular Mechanisms of Cancer,Mouse Embryonic Stem Cell Pluripotency,MSP-RON Signaling Pathway,mTOR Signaling,Myc Mediated Apoptosis Signaling,Natural Killer Cell Signaling,Neuregulin Signaling,Neuropathic Pain Signaling In Dorsal Horn Neurons,Neurotrophin/TRK Signaling,NF-κB Activation by Viruses,NF-κB Signaling,NGF Signaling,Nitric Oxide Signaling in the Cardiovascular System,Non-Small Cell Lung Cancer Signaling,NRF2-mediated Oxidative Stress Response,Ovarian Cancer Signaling,P2Y Purigenic Receptor Signaling Pathway,p53 Signaling,p70S6K Signaling,PAK Signaling,Pancreatic Adenocarcinoma Signaling,Paxillin Signaling,PDGF Signaling,PEDF |

| | | | | | |
|---|---|---|---|---|---|
| | | | | | Signaling,phagosome formation,PI3K/AKT Signaling,PKCθ Signaling in T Lymphocytes,Production of Nitric Oxide and Reactive Oxygen Species in Macrophages,Prolactin Signaling,Prostate Cancer Signaling,PTEN Signaling,Rac Signaling,RANK Signaling in Osteoclasts,RAR Activation,Reelin Signaling in Neurons,Regulation of eIF4 and p70S6K Signaling,Regulation of the Epithelial-Mesenchymal Transition Pathway,Relaxin Signaling,Renal Cell Carcinoma Signaling,Renin-Angiotensin Signaling,Role of IL-17A in Arthritis,Role of JAK1 and JAK3 in γc Cytokine Signaling,Role of Macrophages, Fibroblasts and Endothelial Cells in Rheumatoid Arthritis,Role of NANOG in Mammalian Embryonic Stem Cell Pluripotency,Role of NFAT in Cardiac Hypertrophy,Role of NFAT in Regulation of the Immune Response,Role of Osteoblasts, Osteoclasts and Chondrocytes in Rheumatoid Arthritis,Role of p14/p19ARF in Tumor Suppression,Role of Pattern Recognition Receptors in Recognition of Bacteria and Viruses,Role of PI3K/AKT Signaling in the Pathogenesis of Influenza,Role of Tissue Factor in Cancer,SAPK/JNK Signaling,Signaling by Rho Family GTPases,Small Cell Lung Cancer Signaling,Sphingosine-1-phosphate Signaling,Superpathway of Inositol Phosphate Compounds,Systemic Lupus Erythematosus Signaling,T Cell Receptor Signaling,Tec Kinase Signaling,Telomerase Signaling,Thrombin Signaling,Thrombopoietin Signaling,TR/RXR Activation,Type II Diabetes Mellitus Signaling,UVA-Induced MAPK Signaling,UVB-Induced MAPK Signaling,VEGF Family Ligand-Receptor Interactions,VEGF Signaling,Virus Entry via Endocytic Pathways,Xenobiotic Metabolism Signaling |
| MIMAT0000751 | miR-330-3p (and other miRNAs w/seed CAAAGCA) | TargetScan Human | Moderate (predicted) | PPM1B | AMPK Signaling |
| MIMAT0000751 | miR-330-3p (and other miRNAs w/seed CAAAGCA) | TargetScan Human | Moderate (predicted) | PPP1R1B | 3-phosphoinositide Biosynthesis,3-phosphoinositide Degradation,CDK5 Signaling,D-myo-inositol (1,4,5,6)-Tetrakisphosphate Biosynthesis,D-myo-inositol (3,4,5,6)-tetrakisphosphate Biosynthesis,D-myo-inositol-5-phosphate Metabolism,Dopamine Receptor Signaling,Dopamine-DARPP32 Feedback in cAMP Signaling,Protein Kinase A Signaling,Superpathway of Inositol Phosphate Compounds |
| MIMAT0000751 | miR-330-3p (and other miRNAs w/seed CAAAGCA) | TargetScan Human | High (predicted) | PRKAB2 | AMPK Signaling,eNOS Signaling,Glucocorticoid Receptor Signaling,mTOR Signaling,PPARα/RXRα Activation,Type II Diabetes Mellitus Signaling |

| | | | | | |
|---|---|---|---|---|---|
| MIMAT0000751 | miR-330-3p (and other miRNAs w/seed CAAAGCA) | TargetScan Human | Moderate (predicted) | PRKACB | AMPK Signaling,Amyloid Processing,Androgen Signaling,Axonal Guidance Signaling,BMP signaling pathway,Breast Cancer Regulation by Stathmin1,Calcium Signaling,cAMP-mediated signaling,Cardiac Hypertrophy Signaling,Cardiac β-adrenergic Signaling,CDK5 Signaling,Cellular Effects of Sildenafil (Viagra),Colorectal Cancer Metastasis Signaling,Corticotropin Releasing Hormone Signaling,CREB Signaling in Neurons,Dopamine Receptor Signaling,Dopamine-DARPP32 Feedback in cAMP Signaling,eNOS Signaling,ERK/MAPK Signaling,G Beta Gamma Signaling,G-Protein Coupled Receptor Signaling,Gap Junction Signaling,Glucocorticoid Receptor Signaling,GNRH Signaling,GPCR-Mediated Integration of Enteroendocrine Signaling Exemplified by an L Cell,GPCR-Mediated Nutrient Sensing in Enteroendocrine Cells,Gustation Pathway,Gαi Signaling,Gαs Signaling,Hepatic Cholestasis,IGF-1 Signaling,IL-1 Signaling,Insulin Receptor Signaling,Leptin Signaling in Obesity,Melanocyte Development and Pigmentation Signaling,Melatonin Signaling,Molecular Mechanisms of Cancer,Netrin Signaling,Neuropathic Pain Signaling In Dorsal Horn Neurons,Neuroprotective Role of THOP1 in Alzheimer's Disease,NF-κB Signaling,Nitric Oxide Signaling in the Cardiovascular System,Ovarian Cancer Signaling,P2Y Purigenic Receptor Signaling Pathway,Phototransduction Pathway,PPARα/RXRα Activation,Protein Kinase A Signaling,PXR/RXR Activation,RAR Activation,Relaxin Signaling,Renin-Angiotensin Signaling,Role of NFAT in Cardiac Hypertrophy,Sertoli Cell-Sertoli Cell Junction Signaling,Sonic Hedgehog Signaling,Sperm Motility,Synaptic Long Term Potentiation,Tight Junction Signaling,α-Adrenergic Signaling |
| MIMAT0000751 | miR-330-3p (and other miRNAs w/seed CAAAGCA) | TargetScan Human | Moderate (predicted) | PRKCB | 14-3-3-mediated Signaling,Aldosterone Signaling in Epithelial Cells,Androgen Signaling,Axonal Guidance Signaling,B Cell Receptor Signaling,Breast Cancer Regulation by Stathmin1,Calcium-induced T Lymphocyte Apoptosis,CCR3 Signaling in Eosinophils,CCR5 Signaling in Macrophages,Chemokine Signaling,Cholecystokinin/Gastrin-mediated Signaling,Corticotropin Releasing Hormone Signaling,CREB Signaling in Neurons,CXCR4 Signaling,Dopamine-DARPP32 Feedback in cAMP Signaling,Endothelin-1 Signaling,eNOS Signaling,ErbB Signaling,ErbB4 Signaling,ERK/MAPK Signaling,Erythropoietin Signaling,Factors Promoting Cardiogenesis in Vertebrates,Fc Epsilon RI Signaling,Fcγ Receptor-mediated Phagocytosis in Macrophages and Monocytes,fMLP Signaling in Neutrophils,G Beta Gamma Signaling,G-Protein Coupled Receptor Signaling,Gap Junction Signaling,Glioma Signaling,GM-CSF Signaling,GNRH Signaling,GPCR-Mediated Nutrient Sensing in Enteroendocrine Cells,Growth Hormone Signaling,Gαq Signaling,Hepatic Cholestasis,HER-2 Signaling in Breast Cancer,HGF Signaling,Huntington's Disease Signaling,IL-12 Signaling and Production in Macrophages,IL-3 |

| | | | | | |
|---|---|---|---|---|---|
| | | | | | Signaling,IL-8 Signaling,Leukocyte Extravasation Signaling,LPS-stimulated MAPK Signaling,Macropinocytosis Signaling,Mechanisms of Viral Exit from Host Cells,Melatonin Signaling,Molecular Mechanisms of Cancer,mTOR Signaling,Natural Killer Cell Signaling,Neuregulin Signaling,Neuropathic Pain Signaling In Dorsal Horn Neurons,NF-κB Activation by Viruses,NF-κB Signaling,Nitric Oxide Signaling in the Cardiovascular System,nNOS Signaling in Neurons,NRF2-mediated Oxidative Stress Response,P2Y Purigenic Receptor Signaling Pathway,p70S6K Signaling,PDGF Signaling,phagosome formation,Phospholipase C Signaling,PI3K Signaling in B Lymphocytes,PPARα/RXRα Activation,Production of Nitric Oxide and Reactive Oxygen Species in Macrophages,Prolactin Signaling,Protein Kinase A Signaling,RAR Activation,Renin-Angiotensin Signaling,Role of Macrophages, Fibroblasts and Endothelial Cells in Rheumatoid Arthritis,Role of NFAT in Cardiac Hypertrophy,Role of Pattern Recognition Receptors in Recognition of Bacteria and Viruses,Sperm Motility,Synaptic Long Term Depression,Synaptic Long Term Potentiation,Tec Kinase Signaling,Thrombin Signaling,Thrombopoietin Signaling,Type II Diabetes Mellitus Signaling,UVB-Induced MAPK Signaling,UVC-Induced MAPK Signaling,VDR/RXR Activation,VEGF Family Ligand-Receptor Interactions,VEGF Signaling,Virus Entry via Endocytic Pathways,Xenobiotic Metabolism Signaling,α-Adrenergic Signaling |
| MIMAT0000751 | miR-330-3p (and other miRNAs w/seed CAAAGCA) | TargetScan Human | Moderate (predicted) | PTPRA | Protein Kinase A Signaling |
| MIMAT0000751 | miR-330-3p (and other miRNAs w/seed CAAAGCA) | TargetScan Human | Moderate (predicted) | PTPRM | 3-phosphoinositide Biosynthesis,3-phosphoinositide Degradation,D-myo-inositol (1,4,5,6)-Tetrakisphosphate Biosynthesis,D-myo-inositol (3,4,5,6)-tetrakisphosphate Biosynthesis,D-myo-inositol-5-phosphate Metabolism,Epithelial Adherens Junction Signaling,Protein Kinase A Signaling,Superpathway of Inositol Phosphate Compounds |
| MIMAT0000751 | miR-330-3p (and other miRNAs w/seed CAAAGCA) | TargetScan Human | Moderate (predicted) | RALA | Cdc42 Signaling,Gαi Signaling,Integrin Signaling,Molecular Mechanisms of Cancer,Pancreatic Adenocarcinoma Signaling,Phospholipase C Signaling,Remodeling of Epithelial Adherens Junctions |
| MIMAT0000751 | miR-330-3p (and other miRNAs w/seed | TargetScan Human | Moderate (predicted) | RAP1B | Antiproliferative Role of Somatostatin Receptor 2,Axonal Guidance Signaling,B Cell Receptor Signaling,Calcium Signaling,Corticotropin Releasing Hormone Signaling,Ephrin Receptor Signaling,Epithelial Adherens Junction |

| | | | | | |
|---|---|---|---|---|---|
| | CAAAGCA) | | | | Signaling,ERK/MAPK Signaling,HGF Signaling,Integrin Signaling,Leukocyte Extravasation Signaling,Molecular Mechanisms of Cancer,NGF Signaling,Phospholipase C Signaling,Production of Nitric Oxide and Reactive Oxygen Species in Macrophages,Protein Kinase A Signaling,Relaxin Signaling,Synaptic Long Term Potentiation |
| MIMAT0000751 | miR-330-3p (and other miRNAs w/seed CAAAGCA) | TargetScan Human | Moderate (predicted) | RAP2A | B Cell Receptor Signaling,Calcium Signaling,Integrin Signaling,Molecular Mechanisms of Cancer |
| MIMAT0000751 | miR-330-3p (and other miRNAs w/seed CAAAGCA) | TargetScan Human | Moderate (predicted) | RAP2B | B Cell Receptor Signaling,Calcium Signaling,Integrin Signaling,Molecular Mechanisms of Cancer |
| MIMAT0000751 | miR-330-3p (and other miRNAs w/seed CAAAGCA) | TargetScan Human | Moderate (predicted) | RASA1 | 3-phosphoinositide Biosynthesis,3-phosphoinositide Degradation,Angiopoietin Signaling,Axonal Guidance Signaling,Cdc42 Signaling,D-myo-inositol (1,4,5,6)-Tetrakisphosphate Biosynthesis,D-myo-inositol (3,4,5,6)-tetrakisphosphate Biosynthesis,D-myo-inositol-5-phosphate Metabolism,EGF Signaling,Ephrin Receptor Signaling,G-Protein Coupled Receptor Signaling,GDNF Family Ligand-Receptor Interactions,Gα12/13 Signaling,Huntington's Disease Signaling,IGF-1 Signaling,Molecular Mechanisms of Cancer,PDGF Signaling,Superpathway of Inositol Phosphate Compounds,T Cell Receptor Signaling |
| MIMAT0000751 | miR-330-3p (and other miRNAs w/seed CAAAGCA) | TargetScan Human | High (predicted) | RCAN1 | Calcium Signaling,Role of NFAT in Cardiac Hypertrophy,Role of NFAT in Regulation of the Immune Response |
| MIMAT0000751 | miR-330-3p (and other miRNAs w/seed CAAAGCA) | TargetScan Human | Moderate (predicted) | RHEB | mTOR Signaling,PI3K/AKT Signaling |
| MIMAT0000751 | miR-330-3p (and other miRNAs w/seed CAAAGCA) | TargetScan Human | Moderate (predicted) | RHOQ | Actin Nucleation by ARP-WASP Complex,Cardiac Hypertrophy Signaling,Cholecystokinin/Gastrin-mediated Signaling,Colorectal Cancer Metastasis Signaling,CXCR4 Signaling,Germ Cell-Sertoli Cell Junction Signaling,Glioblastoma Multiforme Signaling,Glioma Invasiveness Signaling,Gαq Signaling,HMGB1 Signaling,IL-8 Signaling,ILK Signaling,Insulin |

| | | | | | |
|---|---|---|---|---|---|
| | | | | | Receptor Signaling,Integrin Signaling,Molecular Mechanisms of Cancer,mTOR Signaling,phagosome formation,Phospholipase C Signaling,Production of Nitric Oxide and Reactive Oxygen Species in Macrophages,Regulation of Actin-based Motility by Rho,RhoGDI Signaling,Semaphorin Signaling in Neurons,Signaling by Rho Family GTPases,Sphingosine-1-phosphate Signaling,Tec Kinase Signaling,Thrombin Signaling |
| MIMAT0000751 | miR-330-3p (and other miRNAs w/seed CAAAGCA) | TargetScan Human | Moderate (predicted) | RHOT2 | Actin Nucleation by ARP-WASP Complex,Cardiac Hypertrophy Signaling,Cholecystokinin/Gastrin-mediated Signaling,Colorectal Cancer Metastasis Signaling,CXCR4 Signaling,Germ Cell-Sertoli Cell Junction Signaling,Glioblastoma Multiforme Signaling,Glioma Invasiveness Signaling,Gαq Signaling,HMGB1 Signaling,IL-8 Signaling,ILK Signaling,Integrin Signaling,Mitochondrial Dysfunction,Molecular Mechanisms of Cancer,mTOR Signaling,phagosome formation,Phospholipase C Signaling,Production of Nitric Oxide and Reactive Oxygen Species in Macrophages,Regulation of Actin-based Motility by Rho,RhoGDI Signaling,Semaphorin Signaling in Neurons,Signaling by Rho Family GTPases,Sphingosine-1-phosphate Signaling,Tec Kinase Signaling,Thrombin Signaling |
| MIMAT0000751 | miR-330-3p (and other miRNAs w/seed CAAAGCA) | TargetScan Human | High (predicted) | RND3 | Actin Nucleation by ARP-WASP Complex,Cardiac Hypertrophy Signaling,Cholecystokinin/Gastrin-mediated Signaling,Colorectal Cancer Metastasis Signaling,CXCR4 Signaling,Germ Cell-Sertoli Cell Junction Signaling,Glioblastoma Multiforme Signaling,Glioma Invasiveness Signaling,Gαq Signaling,HMGB1 Signaling,IL-8 Signaling,ILK Signaling,Integrin Signaling,Molecular Mechanisms of Cancer,mTOR Signaling,phagosome formation,Phospholipase C Signaling,Production of Nitric Oxide and Reactive Oxygen Species in Macrophages,Regulation of Actin-based Motility by Rho,RhoA Signaling,RhoGDI Signaling,Semaphorin Signaling in Neurons,Signaling by Rho Family GTPases,Sphingosine-1-phosphate Signaling,Tec Kinase Signaling,Thrombin Signaling |
| MIMAT0000751 | miR-330-3p (and other miRNAs w/seed CAAAGCA) | TargetScan Human | Moderate (predicted) | SALL4 | Human Embryonic Stem Cell Pluripotency,Role of NANOG in Mammalian Embryonic Stem Cell Pluripotency,Role of Oct4 in Mammalian Embryonic Stem Cell Pluripotency |
| MIMAT0000751 | miR-330-3p (and other miRNAs w/seed CAAAGCA) | TargetScan Human | Moderate (predicted) | SCGB1A1 | Glucocorticoid Receptor Signaling |

| | | | | | |
|---|---|---|---|---|---|
| MIMAT0000751 | miR-330-3p (and other miRNAs w/seed CAAAGCA) | TargetScan Human | Moderate (predicted) | SEMA4D | Axonal Guidance Signaling,Semaphorin Signaling in Neurons |
| MIMAT0000751 | miR-330-3p (and other miRNAs w/seed CAAAGCA) | TargetScan Human | Moderate (predicted) | SMARCC1 | AMPK Signaling,Glucocorticoid Receptor Signaling,Hereditary Breast Cancer Signaling,RAR Activation,Role of BRCA1 in DNA Damage Response |
| MIMAT0000751 | miR-330-3p (and other miRNAs w/seed CAAAGCA) | TargetScan Human | Moderate (predicted) | SYK | B Cell Receptor Signaling,CD28 Signaling in T Helper Cells,CTLA4 Signaling in Cytotoxic T Lymphocytes,Fc Epsilon RI Signaling,Fcγ Receptor-mediated Phagocytosis in Macrophages and Monocytes,FcγRIIB Signaling in B Lymphocytes,IL-15 Signaling,IL-2 Signaling,Natural Killer Cell Signaling,p70S6K Signaling,phagosome formation,Phospholipase C Signaling,PI3K Signaling in B Lymphocytes,Role of JAK1 and JAK3 in γc Cytokine Signaling,Role of NFAT in Regulation of the Immune Response,Role of Pattern Recognition Receptors in Recognition of Bacteria and Viruses |
| MIMAT0000751 | miR-330-3p (and other miRNAs w/seed CAAAGCA) | TargetScan Human | Moderate (predicted) | TAF1B | Assembly of RNA Polymerase I Complex |
| MIMAT0000751 | miR-330-3p (and other miRNAs w/seed CAAAGCA) | TargetScan Human | Moderate (predicted) | TAF5 | Assembly of RNA Polymerase II Complex,Estrogen Receptor Signaling,Glucocorticoid Receptor Signaling |
| MIMAT0000751 | miR-330-3p (and other miRNAs w/seed CAAAGCA) | TargetScan Human | Moderate (predicted) | TGFBR3 | Epithelial Adherens Junction Signaling,Factors Promoting Cardiogenesis in Vertebrates,NF-κB Signaling,PPARα/RXRα Activation,PTEN Signaling,Sertoli Cell-Sertoli Cell Junction Signaling,STAT3 Pathway,Wnt/β-catenin Signaling |
| MIMAT0000751 | miR-330-3p (and other miRNAs w/seed CAAAGCA) | TargetScan Human | Moderate (predicted) | TRPC3 | Calcium Signaling |
| MIMAT0000751 | miR-330-3p (and other miRNAs w/seed | TargetScan Human | Moderate (predicted) | UBXN4 | Unfolded protein response |

| | | | | | |
|---|---|---|---|---|---|
| | CAAAGCA) | | | | |
| MIMAT0000751 | miR-330-3p (and other miRNAs w/seed CAAAGCA) | TargetScan Human | Moderate (predicted) | UNC5D | Axonal Guidance Signaling,Netrin Signaling |
| MIMAT0000751 | miR-330-3p (and other miRNAs w/seed CAAAGCA) | TargetScan Human | Moderate (predicted) | VRK2 | ERK/MAPK Signaling |
| MIMAT0000751 | miR-330-3p (and other miRNAs w/seed CAAAGCA) | TargetScan Human | Moderate (predicted) | YWHAH | 14-3-3-mediated Signaling,Cell Cycle: G2/M DNA Damage Checkpoint Regulation,ERK/MAPK Signaling,ERK5 Signaling,Glucocorticoid Receptor Signaling,HIPPO signaling,IGF-1 Signaling,Myc Mediated Apoptosis Signaling,p70S6K Signaling,PI3K/AKT Signaling,Protein Kinase A Signaling,PTEN Signaling |
| MIMAT0001638 | miR-409-5p (and other miRNAs w/seed GGUUACC) | TargetScan Human | High (predicted) | ADAM10 | Axonal Guidance Signaling,Ephrin A Signaling,Ephrin Receptor Signaling,Inhibition of Matrix Metalloproteases |
| MIMAT0001638 | miR-409-5p (and other miRNAs w/seed GGUUACC) | TargetScan Human | Moderate (predicted) | ADAMTS6 | Axonal Guidance Signaling |
| MIMAT0001638 | miR-409-5p (and other miRNAs w/seed GGUUACC) | TargetScan Human | Moderate (predicted) | ADCY9 | Breast Cancer Regulation by Stathmin1,cAMP-mediated signaling,Cardiac Hypertrophy Signaling,Cardiac β-adrenergic Signaling,CDK5 Signaling,Cellular Effects of Sildenafil (Viagra),Colorectal Cancer Metastasis Signaling,Corticotropin Releasing Hormone Signaling,CREB Signaling in Neurons,CXCR4 Signaling,Dopamine Receptor Signaling,Dopamine-DARPP32 Feedback in cAMP Signaling,Endothelin-1 Signaling,eNOS Signaling,G-Protein Coupled Receptor Signaling,GABA Receptor Signaling,Gap Junction Signaling,GNRH Signaling,GPCR-Mediated Integration of Enteroendocrine Signaling Exemplified by an L Cell,GPCR-Mediated Nutrient Sensing in Enteroendocrine Cells,Gustation Pathway,Gαi Signaling,Gαs Signaling,Hepatic Cholestasis,IL-1 Signaling,Leptin Signaling in Obesity,Melanocyte Development and Pigmentation Signaling,Molecular Mechanisms of Cancer,P2Y Purigenic Receptor Signaling Pathway,Phospholipase C |

| | | | | | |
|---|---|---|---|---|---|
| | | | | | Signaling,PPARα/RXRα Activation,Protein Kinase A Signaling,RAR Activation,Relaxin Signaling,Renin-Angiotensin Signaling,Role of NFAT in Cardiac Hypertrophy,Serotonin Receptor Signaling,Sphingosine-1-phosphate Signaling,Thrombin Signaling,α-Adrenergic Signaling |
| MIMAT0001638 | miR-409-5p (and other miRNAs w/seed GGUUACC) | TargetScan Human | High (predicted) | ARHGEF6 | Actin Cytoskeleton Signaling,Agrin Interactions at Neuromuscular Junction,Axonal Guidance Signaling,Breast Cancer Regulation by Stathmin1,Cdc42 Signaling,FAK Signaling,G Beta Gamma Signaling,ILK Signaling,Molecular Mechanisms of Cancer,PAK Signaling,Paxillin Signaling,Phospholipase C Signaling,Reelin Signaling in Neurons,RhoGDI Signaling,Signaling by Rho Family GTPases,Thrombin Signaling |
| MIMAT0001638 | miR-409-5p (and other miRNAs w/seed GGUUACC) | TargetScan Human | Moderate (predicted) | ASH2L | Role of Oct4 in Mammalian Embryonic Stem Cell Pluripotency |
| MIMAT0001638 | miR-409-5p (and other miRNAs w/seed GGUUACC) | TargetScan Human | Moderate (predicted) | ASPH | Calcium Signaling |
| MIMAT0001638 | miR-409-5p (and other miRNAs w/seed GGUUACC) | TargetScan Human | Moderate (predicted) | CAMK1D | Breast Cancer Regulation by Stathmin1,Calcium Signaling,cAMP-mediated signaling,Chemokine Signaling,Glioma Signaling,Neuropathic Pain Signaling In Dorsal Horn Neurons,Role of NFAT in Cardiac Hypertrophy,Thrombin Signaling,Xenobiotic Metabolism Signaling |
| MIMAT0001638 | miR-409-5p (and other miRNAs w/seed GGUUACC) | TargetScan Human | Moderate (predicted) | CCL11 | Agranulocyte Adhesion and Diapedesis,Atherosclerosis Signaling,CCR3 Signaling in Eosinophils,Chemokine Signaling,Glucocorticoid Receptor Signaling,Granulocyte Adhesion and Diapedesis,IL-17 Signaling,IL-17A Signaling in Airway Cells |
| MIMAT0001638 | miR-409-5p (and other miRNAs w/seed GGUUACC) | TargetScan Human | Moderate (predicted) | DIRAS3 | Actin Nucleation by ARP-WASP Complex,Cardiac Hypertrophy Signaling,Cholecystokinin/Gastrin-mediated Signaling,Colorectal Cancer Metastasis Signaling,CXCR4 Signaling,Germ Cell-Sertoli Cell Junction Signaling,Glioblastoma Multiforme Signaling,Glioma Invasiveness Signaling,Gαq Signaling,HMGB1 Signaling,IL-8 Signaling,ILK Signaling,Integrin Signaling,Molecular Mechanisms of Cancer,mTOR Signaling,phagosome formation,Phospholipase C Signaling,Production of Nitric Oxide and Reactive Oxygen Species in Macrophages,Regulation of Actin-based Motility by Rho,RhoGDI Signaling,Semaphorin Signaling in Neurons,Signaling by Rho |

| | | | | | |
|---|---|---|---|---|---|
| | | | | | Family GTPases,Sphingosine-1-phosphate Signaling,Tec Kinase Signaling,Thrombin Signaling |
| MIMAT0001638 | miR-409-5p (and other miRNAs w/seed GGUUACC) | TargetScan Human | Moderate (predicted) | EFNB2 | Axonal Guidance Signaling,Ephrin B Signaling,Ephrin Receptor Signaling |
| MIMAT0001638 | miR-409-5p (and other miRNAs w/seed GGUUACC) | TargetScan Human | Moderate (predicted) | FRS2 | FGF Signaling,GDNF Family Ligand-Receptor Interactions,Neurotrophin/TRK Signaling,Regulation of the Epithelial-Mesenchymal Transition Pathway |
| MIMAT0001638 | miR-409-5p (and other miRNAs w/seed GGUUACC) | TargetScan Human | Moderate (predicted) | FZD3 | Adipogenesis pathway,Axonal Guidance Signaling,Basal Cell Carcinoma Signaling,Colorectal Cancer Metastasis Signaling,Factors Promoting Cardiogenesis in Vertebrates,Glioblastoma Multiforme Signaling,Human Embryonic Stem Cell Pluripotency,Molecular Mechanisms of Cancer,Mouse Embryonic Stem Cell Pluripotency,Ovarian Cancer Signaling,PCP pathway,Regulation of the Epithelial-Mesenchymal Transition Pathway,Role of Macrophages, Fibroblasts and Endothelial Cells in Rheumatoid Arthritis,Role of NANOG in Mammalian Embryonic Stem Cell Pluripotency,Role of Osteoblasts, Osteoclasts and Chondrocytes in Rheumatoid Arthritis,Role of Wnt/GSK-3β Signaling in the Pathogenesis of Influenza,Wnt/Ca+ pathway,Wnt/β-catenin Signaling |
| MIMAT0001638 | miR-409-5p (and other miRNAs w/seed GGUUACC) | TargetScan Human | High (predicted) | GNG12 | Actin Cytoskeleton Signaling,Androgen Signaling,Antiproliferative Role of Somatostatin Receptor 2,Axonal Guidance Signaling,Breast Cancer Regulation by Stathmin1,Cardiac Hypertrophy Signaling,Cardiac β-adrenergic Signaling,CCR3 Signaling in Eosinophils,CCR5 Signaling in Macrophages,Colorectal Cancer Metastasis Signaling,CREB Signaling in Neurons,CXCR4 Signaling,Ephrin B Signaling,Ephrin Receptor Signaling,fMLP Signaling in Neutrophils,G Beta Gamma Signaling,G Protein Signaling Mediated by Tubby,GPCR-Mediated Nutrient Sensing in Enteroendocrine Cells,Gαi Signaling,Gαq Signaling,Gαs Signaling,Huntington's Disease Signaling,IL-1 Signaling,IL-8 Signaling,P2Y Purigenic Receptor Signaling Pathway,Phospholipase C Signaling,Protein Kinase A Signaling,Relaxin Signaling,RhoGDI Signaling,Role of NFAT in Cardiac Hypertrophy,Role of NFAT in Regulation of the Immune Response,Signaling by Rho Family GTPases,Tec Kinase Signaling,Thrombin Signaling,α-Adrenergic Signaling |
| MIMAT0001638 | miR-409-5p (and | TargetScan Human | Moderate (predicted) | GNGT1 | Phototransduction Pathway |

| | | | | | |
|---|---|---|---|---|---|
| | other miRNAs w/seed GGUUACC) | | | | |
| MIMAT0001638 | miR-409-5p (and other miRNAs w/seed GGUUACC) | TargetScan Human | Moderate (predicted) | GTF3C2 | Assembly of RNA Polymerase III Complex |
| MIMAT0001638 | miR-409-5p (and other miRNAs w/seed GGUUACC) | TargetScan Human | High (predicted) | KIF7 | Axonal Guidance Signaling,Basal Cell Carcinoma Signaling |
| MIMAT0001638 | miR-409-5p (and other miRNAs w/seed GGUUACC) | TargetScan Human | High (predicted) | LRP8 | Reelin Signaling in Neurons |
| MIMAT0001638 | miR-409-5p (and other miRNAs w/seed GGUUACC) | TargetScan Human | Moderate (predicted) | MAP3K10 | B Cell Receptor Signaling,Cardiac Hypertrophy Signaling,CD27 Signaling in Lymphocytes,Germ Cell-Sertoli Cell Junction Signaling,GNRH Signaling,HGF Signaling,Huntington's Disease Signaling,NGF Signaling,PKCθ Signaling in T Lymphocytes,Production of Nitric Oxide and Reactive Oxygen Species in Macrophages,RANK Signaling in Osteoclasts,Reelin Signaling in Neurons,SAPK/JNK Signaling,Sertoli Cell-Sertoli Cell Junction Signaling,Signaling by Rho Family GTPases,STAT3 Pathway,Xenobiotic Metabolism Signaling |
| MIMAT0001638 | miR-409-5p (and other miRNAs w/seed GGUUACC) | TargetScan Human | High (predicted) | MARCKS | phagosome formation,Phospholipase C Signaling |
| MIMAT0001638 | miR-409-5p (and other miRNAs w/seed GGUUACC) | TargetScan Human | Moderate (predicted) | MC2R | cAMP-mediated signaling,G-Protein Coupled Receptor Signaling,Gαs Signaling |
| MIMAT0001638 | miR-409-5p (and other miRNAs w/seed | TargetScan Human | High (predicted) | MDM2 | Aryl Hydrocarbon Receptor Signaling,ATM Signaling,Bladder Cancer Signaling,Cell Cycle: G1/S Checkpoint Regulation,Cell Cycle: G2/M DNA Damage Checkpoint Regulation,Chronic Myeloid Leukemia Signaling,Clathrin-mediated Endocytosis Signaling,Glioblastoma Multiforme Signaling,Glioma |

| | | | | | |
|---|---|---|---|---|---|
| | GGUUACC) | | | | Signaling,HER-2 Signaling in Breast Cancer,HIF1α Signaling,Hypoxia Signaling in the Cardiovascular System,Melanoma Signaling,Molecular Mechanisms of Cancer,p53 Signaling,Pancreatic Adenocarcinoma Signaling,PI3K/AKT Signaling,Prostate Cancer Signaling,Protein Ubiquitination Pathway,Role of p14/p19ARF in Tumor Suppression,TR/RXR Activation,Wnt/β-catenin Signaling |
| MIMAT0001638 | miR-409-5p (and other miRNAs w/seed GGUUACC) | TargetScan Human | Moderate (predicted) | OPRL1 | cAMP-mediated signaling,G-Protein Coupled Receptor Signaling,Gαi Signaling |
| MIMAT0001638 | miR-409-5p (and other miRNAs w/seed GGUUACC) | TargetScan Human | Moderate (predicted) | PDE12 | cAMP-mediated signaling,Cardiac β-adrenergic Signaling,G-Protein Coupled Receptor Signaling,Gustation Pathway,Protein Kinase A Signaling,Relaxin Signaling,tRNA Splicing |
| MIMAT0001638 | miR-409-5p (and other miRNAs w/seed GGUUACC) | TargetScan Human | Moderate (predicted) | PDE6D | cAMP-mediated signaling,Cardiac β-adrenergic Signaling,G-Protein Coupled Receptor Signaling,Gustation Pathway,Phototransduction Pathway,Protein Kinase A Signaling,Relaxin Signaling,tRNA Splicing |
| MIMAT0001638 | miR-409-5p (and other miRNAs w/seed GGUUACC) | TargetScan Human | Moderate (predicted) | PFN2 | Actin Cytoskeleton Signaling,Axonal Guidance Signaling,PCP pathway,Regulation of Actin-based Motility by Rho,RhoA Signaling |
| MIMAT0001638 | miR-409-5p (and other miRNAs w/seed GGUUACC) | TargetScan Human | Moderate (predicted) | PGP | Protein Kinase A Signaling |
| MIMAT0001638 | miR-409-5p (and other miRNAs w/seed GGUUACC) | TargetScan Human | Moderate (predicted) | PLA2G3 | Antioxidant Action of Vitamin C,Atherosclerosis Signaling,CCR3 Signaling in Eosinophils,Eicosanoid Signaling,Endothelin-1 Signaling,ERK/MAPK Signaling,Fc Epsilon RI Signaling,MIF Regulation of Innate Immunity,MIF-mediated Glucocorticoid Regulation,p38 MAPK Signaling,Phospholipase C Signaling,Phospholipases,Role of MAPK Signaling in the Pathogenesis of Influenza,Sperm Motility,Synaptic Long Term Depression,VEGF Family Ligand-Receptor Interactions |
| MIMAT0001638 | miR-409-5p (and other miRNAs | TargetScan Human | Moderate (predicted) | POLR2J2/POLR2J | Androgen Signaling,Assembly of RNA Polymerase II Complex,CREB Signaling in Neurons,Estrogen Receptor Signaling,Glucocorticoid Receptor |

| | | | | | |
|---|---|---|---|---|---|
| | w/seed GGUUACC) | | | 3 | Signaling,Hereditary Breast Cancer Signaling,Huntington's Disease Signaling,Nucleotide Excision Repair Pathway |
| MIMAT0001638 | miR-409-5p (and other miRNAs w/seed GGUUACC) | TargetScan Human | High (predicted) | POLR2K | Androgen Signaling,Assembly of RNA Polymerase II Complex,CREB Signaling in Neurons,Estrogen Receptor Signaling,Glucocorticoid Receptor Signaling,Hereditary Breast Cancer Signaling,Huntington's Disease Signaling,Nucleotide Excision Repair Pathway |
| MIMAT0001638 | miR-409-5p (and other miRNAs w/seed GGUUACC) | TargetScan Human | Moderate (predicted) | PPARGC1A | AMPK Signaling,Estrogen Receptor Signaling,FXR/RXR Activation,LPS/IL-1 Mediated Inhibition of RXR Function,PPAR Signaling,PPARα/RXRα Activation,PXR/RXR Activation,RAR Activation,TR/RXR Activation,Xenobiotic Metabolism Signaling |
| MIMAT0001638 | miR-409-5p (and other miRNAs w/seed GGUUACC) | TargetScan Human | Moderate (predicted) | PPP2R3A | 3-phosphoinositide Biosynthesis,3-phosphoinositide Degradation,AMPK Signaling,Breast Cancer Regulation by Stathmin1,Cardiac β-adrenergic Signaling,CDK5 Signaling,Cell Cycle Regulation by BTG Family Proteins,Ceramide Signaling,CTLA4 Signaling in Cytotoxic T Lymphocytes,Cyclins and Cell Cycle Regulation,D-myo-inositol (1,4,5,6)-Tetrakisphosphate Biosynthesis,D-myo-inositol (3,4,5,6)-tetrakisphosphate Biosynthesis,D-myo-inositol-5-phosphate Metabolism,Dopamine Receptor Signaling,Dopamine-DARPP32 Feedback in cAMP Signaling,ERK/MAPK Signaling,HIPPO signaling,ILK Signaling,Mitotic Roles of Polo-Like Kinase,mTOR Signaling,p70S6K Signaling,PI3K/AKT Signaling,Production of Nitric Oxide and Reactive Oxygen Species in Macrophages,Regulation of eIF4 and p70S6K Signaling,Role of CHK Proteins in Cell Cycle Checkpoint Control,Superpathway of Inositol Phosphate Compounds,Synaptic Long Term Depression,Telomerase Signaling,Tight Junction Signaling,Wnt/β-catenin Signaling,Xenobiotic Metabolism Signaling |
| MIMAT0001638 | miR-409-5p (and other miRNAs w/seed GGUUACC) | TargetScan Human | High (predicted) | PRKAA2 | AMPK Signaling,eNOS Signaling,Glucocorticoid Receptor Signaling,mTOR Signaling,PPARα/RXRα Activation,Pyridoxal 5'-phosphate Salvage Pathway,Salvage Pathways of Pyrimidine Ribonucleotides,Type II Diabetes Mellitus Signaling |
| MIMAT0001638 | miR-409-5p (and other miRNAs w/seed GGUUACC) | TargetScan Human | Moderate (predicted) | PTGES3 | Aryl Hydrocarbon Receptor Signaling,Glucocorticoid Receptor Signaling,Prostanoid Biosynthesis,Telomerase Signaling,Xenobiotic Metabolism Signaling |
| MIMAT0001638 | miR-409-5p (and other miRNAs | TargetScan Human | High (predicted) | PTP4A1 | Protein Kinase A Signaling |

| | | | | | |
|---|---|---|---|---|---|
| | w/seed GGUUACC) | | | | |
| MIMAT0001638 | miR-409-5p (and other miRNAs w/seed GGUUACC) | TargetScan Human | Moderate (predicted) | RND3 | Actin Nucleation by ARP-WASP Complex,Cardiac Hypertrophy Signaling,Cholecystokinin/Gastrin-mediated Signaling,Colorectal Cancer Metastasis Signaling,CXCR4 Signaling,Germ Cell-Sertoli Cell Junction Signaling,Glioblastoma Multiforme Signaling,Glioma Invasiveness Signaling,Gαq Signaling,HMGB1 Signaling,IL-8 Signaling,ILK Signaling,Integrin Signaling,Molecular Mechanisms of Cancer,mTOR Signaling,phagosome formation,Phospholipase C Signaling,Production of Nitric Oxide and Reactive Oxygen Species in Macrophages,Regulation of Actin-based Motility by Rho,RhoA Signaling,RhoGDI Signaling,Semaphorin Signaling in Neurons,Signaling by Rho Family GTPases,Sphingosine-1-phosphate Signaling,Tec Kinase Signaling,Thrombin Signaling |
| MIMAT0001638 | miR-409-5p (and other miRNAs w/seed GGUUACC) | TargetScan Human | Moderate (predicted) | SEMA4D | Axonal Guidance Signaling,Semaphorin Signaling in Neurons |
| MIMAT0001638 | miR-409-5p (and other miRNAs w/seed GGUUACC) | TargetScan Human | Moderate (predicted) | SOCS4 | Acute Phase Response Signaling,Growth Hormone Signaling,IGF-1 Signaling,JAK/Stat Signaling,Prolactin Signaling,Role of JAK2 in Hormone-like Cytokine Signaling,STAT3 Pathway,Type I Diabetes Mellitus Signaling,Type II Diabetes Mellitus Signaling |
| MIMAT0001638 | miR-409-5p (and other miRNAs w/seed GGUUACC) | TargetScan Human | Moderate (predicted) | SYK | B Cell Receptor Signaling,CD28 Signaling in T Helper Cells,CTLA4 Signaling in Cytotoxic T Lymphocytes,Fc Epsilon RI Signaling,Fcγ Receptor-mediated Phagocytosis in Macrophages and Monocytes,FcγRIIB Signaling in B Lymphocytes,IL-15 Signaling,IL-2 Signaling,Natural Killer Cell Signaling,p70S6K Signaling,phagosome formation,Phospholipase C Signaling,PI3K Signaling in B Lymphocytes,Role of JAK1 and JAK3 in γc Cytokine Signaling,Role of NFAT in Regulation of the Immune Response,Role of Pattern Recognition Receptors in Recognition of Bacteria and Viruses |
| MIMAT0001638 | miR-409-5p (and other miRNAs w/seed GGUUACC) | TargetScan Human | Moderate (predicted) | VLDLR | FXR/RXR Activation,Reelin Signaling in Neurons |
| MIMAT0001638 | miR-409-5p (and other miRNAs | TargetScan Human | Moderate (predicted) | WNT2 | Axonal Guidance Signaling,Basal Cell Carcinoma Signaling,Colorectal Cancer Metastasis Signaling,Glioblastoma Multiforme Signaling,Human Embryonic |

| | | | | | |
|---|---|---|---|---|---|
| | w/seed GGUUACC) | | | | Stem Cell Pluripotency,Molecular Mechanisms of Cancer,Ovarian Cancer Signaling,PCP pathway,Regulation of the Epithelial-Mesenchymal Transition Pathway,Role of Macrophages, Fibroblasts and Endothelial Cells in Rheumatoid Arthritis,Role of NANOG in Mammalian Embryonic Stem Cell Pluripotency,Role of Osteoblasts, Osteoclasts and Chondrocytes in Rheumatoid Arthritis,Role of Wnt/GSK-3β Signaling in the Pathogenesis of Influenza,Wnt/β-catenin Signaling |
| MIMAT0004920 | miR-541-3p (and other miRNAs w/seed GGUGGGC) | TargetScan Human | High (predicted) | ACACA | AMPK Signaling,Biotin-carboxyl Carrier Protein Assembly,LXR/RXR Activation,TR/RXR Activation |
| MIMAT0004920 | miR-541-3p (and other miRNAs w/seed GGUGGGC) | TargetScan Human | High (predicted) | ACACB | AMPK Signaling,Biotin-carboxyl Carrier Protein Assembly |
| MIMAT0004920 | miR-541-3p (and other miRNAs w/seed GGUGGGC) | TargetScan Human | Moderate (predicted) | ACE | Axonal Guidance Signaling,Neuroprotective Role of THOP1 in Alzheimer's Disease,Renin-Angiotensin Signaling |
| MIMAT0004920 | miR-541-3p (and other miRNAs w/seed GGUGGGC) | TargetScan Human | High (predicted) | ADAMTS7 | Axonal Guidance Signaling |
| MIMAT0004920 | miR-541-3p (and other miRNAs w/seed GGUGGGC) | TargetScan Human | Moderate (predicted) | ADCY9 | Breast Cancer Regulation by Stathmin1,cAMP-mediated signaling,Cardiac Hypertrophy Signaling,Cardiac β-adrenergic Signaling,CDK5 Signaling,Cellular Effects of Sildenafil (Viagra),Colorectal Cancer Metastasis Signaling,Corticotropin Releasing Hormone Signaling,CREB Signaling in Neurons,CXCR4 Signaling,Dopamine Receptor Signaling,Dopamine-DARPP32 Feedback in cAMP Signaling,Endothelin-1 Signaling,eNOS Signaling,G-Protein Coupled Receptor Signaling,GABA Receptor Signaling,Gap Junction Signaling,GNRH Signaling,GPCR-Mediated Integration of Enteroendocrine Signaling Exemplified by an L Cell,GPCR-Mediated Nutrient Sensing in Enteroendocrine Cells,Gustation Pathway,Gαi Signaling,Gαs Signaling,Hepatic Cholestasis,IL-1 Signaling,Leptin Signaling in Obesity,Melanocyte Development and Pigmentation Signaling,Molecular Mechanisms of Cancer,P2Y Purigenic Receptor Signaling Pathway,Phospholipase C |

| | | | | | |
|---|---|---|---|---|---|
| | | | | | Signaling,PPARα/RXRα Activation,Protein Kinase A Signaling,RAR Activation,Relaxin Signaling,Renin-Angiotensin Signaling,Role of NFAT in Cardiac Hypertrophy,Serotonin Receptor Signaling,Sphingosine-1-phosphate Signaling,Thrombin Signaling,α-Adrenergic Signaling |
| MIMAT0004920 | miR-541-3p (and other miRNAs w/seed GGUGGGC) | TargetScan Human | Moderate (predicted) | ADD1 | Gαs Signaling,Protein Kinase A Signaling |
| MIMAT0004920 | miR-541-3p (and other miRNAs w/seed GGUGGGC) | TargetScan Human | Moderate (predicted) | AKAP13 | cAMP-mediated signaling,Cardiac β-adrenergic Signaling,Protein Kinase A Signaling |
| MIMAT0004920 | miR-541-3p (and other miRNAs w/seed GGUGGGC) | TargetScan Human | High (predicted) | AKT3 | 14-3-3-mediated Signaling,Acute Myeloid Leukemia Signaling,Acute Phase Response Signaling,AMPK Signaling,Amyloid Processing,Amyotrophic Lateral Sclerosis Signaling,Angiopoietin Signaling,Axonal Guidance Signaling,B Cell Receptor Signaling,CD28 Signaling in T Helper Cells,Ceramide Signaling,Chronic Myeloid Leukemia Signaling,Colorectal Cancer Metastasis Signaling,CREB Signaling in Neurons,CTLA4 Signaling in Cytotoxic T Lymphocytes,CXCR4 Signaling,Dendritic Cell Maturation,DNA damage-induced 14-3-3σ Signaling,Docosahexaenoic Acid (DHA) Signaling,EGF Signaling,EIF2 Signaling,Endometrial Cancer Signaling,eNOS Signaling,Ephrin Receptor Signaling,Epithelial Adherens Junction Signaling,Erythropoietin Signaling,Estrogen-Dependent Breast Cancer Signaling,FAK Signaling,Fc Epsilon RI Signaling,Fcγ Receptor-mediated Phagocytosis in Macrophages and Monocytes,FGF Signaling,FLT3 Signaling in Hematopoietic Progenitor Cells,FXR/RXR Activation,G Beta Gamma Signaling,G-Protein Coupled Receptor Signaling,Gap Junction Signaling,Glioblastoma Multiforme Signaling,Glioma Signaling,Glucocorticoid Receptor Signaling,GM-CSF Signaling,Gα12/13 Signaling,Gαq Signaling,HER-2 Signaling in Breast Cancer,Hereditary Breast Cancer Signaling,HGF Signaling,HIF1α Signaling,HMGB1 Signaling,Human Embryonic Stem Cell Pluripotency,Huntington's Disease Signaling,iCOS-iCOSL Signaling in T Helper Cells,IGF-1 Signaling,IL-12 Signaling and Production in Macrophages,IL-15 Signaling,IL-17 Signaling,IL-17A Signaling in Airway Cells,IL-2 Signaling,IL-22 Signaling,IL-3 Signaling,IL-4 Signaling,IL-6 Signaling,IL-8 Signaling,ILK Signaling,Inhibition of Angiogenesis by TSP1,Insulin Receptor Signaling,Integrin Signaling,JAK/Stat Signaling,Leptin Signaling in Obesity,Lymphotoxin β Receptor Signaling,Melanoma Signaling,Molecular |

| | | | | | |
|---|---|---|---|---|---|
| | | | | | Mechanisms of Cancer,Mouse Embryonic Stem Cell Pluripotency,mTOR Signaling,Myc Mediated Apoptosis Signaling,Natural Killer Cell Signaling,Neuregulin Signaling,NF-κB Activation by Viruses,NF-κB Signaling,NGF Signaling,Nitric Oxide Signaling in the Cardiovascular System,Non-Small Cell Lung Cancer Signaling,Ovarian Cancer Signaling,P2Y Purigenic Receptor Signaling Pathway,p53 Signaling,p70S6K Signaling,Pancreatic Adenocarcinoma Signaling,PEDF Signaling,PI3K Signaling in B Lymphocytes,PI3K/AKT Signaling,Production of Nitric Oxide and Reactive Oxygen Species in Macrophages,Prostate Cancer Signaling,PTEN Signaling,PXR/RXR Activation,RANK Signaling in Osteoclasts,RAR Activation,Regulation of eIF4 and p70S6K Signaling,Regulation of the Epithelial-Mesenchymal Transition Pathway,Relaxin Signaling,Renal Cell Carcinoma Signaling,Role of Macrophages, Fibroblasts and Endothelial Cells in Rheumatoid Arthritis,Role of MAPK Signaling in the Pathogenesis of Influenza,Role of NANOG in Mammalian Embryonic Stem Cell Pluripotency,Role of NFAT in Cardiac Hypertrophy,Role of NFAT in Regulation of the Immune Response,Role of Osteoblasts, Osteoclasts and Chondrocytes in Rheumatoid Arthritis,Role of PI3K/AKT Signaling in the Pathogenesis of Influenza,Role of Tissue Factor in Cancer,Sertoli Cell-Sertoli Cell Junction Signaling,Small Cell Lung Cancer Signaling,Sphingosine-1-phosphate Signaling,Systemic Lupus Erythematosus Signaling,Telomerase Signaling,Thrombin Signaling,Tight Junction Signaling,TR/RXR Activation,TREM1 Signaling,Type II Diabetes Mellitus Signaling,VEGF Family Ligand-Receptor Interactions,VEGF Signaling,Wnt/β-catenin Signaling |
| MIMAT0004920 | miR-541-3p (and other miRNAs w/seed GGUGGGC) | TargetScan Human | Moderate (predicted) | ALDH5A1 | 4-aminobutyrate Degradation I,Aryl Hydrocarbon Receptor Signaling,GABA Receptor Signaling,Glutamate Degradation III (via 4-aminobutyrate),LPS/IL-1 Mediated Inhibition of RXR Function,Xenobiotic Metabolism Signaling |
| MIMAT0004920 | miR-541-3p (and other miRNAs w/seed GGUGGGC) | TargetScan Human | Moderate (predicted) | ANAPC2 | Mitotic Roles of Polo-Like Kinase,Protein Kinase A Signaling,Protein Ubiquitination Pathway |
| MIMAT0004920 | miR-541-3p (and other miRNAs w/seed GGUGGGC) | TargetScan Human | Moderate (predicted) | AP2M1 | Clathrin-mediated Endocytosis Signaling,CTLA4 Signaling in Cytotoxic T Lymphocytes,GABA Receptor Signaling,Lipid Antigen Presentation by CD1,Virus Entry via Endocytic Pathways |

| | | | | | |
|---|---|---|---|---|---|
| MIMAT0004920 | miR-541-3p (and other miRNAs w/seed GGUGGGC) | TargetScan Human | High (predicted) | ARAF | Acute Myeloid Leukemia Signaling,Endothelin-1 Signaling,ERK/MAPK Signaling,IL-8 Signaling,Melatonin Signaling,NF-κB Signaling,Pyridoxal 5'-phosphate Salvage Pathway,Regulation of the Epithelial-Mesenchymal Transition Pathway,Salvage Pathways of Pyrimidine Ribonucleotides,UVC-Induced MAPK Signaling |
| MIMAT0004920 | miR-541-3p (and other miRNAs w/seed GGUGGGC) | TargetScan Human | Moderate (predicted) | ARHGDIA | Regulation of Actin-based Motility by Rho,RhoGDI Signaling |
| MIMAT0004920 | miR-541-3p (and other miRNAs w/seed GGUGGGC) | TargetScan Human | High (predicted) | ASIC1 | Aldosterone Signaling in Epithelial Cells,Gustation Pathway,Insulin Receptor Signaling |
| MIMAT0004920 | miR-541-3p (and other miRNAs w/seed GGUGGGC) | TargetScan Human | Moderate (predicted) | ATP2A3 | Calcium Signaling,Calcium Transport I,Calcium-induced T Lymphocyte Apoptosis,Cardiac β-adrenergic Signaling,Dopamine-DARPP32 Feedback in cAMP Signaling,Nitric Oxide Signaling in the Cardiovascular System |
| MIMAT0004920 | miR-541-3p (and other miRNAs w/seed GGUGGGC) | TargetScan Human | Moderate (predicted) | AVPR1B | G-Protein Coupled Receptor Signaling,Gαq Signaling |
| MIMAT0004920 | miR-541-3p (and other miRNAs w/seed GGUGGGC) | TargetScan Human | Moderate (predicted) | BCL2L1 | Amyotrophic Lateral Sclerosis Signaling,Apoptosis Signaling,B Cell Receptor Signaling,CD27 Signaling in Lymphocytes,Chronic Myeloid Leukemia Signaling,Colorectal Cancer Metastasis Signaling,Docosahexaenoic Acid (DHA) Signaling,Glucocorticoid Receptor Signaling,GM-CSF Signaling,Huntington's Disease Signaling,IL-15 Signaling,IL-8 Signaling,Induction of Apoptosis by HIV1,JAK/Stat Signaling,Lymphotoxin β Receptor Signaling,Molecular Mechanisms of Cancer,OX40 Signaling Pathway,p53 Signaling,Pancreatic Adenocarcinoma Signaling,PEDF Signaling,PI3K/AKT Signaling,PTEN Signaling,Role of Tissue Factor in Cancer,Small Cell Lung Cancer Signaling,UVA-Induced MAPK Signaling,VEGF Signaling |
| MIMAT0004920 | miR-541-3p (and other miRNAs w/seed GGUGGGC) | TargetScan Human | High (predicted) | BMP1 | Axonal Guidance Signaling,Basal Cell Carcinoma Signaling,BMP signaling pathway,Factors Promoting Cardiogenesis in Vertebrates,Human Embryonic Stem Cell Pluripotency,Molecular Mechanisms of Cancer,Role of NANOG in Mammalian Embryonic Stem Cell Pluripotency,Role of Osteoblasts, |

| | | | | | |
|---|---|---|---|---|---|
| | | | | | Osteoclasts and Chondrocytes in Rheumatoid Arthritis |
| MIMAT0004920 | miR-541-3p (and other miRNAs w/seed GGUGGGC) | TargetScan Human | Moderate (predicted) | BMP2 | Adipogenesis pathway,Axonal Guidance Signaling,Basal Cell Carcinoma Signaling,BMP signaling pathway,Cardiomyocyte Differentiation via BMP Receptors,Factors Promoting Cardiogenesis in Vertebrates,Human Embryonic Stem Cell Pluripotency,ILK Signaling,Molecular Mechanisms of Cancer,NF-κB Signaling,RAR Activation,Retinoate Biosynthesis I,Role of NANOG in Mammalian Embryonic Stem Cell Pluripotency,Role of Osteoblasts, Osteoclasts and Chondrocytes in Rheumatoid Arthritis,TGF-β Signaling |
| MIMAT0004920 | miR-541-3p (and other miRNAs w/seed GGUGGGC) | TargetScan Human | High (predicted) | BMP8A | Axonal Guidance Signaling,Basal Cell Carcinoma Signaling,BMP signaling pathway,Factors Promoting Cardiogenesis in Vertebrates,Human Embryonic Stem Cell Pluripotency,Molecular Mechanisms of Cancer,Role of NANOG in Mammalian Embryonic Stem Cell Pluripotency,Role of Osteoblasts, Osteoclasts and Chondrocytes in Rheumatoid Arthritis |
| MIMAT0004920 | miR-541-3p (and other miRNAs w/seed GGUGGGC) | TargetScan Human | Moderate (predicted) | BRF1 | Assembly of RNA Polymerase III Complex |
| MIMAT0004920 | miR-541-3p (and other miRNAs w/seed GGUGGGC) | TargetScan Human | Moderate (predicted) | CABIN1 | Calcium Signaling,Calcium-induced T Lymphocyte Apoptosis,Nur77 Signaling in T Lymphocytes,Role of NFAT in Cardiac Hypertrophy,Role of NFAT in Regulation of the Immune Response |
| MIMAT0004920 | miR-541-3p (and other miRNAs w/seed GGUGGGC) | TargetScan Human | Moderate (predicted) | CACNA1A | Amyotrophic Lateral Sclerosis Signaling,Cardiac Hypertrophy Signaling,Cardiac β-adrenergic Signaling,CDK5 Signaling,Cellular Effects of Sildenafil (Viagra),Dopamine-DARPP32 Feedback in cAMP Signaling,Maturity Onset Diabetes of Young (MODY) Signaling,Nitric Oxide Signaling in the Cardiovascular System |
| MIMAT0004920 | miR-541-3p (and other miRNAs w/seed GGUGGGC) | TargetScan Human | High (predicted) | CACNA1E | Amyotrophic Lateral Sclerosis Signaling,Cardiac Hypertrophy Signaling,Cardiac β-adrenergic Signaling,Cellular Effects of Sildenafil (Viagra),Dopamine-DARPP32 Feedback in cAMP Signaling,Maturity Onset Diabetes of Young (MODY) Signaling,Nitric Oxide Signaling in the Cardiovascular System |
| MIMAT0004920 | miR-541-3p (and other miRNAs w/seed GGUGGGC) | TargetScan Human | Moderate (predicted) | CACNA1S | Amyotrophic Lateral Sclerosis Signaling,Cardiac Hypertrophy Signaling,Cardiac β-adrenergic Signaling,Cellular Effects of Sildenafil (Viagra),Dopamine-DARPP32 Feedback in cAMP Signaling,Maturity Onset Diabetes of Young (MODY) Signaling,Nitric Oxide Signaling in the Cardiovascular System |

| | | | | | |
|---|---|---|---|---|---|
| MIMAT0004920 | miR-541-3p (and other miRNAs w/seed GGUGGGC) | TargetScan Human | Moderate (predicted) | CALM1 (includes others) | Androgen Signaling,B Cell Receptor Signaling,Breast Cancer Regulation by Stathmin1,Calcium Signaling,Calcium-induced T Lymphocyte Apoptosis,cAMP-mediated signaling,Cardiac Hypertrophy Signaling,CCR3 Signaling in Eosinophils,CCR5 Signaling in Macrophages,CD28 Signaling in T Helper Cells,Cellular Effects of Sildenafil (Viagra),Chemokine Signaling,Corticotropin Releasing Hormone Signaling,CREB Signaling in Neurons,Dopamine-DARPP32 Feedback in cAMP Signaling,eNOS Signaling,fMLP Signaling in Neutrophils,Glioma Signaling,Glutamate Receptor Signaling,Gαq Signaling,iCOS-iCOSL Signaling in T Helper Cells,iNOS Signaling,Melatonin Signaling,Nitric Oxide Signaling in the Cardiovascular System,nNOS Signaling in Neurons,nNOS Signaling in Skeletal Muscle Cells,Nur77 Signaling in T Lymphocytes,Phospholipase C Signaling,PI3K Signaling in B Lymphocytes,Protein Kinase A Signaling,RANK Signaling in Osteoclasts,Regulation of IL-2 Expression in Activated and Anergic T Lymphocytes,Role of Macrophages, Fibroblasts and Endothelial Cells in Rheumatoid Arthritis,Role of NFAT in Cardiac Hypertrophy,Role of NFAT in Regulation of the Immune Response,Role of Osteoblasts, Osteoclasts and Chondrocytes in Rheumatoid Arthritis,Sperm Motility,Synaptic Long Term Potentiation,T Cell Receptor Signaling,α-Adrenergic Signaling |
| MIMAT0004920 | miR-541-3p (and other miRNAs w/seed GGUGGGC) | TargetScan Human | High (predicted) | CAMK1G | Breast Cancer Regulation by Stathmin1,Calcium Signaling,cAMP-mediated signaling,Chemokine Signaling,Glioma Signaling,Neuropathic Pain Signaling In Dorsal Horn Neurons,Role of NFAT in Cardiac Hypertrophy,Thrombin Signaling,Xenobiotic Metabolism Signaling |
| MIMAT0004920 | miR-541-3p (and other miRNAs w/seed GGUGGGC) | TargetScan Human | High (predicted) | CAMK2A | B Cell Receptor Signaling,Breast Cancer Regulation by Stathmin1,Calcium Signaling,cAMP-mediated signaling,Chemokine Signaling,CREB Signaling in Neurons,Crosstalk between Dendritic Cells and Natural Killer Cells,G-Protein Coupled Receptor Signaling,Glioma Signaling,GM-CSF Signaling,GNRH Signaling,iCOS-iCOSL Signaling in T Helper Cells,Melatonin Signaling,Molecular Mechanisms of Cancer,Neuropathic Pain Signaling In Dorsal Horn Neurons,nNOS Signaling in Neurons,PI3K Signaling in B Lymphocytes,PKCθ Signaling in T Lymphocytes,Protein Kinase A Signaling,Role of Macrophages, Fibroblasts and Endothelial Cells in Rheumatoid Arthritis,Role of NFAT in Cardiac Hypertrophy,Synaptic Long Term Potentiation,Thrombin Signaling,Wnt/Ca+ pathway,Xenobiotic Metabolism Signaling |
| MIMAT0004920 | miR-541-3p (and other miRNAs w/seed | TargetScan Human | Moderate (predicted) | CAMKK1 | Calcium Signaling,Dopamine-DARPP32 Feedback in cAMP Signaling |

| | | | | | |
|---|---|---|---|---|---|
| | GGUGGGC) | | | | |
| MIMAT0004920 | miR-541-3p (and other miRNAs w/seed GGUGGGC) | TargetScan Human | Moderate (predicted) | CAPN5 | Amyloid Processing,Amyotrophic Lateral Sclerosis Signaling,Apoptosis Signaling,FAK Signaling,Huntington's Disease Signaling,Integrin Signaling,nNOS Signaling in Neurons,Regulation of Cellular Mechanics by Calpain Protease |
| MIMAT0004920 | miR-541-3p (and other miRNAs w/seed GGUGGGC) | TargetScan Human | Moderate (predicted) | CCNF | Role of Oct4 in Mammalian Embryonic Stem Cell Pluripotency |
| MIMAT0004920 | miR-541-3p (and other miRNAs w/seed GGUGGGC) | TargetScan Human | Moderate (predicted) | CD79B | Altered T Cell and B Cell Signaling in Rheumatoid Arthritis,B Cell Development,B Cell Receptor Signaling,FcγRIIB Signaling in B Lymphocytes,p70S6K Signaling,Phospholipase C Signaling,PI3K Signaling in B Lymphocytes,Role of NFAT in Regulation of the Immune Response,Systemic Lupus Erythematosus Signaling |
| MIMAT0004920 | miR-541-3p (and other miRNAs w/seed GGUGGGC) | TargetScan Human | High (predicted) | CHMP1A | Axonal Guidance Signaling |
| MIMAT0004920 | miR-541-3p (and other miRNAs w/seed GGUGGGC) | TargetScan Human | Moderate (predicted) | CHRM2 | cAMP-mediated signaling,G-Protein Coupled Receptor Signaling,GPCR-Mediated Integration of Enteroendocrine Signaling Exemplified by an L Cell,Gαi Signaling |
| MIMAT0004920 | miR-541-3p (and other miRNAs w/seed GGUGGGC) | TargetScan Human | Moderate (predicted) | CHRNA9 | AMPK Signaling,Calcium Signaling,eNOS Signaling |
| MIMAT0004920 | miR-541-3p (and other miRNAs w/seed GGUGGGC) | TargetScan Human | Moderate (predicted) | CHRNE | AMPK Signaling,Calcium Signaling,eNOS Signaling |
| MIMAT0004920 | miR-541-3p (and other miRNAs w/seed | TargetScan Human | Moderate (predicted) | CISH | GM-CSF Signaling,IL-9 Signaling,JAK/Stat Signaling,STAT3 Pathway |

| | | | | | |
|---|---|---|---|---|---|
| | GGUGGGC) | | | | |
| MIMAT0004920 | miR-541-3p (and other miRNAs w/seed GGUGGGC) | TargetScan Human | Moderate (predicted) | CKM | AMPK Signaling,Creatine-phosphate Biosynthesis |
| MIMAT0004920 | miR-541-3p (and other miRNAs w/seed GGUGGGC) | TargetScan Human | Moderate (predicted) | CNR2 | cAMP-mediated signaling,G-Protein Coupled Receptor Signaling,Gαi Signaling,Reelin Signaling in Neurons |
| MIMAT0004920 | miR-541-3p (and other miRNAs w/seed GGUGGGC) | TargetScan Human | Moderate (predicted) | COMT | Dopamine Degradation,Dopamine Receptor Signaling,L-DOPA Degradation,Noradrenaline and Adrenaline Degradation |
| MIMAT0004920 | miR-541-3p (and other miRNAs w/seed GGUGGGC) | TargetScan Human | Moderate (predicted) | CSK | Actin Cytoskeleton Signaling,B Cell Receptor Signaling,CD28 Signaling in T Helper Cells,FAK Signaling,Gαq Signaling,iCOS-iCOSL Signaling in T Helper Cells,Paxillin Signaling,RAR Activation,T Cell Receptor Signaling |
| MIMAT0004920 | miR-541-3p (and other miRNAs w/seed GGUGGGC) | TargetScan Human | Moderate (predicted) | DAG1 | Agrin Interactions at Neuromuscular Junction,nNOS Signaling in Skeletal Muscle Cells |
| MIMAT0004920 | miR-541-3p (and other miRNAs w/seed GGUGGGC) | TargetScan Human | Moderate (predicted) | DCX | Reelin Signaling in Neurons |
| MIMAT0004920 | miR-541-3p (and other miRNAs w/seed GGUGGGC) | TargetScan Human | Moderate (predicted) | DDR1 | Dendritic Cell Maturation,NF-κB Signaling,PTEN Signaling,STAT3 Pathway |
| MIMAT0004920 | miR-541-3p (and other miRNAs w/seed GGUGGGC) | TargetScan Human | High (predicted) | DOK3 | GDNF Family Ligand-Receptor Interactions |

| | | | | | |
|---|---|---|---|---|---|
| MIMAT0004920 | miR-541-3p (and other miRNAs w/seed GGUGGGC) | TargetScan Human | Moderate (predicted) | DPYSL2 | Axonal Guidance Signaling,Semaphorin Signaling in Neurons,Thymine Degradation,Uracil Degradation II (Reductive) |
| MIMAT0004920 | miR-541-3p (and other miRNAs w/seed GGUGGGC) | TargetScan Human | Moderate (predicted) | DUSP3 | Protein Kinase A Signaling |
| MIMAT0004920 | miR-541-3p (and other miRNAs w/seed GGUGGGC) | TargetScan Human | Moderate (predicted) | EFNA1 | Axonal Guidance Signaling,Ephrin A Signaling,Ephrin Receptor Signaling |
| MIMAT0004920 | miR-541-3p (and other miRNAs w/seed GGUGGGC) | TargetScan Human | Moderate (predicted) | EFNA5 | Axonal Guidance Signaling,Ephrin A Signaling,Ephrin Receptor Signaling |
| MIMAT0004920 | miR-541-3p (and other miRNAs w/seed GGUGGGC) | TargetScan Human | High (predicted) | EFNB1 | Axonal Guidance Signaling,Ephrin B Signaling,Ephrin Receptor Signaling,PCP pathway |
| MIMAT0004920 | miR-541-3p (and other miRNAs w/seed GGUGGGC) | TargetScan Human | Moderate (predicted) | EFNB3 | Axonal Guidance Signaling,Ephrin B Signaling,Ephrin Receptor Signaling |
| MIMAT0004920 | miR-541-3p (and other miRNAs w/seed GGUGGGC) | TargetScan Human | Moderate (predicted) | EGFR | Agrin Interactions at Neuromuscular Junction,Bladder Cancer Signaling,Caveolar-mediated Endocytosis Signaling,Cholecystokinin/Gastrin-mediated Signaling,Colorectal Cancer Metastasis Signaling,EGF Signaling,Epithelial Adherens Junction Signaling,ErbB Signaling,ERK5 Signaling,Estrogen-Dependent Breast Cancer Signaling,FAK Signaling,G Beta Gamma Signaling,Gap Junction Signaling,Glioblastoma Multiforme Signaling,Glioma Signaling,GNRH Signaling,Hepatic Fibrosis / Hepatic Stellate Cell Activation,HER-2 Signaling in Breast Cancer,Huntington's Disease Signaling,IL-17A Signaling in Gastric Cells,IL-8 Signaling,Neuregulin Signaling,NF-κB Signaling,Non-Small Cell Lung Cancer Signaling,Ovarian Cancer Signaling,p70S6K Signaling,Pancreatic Adenocarcinoma Signaling,PTEN |

| | | | | | |
|---|---|---|---|---|---|
| | | | | | Signaling,Regulation of Cellular Mechanics by Calpain Protease,Regulation of the Epithelial-Mesenchymal Transition Pathway,Role of Tissue Factor in Cancer,STAT3 Pathway,Telomerase Signaling,Thrombin Signaling,UVA-Induced MAPK Signaling,UVB-Induced MAPK Signaling,UVC-Induced MAPK Signaling |
| MIMAT0004920 | miR-541-3p (and other miRNAs w/seed GGUGGGC) | TargetScan Human | Moderate (predicted) | EIF2B3 | Cardiac Hypertrophy Signaling,EIF2 Signaling,Insulin Receptor Signaling,Regulation of eIF4 and p70S6K Signaling,VEGF Signaling |
| MIMAT0004920 | miR-541-3p (and other miRNAs w/seed GGUGGGC) | TargetScan Human | Moderate (predicted) | EPHA5 | Axonal Guidance Signaling,Ephrin A Signaling,Ephrin Receptor Signaling |
| MIMAT0004920 | miR-541-3p (and other miRNAs w/seed GGUGGGC) | TargetScan Human | High (predicted) | EPHA8 | Axonal Guidance Signaling,Ephrin A Signaling,Ephrin Receptor Signaling |
| MIMAT0004920 | miR-541-3p (and other miRNAs w/seed GGUGGGC) | TargetScan Human | Moderate (predicted) | EYA3 | Protein Kinase A Signaling |
| MIMAT0004920 | miR-541-3p (and other miRNAs w/seed GGUGGGC) | TargetScan Human | Moderate (predicted) | FGFR1 | Adipogenesis pathway,Epithelial Adherens Junction Signaling,FGF Signaling,Hepatic Fibrosis / Hepatic Stellate Cell Activation,Human Embryonic Stem Cell Pluripotency,NF-κB Signaling,PTEN Signaling,Regulation of the Epithelial-Mesenchymal Transition Pathway,STAT3 Pathway |
| MIMAT0004920 | miR-541-3p (and other miRNAs w/seed GGUGGGC) | TargetScan Human | Moderate (predicted) | FGFR3 | Adipogenesis pathway,Bladder Cancer Signaling,FGF Signaling,Human Embryonic Stem Cell Pluripotency,NF-κB Signaling,PTEN Signaling,Regulation of the Epithelial-Mesenchymal Transition Pathway,STAT3 Pathway |
| MIMAT0004920 | miR-541-3p (and other miRNAs w/seed GGUGGGC) | TargetScan Human | Moderate (predicted) | FLNB | Caveolar-mediated Endocytosis Signaling,ILK Signaling,Protein Kinase A Signaling,Virus Entry via Endocytic Pathways |

| | | | | | |
|---|---|---|---|---|---|
| MIMAT0004920 | miR-541-3p (and other miRNAs w/seed GGUGGGC) | TargetScan Human | High (predicted) | FLT4 | eNOS Signaling,Hepatic Fibrosis / Hepatic Stellate Cell Activation,IL-8 Signaling,NF-κB Signaling,Nitric Oxide Signaling in the Cardiovascular System,PTEN Signaling,STAT3 Pathway,VEGF Family Ligand-Receptor Interactions,VEGF Signaling |
| MIMAT0004920 | miR-541-3p (and other miRNAs w/seed GGUGGGC) | TargetScan Human | Moderate (predicted) | FNBP1 | Actin Nucleation by ARP-WASP Complex,Cardiac Hypertrophy Signaling,Cholecystokinin/Gastrin-mediated Signaling,Colorectal Cancer Metastasis Signaling,CXCR4 Signaling,Germ Cell-Sertoli Cell Junction Signaling,Glioblastoma Multiforme Signaling,Glioma Invasiveness Signaling,Gαq Signaling,HMGB1 Signaling,IL-8 Signaling,ILK Signaling,Integrin Signaling,Molecular Mechanisms of Cancer,mTOR Signaling,phagosome formation,Phospholipase C Signaling,Production of Nitric Oxide and Reactive Oxygen Species in Macrophages,Regulation of Actin-based Motility by Rho,RhoGDI Signaling,Semaphorin Signaling in Neurons,Signaling by Rho Family GTPases,Sphingosine-1-phosphate Signaling,Tec Kinase Signaling,Thrombin Signaling |
| MIMAT0004920 | miR-541-3p (and other miRNAs w/seed GGUGGGC) | TargetScan Human | Moderate (predicted) | GABRB3 | GABA Receptor Signaling |
| MIMAT0004920 | miR-541-3p (and other miRNAs w/seed GGUGGGC) | TargetScan Human | Moderate (predicted) | GABRE | GABA Receptor Signaling |
| MIMAT0004920 | miR-541-3p (and other miRNAs w/seed GGUGGGC) | TargetScan Human | Moderate (predicted) | GABRP | GABA Receptor Signaling |
| MIMAT0004920 | miR-541-3p (and other miRNAs w/seed GGUGGGC) | TargetScan Human | High (predicted) | GATA4 | Cardiac Hypertrophy Signaling,Cardiomyocyte Differentiation via BMP Receptors,Embryonic Stem Cell Differentiation into Cardiac Lineages,Factors Promoting Cardiogenesis in Vertebrates,Role of NANOG in Mammalian Embryonic Stem Cell Pluripotency,Role of NFAT in Cardiac Hypertrophy,Role of NFAT in Regulation of the Immune Response,Thrombin Signaling,Transcriptional Regulatory Network in Embryonic Stem Cells |
| MIMAT0004920 | miR-541-3p (and other miRNAs | TargetScan Human | High (predicted) | GH1 | Cholecystokinin/Gastrin-mediated Signaling,Growth Hormone Signaling,NF-κB Signaling,PPARα/RXRα Activation,Role of JAK2 in Hormone-like Cytokine |

| | | | | | |
|---|---|---|---|---|---|
| | w/seed GGUGGGC) | | | | Signaling,TR/RXR Activation |
| MIMAT0004920 | miR-541-3p (and other miRNAs w/seed GGUGGGC) | TargetScan Human | High (predicted) | GIT1 | Actin Cytoskeleton Signaling,Axonal Guidance Signaling,Integrin Signaling,PAK Signaling |
| MIMAT0004920 | miR-541-3p (and other miRNAs w/seed GGUGGGC) | TargetScan Human | Moderate (predicted) | GLI3 | Axonal Guidance Signaling,Basal Cell Carcinoma Signaling,Corticotropin Releasing Hormone Signaling,Protein Kinase A Signaling,Sonic Hedgehog Signaling |
| MIMAT0004920 | miR-541-3p (and other miRNAs w/seed GGUGGGC) | TargetScan Human | Moderate (predicted) | GLIS1 | Axonal Guidance Signaling,Basal Cell Carcinoma Signaling,Sonic Hedgehog Signaling |
| MIMAT0004920 | miR-541-3p (and other miRNAs w/seed GGUGGGC) | TargetScan Human | Moderate (predicted) | GLIS2 | Axonal Guidance Signaling,Basal Cell Carcinoma Signaling,Sonic Hedgehog Signaling |
| MIMAT0004920 | miR-541-3p (and other miRNAs w/seed GGUGGGC) | TargetScan Human | Moderate (predicted) | GNAO1 | Androgen Signaling,Axonal Guidance Signaling,cAMP-mediated signaling,Cardiac Hypertrophy Signaling,Corticotropin Releasing Hormone Signaling,CREB Signaling in Neurons,CXCR4 Signaling,Endothelin-1 Signaling,Ephrin B Signaling,Ephrin Receptor Signaling,G Beta Gamma Signaling,G-Protein Coupled Receptor Signaling,IL-1 Signaling,Melatonin Signaling,Molecular Mechanisms of Cancer,Relaxin Signaling,RhoGDI Signaling,Role of Macrophages, Fibroblasts and Endothelial Cells in Rheumatoid Arthritis,Role of NFAT in Regulation of the Immune Response,Signaling by Rho Family GTPases,Synaptic Long Term Depression,Tec Kinase Signaling,Thrombin Signaling,Wnt/β-catenin Signaling |
| MIMAT0004920 | miR-541-3p (and other miRNAs w/seed GGUGGGC) | TargetScan Human | Moderate (predicted) | GNAS | AMPK Signaling,Androgen Signaling,Axonal Guidance Signaling,Breast Cancer Regulation by Stathmin1,cAMP-mediated signaling,Cardiac Hypertrophy Signaling,Cardiac β-adrenergic Signaling,CCR3 Signaling in Eosinophils,CCR5 Signaling in Macrophages,CDK5 Signaling,Cellular Effects of Sildenafil (Viagra),Colorectal Cancer Metastasis Signaling,Corticotropin Releasing Hormone Signaling,CREB Signaling in Neurons,CXCR4 Signaling,Dopamine Receptor Signaling,Dopamine-DARPP32 Feedback in cAMP |

| | | | | | |
|---|---|---|---|---|---|
| | | | | | Signaling,Endothelin-1 Signaling,eNOS Signaling,Ephrin B Signaling,Ephrin Receptor Signaling,fMLP Signaling in Neutrophils,G Beta Gamma Signaling,G Protein Signaling Mediated by Tubby,G-Protein Coupled Receptor Signaling,GABA Receptor Signaling,Gap Junction Signaling,GNRH Signaling,GPCR-Mediated Integration of Enteroendocrine Signaling Exemplified by an L Cell,GPCR-Mediated Nutrient Sensing in Enteroendocrine Cells,Gustation Pathway,Gαi Signaling,Gαq Signaling,Gαs Signaling,Hepatic Cholestasis,Human Embryonic Stem Cell Pluripotency,IL-1 Signaling,IL-8 Signaling,Leptin Signaling in Obesity,Melanocyte Development and Pigmentation Signaling,Molecular Mechanisms of Cancer,P2Y Purigenic Receptor Signaling Pathway,Phospholipase C Signaling,PPARα/RXRα Activation,Protein Kinase A Signaling,RAR Activation,Relaxin Signaling,Renin-Angiotensin Signaling,RhoGDI Signaling,Role of NFAT in Cardiac Hypertrophy,Role of NFAT in Regulation of the Immune Response,Serotonin Receptor Signaling,Signaling by Rho Family GTPases,Sperm Motility,Sphingosine-1-phosphate Signaling,Synaptic Long Term Depression,Tec Kinase Signaling,Thrombin Signaling,α-Adrenergic Signaling |
| MIMAT0004920 | miR-541-3p (and other miRNAs w/seed GGUGGGC) | TargetScan Human | Moderate (predicted) | GNB5 | Androgen Signaling,Antiproliferative Role of Somatostatin Receptor 2,Axonal Guidance Signaling,Breast Cancer Regulation by Stathmin1,Cardiac Hypertrophy Signaling,Cardiac β-adrenergic Signaling,CCR3 Signaling in Eosinophils,CCR5 Signaling in Macrophages,Colorectal Cancer Metastasis Signaling,CREB Signaling in Neurons,CXCR4 Signaling,Ephrin B Signaling,Ephrin Receptor Signaling,fMLP Signaling in Neutrophils,G Beta Gamma Signaling,G Protein Signaling Mediated by Tubby,Gαi Signaling,Gαq Signaling,Gαs Signaling,Huntington's Disease Signaling,IL-1 Signaling,IL-8 Signaling,P2Y Purigenic Receptor Signaling Pathway,Phospholipase C Signaling,Phototransduction Pathway,Protein Kinase A Signaling,Relaxin Signaling,RhoGDI Signaling,Role of NFAT in Cardiac Hypertrophy,Role of NFAT in Regulation of the Immune Response,Signaling by Rho Family GTPases,Tec Kinase Signaling,Thrombin Signaling,α-Adrenergic Signaling |
| MIMAT0004920 | miR-541-3p (and other miRNAs w/seed GGUGGGC) | TargetScan Human | High (predicted) | GNG13 | Androgen Signaling,Antiproliferative Role of Somatostatin Receptor 2,Axonal Guidance Signaling,Breast Cancer Regulation by Stathmin1,Cardiac Hypertrophy Signaling,Cardiac β-adrenergic Signaling,CCR3 Signaling in Eosinophils,CCR5 Signaling in Macrophages,Colorectal Cancer Metastasis Signaling,CREB Signaling in Neurons,CXCR4 Signaling,Ephrin B Signaling,Ephrin Receptor Signaling,fMLP Signaling in Neutrophils,G Beta Gamma Signaling,G Protein Signaling Mediated by Tubby,GPCR-Mediated Nutrient Sensing in Enteroendocrine Cells,Gustation Pathway,Gαi Signaling,Gαq Signaling,Gαs Signaling,Huntington's Disease Signaling,IL-1 Signaling,IL-8 Signaling,P2Y |

| | | | | | |
|---|---|---|---|---|---|
| | | | | | Purigenic Receptor Signaling Pathway,Phospholipase C Signaling,Protein Kinase A Signaling,Relaxin Signaling,RhoGDI Signaling,Role of NFAT in Cardiac Hypertrophy,Role of NFAT in Regulation of the Immune Response,Signaling by Rho Family GTPases,Tec Kinase Signaling,Thrombin Signaling,α-Adrenergic Signaling |
| MIMAT0004920 | miR-541-3p (and other miRNAs w/seed GGUGGGC) | TargetScan Human | Moderate (predicted) | GNG3 | Androgen Signaling,Antiproliferative Role of Somatostatin Receptor 2,Axonal Guidance Signaling,Breast Cancer Regulation by Stathmin1,Cardiac Hypertrophy Signaling,Cardiac β-adrenergic Signaling,CCR3 Signaling in Eosinophils,CCR5 Signaling in Macrophages,Colorectal Cancer Metastasis Signaling,CREB Signaling in Neurons,CXCR4 Signaling,Ephrin B Signaling,Ephrin Receptor Signaling,fMLP Signaling in Neutrophils,G Beta Gamma Signaling,G Protein Signaling Mediated by Tubby,GPCR-Mediated Nutrient Sensing in Enteroendocrine Cells,Gαi Signaling,Gαq Signaling,Gαs Signaling,Huntington's Disease Signaling,IL-1 Signaling,IL-8 Signaling,P2Y Purigenic Receptor Signaling Pathway,Phospholipase C Signaling,Protein Kinase A Signaling,Relaxin Signaling,RhoGDI Signaling,Role of NFAT in Cardiac Hypertrophy,Role of NFAT in Regulation of the Immune Response,Signaling by Rho Family GTPases,Tec Kinase Signaling,Thrombin Signaling,α-Adrenergic Signaling |
| MIMAT0004920 | miR-541-3p (and other miRNAs w/seed GGUGGGC) | TargetScan Human | Moderate (predicted) | GPLD1 | Antioxidant Action of Vitamin C,Choline Biosynthesis III,Endothelin-1 Signaling,Fcγ Receptor-mediated Phagocytosis in Macrophages and Monocytes,Gαq Signaling,IL-8 Signaling,mTOR Signaling,Pancreatic Adenocarcinoma Signaling,Phospholipase C Signaling,Phospholipases |
| MIMAT0004920 | miR-541-3p (and other miRNAs w/seed GGUGGGC) | TargetScan Human | High (predicted) | GPR17 | cAMP-mediated signaling,G-Protein Coupled Receptor Signaling,Gαi Signaling |
| MIMAT0004920 | miR-541-3p (and other miRNAs w/seed GGUGGGC) | TargetScan Human | Moderate (predicted) | GRB2 | 14-3-3-mediated Signaling,Actin Cytoskeleton Signaling,Actin Nucleation by ARP-WASP Complex,Acute Myeloid Leukemia Signaling,Acute Phase Response Signaling,Angiopoietin Signaling,Axonal Guidance Signaling,B Cell Receptor Signaling,BMP signaling pathway,Breast Cancer Regulation by Stathmin1,Cardiac Hypertrophy Signaling,CD28 Signaling in T Helper Cells,Cholecystokinin/Gastrin-mediated Signaling,Chronic Myeloid Leukemia Signaling,Clathrin-mediated Endocytosis Signaling,CNTF Signaling,Colorectal Cancer Metastasis Signaling,CREB Signaling in Neurons,CTLA4 Signaling in Cytotoxic T Lymphocytes,EGF Signaling,EIF2 Signaling,Endometrial Cancer Signaling,Endothelin-1 Signaling,Ephrin Receptor Signaling,ErbB Signaling,ErbB2-ErbB3 Signaling,ErbB4 Signaling,ERK/MAPK |

| | | | | | |
|---|---|---|---|---|---|
| | | | | | Signaling,Erythropoietin Signaling,Estrogen Receptor Signaling,FAK Signaling,Fc Epsilon RI Signaling,FcγRIIB Signaling in B Lymphocytes,FGF Signaling,FLT3 Signaling in Hematopoietic Progenitor Cells,G Beta Gamma Signaling,G-Protein Coupled Receptor Signaling,Gap Junction Signaling,GDNF Family Ligand-Receptor Interactions,Glioblastoma Multiforme Signaling,Glioma Signaling,Glucocorticoid Receptor Signaling,GM-CSF Signaling,GNRH Signaling,Gαi Signaling,HER-2 Signaling in Breast Cancer,HGF Signaling,Huntington's Disease Signaling,iCOS-iCOSL Signaling in T Helper Cells,IGF-1 Signaling,IL-2 Signaling,IL-3 Signaling,IL-4 Signaling,IL-6 Signaling,Insulin Receptor Signaling,Integrin Signaling,JAK/Stat Signaling,Leptin Signaling in Obesity,Melanocyte Development and Pigmentation Signaling,Molecular Mechanisms of Cancer,Mouse Embryonic Stem Cell Pluripotency,Myc Mediated Apoptosis Signaling,Natural Killer Cell Signaling,Neuregulin Signaling,Neurotrophin/TRK Signaling,NGF Signaling,Non-Small Cell Lung Cancer Signaling,Oncostatin M Signaling,p70S6K Signaling,PAK Signaling,Pancreatic Adenocarcinoma Signaling,Paxillin Signaling,PDGF Signaling,Phospholipase C Signaling,PI3K/AKT Signaling,PKCθ Signaling in T Lymphocytes,PPAR Signaling,PPARα/RXRα Activation,Prolactin Signaling,Prostate Cancer Signaling,PTEN Signaling,Regulation of Cellular Mechanics by Calpain Protease,Regulation of eIF4 and p70S6K Signaling,Regulation of IL-2 Expression in Activated and Anergic T Lymphocytes,Regulation of the Epithelial-Mesenchymal Transition Pathway,Renal Cell Carcinoma Signaling,Renin-Angiotensin Signaling,Role of JAK1 and JAK3 in γc Cytokine Signaling,Role of NANOG in Mammalian Embryonic Stem Cell Pluripotency,Role of NFAT in Cardiac Hypertrophy,Role of NFAT in Regulation of the Immune Response,SAPK/JNK Signaling,Systemic Lupus Erythematosus Signaling,T Cell Receptor Signaling,Telomerase Signaling,TGF-β Signaling,Thrombin Signaling,Thrombopoietin Signaling,TREM1 Signaling,VEGF Family Ligand-Receptor Interactions,VEGF Signaling |
| MIMAT0004920 | miR-541-3p (and other miRNAs w/seed GGUGGGC) | TargetScan Human | Moderate (predicted) | GRIA1 | Amyotrophic Lateral Sclerosis Signaling,Calcium Signaling,CREB Signaling in Neurons,Glutamate Receptor Signaling,Neuropathic Pain Signaling In Dorsal Horn Neurons,Synaptic Long Term Depression,Synaptic Long Term Potentiation |
| MIMAT0004920 | miR-541-3p (and other miRNAs w/seed GGUGGGC) | TargetScan Human | Moderate (predicted) | GRID1 | Amyotrophic Lateral Sclerosis Signaling,CREB Signaling in Neurons,Glutamate Receptor Signaling,Synaptic Long Term Depression |

| | | | | | |
|---|---|---|---|---|---|
| MIMAT0004920 | miR-541-3p (and other miRNAs w/seed GGUGGGC) | TargetScan Human | Moderate (predicted) | GRIK3 | Amyotrophic Lateral Sclerosis Signaling,CREB Signaling in Neurons,Glutamate Receptor Signaling |
| MIMAT0004920 | miR-541-3p (and other miRNAs w/seed GGUGGGC) | TargetScan Human | High (predicted) | GRIN1 | Amyotrophic Lateral Sclerosis Signaling,Calcium Signaling,Circadian Rhythm Signaling,CREB Signaling in Neurons,Dopamine-DARPP32 Feedback in cAMP Signaling,Ephrin Receptor Signaling,Glutamate Receptor Signaling,Neuropathic Pain Signaling In Dorsal Horn Neurons,nNOS Signaling in Neurons,Synaptic Long Term Potentiation |
| MIMAT0004920 | miR-541-3p (and other miRNAs w/seed GGUGGGC) | TargetScan Human | High (predicted) | GRIN2A | Amyotrophic Lateral Sclerosis Signaling,Calcium Signaling,Circadian Rhythm Signaling,CREB Signaling in Neurons,Dopamine-DARPP32 Feedback in cAMP Signaling,Ephrin Receptor Signaling,Glutamate Receptor Signaling,Neuropathic Pain Signaling In Dorsal Horn Neurons,nNOS Signaling in Neurons,Synaptic Long Term Potentiation |
| MIMAT0004920 | miR-541-3p (and other miRNAs w/seed GGUGGGC) | TargetScan Human | Moderate (predicted) | GRIN2D | Amyotrophic Lateral Sclerosis Signaling,Calcium Signaling,Circadian Rhythm Signaling,CREB Signaling in Neurons,Dopamine-DARPP32 Feedback in cAMP Signaling,Ephrin Receptor Signaling,Glutamate Receptor Signaling,Neuropathic Pain Signaling In Dorsal Horn Neurons,nNOS Signaling in Neurons,Synaptic Long Term Potentiation |
| MIMAT0004920 | miR-541-3p (and other miRNAs w/seed GGUGGGC) | TargetScan Human | Moderate (predicted) | GTF2E1 | Androgen Signaling,Assembly of RNA Polymerase II Complex,Estrogen Receptor Signaling,Glucocorticoid Receptor Signaling |
| MIMAT0004920 | miR-541-3p (and other miRNAs w/seed GGUGGGC) | TargetScan Human | High (predicted) | GUCA2B | Phototransduction Pathway |
| MIMAT0004920 | miR-541-3p (and other miRNAs w/seed GGUGGGC) | TargetScan Human | High (predicted) | HDAC7 | Adipogenesis pathway,Calcium Signaling,Cell Cycle: G1/S Checkpoint Regulation,Chronic Myeloid Leukemia Signaling,Cyclins and Cell Cycle Regulation,Hereditary Breast Cancer Signaling,Huntington's Disease Signaling,Phospholipase C Signaling,Role of NFAT in Cardiac Hypertrophy,Telomerase Signaling |
| MIMAT0004920 | miR-541-3p (and other miRNAs w/seed | TargetScan Human | Moderate (predicted) | HKR1 | Axonal Guidance Signaling,Basal Cell Carcinoma Signaling,Sonic Hedgehog Signaling |

| | | | | | |
|---|---|---|---|---|---|
| | GGUGGGC) | | | | |
| MIMAT0004920 | miR-541-3p (and other miRNAs w/seed GGUGGGC) | TargetScan Human | High (predicted) | HRH2 | cAMP-mediated signaling,G-Protein Coupled Receptor Signaling,Granulocyte Adhesion and Diapedesis,Gαs Signaling |
| MIMAT0004920 | miR-541-3p (and other miRNAs w/seed GGUGGGC) | TargetScan Human | High (predicted) | HRH3 | cAMP-mediated signaling,G-Protein Coupled Receptor Signaling,Granulocyte Adhesion and Diapedesis,Gαi Signaling |
| MIMAT0004920 | miR-541-3p (and other miRNAs w/seed GGUGGGC) | TargetScan Human | Moderate (predicted) | HSPB1 | Aldosterone Signaling in Epithelial Cells,Aryl Hydrocarbon Receptor Signaling,Cardiac Hypertrophy Signaling,Death Receptor Signaling,ERK/MAPK Signaling,Granulocyte Adhesion and Diapedesis,IL-6 Signaling,p38 MAPK Signaling,PCP pathway,Protein Ubiquitination Pathway |
| MIMAT0004920 | miR-541-3p (and other miRNAs w/seed GGUGGGC) | TargetScan Human | High (predicted) | HSPB2 | Aldosterone Signaling in Epithelial Cells,Aryl Hydrocarbon Receptor Signaling,Death Receptor Signaling,ERK/MAPK Signaling,IL-6 Signaling,p38 MAPK Signaling,Protein Ubiquitination Pathway |
| MIMAT0004920 | miR-541-3p (and other miRNAs w/seed GGUGGGC) | TargetScan Human | Moderate (predicted) | HSPB7 | Aldosterone Signaling in Epithelial Cells,Aryl Hydrocarbon Receptor Signaling,Death Receptor Signaling,ERK/MAPK Signaling,IL-6 Signaling,p38 MAPK Signaling,Protein Ubiquitination Pathway |
| MIMAT0004920 | miR-541-3p (and other miRNAs w/seed GGUGGGC) | TargetScan Human | Moderate (predicted) | HTR7 | cAMP-mediated signaling,G-Protein Coupled Receptor Signaling,Gαs Signaling,Serotonin Receptor Signaling |
| MIMAT0004920 | miR-541-3p (and other miRNAs w/seed GGUGGGC) | TargetScan Human | Moderate (predicted) | IGF1 | Amyotrophic Lateral Sclerosis Signaling,Axonal Guidance Signaling,Cardiac Hypertrophy Signaling,Clathrin-mediated Endocytosis Signaling,Estrogen-Dependent Breast Cancer Signaling,Glioblastoma Multiforme Signaling,Glioma Signaling,Growth Hormone Signaling,Hepatic Fibrosis / Hepatic Stellate Cell Activation,Huntington's Disease Signaling,IGF-1 Signaling,Myc Mediated Apoptosis Signaling,RhoA Signaling,Role of IL-17F in Allergic Inflammatory Airway Diseases,Role of NFAT in Cardiac Hypertrophy,Role of Osteoblasts, Osteoclasts and Chondrocytes in Rheumatoid Arthritis,Synaptic Long Term Depression |

| | | | | | |
|---|---|---|---|---|---|
| MIMAT0004920 | miR-541-3p (and other miRNAs w/seed GGUGGGC) | TargetScan Human | Moderate (predicted) | IGF2BP1 | Role of Oct4 in Mammalian Embryonic Stem Cell Pluripotency |
| MIMAT0004920 | miR-541-3p (and other miRNAs w/seed GGUGGGC) | TargetScan Human | Moderate (predicted) | IL36B | Acute Phase Response Signaling,Agranulocyte Adhesion and Diapedesis,Altered T Cell and B Cell Signaling in Rheumatoid Arthritis,Atherosclerosis Signaling,Cholecystokinin/Gastrin-mediated Signaling,Communication between Innate and Adaptive Immune Cells,Dendritic Cell Maturation,FXR/RXR Activation,Graft-versus-Host Disease Signaling,Granulocyte Adhesion and Diapedesis,Hepatic Cholestasis,IL-10 Signaling,IL-6 Signaling,LPS/IL-1 Mediated Inhibition of RXR Function,LXR/RXR Activation,NF-κB Signaling,p38 MAPK Signaling,PPAR Signaling,Role of Cytokines in Mediating Communication between Immune Cells,Role of Hypercytokinemia/hyperchemokinemia in the Pathogenesis of Influenza,Role of Macrophages, Fibroblasts and Endothelial Cells in Rheumatoid Arthritis,Role of Osteoblasts, Osteoclasts and Chondrocytes in Rheumatoid Arthritis,Systemic Lupus Erythematosus Signaling,Toll-like Receptor Signaling |
| MIMAT0004920 | miR-541-3p (and other miRNAs w/seed GGUGGGC) | TargetScan Human | High (predicted) | INPP5B | 1D-myo-inositol Hexakisphosphate Biosynthesis II (Mammalian),3-phosphoinositide Degradation,B Cell Receptor Signaling,D-myo-inositol (1,3,4)-trisphosphate Biosynthesis,D-myo-inositol (1,4,5)-trisphosphate Degradation,Fc Epsilon RI Signaling,IL-4 Signaling,Insulin Receptor Signaling,Natural Killer Cell Signaling,PDGF Signaling,PI3K/AKT Signaling,PTEN Signaling,Superpathway of D-myo-inositol (1,4,5)-trisphosphate Metabolism,Superpathway of Inositol Phosphate Compounds |
| MIMAT0004920 | miR-541-3p (and other miRNAs w/seed GGUGGGC) | TargetScan Human | High (predicted) | IRS1 | AMPK Signaling,Cardiac Hypertrophy Signaling,GDNF Family Ligand-Receptor Interactions,Growth Hormone Signaling,IGF-1 Signaling,IL-4 Signaling,IL-9 Signaling,ILK Signaling,Insulin Receptor Signaling,Molecular Mechanisms of Cancer,mTOR Signaling,p70S6K Signaling,PI3K Signaling in B Lymphocytes,PPARα/RXRα Activation,Prolactin Signaling,Regulation of eIF4 and p70S6K Signaling,Role of JAK1 and JAK3 in γc Cytokine Signaling,Role of JAK2 in Hormone-like Cytokine Signaling,SAPK/JNK Signaling,Type II Diabetes Mellitus Signaling |
| MIMAT0004920 | miR-541-3p (and other miRNAs w/seed GGUGGGC) | TargetScan Human | Moderate (predicted) | ITGA3 | Actin Cytoskeleton Signaling,Actin Nucleation by ARP-WASP Complex,Agranulocyte Adhesion and Diapedesis,Agrin Interactions at Neuromuscular Junction,Axonal Guidance Signaling,Caveolar-mediated Endocytosis Signaling,Cdc42 Signaling,CDK5 Signaling,Ephrin Receptor Signaling,ERK/MAPK Signaling,FAK Signaling,Germ Cell-Sertoli Cell Junction |

| | | | | | |
|---|---|---|---|---|---|
| | | | | | Signaling,Granulocyte Adhesion and Diapedesis,HGF Signaling,Integrin Signaling,Leukocyte Extravasation Signaling,Molecular Mechanisms of Cancer,Neuregulin Signaling,NF-κB Activation by Viruses,PAK Signaling,Paxillin Signaling,phagosome formation,Phospholipase C Signaling,PI3K/AKT Signaling,PTEN Signaling,Rac Signaling,Reelin Signaling in Neurons,Regulation of Actin-based Motility by Rho,Regulation of Cellular Mechanics by Calpain Protease,Regulation of eIF4 and p70S6K Signaling,RhoGDI Signaling,Role of Osteoblasts, Osteoclasts and Chondrocytes in Rheumatoid Arthritis,Role of Tissue Factor in Cancer,Sertoli Cell-Sertoli Cell Junction Signaling,Signaling by Rho Family GTPases,Tec Kinase Signaling,Virus Entry via Endocytic Pathways |
| MIMAT0004920 | miR-541-3p (and other miRNAs w/seed GGUGGGC) | TargetScan Human | Moderate (predicted) | KCNJ10 | Dopamine-DARPP32 Feedback in cAMP Signaling |
| MIMAT0004920 | miR-541-3p (and other miRNAs w/seed GGUGGGC) | TargetScan Human | Moderate (predicted) | KCNJ11 | Dopamine-DARPP32 Feedback in cAMP Signaling,Type II Diabetes Mellitus Signaling |
| MIMAT0004920 | miR-541-3p (and other miRNAs w/seed GGUGGGC) | TargetScan Human | Moderate (predicted) | KDM5B | Role of Oct4 in Mammalian Embryonic Stem Cell Pluripotency |
| MIMAT0004920 | miR-541-3p (and other miRNAs w/seed GGUGGGC) | TargetScan Human | Moderate (predicted) | KIF7 | Axonal Guidance Signaling,Basal Cell Carcinoma Signaling |
| MIMAT0004920 | miR-541-3p (and other miRNAs w/seed GGUGGGC) | TargetScan Human | Moderate (predicted) | KLK3 | Human Embryonic Stem Cell Pluripotency,Intrinsic Prothrombin Activation Pathway,MSP-RON Signaling Pathway,Neurotrophin/TRK Signaling,Prostate Cancer Signaling,Thyroid Cancer Signaling |
| MIMAT0004920 | miR-541-3p (and other miRNAs w/seed GGUGGGC) | TargetScan Human | Moderate (predicted) | L1CAM | Axonal Guidance Signaling,Transcriptional Regulatory Network in Embryonic Stem Cells |

| | | | | | |
|---|---|---|---|---|---|
| MIMAT0004920 | miR-541-3p (and other miRNAs w/seed GGUGGGC) | TargetScan Human | Moderate (predicted) | LCK | Calcium-induced T Lymphocyte Apoptosis,CD28 Signaling in T Helper Cells,CTLA4 Signaling in Cytotoxic T Lymphocytes,G Protein Signaling Mediated by Tubby,iCOS-iCOSL Signaling in T Helper Cells,IL-15 Signaling,IL-2 Signaling,Natural Killer Cell Signaling,NF-κB Activation by Viruses,NF-κB Signaling,Phospholipase C Signaling,PKCθ Signaling in T Lymphocytes,Primary Immunodeficiency Signaling,Reelin Signaling in Neurons,Role of NFAT in Regulation of the Immune Response,Role of Tissue Factor in Cancer,SAPK/JNK Signaling,Systemic Lupus Erythematosus Signaling,T Cell Receptor Signaling,Tec Kinase Signaling |
| MIMAT0004920 | miR-541-3p (and other miRNAs w/seed GGUGGGC) | TargetScan Human | Moderate (predicted) | LHX5 | Transcriptional Regulatory Network in Embryonic Stem Cells |
| MIMAT0004920 | miR-541-3p (and other miRNAs w/seed GGUGGGC) | TargetScan Human | Moderate (predicted) | LIF | ERK5 Signaling,Hematopoiesis from Pluripotent Stem Cells,Hepatic Cholestasis,HMGB1 Signaling,Mouse Embryonic Stem Cell Pluripotency,Role of NANOG in Mammalian Embryonic Stem Cell Pluripotency,Role of NFAT in Cardiac Hypertrophy,Role of Pattern Recognition Receptors in Recognition of Bacteria and Viruses |
| MIMAT0004920 | miR-541-3p (and other miRNAs w/seed GGUGGGC) | TargetScan Human | Moderate (predicted) | LINGO1 | Axonal Guidance Signaling |
| MIMAT0004920 | miR-541-3p (and other miRNAs w/seed GGUGGGC) | TargetScan Human | Moderate (predicted) | LPAR5 | eNOS Signaling,GPCR-Mediated Nutrient Sensing in Enteroendocrine Cells,Gα12/13 Signaling,RhoA Signaling |
| MIMAT0004920 | miR-541-3p (and other miRNAs w/seed GGUGGGC) | TargetScan Human | Moderate (predicted) | LRP8 | Reelin Signaling in Neurons |
| MIMAT0004920 | miR-541-3p (and other miRNAs w/seed GGUGGGC) | TargetScan Human | Moderate (predicted) | MAP2K2 | 14-3-3-mediated Signaling,4-1BB Signaling in T Lymphocytes,Actin Cytoskeleton Signaling,Acute Myeloid Leukemia Signaling,Acute Phase Response Signaling,Aldosterone Signaling in Epithelial Cells,Antiproliferative Role of Somatostatin Receptor 2,Apoptosis Signaling,Axonal Guidance Signaling,B Cell Receptor Signaling,Bladder Cancer Signaling,BMP signaling |

| | | | | | |
|---|---|---|---|---|---|
| | | | | | pathway,Breast Cancer Regulation by Stathmin1,cAMP-mediated signaling,Cardiac Hypertrophy Signaling,CCR3 Signaling in Eosinophils,CD27 Signaling in Lymphocytes,CD28 Signaling in T Helper Cells,CD40 Signaling,CDK5 Signaling,Chemokine Signaling,Cholecystokinin/Gastrin-mediated Signaling,Chronic Myeloid Leukemia Signaling,CNTF Signaling,Colorectal Cancer Metastasis Signaling,Corticotropin Releasing Hormone Signaling,CREB Signaling in Neurons,CXCR4 Signaling,EIF2 Signaling,Endometrial Cancer Signaling,Ephrin Receptor Signaling,ErbB Signaling,ErbB2-ErbB3 Signaling,ErbB4 Signaling,ERK/MAPK Signaling,Erythropoietin Signaling,Estrogen Receptor Signaling,FAK Signaling,Fc Epsilon RI Signaling,FLT3 Signaling in Hematopoietic Progenitor Cells,fMLP Signaling in Neutrophils,G-Protein Coupled Receptor Signaling,Gap Junction Signaling,GDNF Family Ligand-Receptor Interactions,Germ Cell-Sertoli Cell Junction Signaling,Glioblastoma Multiforme Signaling,Glioma Signaling,Glucocorticoid Receptor Signaling,GM-CSF Signaling,GNRH Signaling,Gα12/13 Signaling,Gαq Signaling,Gαs Signaling,HGF Signaling,HMGB1 Signaling,IGF-1 Signaling,IL-12 Signaling and Production in Macrophages,IL-15 Signaling,IL-17 Signaling,IL-17A Signaling in Airway Cells,IL-2 Signaling,IL-3 Signaling,IL-6 Signaling,IL-8 Signaling,Insulin Receptor Signaling,Integrin Signaling,JAK/Stat Signaling,Leptin Signaling in Obesity,Leukocyte Extravasation Signaling,LPS-stimulated MAPK Signaling,Melanocyte Development and Pigmentation Signaling,Melanoma Signaling,Melatonin Signaling,Molecular Mechanisms of Cancer,Mouse Embryonic Stem Cell Pluripotency,Natural Killer Cell Signaling,Neuregulin Signaling,Neurotrophin/TRK Signaling,NGF Signaling,Nitric Oxide Signaling in the Cardiovascular System,Non-Small Cell Lung Cancer Signaling,NRF2-mediated Oxidative Stress Response,Oncostatin M Signaling,Ovarian Cancer Signaling,P2Y Purigenic Receptor Signaling Pathway,p70S6K Signaling,PAK Signaling,Pancreatic Adenocarcinoma Signaling,PDGF Signaling,Phospholipase C Signaling,PI3K Signaling in B Lymphocytes,PI3K/AKT Signaling,PPAR Signaling,PPARα/RXRα Activation,Prolactin Signaling,Prostate Cancer Signaling,Protein Kinase A Signaling,PTEN Signaling,Pyridoxal 5'-phosphate Salvage Pathway,Rac Signaling,RANK Signaling in Osteoclasts,Regulation of eIF4 and p70S6K Signaling,Regulation of IL-2 Expression in Activated and Anergic T Lymphocytes,Regulation of the Epithelial-Mesenchymal Transition Pathway,Renal Cell Carcinoma Signaling,Renin-Angiotensin Signaling,Role of IL-17A in Arthritis,Role of IL-17F in Allergic Inflammatory Airway Diseases,Role of Macrophages, Fibroblasts and Endothelial Cells in Rheumatoid Arthritis,Role of MAPK Signaling in the Pathogenesis of Influenza,Role of NANOG in Mammalian Embryonic Stem Cell Pluripotency,Role of NFAT in Cardiac Hypertrophy,Role of NFAT in Regulation |

| | | | | | |
|---|---|---|---|---|---|
| | | | | | of the Immune Response,Role of PI3K/AKT Signaling in the Pathogenesis of Influenza,Salvage Pathways of Pyrimidine Ribonucleotides,Sertoli Cell-Sertoli Cell Junction Signaling,Signaling by Rho Family GTPases,STAT3 Pathway,Synaptic Long Term Depression,Synaptic Long Term Potentiation,T Cell Receptor Signaling,Telomerase Signaling,TGF-β Signaling,Thrombin Signaling,Thrombopoietin Signaling,Thyroid Cancer Signaling,UVC-Induced MAPK Signaling,VEGF Family Ligand-Receptor Interactions,VEGF Signaling,Xenobiotic Metabolism Signaling,α-Adrenergic Signaling |
| MIMAT0004920 | miR-541-3p (and other miRNAs w/seed GGUGGGC) | TargetScan Human | High (predicted) | MAP2K7 | Acute Myeloid Leukemia Signaling,Acute Phase Response Signaling,Apoptosis Signaling,April Mediated Signaling,B Cell Activating Factor Signaling,B Cell Receptor Signaling,Cardiac Hypertrophy Signaling,CD27 Signaling in Lymphocytes,CD40 Signaling,Death Receptor Signaling,EGF Signaling,Fc Epsilon RI Signaling,Germ Cell-Sertoli Cell Junction Signaling,Glucocorticoid Receptor Signaling,GNRH Signaling,Gα12/13 Signaling,HGF Signaling,HMGB1 Signaling,Huntington's Disease Signaling,IL-1 Signaling,IL-6 Signaling,Induction of Apoptosis by HIV1,LPS/IL-1 Mediated Inhibition of RXR Function,Melatonin Signaling,Neurotrophin/TRK Signaling,NF-κB Signaling,NRF2-mediated Oxidative Stress Response,PPARα/RXRα Activation,Production of Nitric Oxide and Reactive Oxygen Species in Macrophages,Rac Signaling,RANK Signaling in Osteoclasts,Reelin Signaling in Neurons,Regulation of IL-2 Expression in Activated and Anergic T Lymphocytes,Regulation of the Epithelial-Mesenchymal Transition Pathway,Role of Macrophages, Fibroblasts and Endothelial Cells in Rheumatoid Arthritis,Role of MAPK Signaling in the Pathogenesis of Influenza,Role of NFAT in Cardiac Hypertrophy,Role of Osteoblasts, Osteoclasts and Chondrocytes in Rheumatoid Arthritis,SAPK/JNK Signaling,Sertoli Cell-Sertoli Cell Junction Signaling,Signaling by Rho Family GTPases,Type I Diabetes Mellitus Signaling,Type II Diabetes Mellitus Signaling,Unfolded protein response,Xenobiotic Metabolism Signaling |
| MIMAT0004920 | miR-541-3p (and other miRNAs w/seed GGUGGGC) | TargetScan Human | High (predicted) | MAP3K1 | Acute Phase Response Signaling,April Mediated Signaling,B Cell Activating Factor Signaling,B Cell Receptor Signaling,Cardiac Hypertrophy Signaling,CD27 Signaling in Lymphocytes,CD28 Signaling in T Helper Cells,Ceramide Signaling,EGF Signaling,FGF Signaling,Germ Cell-Sertoli Cell Junction Signaling,Glucocorticoid Receptor Signaling,GNRH Signaling,Gα12/13 Signaling,HGF Signaling,IL-1 Signaling,LPS/IL-1 Mediated Inhibition of RXR Function,NF-κB Activation by Viruses,NF-κB Signaling,NGF Signaling,NRF2-mediated Oxidative Stress Response,PDGF Signaling,PKCθ Signaling in T Lymphocytes,Production of Nitric Oxide and Reactive Oxygen Species in Macrophages,Protein Kinase A Signaling,Rac Signaling,RANK Signaling in Osteoclasts,RAR Activation,Regulation of IL-2 Expression in Activated and |

| | | | | | |
|---|---|---|---|---|---|
| | | | | | Anergic T Lymphocytes,Renin-Angiotensin Signaling,Role of NFAT in Cardiac Hypertrophy,SAPK/JNK Signaling,Sertoli Cell-Sertoli Cell Junction Signaling,T Cell Receptor Signaling,TNFR1 Signaling,TNFR2 Signaling,Toll-like Receptor Signaling,Type II Diabetes Mellitus Signaling,Xenobiotic Metabolism Signaling |
| MIMAT0004920 | miR-541-3p (and other miRNAs w/seed GGUGGGC) | TargetScan Human | Moderate (predicted) | MAP3K10 | B Cell Receptor Signaling,Cardiac Hypertrophy Signaling,CD27 Signaling in Lymphocytes,Germ Cell-Sertoli Cell Junction Signaling,GNRH Signaling,HGF Signaling,Huntington's Disease Signaling,NGF Signaling,PKCθ Signaling in T Lymphocytes,Production of Nitric Oxide and Reactive Oxygen Species in Macrophages,RANK Signaling in Osteoclasts,Reelin Signaling in Neurons,SAPK/JNK Signaling,Sertoli Cell-Sertoli Cell Junction Signaling,Signaling by Rho Family GTPases,STAT3 Pathway,Xenobiotic Metabolism Signaling |
| MIMAT0004920 | miR-541-3p (and other miRNAs w/seed GGUGGGC) | TargetScan Human | Moderate (predicted) | MAP3K12 | B Cell Receptor Signaling,Cardiac Hypertrophy Signaling,CD27 Signaling in Lymphocytes,Germ Cell-Sertoli Cell Junction Signaling,GNRH Signaling,HGF Signaling,NGF Signaling,PKCθ Signaling in T Lymphocytes,Production of Nitric Oxide and Reactive Oxygen Species in Macrophages,RANK Signaling in Osteoclasts,SAPK/JNK Signaling,Sertoli Cell-Sertoli Cell Junction Signaling,Signaling by Rho Family GTPases,STAT3 Pathway,Xenobiotic Metabolism Signaling |
| MIMAT0004920 | miR-541-3p (and other miRNAs w/seed GGUGGGC) | TargetScan Human | Moderate (predicted) | MAP3K9 | B Cell Receptor Signaling,Cardiac Hypertrophy Signaling,CD27 Signaling in Lymphocytes,Germ Cell-Sertoli Cell Junction Signaling,GNRH Signaling,HGF Signaling,NGF Signaling,PKCθ Signaling in T Lymphocytes,Production of Nitric Oxide and Reactive Oxygen Species in Macrophages,Pyridoxal 5'-phosphate Salvage Pathway,RANK Signaling in Osteoclasts,Reelin Signaling in Neurons,Salvage Pathways of Pyrimidine Ribonucleotides,SAPK/JNK Signaling,Sertoli Cell-Sertoli Cell Junction Signaling,Signaling by Rho Family GTPases,STAT3 Pathway,Xenobiotic Metabolism Signaling |
| MIMAT0004920 | miR-541-3p (and other miRNAs w/seed GGUGGGC) | TargetScan Human | Moderate (predicted) | MAPK11 | 4-1BB Signaling in T Lymphocytes,Acute Phase Response Signaling,AMPK Signaling,Amyloid Processing,Antioxidant Action of Vitamin C,April Mediated Signaling,ATM Signaling,B Cell Activating Factor Signaling,B Cell Receptor Signaling,BMP signaling pathway,Cardiac Hypertrophy Signaling,CCR3 Signaling in Eosinophils,CCR5 Signaling in Macrophages,CD40 Signaling,Cdc42 Signaling,CDK5 Signaling,Chemokine Signaling,Corticotropin Releasing Hormone Signaling,Dendritic Cell Maturation,EGF Signaling,Endothelin-1 Signaling,ErbB Signaling,Fc Epsilon RI Signaling,FGF Signaling,FLT3 Signaling in Hematopoietic Progenitor Cells,Glucocorticoid Receptor Signaling,GNRH Signaling,HIF1α Signaling,HMGB1 Signaling,IL-1 Signaling,IL-10 Signaling,IL-12 |

| | | | | | |
|---|---|---|---|---|---|
| | | | | | Signaling and Production in Macrophages,IL-15 Signaling,IL-17 Signaling,IL-17A Signaling in Airway Cells,IL-17A Signaling in Fibroblasts,IL-17A Signaling in Gastric Cells,IL-22 Signaling,IL-6 Signaling,Inhibition of Angiogenesis by TSP1,iNOS Signaling,Leukocyte Extravasation Signaling,LPS-stimulated MAPK Signaling,Molecular Mechanisms of Cancer,Mouse Embryonic Stem Cell Pluripotency,p38 MAPK Signaling,Parkinson's Signaling,Paxillin Signaling,PEDF Signaling,Production of Nitric Oxide and Reactive Oxygen Species in Macrophages,RANK Signaling in Osteoclasts,RAR Activation,Regulation of eIF4 and p70S6K Signaling,Renin-Angiotensin Signaling,Role of IL-17A in Arthritis,Role of JAK family kinases in IL-6-type Cytokine Signaling,Role of MAPK Signaling in the Pathogenesis of Influenza,Role of NFAT in Cardiac Hypertrophy,Role of Tissue Factor in Cancer,Sertoli Cell-Sertoli Cell Junction Signaling,STAT3 Pathway,TGF-β Signaling,Thrombin Signaling,Toll-like Receptor Signaling,Type I Diabetes Mellitus Signaling,UVA-Induced MAPK Signaling,UVB-Induced MAPK Signaling,UVC-Induced MAPK Signaling,Xenobiotic Metabolism Signaling |
| MIMAT0004920 | miR-541-3p (and other miRNAs w/seed GGUGGGC) | TargetScan Human | Moderate (predicted) | MAPK13 | 4-1BB Signaling in T Lymphocytes,Acute Phase Response Signaling,AMPK Signaling,Amyloid Processing,Antioxidant Action of Vitamin C,April Mediated Signaling,ATM Signaling,B Cell Activating Factor Signaling,B Cell Receptor Signaling,BMP signaling pathway,Cardiac Hypertrophy Signaling,CCR3 Signaling in Eosinophils,CCR5 Signaling in Macrophages,CD40 Signaling,Cdc42 Signaling,CDK5 Signaling,Chemokine Signaling,Corticotropin Releasing Hormone Signaling,Dendritic Cell Maturation,EGF Signaling,Endothelin-1 Signaling,ErbB Signaling,Fc Epsilon RI Signaling,FGF Signaling,FLT3 Signaling in Hematopoietic Progenitor Cells,Glucocorticoid Receptor Signaling,GNRH Signaling,HIF1α Signaling,HMGB1 Signaling,IL-1 Signaling,IL-10 Signaling,IL-12 Signaling and Production in Macrophages,IL-15 Signaling,IL-17 Signaling,IL-17A Signaling in Airway Cells,IL-17A Signaling in Fibroblasts,IL-17A Signaling in Gastric Cells,IL-22 Signaling,IL-6 Signaling,Inhibition of Angiogenesis by TSP1,iNOS Signaling,Leukocyte Extravasation Signaling,LPS-stimulated MAPK Signaling,Molecular Mechanisms of Cancer,Mouse Embryonic Stem Cell Pluripotency,p38 MAPK Signaling,Parkinson's Signaling,Paxillin Signaling,PEDF Signaling,Production of Nitric Oxide and Reactive Oxygen Species in Macrophages,RANK Signaling in Osteoclasts,RAR Activation,Regulation of eIF4 and p70S6K Signaling,Renin-Angiotensin Signaling,Role of IL-17A in Arthritis,Role of JAK family kinases in IL-6-type Cytokine Signaling,Role of MAPK Signaling in the Pathogenesis of Influenza,Role of NFAT in Cardiac Hypertrophy,Role of Tissue Factor in Cancer,Sertoli Cell-Sertoli Cell Junction Signaling,STAT3 Pathway,TGF-β Signaling,Thrombin Signaling,Toll-like Receptor Signaling,Type I Diabetes Mellitus Signaling,UVA-Induced MAPK |

| | | | | | |
|---|---|---|---|---|---|
| | | | | | Signaling,UVB-Induced MAPK Signaling,UVC-Induced MAPK Signaling,Xenobiotic Metabolism Signaling |
| MIMAT0004920 | miR-541-3p (and other miRNAs w/seed GGUGGGC) | TargetScan Human | High (predicted) | MAPK14 | 4-1BB Signaling in T Lymphocytes,Acute Phase Response Signaling,AMPK Signaling,Amyloid Processing,Antioxidant Action of Vitamin C,April Mediated Signaling,ATM Signaling,B Cell Activating Factor Signaling,B Cell Receptor Signaling,BMP signaling pathway,Cardiac Hypertrophy Signaling,CCR3 Signaling in Eosinophils,CCR5 Signaling in Macrophages,CD40 Signaling,Cdc42 Signaling,CDK5 Signaling,Chemokine Signaling,Cholecystokinin/Gastrin-mediated Signaling,Corticotropin Releasing Hormone Signaling,Dendritic Cell Maturation,EGF Signaling,Endothelin-1 Signaling,ErbB Signaling,Factors Promoting Cardiogenesis in Vertebrates,Fc Epsilon RI Signaling,FGF Signaling,FLT3 Signaling in Hematopoietic Progenitor Cells,Germ Cell-Sertoli Cell Junction Signaling,Glucocorticoid Receptor Signaling,GNRH Signaling,HIF1α Signaling,HMGB1 Signaling,IL-1 Signaling,IL-10 Signaling,IL-12 Signaling and Production in Macrophages,IL-15 Signaling,IL-17 Signaling,IL-17A Signaling in Airway Cells,IL-17A Signaling in Fibroblasts,IL-17A Signaling in Gastric Cells,IL-22 Signaling,IL-6 Signaling,Inhibition of Angiogenesis by TSP1,iNOS Signaling,Leukocyte Extravasation Signaling,LPS-stimulated MAPK Signaling,Molecular Mechanisms of Cancer,Mouse Embryonic Stem Cell Pluripotency,NRF2-mediated Oxidative Stress Response,p38 MAPK Signaling,p53 Signaling,Parkinson's Signaling,Paxillin Signaling,PEDF Signaling,PPARα/RXRα Activation,Production of Nitric Oxide and Reactive Oxygen Species in Macrophages,RANK Signaling in Osteoclasts,RAR Activation,Regulation of eIF4 and p70S6K Signaling,Renin-Angiotensin Signaling,Role of IL-17A in Arthritis,Role of JAK family kinases in IL-6-type Cytokine Signaling,Role of Macrophages, Fibroblasts and Endothelial Cells in Rheumatoid Arthritis,Role of MAPK Signaling in the Pathogenesis of Influenza,Role of NFAT in Cardiac Hypertrophy,Role of Osteoblasts, Osteoclasts and Chondrocytes in Rheumatoid Arthritis,Role of PKR in Interferon Induction and Antiviral Response,Role of Tissue Factor in Cancer,Sertoli Cell-Sertoli Cell Junction Signaling,STAT3 Pathway,TGF-β Signaling,Thrombin Signaling,Toll-like Receptor Signaling,Type I Diabetes Mellitus Signaling,UVA-Induced MAPK Signaling,UVB-Induced MAPK Signaling,UVC-Induced MAPK Signaling,Xenobiotic Metabolism Signaling |
| MIMAT0004920 | miR-541-3p (and other miRNAs w/seed GGUGGGC) | TargetScan Human | Moderate (predicted) | MAPK8IP3 | Reelin Signaling in Neurons,SAPK/JNK Signaling |

| | | | | | |
|---|---|---|---|---|---|
| MIMAT0004920 | miR-541-3p (and other miRNAs w/seed GGUGGGC) | TargetScan Human | Moderate (predicted) | MAPK9 | 14-3-3-mediated Signaling,4-1BB Signaling in T Lymphocytes,Activation of IRF by Cytosolic Pattern Recognition Receptors,Acute Phase Response Signaling,Agrin Interactions at Neuromuscular Junction,Antioxidant Action of Vitamin C,April Mediated Signaling,ATM Signaling,B Cell Activating Factor Signaling,B Cell Receptor Signaling,BMP signaling pathway,Cardiac Hypertrophy Signaling,CCR5 Signaling in Macrophages,CD27 Signaling in Lymphocytes,CD28 Signaling in T Helper Cells,CD40 Signaling,Cdc42 Signaling,CDK5 Signaling,Cholecystokinin/Gastrin-mediated Signaling,Colorectal Cancer Metastasis Signaling,CXCR4 Signaling,Dendritic Cell Maturation,Endothelin-1 Signaling,ErbB Signaling,Fc Epsilon RI Signaling,FcγRIIB Signaling in B Lymphocytes,FXR/RXR Activation,GDNF Family Ligand-Receptor Interactions,Germ Cell-Sertoli Cell Junction Signaling,Glucocorticoid Receptor Signaling,GNRH Signaling,Gα12/13 Signaling,Hepatic Cholestasis,HGF Signaling,HIF1α Signaling,HMGB1 Signaling,Huntington's Disease Signaling,IL-1 Signaling,IL-12 Signaling and Production in Macrophages,IL-17 Signaling,IL-17A Signaling in Airway Cells,IL-17A Signaling in Gastric Cells,IL-22 Signaling,IL-6 Signaling,IL-8 Signaling,ILK Signaling,Induction of Apoptosis by HIV1,Inhibition of Angiogenesis by TSP1,Leukocyte Extravasation Signaling,LPS-stimulated MAPK Signaling,LPS/IL-1 Mediated Inhibition of RXR Function,MIF Regulation of Innate Immunity,Mitochondrial Dysfunction,Molecular Mechanisms of Cancer,Myc Mediated Apoptosis Signaling,NGF Signaling,NRF2-mediated Oxidative Stress Response,OX40 Signaling Pathway,PAK Signaling,Pancreatic Adenocarcinoma Signaling,Paxillin Signaling,PCP pathway,Production of Nitric Oxide and Reactive Oxygen Species in Macrophages,Pyridoxal 5'-phosphate Salvage Pathway,RANK Signaling in Osteoclasts,RAR Activation,Reelin Signaling in Neurons,Regulation of IL-2 Expression in Activated and Anergic T Lymphocytes,Renin-Angiotensin Signaling,Role of IL-17A in Arthritis,Role of JAK family kinases in IL-6-type Cytokine Signaling,Role of Macrophages, Fibroblasts and Endothelial Cells in Rheumatoid Arthritis,Role of MAPK Signaling in the Pathogenesis of Influenza,Role of NFAT in Cardiac Hypertrophy,Role of Osteoblasts, Osteoclasts and Chondrocytes in Rheumatoid Arthritis,Role of Pattern Recognition Receptors in Recognition of Bacteria and Viruses,Salvage Pathways of Pyrimidine Ribonucleotides,SAPK/JNK Signaling,Sertoli Cell-Sertoli Cell Junction Signaling,Signaling by Rho Family GTPases,STAT3 Pathway,Tec Kinase Signaling,TGF-β Signaling,Type I Diabetes Mellitus Signaling,Type II Diabetes Mellitus Signaling,UVA-Induced MAPK Signaling,UVB-Induced MAPK Signaling,UVC-Induced MAPK Signaling,Xenobiotic Metabolism Signaling |

| | | | | | |
|---|---|---|---|---|---|
| MIMAT0004920 | miR-541-3p (and other miRNAs w/seed GGUGGGC) | TargetScan Human | High (predicted) | MEF2D | Calcium Signaling,Calcium-induced T Lymphocyte Apoptosis,Cardiac Hypertrophy Signaling,Cholecystokinin/Gastrin-mediated Signaling,Corticotropin Releasing Hormone Signaling,ERK5 Signaling,Gα12/13 Signaling,Nur77 Signaling in T Lymphocytes,p38 MAPK Signaling,Phospholipase C Signaling,Role of NFAT in Cardiac Hypertrophy,Role of NFAT in Regulation of the Immune Response |
| MIMAT0004920 | miR-541-3p (and other miRNAs w/seed GGUGGGC) | TargetScan Human | Moderate (predicted) | MRAS | 14-3-3-mediated Signaling,Actin Cytoskeleton Signaling,Actin Nucleation by ARP-WASP Complex,Acute Myeloid Leukemia Signaling,Acute Phase Response Signaling,Agrin Interactions at Neuromuscular Junction,AMPK Signaling,Androgen Signaling,Angiopoietin Signaling,Antiproliferative Role of Somatostatin Receptor 2,Apoptosis Signaling,Axonal Guidance Signaling,B Cell Receptor Signaling,Bladder Cancer Signaling,BMP signaling pathway,Breast Cancer Regulation by Stathmin1,Cardiac Hypertrophy Signaling,Cardiac β-adrenergic Signaling,CCR3 Signaling in Eosinophils,CCR5 Signaling in Macrophages,CDK5 Signaling,Ceramide Signaling,Chemokine Signaling,Cholecystokinin/Gastrin-mediated Signaling,Chronic Myeloid Leukemia Signaling,CNTF Signaling,Colorectal Cancer Metastasis Signaling,CREB Signaling in Neurons,CXCR4 Signaling,EIF2 Signaling,Endometrial Cancer Signaling,Endothelin-1 Signaling,Ephrin B Signaling,Ephrin Receptor Signaling,Epithelial Adherens Junction Signaling,ErbB Signaling,ErbB2-ErbB3 Signaling,ErbB4 Signaling,ERK/MAPK Signaling,ERK5 Signaling,Erythropoietin Signaling,Estrogen Receptor Signaling,Estrogen-Dependent Breast Cancer Signaling,FAK Signaling,Fc Epsilon RI Signaling,FcγRIIB Signaling in B Lymphocytes,FLT3 Signaling in Hematopoietic Progenitor Cells,fMLP Signaling in Neutrophils,G Beta Gamma Signaling,G Protein Signaling Mediated by Tubby,G-Protein Coupled Receptor Signaling,GABA Receptor Signaling,Gap Junction Signaling,GDNF Family Ligand-Receptor Interactions,Germ Cell-Sertoli Cell Junction Signaling,Glioblastoma Multiforme Signaling,Glioma Invasiveness Signaling,Glioma Signaling,Glucocorticoid Receptor Signaling,GM-CSF Signaling,GNRH Signaling,Gα12/13 Signaling,Gαi Signaling,Gαq Signaling,Gαs Signaling,HER-2 Signaling in Breast Cancer,Hereditary Breast Cancer Signaling,HGF Signaling,HIF1α Signaling,HMGB1 Signaling,Human Embryonic Stem Cell Pluripotency,IGF-1 Signaling,IL-1 Signaling,IL-15 Signaling,IL-17 Signaling,IL-2 Signaling,IL-3 Signaling,IL-4 Signaling,IL-6 Signaling,IL-8 Signaling,Insulin Receptor Signaling,Integrin Signaling,JAK/Stat Signaling,LPS-stimulated MAPK Signaling,Macropinocytosis Signaling,Melanocyte Development and Pigmentation Signaling,Melanoma Signaling,Molecular Mechanisms of Cancer,Mouse Embryonic Stem Cell Pluripotency,mTOR Signaling,Myc Mediated Apoptosis Signaling,Natural Killer Cell |

| | | | | | |
|---|---|---|---|---|---|
| | | | | | Signaling,Neuregulin Signaling,Neurotrophin/TRK Signaling,NF-κB Activation by Viruses,NF-κB Signaling,NGF Signaling,Non-Small Cell Lung Cancer Signaling,NRF2-mediated Oxidative Stress Response,Oncostatin M Signaling,Ovarian Cancer Signaling,P2Y Purigenic Receptor Signaling Pathway,p70S6K Signaling,PAK Signaling,Paxillin Signaling,PDGF Signaling,PEDF Signaling,Phospholipase C Signaling,PI3K Signaling in B Lymphocytes,PI3K/AKT Signaling,PKCθ Signaling in T Lymphocytes,PPAR Signaling,PPARα/RXRα Activation,Prolactin Signaling,Prostate Cancer Signaling,PTEN Signaling,Rac Signaling,Regulation of Cellular Mechanics by Calpain Protease,Regulation of eIF4 and p70S6K Signaling,Regulation of IL-2 Expression in Activated and Anergic T Lymphocytes,Regulation of the Epithelial-Mesenchymal Transition Pathway,Relaxin Signaling,Renal Cell Carcinoma Signaling,Renin-Angiotensin Signaling,RhoGDI Signaling,Role of JAK1 and JAK3 in γc Cytokine Signaling,Role of Macrophages, Fibroblasts and Endothelial Cells in Rheumatoid Arthritis,Role of MAPK Signaling in the Pathogenesis of Influenza,Role of NANOG in Mammalian Embryonic Stem Cell Pluripotency,Role of NFAT in Cardiac Hypertrophy,Role of NFAT in Regulation of the Immune Response,Role of Tissue Factor in Cancer,SAPK/JNK Signaling,Sertoli Cell-Sertoli Cell Junction Signaling,Signaling by Rho Family GTPases,Sperm Motility,STAT3 Pathway,Synaptic Long Term Depression,Synaptic Long Term Potentiation,Systemic Lupus Erythematosus Signaling,T Cell Receptor Signaling,Tec Kinase Signaling,Telomerase Signaling,TGF-β Signaling,Thrombin Signaling,Thrombopoietin Signaling,Thyroid Cancer Signaling,UVA-Induced MAPK Signaling,UVC-Induced MAPK Signaling,VEGF Family Ligand-Receptor Interactions,VEGF Signaling,Virus Entry via Endocytic Pathways,Xenobiotic Metabolism Signaling,α-Adrenergic Signaling |
| MIMAT0004920 | miR-541-3p (and other miRNAs w/seed GGUGGGC) | TargetScan Human | Moderate (predicted) | MYLK3 | Actin Cytoskeleton Signaling,Integrin Signaling,Protein Kinase A Signaling,RhoA Signaling |
| MIMAT0004920 | miR-541-3p (and other miRNAs w/seed GGUGGGC) | TargetScan Human | Moderate (predicted) | NCS1 | Dopamine Receptor Signaling |
| MIMAT0004920 | miR-541-3p (and other miRNAs w/seed | TargetScan Human | Moderate (predicted) | NFATC3 | April Mediated Signaling,Axonal Guidance Signaling,B Cell Activating Factor Signaling,B Cell Receptor Signaling,Calcium Signaling,CD28 Signaling in T Helper Cells,fMLP Signaling in Neutrophils,Glucocorticoid Receptor |

| | | | | | |
|---|---|---|---|---|---|
| | GGUGGGC) | | | | Signaling,Gαq Signaling,iCOS-iCOSL Signaling in T Helper Cells,IL-4 Signaling,Netrin Signaling,Phospholipase C Signaling,PI3K Signaling in B Lymphocytes,PKCθ Signaling in T Lymphocytes,Protein Kinase A Signaling,Regulation of IL-2 Expression in Activated and Anergic T Lymphocytes,Role of Macrophages, Fibroblasts and Endothelial Cells in Rheumatoid Arthritis,Role of NFAT in Regulation of the Immune Response,Role of Osteoblasts, Osteoclasts and Chondrocytes in Rheumatoid Arthritis,SAPK/JNK Signaling,Systemic Lupus Erythematosus Signaling,T Cell Receptor Signaling,Wnt/Ca+ pathway |
| MIMAT0004920 | miR-541-3p (and other miRNAs w/seed GGUGGGC) | TargetScan Human | Moderate (predicted) | NGFR | Acute Phase Response Signaling,Axonal Guidance Signaling,CDK5 Signaling,Ceramide Signaling,Dendritic Cell Maturation,Ephrin A Signaling,Granulocyte Adhesion and Diapedesis,Hepatic Cholestasis,Hepatic Fibrosis / Hepatic Stellate Cell Activation,HMGB1 Signaling,IL-6 Signaling,Induction of Apoptosis by HIV1,LPS/IL-1 Mediated Inhibition of RXR Function,LXR/RXR Activation,Neurotrophin/TRK Signaling,NF-κB Signaling,NGF Signaling,PPAR Signaling,Production of Nitric Oxide and Reactive Oxygen Species in Macrophages,Protein Kinase A Signaling,PTEN Signaling,Role of Macrophages, Fibroblasts and Endothelial Cells in Rheumatoid Arthritis,Role of Osteoblasts, Osteoclasts and Chondrocytes in Rheumatoid Arthritis,STAT3 Pathway,T Helper Cell Differentiation,Tight Junction Signaling,Type I Diabetes Mellitus Signaling,Type II Diabetes Mellitus Signaling |
| MIMAT0004920 | miR-541-3p (and other miRNAs w/seed GGUGGGC) | TargetScan Human | Moderate (predicted) | NR2F1 | Aryl Hydrocarbon Receptor Signaling,PPAR Signaling,PPARα/RXRα Activation,RAR Activation,Role of Oct4 in Mammalian Embryonic Stem Cell Pluripotency |
| MIMAT0004920 | miR-541-3p (and other miRNAs w/seed GGUGGGC) | TargetScan Human | Moderate (predicted) | NR5A1 | Role of Oct4 in Mammalian Embryonic Stem Cell Pluripotency |
| MIMAT0004920 | miR-541-3p (and other miRNAs w/seed GGUGGGC) | TargetScan Human | Moderate (predicted) | NTN1 | Axonal Guidance Signaling,Netrin Signaling,Protein Kinase A Signaling |
| MIMAT0004920 | miR-541-3p (and other miRNAs | TargetScan Human | High (predicted) | NTRK2 | Axonal Guidance Signaling,CDK5 Signaling,Human Embryonic Stem Cell Pluripotency,Neuropathic Pain Signaling In Dorsal Horn |

| | | | | | |
|---|---|---|---|---|---|
| | w/seed GGUGGGC) | | | | Neurons,Neurotrophin/TRK Signaling,NF-κB Signaling,PTEN Signaling,STAT3 Pathway,Thyroid Cancer Signaling |
| MIMAT0004920 | miR-541-3p (and other miRNAs w/seed GGUGGGC) | TargetScan Human | Moderate (predicted) | NTRK3 | Axonal Guidance Signaling,Human Embryonic Stem Cell Pluripotency,Neurotrophin/TRK Signaling,NF-κB Signaling,PTEN Signaling,STAT3 Pathway,Thyroid Cancer Signaling |
| MIMAT0004920 | miR-541-3p (and other miRNAs w/seed GGUGGGC) | TargetScan Human | High (predicted) | OS9 | Unfolded protein response |
| MIMAT0004920 | miR-541-3p (and other miRNAs w/seed GGUGGGC) | TargetScan Human | Moderate (predicted) | P4HB | Hypoxia Signaling in the Cardiovascular System,Role of Tissue Factor in Cancer,Unfolded protein response |
| MIMAT0004920 | miR-541-3p (and other miRNAs w/seed GGUGGGC) | TargetScan Human | Moderate (predicted) | PBX1 | Glucocorticoid Receptor Signaling |
| MIMAT0004920 | miR-541-3p (and other miRNAs w/seed GGUGGGC) | TargetScan Human | Moderate (predicted) | PCBD1 | Dopamine Receptor Signaling,Phenylalanine Degradation I (Aerobic),Serotonin Receptor Signaling,Tyrosine Biosynthesis IV |
| MIMAT0004920 | miR-541-3p (and other miRNAs w/seed GGUGGGC) | TargetScan Human | High (predicted) | PDE4B | cAMP-mediated signaling,Cardiac β-adrenergic Signaling,Cellular Effects of Sildenafil (Viagra),G-Protein Coupled Receptor Signaling,Gustation Pathway,Protein Kinase A Signaling,Relaxin Signaling,Sperm Motility,tRNA Splicing |
| MIMAT0004920 | miR-541-3p (and other miRNAs w/seed GGUGGGC) | TargetScan Human | Moderate (predicted) | PDE6B | cAMP-mediated signaling,Cardiac β-adrenergic Signaling,G-Protein Coupled Receptor Signaling,Gustation Pathway,Phototransduction Pathway,Protein Kinase A Signaling,Relaxin Signaling,tRNA Splicing |
| MIMAT0004920 | miR-541-3p (and other miRNAs w/seed | TargetScan Human | High (predicted) | PDE6G | cAMP-mediated signaling,Cardiac β-adrenergic Signaling,G-Protein Coupled Receptor Signaling,Gustation Pathway,Phototransduction Pathway,Protein Kinase A Signaling,Relaxin Signaling,tRNA Splicing |

| | | | | | |
|---|---|---|---|---|---|
| | GGUGGGC) | | | | |
| MIMAT0004920 | miR-541-3p (and other miRNAs w/seed GGUGGGC) | TargetScan Human | High (predicted) | PDGFRB | Glioblastoma Multiforme Signaling,Glioma Signaling,Hepatic Fibrosis / Hepatic Stellate Cell Activation,Human Embryonic Stem Cell Pluripotency,NF-κB Signaling,PAK Signaling,PDGF Signaling,PPAR Signaling,PTEN Signaling,Regulation of the Epithelial-Mesenchymal Transition Pathway,Sphingosine-1-phosphate Signaling,STAT3 Pathway |
| MIMAT0004920 | miR-541-3p (and other miRNAs w/seed GGUGGGC) | TargetScan Human | Moderate (predicted) | PFKFB2 | AMPK Signaling |
| MIMAT0004920 | miR-541-3p (and other miRNAs w/seed GGUGGGC) | TargetScan Human | Moderate (predicted) | PFKFB4 | AMPK Signaling |
| MIMAT0004920 | miR-541-3p (and other miRNAs w/seed GGUGGGC) | TargetScan Human | High (predicted) | PIK3R6 | 14-3-3-mediated Signaling,3-phosphoinositide Biosynthesis,Actin Cytoskeleton Signaling,Acute Myeloid Leukemia Signaling,Aldosterone Signaling in Epithelial Cells,AMPK Signaling,Amyotrophic Lateral Sclerosis Signaling,Angiopoietin Signaling,Antiproliferative Role of Somatostatin Receptor 2,Axonal Guidance Signaling,B Cell Receptor Signaling,Breast Cancer Regulation by Stathmin1,Cardiac Hypertrophy Signaling,CCR3 Signaling in Eosinophils,CD28 Signaling in T Helper Cells,CD40 Signaling,Ceramide Signaling,Chronic Myeloid Leukemia Signaling,Clathrin-mediated Endocytosis Signaling,CNTF Signaling,Colorectal Cancer Metastasis Signaling,CREB Signaling in Neurons,CTLA4 Signaling in Cytotoxic T Lymphocytes,CXCR4 Signaling,Dendritic Cell Maturation,Docosahexaenoic Acid (DHA) Signaling,EGF Signaling,EIF2 Signaling,Endometrial Cancer Signaling,Endothelin-1 Signaling,eNOS Signaling,Ephrin A Signaling,ErbB Signaling,ErbB2-ErbB3 Signaling,ErbB4 Signaling,ERK/MAPK Signaling,Erythropoietin Signaling,Estrogen-Dependent Breast Cancer Signaling,FAK Signaling,Fc Epsilon RI Signaling,FcγRIIB Signaling in B Lymphocytes,FGF Signaling,FLT3 Signaling in Hematopoietic Progenitor Cells,fMLP Signaling in Neutrophils,G-Protein Coupled Receptor Signaling,Gap Junction Signaling,GDNF Family Ligand-Receptor Interactions,Germ Cell-Sertoli Cell Junction Signaling,Glioblastoma Multiforme Signaling,Glioma Invasiveness Signaling,Glioma Signaling,Glucocorticoid Receptor Signaling,GM-CSF Signaling,Growth Hormone Signaling,Gα12/13 Signaling,Gαq Signaling,HER-2 Signaling in Breast Cancer,Hereditary Breast |

| | | | | | |
|---|---|---|---|---|---|
| | | | | | Cancer Signaling,HGF Signaling,HIF1α Signaling,HMGB1 Signaling,Human Embryonic Stem Cell Pluripotency,Huntington's Disease Signaling,iCOS-iCOSL Signaling in T Helper Cells,IGF-1 Signaling,IL-12 Signaling and Production in Macrophages,IL-15 Signaling,IL-17 Signaling,IL-17A Signaling in Airway Cells,IL-2 Signaling,IL-3 Signaling,IL-4 Signaling,IL-6 Signaling,IL-8 Signaling,IL-9 Signaling,ILK Signaling,Insulin Receptor Signaling,Integrin Signaling,JAK/Stat Signaling,Leptin Signaling in Obesity,Leukocyte Extravasation Signaling,LPS-stimulated MAPK Signaling,Lymphotoxin β Receptor Signaling,Macropinocytosis Signaling,Melanocyte Development and Pigmentation Signaling,Melanoma Signaling,Molecular Mechanisms of Cancer,Mouse Embryonic Stem Cell Pluripotency,MSP-RON Signaling Pathway,mTOR Signaling,Myc Mediated Apoptosis Signaling,Natural Killer Cell Signaling,Neuropathic Pain Signaling In Dorsal Horn Neurons,Neurotrophin/TRK Signaling,NF-κB Activation by Viruses,NF-κB Signaling,NGF Signaling,Nitric Oxide Signaling in the Cardiovascular System,Non-Small Cell Lung Cancer Signaling,NRF2-mediated Oxidative Stress Response,Ovarian Cancer Signaling,P2Y Purigenic Receptor Signaling Pathway,p53 Signaling,p70S6K Signaling,PAK Signaling,Pancreatic Adenocarcinoma Signaling,Paxillin Signaling,PDGF Signaling,PEDF Signaling,phagosome formation,PKCθ Signaling in T Lymphocytes,Production of Nitric Oxide and Reactive Oxygen Species in Macrophages,Prolactin Signaling,Prostate Cancer Signaling,Rac Signaling,RANK Signaling in Osteoclasts,Reelin Signaling in Neurons,Regulation of eIF4 and p70S6K Signaling,Regulation of the Epithelial-Mesenchymal Transition Pathway,Relaxin Signaling,Renal Cell Carcinoma Signaling,Renin-Angiotensin Signaling,Role of IL-17A in Arthritis,Role of JAK1 and JAK3 in γc Cytokine Signaling,Role of Macrophages, Fibroblasts and Endothelial Cells in Rheumatoid Arthritis,Role of NANOG in Mammalian Embryonic Stem Cell Pluripotency,Role of NFAT in Cardiac Hypertrophy,Role of NFAT in Regulation of the Immune Response,Role of Osteoblasts, Osteoclasts and Chondrocytes in Rheumatoid Arthritis,Role of p14/p19ARF in Tumor Suppression,Role of Pattern Recognition Receptors in Recognition of Bacteria and Viruses,Role of PI3K/AKT Signaling in the Pathogenesis of Influenza,Role of Tissue Factor in Cancer,SAPK/JNK Signaling,Signaling by Rho Family GTPases,Small Cell Lung Cancer Signaling,Sphingosine-1-phosphate Signaling,Superpathway of Inositol Phosphate Compounds,Systemic Lupus Erythematosus Signaling,T Cell Receptor Signaling,Tec Kinase Signaling,Telomerase Signaling,Thrombin Signaling,Thrombopoietin Signaling,TR/RXR Activation,Type II Diabetes Mellitus Signaling,UVA-Induced MAPK Signaling,UVB-Induced MAPK Signaling,VEGF Family Ligand-Receptor Interactions,VEGF Signaling,Virus |

| | | | | | |
|---|---|---|---|---|---|
| | | | | | Entry via Endocytic Pathways,Xenobiotic Metabolism Signaling |
| MIMAT0004920 | miR-541-3p (and other miRNAs w/seed GGUGGGC) | TargetScan Human | Moderate (predicted) | PIP4K2B | 3-phosphoinositide Biosynthesis,Actin Cytoskeleton Signaling,Aldosterone Signaling in Epithelial Cells,D-myo-inositol (1,4,5)-Trisphosphate Biosynthesis,Rac Signaling,Regulation of Actin-based Motility by Rho,RhoA Signaling,RhoGDI Signaling,Signaling by Rho Family GTPases,Superpathway of Inositol Phosphate Compounds |
| MIMAT0004920 | miR-541-3p (and other miRNAs w/seed GGUGGGC) | TargetScan Human | High (predicted) | PIP5K1C | 3-phosphoinositide Biosynthesis,Actin Cytoskeleton Signaling,Aldosterone Signaling in Epithelial Cells,Clathrin-mediated Endocytosis Signaling,D-myo-inositol (1,4,5)-Trisphosphate Biosynthesis,Rac Signaling,Regulation of Actin-based Motility by Rho,RhoA Signaling,RhoGDI Signaling,Signaling by Rho Family GTPases,Superpathway of Inositol Phosphate Compounds |
| MIMAT0004920 | miR-541-3p (and other miRNAs w/seed GGUGGGC) | TargetScan Human | Moderate (predicted) | PLA2G5 | Antioxidant Action of Vitamin C,Atherosclerosis Signaling,CCR3 Signaling in Eosinophils,Eicosanoid Signaling,Endothelin-1 Signaling,ERK/MAPK Signaling,Fc Epsilon RI Signaling,MIF Regulation of Innate Immunity,MIF-mediated Glucocorticoid Regulation,p38 MAPK Signaling,Phospholipase C Signaling,Phospholipases,Role of MAPK Signaling in the Pathogenesis of Influenza,Sperm Motility,Synaptic Long Term Depression,VEGF Family Ligand-Receptor Interactions |
| MIMAT0004920 | miR-541-3p (and other miRNAs w/seed GGUGGGC) | TargetScan Human | High (predicted) | PLCD3 | 14-3-3-mediated Signaling,Aldosterone Signaling in Epithelial Cells,Antioxidant Action of Vitamin C,Axonal Guidance Signaling,Cardiac Hypertrophy Signaling,Cellular Effects of Sildenafil (Viagra),CREB Signaling in Neurons,D-myo-inositol (1,4,5)-Trisphosphate Biosynthesis,D-myo-inositol-5-phosphate Metabolism,Dendritic Cell Maturation,Dopamine-DARPP32 Feedback in cAMP Signaling,Endothelin-1 Signaling,Gap Junction Signaling,Glioblastoma Multiforme Signaling,GPCR-Mediated Integration of Enteroendocrine Signaling Exemplified by an L Cell,GPCR-Mediated Nutrient Sensing in Enteroendocrine Cells,Leptin Signaling in Obesity,Melatonin Signaling,Neuropathic Pain Signaling In Dorsal Horn Neurons,P2Y Purigenic Receptor Signaling Pathway,p70S6K Signaling,phagosome formation,Phospholipases,PI3K Signaling in B Lymphocytes,PPARα/RXRα Activation,Protein Kinase A Signaling,Role of Macrophages, Fibroblasts and Endothelial Cells in Rheumatoid Arthritis,Role of NFAT in Cardiac Hypertrophy,Sperm Motility,Sphingosine-1-phosphate Signaling,Superpathway of Inositol Phosphate Compounds,Synaptic Long Term Depression,Synaptic Long Term Potentiation,Thrombin Signaling,UVA-Induced MAPK Signaling,Wnt/Ca+ pathway |

| | | | | | |
|---|---|---|---|---|---|
| MIMAT0004920 | miR-541-3p (and other miRNAs w/seed GGUGGGC) | TargetScan Human | Moderate (predicted) | PLD1 | Antioxidant Action of Vitamin C,Choline Biosynthesis III,Endothelin-1 Signaling,Fcγ Receptor-mediated Phagocytosis in Macrophages and Monocytes,Gαq Signaling,IL-8 Signaling,mTOR Signaling,p70S6K Signaling,Pancreatic Adenocarcinoma Signaling,Phospholipase C Signaling,Phospholipases,Rac Signaling,RhoA Signaling,Signaling by Rho Family GTPases |
| MIMAT0004920 | miR-541-3p (and other miRNAs w/seed GGUGGGC) | TargetScan Human | Moderate (predicted) | PLD2 | Antioxidant Action of Vitamin C,Choline Biosynthesis III,Endothelin-1 Signaling,Fcγ Receptor-mediated Phagocytosis in Macrophages and Monocytes,Gαq Signaling,IL-8 Signaling,mTOR Signaling,Pancreatic Adenocarcinoma Signaling,Phospholipase C Signaling,Phospholipases |
| MIMAT0004920 | miR-541-3p (and other miRNAs w/seed GGUGGGC) | TargetScan Human | Moderate (predicted) | PLXNA2 | Axonal Guidance Signaling |
| MIMAT0004920 | miR-541-3p (and other miRNAs w/seed GGUGGGC) | TargetScan Human | Moderate (predicted) | PLXNB3 | Axonal Guidance Signaling |
| MIMAT0004920 | miR-541-3p (and other miRNAs w/seed GGUGGGC) | TargetScan Human | Moderate (predicted) | PLXND1 | Axonal Guidance Signaling |
| MIMAT0004920 | miR-541-3p (and other miRNAs w/seed GGUGGGC) | TargetScan Human | Moderate (predicted) | POU2F1 | Glucocorticoid Receptor Signaling,PKCθ Signaling in T Lymphocytes,Role of BRCA1 in DNA Damage Response |
| MIMAT0004920 | miR-541-3p (and other miRNAs w/seed GGUGGGC) | TargetScan Human | Moderate (predicted) | PPM1G | AMPK Signaling |
| MIMAT0004920 | miR-541-3p (and other miRNAs w/seed GGUGGGC) | TargetScan Human | Moderate (predicted) | PPP1R17 | Synaptic Long Term Depression |

| | | | | | |
|---|---|---|---|---|---|
| MIMAT0004920 | miR-541-3p (and other miRNAs w/seed GGUGGGC) | TargetScan Human | High (predicted) | PPP1R1B | 3-phosphoinositide Biosynthesis,3-phosphoinositide Degradation,CDK5 Signaling,D-myo-inositol (1,4,5,6)-Tetrakisphosphate Biosynthesis,D-myo-inositol (3,4,5,6)-tetrakisphosphate Biosynthesis,D-myo-inositol-5-phosphate Metabolism,Dopamine Receptor Signaling,Dopamine-DARPP32 Feedback in cAMP Signaling,Protein Kinase A Signaling,Superpathway of Inositol Phosphate Compounds |
| MIMAT0004920 | miR-541-3p (and other miRNAs w/seed GGUGGGC) | TargetScan Human | Moderate (predicted) | PPP3CA | 3-phosphoinositide Biosynthesis,3-phosphoinositide Degradation,Amyotrophic Lateral Sclerosis Signaling,Axonal Guidance Signaling,B Cell Receptor Signaling,Calcium Signaling,Calcium-induced T Lymphocyte Apoptosis,cAMP-mediated signaling,Cardiac Hypertrophy Signaling,CD28 Signaling in T Helper Cells,Clathrin-mediated Endocytosis Signaling,D-myo-inositol (1,4,5,6)-Tetrakisphosphate Biosynthesis,D-myo-inositol (3,4,5,6)-tetrakisphosphate Biosynthesis,D-myo-inositol-5-phosphate Metabolism,Dopamine-DARPP32 Feedback in cAMP Signaling,fMLP Signaling in Neutrophils,Gap Junction Signaling,Glucocorticoid Receptor Signaling,GM-CSF Signaling,Gαq Signaling,iCOS-iCOSL Signaling in T Helper Cells,IL-3 Signaling,Netrin Signaling,nNOS Signaling in Neurons,Nur77 Signaling in T Lymphocytes,Phospholipase C Signaling,PI3K Signaling in B Lymphocytes,PKCθ Signaling in T Lymphocytes,Protein Kinase A Signaling,RANK Signaling in Osteoclasts,Regulation of IL-2 Expression in Activated and Anergic T Lymphocytes,Role of Macrophages, Fibroblasts and Endothelial Cells in Rheumatoid Arthritis,Role of NFAT in Cardiac Hypertrophy,Role of NFAT in Regulation of the Immune Response,Role of Osteoblasts, Osteoclasts and Chondrocytes in Rheumatoid Arthritis,Superpathway of Inositol Phosphate Compounds,Synaptic Long Term Potentiation,T Cell Receptor Signaling,Wnt/Ca+ pathway |
| MIMAT0004920 | miR-541-3p (and other miRNAs w/seed GGUGGGC) | TargetScan Human | Moderate (predicted) | PRKCI | 14-3-3-mediated Signaling,Aldosterone Signaling in Epithelial Cells,Androgen Signaling,Axonal Guidance Signaling,Breast Cancer Regulation by Stathmin1,Calcium-induced T Lymphocyte Apoptosis,CCR3 Signaling in Eosinophils,CCR5 Signaling in Macrophages,Cdc42 Signaling,Cholecystokinin/Gastrin-mediated Signaling,Corticotropin Releasing Hormone Signaling,CREB Signaling in Neurons,CXCR4 Signaling,Dopamine-DARPP32 Feedback in cAMP Signaling,Endothelin-1 Signaling,eNOS Signaling,ErbB Signaling,ErbB4 Signaling,ERK/MAPK Signaling,Erythropoietin Signaling,Factors Promoting Cardiogenesis in Vertebrates,Fc Epsilon RI Signaling,Fcγ Receptor-mediated Phagocytosis in Macrophages and Monocytes,fMLP Signaling in Neutrophils,G Beta Gamma Signaling,Gap Junction Signaling,Glioma Signaling,GNRH Signaling,GPCR-Mediated Nutrient Sensing in Enteroendocrine Cells,Growth Hormone Signaling,Gαq |

| | | | | | |
|---|---|---|---|---|---|
| | | | | | Signaling,Hepatic Cholestasis,HER-2 Signaling in Breast Cancer,HGF Signaling,Huntington's Disease Signaling,IGF-1 Signaling,IL-12 Signaling and Production in Macrophages,IL-15 Production,IL-3 Signaling,IL-8 Signaling,Insulin Receptor Signaling,Leukocyte Extravasation Signaling,LPS-stimulated MAPK Signaling,Macropinocytosis Signaling,Mechanisms of Viral Exit from Host Cells,Melatonin Signaling,Molecular Mechanisms of Cancer,mTOR Signaling,Natural Killer Cell Signaling,Neuregulin Signaling,Neuropathic Pain Signaling In Dorsal Horn Neurons,NF-κB Activation by Viruses,Nitric Oxide Signaling in the Cardiovascular System,nNOS Signaling in Neurons,NRF2-mediated Oxidative Stress Response,P2Y Purigenic Receptor Signaling Pathway,p70S6K Signaling,phagosome formation,Phospholipase C Signaling,PI3K Signaling in B Lymphocytes,Production of Nitric Oxide and Reactive Oxygen Species in Macrophages,Prolactin Signaling,Protein Kinase A Signaling,Rac Signaling,RAR Activation,Renin-Angiotensin Signaling,Role of Macrophages, Fibroblasts and Endothelial Cells in Rheumatoid Arthritis,Role of NFAT in Cardiac Hypertrophy,Role of Pattern Recognition Receptors in Recognition of Bacteria and Viruses,Signaling by Rho Family GTPases,Sperm Motility,Synaptic Long Term Depression,Synaptic Long Term Potentiation,Tec Kinase Signaling,Thrombin Signaling,Thrombopoietin Signaling,Tight Junction Signaling,Type II Diabetes Mellitus Signaling,UVB-Induced MAPK Signaling,UVC-Induced MAPK Signaling,VDR/RXR Activation,VEGF Family Ligand-Receptor Interactions,Virus Entry via Endocytic Pathways,Xenobiotic Metabolism Signaling,α-Adrenergic Signaling |
| MIMAT0004920 | miR-541-3p (and other miRNAs w/seed GGUGGGC) | TargetScan Human | Moderate (predicted) | PTGER3 | cAMP-mediated signaling,Colorectal Cancer Metastasis Signaling,Eicosanoid Signaling,G-Protein Coupled Receptor Signaling,Gαi Signaling |
| MIMAT0004920 | miR-541-3p (and other miRNAs w/seed GGUGGGC) | TargetScan Human | Moderate (predicted) | PTGIR | cAMP-mediated signaling,Eicosanoid Signaling,G-Protein Coupled Receptor Signaling,Gαs Signaling |
| MIMAT0004920 | miR-541-3p (and other miRNAs w/seed GGUGGGC) | TargetScan Human | Moderate (predicted) | PTPN23 | 3-phosphoinositide Biosynthesis,3-phosphoinositide Degradation,D-myo-inositol (1,4,5,6)-Tetrakisphosphate Biosynthesis,D-myo-inositol (3,4,5,6)-tetrakisphosphate Biosynthesis,D-myo-inositol-5-phosphate Metabolism,Protein Kinase A Signaling,Superpathway of Inositol Phosphate Compounds |

| | | | | | |
|---|---|---|---|---|---|
| MIMAT0004920 | miR-541-3p (and other miRNAs w/seed GGUGGGC) | TargetScan Human | Moderate (predicted) | PTPRN | 3-phosphoinositide Biosynthesis,3-phosphoinositide Degradation,D-myo-inositol (1,4,5,6)-Tetrakisphosphate Biosynthesis,D-myo-inositol (3,4,5,6)-tetrakisphosphate Biosynthesis,D-myo-inositol-5-phosphate Metabolism,Protein Kinase A Signaling,Superpathway of Inositol Phosphate Compounds,Type I Diabetes Mellitus Signaling |
| MIMAT0004920 | miR-541-3p (and other miRNAs w/seed GGUGGGC) | TargetScan Human | High (predicted) | PYGB | Glycogen Degradation II,Glycogen Degradation III,Protein Kinase A Signaling,α-Adrenergic Signaling |
| MIMAT0004920 | miR-541-3p (and other miRNAs w/seed GGUGGGC) | TargetScan Human | Moderate (predicted) | RAP2B | B Cell Receptor Signaling,Calcium Signaling,Integrin Signaling,Molecular Mechanisms of Cancer |
| MIMAT0004920 | miR-541-3p (and other miRNAs w/seed GGUGGGC) | TargetScan Human | Moderate (predicted) | RET | GDNF Family Ligand-Receptor Interactions,Thyroid Cancer Signaling |
| MIMAT0004920 | miR-541-3p (and other miRNAs w/seed GGUGGGC) | TargetScan Human | Moderate (predicted) | RGS12 | cAMP-mediated signaling,G-Protein Coupled Receptor Signaling,Gαi Signaling |
| MIMAT0004920 | miR-541-3p (and other miRNAs w/seed GGUGGGC) | TargetScan Human | High (predicted) | RHOA | Actin Cytoskeleton Signaling,Actin Nucleation by ARP-WASP Complex,Axonal Guidance Signaling,Breast Cancer Regulation by Stathmin1,Cardiac Hypertrophy Signaling,CCR3 Signaling in Eosinophils,Chemokine Signaling,Cholecystokinin/Gastrin-mediated Signaling,Colorectal Cancer Metastasis Signaling,CXCR4 Signaling,Ephrin A Signaling,Ephrin B Signaling,Ephrin Receptor Signaling,Epithelial Adherens Junction Signaling,Germ Cell-Sertoli Cell Junction Signaling,Glioblastoma Multiforme Signaling,Glioma Invasiveness Signaling,Gα12/13 Signaling,Gαq Signaling,HMGB1 Signaling,IL-8 Signaling,ILK Signaling,Integrin Signaling,Leukocyte Extravasation Signaling,Macropinocytosis Signaling,Molecular Mechanisms of Cancer,mTOR Signaling,NGF Signaling,PCP pathway,PEDF Signaling,phagosome formation,Phospholipase C Signaling,Production of Nitric Oxide and Reactive Oxygen Species in Macrophages,Protein Kinase A Signaling,Rac Signaling,Regulation of Actin-based Motility by Rho,Regulation of the Epithelial-Mesenchymal Transition |

| | | | | | |
|---|---|---|---|---|---|
| | | | | | Pathway,RhoA Signaling,RhoGDI Signaling,Role of Macrophages, Fibroblasts and Endothelial Cells in Rheumatoid Arthritis,Semaphorin Signaling in Neurons,Signaling by Rho Family GTPases,Sphingosine-1-phosphate Signaling,Tec Kinase Signaling,Thrombin Signaling,Tight Junction Signaling |
| MIMAT0004920 | miR-541-3p (and other miRNAs w/seed GGUGGGC) | TargetScan Human | Moderate (predicted) | RHOD | Actin Nucleation by ARP-WASP Complex,Axonal Guidance Signaling,Cardiac Hypertrophy Signaling,Cholecystokinin/Gastrin-mediated Signaling,Colorectal Cancer Metastasis Signaling,CXCR4 Signaling,Germ Cell-Sertoli Cell Junction Signaling,Glioblastoma Multiforme Signaling,Glioma Invasiveness Signaling,Gαq Signaling,HMGB1 Signaling,IL-8 Signaling,ILK Signaling,Integrin Signaling,Molecular Mechanisms of Cancer,mTOR Signaling,phagosome formation,Phospholipase C Signaling,Production of Nitric Oxide and Reactive Oxygen Species in Macrophages,Regulation of Actin-based Motility by Rho,RhoGDI Signaling,Semaphorin Signaling in Neurons,Signaling by Rho Family GTPases,Sphingosine-1-phosphate Signaling,Tec Kinase Signaling,Thrombin Signaling |
| MIMAT0004920 | miR-541-3p (and other miRNAs w/seed GGUGGGC) | TargetScan Human | Moderate (predicted) | RHOT2 | Actin Nucleation by ARP-WASP Complex,Cardiac Hypertrophy Signaling,Cholecystokinin/Gastrin-mediated Signaling,Colorectal Cancer Metastasis Signaling,CXCR4 Signaling,Germ Cell-Sertoli Cell Junction Signaling,Glioblastoma Multiforme Signaling,Glioma Invasiveness Signaling,Gαq Signaling,HMGB1 Signaling,IL-8 Signaling,ILK Signaling,Integrin Signaling,Mitochondrial Dysfunction,Molecular Mechanisms of Cancer,mTOR Signaling,phagosome formation,Phospholipase C Signaling,Production of Nitric Oxide and Reactive Oxygen Species in Macrophages,Regulation of Actin-based Motility by Rho,RhoGDI Signaling,Semaphorin Signaling in Neurons,Signaling by Rho Family GTPases,Sphingosine-1-phosphate Signaling,Tec Kinase Signaling,Thrombin Signaling |
| MIMAT0004920 | miR-541-3p (and other miRNAs w/seed GGUGGGC) | TargetScan Human | Moderate (predicted) | RND2 | Actin Nucleation by ARP-WASP Complex,Cardiac Hypertrophy Signaling,Cholecystokinin/Gastrin-mediated Signaling,Colorectal Cancer Metastasis Signaling,CXCR4 Signaling,Germ Cell-Sertoli Cell Junction Signaling,Glioblastoma Multiforme Signaling,Glioma Invasiveness Signaling,Gαq Signaling,HMGB1 Signaling,IL-8 Signaling,ILK Signaling,Integrin Signaling,Molecular Mechanisms of Cancer,mTOR Signaling,phagosome formation,Phospholipase C Signaling,Production of Nitric Oxide and Reactive Oxygen Species in Macrophages,Regulation of Actin-based Motility by Rho,RhoGDI Signaling,Semaphorin Signaling in Neurons,Signaling by Rho Family GTPases,Sphingosine-1-phosphate Signaling,Tec Kinase Signaling,Thrombin Signaling |

| | | | | | |
|---|---|---|---|---|---|
| MIMAT0004920 | miR-541-3p (and other miRNAs w/seed GGUGGGC) | TargetScan Human | High (predicted) | RNF41 | Neuregulin Signaling |
| MIMAT0004920 | miR-541-3p (and other miRNAs w/seed GGUGGGC) | TargetScan Human | Moderate (predicted) | RORC | Melatonin Signaling,T Helper Cell Differentiation |
| MIMAT0004920 | miR-541-3p (and other miRNAs w/seed GGUGGGC) | TargetScan Human | High (predicted) | RPTOR | AMPK Signaling,Insulin Receptor Signaling,mTOR Signaling |
| MIMAT0004920 | miR-541-3p (and other miRNAs w/seed GGUGGGC) | TargetScan Human | High (predicted) | SCGB1A1 | Glucocorticoid Receptor Signaling |
| MIMAT0004920 | miR-541-3p (and other miRNAs w/seed GGUGGGC) | TargetScan Human | Moderate (predicted) | SEMA3B | Axonal Guidance Signaling,VDR/RXR Activation |
| MIMAT0004920 | miR-541-3p (and other miRNAs w/seed GGUGGGC) | TargetScan Human | Moderate (predicted) | SEMA4B | Axonal Guidance Signaling |
| MIMAT0004920 | miR-541-3p (and other miRNAs w/seed GGUGGGC) | TargetScan Human | High (predicted) | SERPINE1 | Acute Phase Response Signaling,Coagulation System,Glucocorticoid Receptor Signaling,Hepatic Fibrosis / Hepatic Stellate Cell Activation,HMGB1 Signaling,TGF-β Signaling |
| MIMAT0004920 | miR-541-3p (and other miRNAs w/seed GGUGGGC) | TargetScan Human | Moderate (predicted) | SLC18A3 | Dopamine Receptor Signaling,Serotonin Receptor Signaling |
| MIMAT0004920 | miR-541-3p (and other miRNAs | TargetScan Human | High (predicted) | SMARCC1 | AMPK Signaling,Glucocorticoid Receptor Signaling,Hereditary Breast Cancer |

| | | | | | |
|---|---|---|---|---|---|
| | w/seed GGUGGGC) | | | | Signaling,RAR Activation,Role of BRCA1 in DNA Damage Response |
| MIMAT0004920 | miR-541-3p (and other miRNAs w/seed GGUGGGC) | TargetScan Human | Moderate (predicted) | SMARCC2 | AMPK Signaling,Glucocorticoid Receptor Signaling,Hereditary Breast Cancer Signaling,RAR Activation,Role of BRCA1 in DNA Damage Response |
| MIMAT0004920 | miR-541-3p (and other miRNAs w/seed GGUGGGC) | TargetScan Human | Moderate (predicted) | SMARCE1 | AMPK Signaling,Glucocorticoid Receptor Signaling,Hereditary Breast Cancer Signaling,RAR Activation,Role of BRCA1 in DNA Damage Response |
| MIMAT0004920 | miR-541-3p (and other miRNAs w/seed GGUGGGC) | TargetScan Human | Moderate (predicted) | SORBS1 | Ephrin Receptor Signaling,Epithelial Adherens Junction Signaling,Germ Cell-Sertoli Cell Junction Signaling,Sertoli Cell-Sertoli Cell Junction Signaling |
| MIMAT0004920 | miR-541-3p (and other miRNAs w/seed GGUGGGC) | TargetScan Human | Moderate (predicted) | SSTR5 | GPCR-Mediated Integration of Enteroendocrine Signaling Exemplified by an L Cell |
| MIMAT0004920 | miR-541-3p (and other miRNAs w/seed GGUGGGC) | TargetScan Human | High (predicted) | SYNGAP1 | G-Protein Coupled Receptor Signaling,Molecular Mechanisms of Cancer |
| MIMAT0004920 | miR-541-3p (and other miRNAs w/seed GGUGGGC) | TargetScan Human | Moderate (predicted) | SYNJ1 | 1D-myo-inositol Hexakisphosphate Biosynthesis II (Mammalian),3-phosphoinositide Biosynthesis,3-phosphoinositide Degradation,B Cell Receptor Signaling,Clathrin-mediated Endocytosis Signaling,D-myo-inositol (1,3,4)-trisphosphate Biosynthesis,D-myo-inositol (1,4,5)-trisphosphate Degradation,D-myo-inositol (1,4,5,6)-Tetrakisphosphate Biosynthesis,D-myo-inositol (3,4,5,6)-tetrakisphosphate Biosynthesis,D-myo-inositol-5-phosphate Metabolism,Fc Epsilon RI Signaling,IL-4 Signaling,Insulin Receptor Signaling,Natural Killer Cell Signaling,PDGF Signaling,PI3K/AKT Signaling,PTEN Signaling,Superpathway of D-myo-inositol (1,4,5)-trisphosphate Metabolism,Superpathway of Inositol Phosphate Compounds |
| MIMAT0004920 | miR-541-3p (and other miRNAs w/seed | TargetScan Human | High (predicted) | TAB1 | Acute Phase Response Signaling,BMP signaling pathway,Cardiac Hypertrophy Signaling,Dendritic Cell Maturation,Glucocorticoid Receptor Signaling,IL-1 Signaling,IL-10 Signaling,IL-6 Signaling,iNOS Signaling,Molecular Mechanisms |

| | | | | | |
|---|---|---|---|---|---|
| | GGUGGGC) | | | | of Cancer,Mouse Embryonic Stem Cell Pluripotency,NF-κB Signaling,p38 MAPK Signaling,PPAR Signaling,SAPK/JNK Signaling,TGF-β Signaling,Toll-like Receptor Signaling,Wnt/β-catenin Signaling |
| MIMAT0004920 | miR-541-3p (and other miRNAs w/seed GGUGGGC) | TargetScan Human | Moderate (predicted) | TBP | Androgen Signaling,Assembly of RNA Polymerase I Complex,Assembly of RNA Polymerase II Complex,Assembly of RNA Polymerase III Complex,CREB Signaling in Neurons,Estrogen Receptor Signaling,Glucocorticoid Receptor Signaling,Huntington's Disease Signaling,NF-κB Activation by Viruses,Thrombin Signaling |
| MIMAT0004920 | miR-541-3p (and other miRNAs w/seed GGUGGGC) | TargetScan Human | Moderate (predicted) | TGFB1 | Adipogenesis pathway,Altered T Cell and B Cell Signaling in Rheumatoid Arthritis,Antiproliferative Role of TOB in T Cell Signaling,Aryl Hydrocarbon Receptor Signaling,Atherosclerosis Signaling,Cardiac Hypertrophy Signaling,Cell Cycle: G1/S Checkpoint Regulation,Chronic Myeloid Leukemia Signaling,Colorectal Cancer Metastasis Signaling,Cyclins and Cell Cycle Regulation,Factors Promoting Cardiogenesis in Vertebrates,Germ Cell-Sertoli Cell Junction Signaling,Glucocorticoid Receptor Signaling,Hepatic Cholestasis,Hepatic Fibrosis / Hepatic Stellate Cell Activation,HMGB1 Signaling,Human Embryonic Stem Cell Pluripotency,IL-12 Signaling and Production in Macrophages,Inhibition of Angiogenesis by TSP1,Mitotic Roles of Polo-Like Kinase,Molecular Mechanisms of Cancer,p38 MAPK Signaling,Pancreatic Adenocarcinoma Signaling,PPARα/RXRα Activation,Protein Kinase A Signaling,RAR Activation,Regulation of IL-2 Expression in Activated and Anergic T Lymphocytes,Regulation of the Epithelial-Mesenchymal Transition Pathway,Renal Cell Carcinoma Signaling,Role of Cytokines in Mediating Communication between Immune Cells,Role of Macrophages, Fibroblasts and Endothelial Cells in Rheumatoid Arthritis,Role of NFAT in Cardiac Hypertrophy,Role of Osteoblasts, Osteoclasts and Chondrocytes in Rheumatoid Arthritis,Role of Pattern Recognition Receptors in Recognition of Bacteria and Viruses,T Helper Cell Differentiation,TGF-β Signaling,Tight Junction Signaling,Wnt/β-catenin Signaling |
| MIMAT0004920 | miR-541-3p (and other miRNAs w/seed GGUGGGC) | TargetScan Human | Moderate (predicted) | THEM4 | Acyl-CoA Hydrolysis,PI3K/AKT Signaling,Stearate Biosynthesis I (Animals) |
| MIMAT0004920 | miR-541-3p (and other miRNAs w/seed | TargetScan Human | High (predicted) | TLN2 | Actin Cytoskeleton Signaling,Crosstalk between Dendritic Cells and Natural Killer Cells,ERK/MAPK Signaling,FAK Signaling,Fcγ Receptor-mediated Phagocytosis in Macrophages and Monocytes,Integrin Signaling,Paxillin |

| | | | | | |
|---|---|---|---|---|---|
| | GGUGGGC) | | | | Signaling,Regulation of Cellular Mechanics by Calpain Protease |
| MIMAT0004920 | miR-541-3p (and other miRNAs w/seed GGUGGGC) | TargetScan Human | High (predicted) | TPM2 | Calcium Signaling |
| MIMAT0004920 | miR-541-3p (and other miRNAs w/seed GGUGGGC) | TargetScan Human | Moderate (predicted) | TRPC6 | Calcium Signaling |
| MIMAT0004920 | miR-541-3p (and other miRNAs w/seed GGUGGGC) | TargetScan Human | High (predicted) | TYK2 | CNTF Signaling,Colorectal Cancer Metastasis Signaling,ErbB2-ErbB3 Signaling,IL-10 Signaling,IL-15 Production,IL-15 Signaling,IL-17A Signaling in Airway Cells,IL-22 Signaling,IL-4 Signaling,iNOS Signaling,Interferon Signaling,JAK/Stat Signaling,Molecular Mechanisms of Cancer,Mouse Embryonic Stem Cell Pluripotency,Oncostatin M Signaling,Pancreatic Adenocarcinoma Signaling,PDGF Signaling,PI3K/AKT Signaling,Production of Nitric Oxide and Reactive Oxygen Species in Macrophages,Regulation of the Epithelial-Mesenchymal Transition Pathway,Role of JAK family kinases in IL-6-type Cytokine Signaling,Role of JAK1, JAK2 and TYK2 in Interferon Signaling,Role of JAK2 in Hormone-like Cytokine Signaling,Role of NANOG in Mammalian Embryonic Stem Cell Pluripotency,STAT3 Pathway,Tec Kinase Signaling |
| MIMAT0004920 | miR-541-3p (and other miRNAs w/seed GGUGGGC) | TargetScan Human | High (predicted) | UNC5C | Axonal Guidance Signaling,Netrin Signaling |
| MIMAT0004920 | miR-541-3p (and other miRNAs w/seed GGUGGGC) | TargetScan Human | High (predicted) | WAS | Actin Cytoskeleton Signaling,Actin Nucleation by ARP-WASP Complex,Axonal Guidance Signaling,CD28 Signaling in T Helper Cells,Cdc42 Signaling,Ephrin Receptor Signaling,Epithelial Adherens Junction Signaling,FAK Signaling,Fcγ Receptor-mediated Phagocytosis in Macrophages and Monocytes,fMLP Signaling in Neutrophils,Integrin Signaling,Leukocyte Extravasation Signaling,Regulation of Actin-based Motility by Rho,Sertoli Cell-Sertoli Cell Junction Signaling,Signaling by Rho Family GTPases,Tec Kinase Signaling |
| MIMAT0004920 | miR-541-3p (and other miRNAs w/seed | TargetScan Human | Moderate (predicted) | WNT1 | Axonal Guidance Signaling,Basal Cell Carcinoma Signaling,Colorectal Cancer Metastasis Signaling,Glioblastoma Multiforme Signaling,Human Embryonic Stem Cell Pluripotency,Molecular Mechanisms of Cancer,Ovarian Cancer |

| | | | | | |
|---|---|---|---|---|---|
| | GGUGGGC) | | | | Signaling,PCP pathway,Regulation of the Epithelial-Mesenchymal Transition Pathway,Role of Macrophages, Fibroblasts and Endothelial Cells in Rheumatoid Arthritis,Role of NANOG in Mammalian Embryonic Stem Cell Pluripotency,Role of Osteoblasts, Osteoclasts and Chondrocytes in Rheumatoid Arthritis,Role of Wnt/GSK-3β Signaling in the Pathogenesis of Influenza,Wnt/β-catenin Signaling |
| MIMAT0004920 | miR-541-3p (and other miRNAs w/seed GGUGGGC) | TargetScan Human | High (predicted) | WNT11 | Axonal Guidance Signaling,Basal Cell Carcinoma Signaling,Colorectal Cancer Metastasis Signaling,Factors Promoting Cardiogenesis in Vertebrates,Glioblastoma Multiforme Signaling,Human Embryonic Stem Cell Pluripotency,Molecular Mechanisms of Cancer,Ovarian Cancer Signaling,PCP pathway,Regulation of the Epithelial-Mesenchymal Transition Pathway,Role of Macrophages, Fibroblasts and Endothelial Cells in Rheumatoid Arthritis,Role of NANOG in Mammalian Embryonic Stem Cell Pluripotency,Role of Osteoblasts, Osteoclasts and Chondrocytes in Rheumatoid Arthritis,Role of Wnt/GSK-3β Signaling in the Pathogenesis of Influenza,Wnt/β-catenin Signaling |
| MIMAT0004920 | miR-541-3p (and other miRNAs w/seed GGUGGGC) | TargetScan Human | Moderate (predicted) | WNT3A | Axonal Guidance Signaling,Basal Cell Carcinoma Signaling,Colorectal Cancer Metastasis Signaling,Glioblastoma Multiforme Signaling,Human Embryonic Stem Cell Pluripotency,Molecular Mechanisms of Cancer,Mouse Embryonic Stem Cell Pluripotency,Ovarian Cancer Signaling,PCP pathway,Regulation of the Epithelial-Mesenchymal Transition Pathway,Role of Macrophages, Fibroblasts and Endothelial Cells in Rheumatoid Arthritis,Role of NANOG in Mammalian Embryonic Stem Cell Pluripotency,Role of Osteoblasts, Osteoclasts and Chondrocytes in Rheumatoid Arthritis,Role of Wnt/GSK-3β Signaling in the Pathogenesis of Influenza,Wnt/β-catenin Signaling |
| MIMAT0004920 | miR-541-3p (and other miRNAs w/seed GGUGGGC) | TargetScan Human | Moderate (predicted) | WNT4 | Axonal Guidance Signaling,Basal Cell Carcinoma Signaling,Colorectal Cancer Metastasis Signaling,Glioblastoma Multiforme Signaling,Human Embryonic Stem Cell Pluripotency,Molecular Mechanisms of Cancer,Ovarian Cancer Signaling,PCP pathway,Regulation of the Epithelial-Mesenchymal Transition Pathway,Role of Macrophages, Fibroblasts and Endothelial Cells in Rheumatoid Arthritis,Role of NANOG in Mammalian Embryonic Stem Cell Pluripotency,Role of Osteoblasts, Osteoclasts and Chondrocytes in Rheumatoid Arthritis,Role of Wnt/GSK-3β Signaling in the Pathogenesis of Influenza,Wnt/β-catenin Signaling |
| MIMAT0004920 | miR-541-3p (and other miRNAs w/seed | TargetScan Human | Moderate (predicted) | ZFP42 | Mouse Embryonic Stem Cell Pluripotency,Role of NANOG in Mammalian Embryonic Stem Cell Pluripotency |

| | | | | | |
|---|---|---|---|---|---|
| | GGUGGGC) | | | | |
| MIMAT0004800 | miR-550a-5p (and other miRNAs w/seed GUGCCUG) | TargetScan Human | Moderate (predicted) | ACACA | AMPK Signaling,Biotin-carboxyl Carrier Protein Assembly,LXR/RXR Activation,TR/RXR Activation |
| MIMAT0004800 | miR-550a-5p (and other miRNAs w/seed GUGCCUG) | TargetScan Human | Moderate (predicted) | ADAMTS5 | Axonal Guidance Signaling,Role of Osteoblasts, Osteoclasts and Chondrocytes in Rheumatoid Arthritis |
| MIMAT0004800 | miR-550a-5p (and other miRNAs w/seed GUGCCUG) | TargetScan Human | High (predicted) | ADAMTS6 | Axonal Guidance Signaling |
| MIMAT0004800 | miR-550a-5p (and other miRNAs w/seed GUGCCUG) | TargetScan Human | High (predicted) | ADCY1 | Breast Cancer Regulation by Stathmin1,cAMP-mediated signaling,Cardiac Hypertrophy Signaling,Cardiac β-adrenergic Signaling,CDK5 Signaling,Cellular Effects of Sildenafil (Viagra),Colorectal Cancer Metastasis Signaling,Corticotropin Releasing Hormone Signaling,CREB Signaling in Neurons,CXCR4 Signaling,Dopamine Receptor Signaling,Dopamine-DARPP32 Feedback in cAMP Signaling,Endothelin-1 Signaling,eNOS Signaling,G Beta Gamma Signaling,G-Protein Coupled Receptor Signaling,GABA Receptor Signaling,Gap Junction Signaling,GNRH Signaling,GPCR-Mediated Integration of Enteroendocrine Signaling Exemplified by an L Cell,GPCR-Mediated Nutrient Sensing in Enteroendocrine Cells,Gustation Pathway,Gαi Signaling,Gαs Signaling,Hepatic Cholestasis,IL-1 Signaling,Leptin Signaling in Obesity,Melanocyte Development and Pigmentation Signaling,Molecular Mechanisms of Cancer,P2Y Purigenic Receptor Signaling Pathway,Phospholipase C Signaling,PPARα/RXRα Activation,Protein Kinase A Signaling,RAR Activation,Relaxin Signaling,Renin-Angiotensin Signaling,Role of NFAT in Cardiac Hypertrophy,Serotonin Receptor Signaling,Sphingosine-1-phosphate Signaling,Synaptic Long Term Potentiation,Thrombin Signaling,α-Adrenergic Signaling |
| MIMAT0004800 | miR-550a-5p (and other miRNAs w/seed GUGCCUG) | TargetScan Human | Moderate (predicted) | ADCY2 | Breast Cancer Regulation by Stathmin1,cAMP-mediated signaling,Cardiac Hypertrophy Signaling,Cardiac β-adrenergic Signaling,CDK5 Signaling,Cellular Effects of Sildenafil (Viagra),Colorectal Cancer Metastasis Signaling,Corticotropin Releasing Hormone Signaling,CREB Signaling in Neurons,CXCR4 Signaling,Dopamine Receptor Signaling,Dopamine-DARPP32 |

| | | | | | |
|---|---|---|---|---|---|
| | | | | | Feedback in cAMP Signaling,Endothelin-1 Signaling,eNOS Signaling,G Beta Gamma Signaling,G-Protein Coupled Receptor Signaling,GABA Receptor Signaling,Gap Junction Signaling,GNRH Signaling,GPCR-Mediated Integration of Enteroendocrine Signaling Exemplified by an L Cell,GPCR-Mediated Nutrient Sensing in Enteroendocrine Cells,Gustation Pathway,Gαi Signaling,Gαs Signaling,Hepatic Cholestasis,IL-1 Signaling,Leptin Signaling in Obesity,Melanocyte Development and Pigmentation Signaling,Molecular Mechanisms of Cancer,P2Y Purigenic Receptor Signaling Pathway,Phospholipase C Signaling,PPARα/RXRα Activation,Protein Kinase A Signaling,RAR Activation,Relaxin Signaling,Renin-Angiotensin Signaling,Role of NFAT in Cardiac Hypertrophy,Serotonin Receptor Signaling,Sphingosine-1-phosphate Signaling,Thrombin Signaling,α-Adrenergic Signaling |
| MIMAT0004800 | miR-550a-5p (and other miRNAs w/seed GUGCCUG) | TargetScan Human | High (predicted) | ADCYAP1 | Circadian Rhythm Signaling,GPCR-Mediated Integration of Enteroendocrine Signaling Exemplified by an L Cell |
| MIMAT0004800 | miR-550a-5p (and other miRNAs w/seed GUGCCUG) | TargetScan Human | Moderate (predicted) | ADD2 | Gαs Signaling,Protein Kinase A Signaling |
| MIMAT0004800 | miR-550a-5p (and other miRNAs w/seed GUGCCUG) | TargetScan Human | Moderate (predicted) | ADIPOQ | AMPK Signaling,PPARα/RXRα Activation,Type II Diabetes Mellitus Signaling |
| MIMAT0004800 | miR-550a-5p (and other miRNAs w/seed GUGCCUG) | TargetScan Human | Moderate (predicted) | ADRB3 | AMPK Signaling,cAMP-mediated signaling,Cardiac Hypertrophy Signaling,G-Protein Coupled Receptor Signaling,GPCR-Mediated Integration of Enteroendocrine Signaling Exemplified by an L Cell,Gαs Signaling,Nitric Oxide Signaling in the Cardiovascular System |
| MIMAT0004800 | miR-550a-5p (and other miRNAs w/seed GUGCCUG) | TargetScan Human | Moderate (predicted) | ADRBK1 | cAMP-mediated signaling,Cardiac β-adrenergic Signaling,Colorectal Cancer Metastasis Signaling,G-Protein Coupled Receptor Signaling,Gαq Signaling,Sonic Hedgehog Signaling |
| MIMAT0004800 | miR-550a-5p (and other miRNAs w/seed | TargetScan Human | Moderate (predicted) | AKAP12 | cAMP-mediated signaling,Cardiac β-adrenergic Signaling,Protein Kinase A Signaling |

| | | | | | |
|---|---|---|---|---|---|
| | GUGCCUG) | | | | |
| MIMAT0004800 | miR-550a-5p (and other miRNAs w/seed GUGCCUG) | TargetScan Human | Moderate (predicted) | AKT3 | 14-3-3-mediated Signaling,Acute Myeloid Leukemia Signaling,Acute Phase Response Signaling,AMPK Signaling,Amyloid Processing,Amyotrophic Lateral Sclerosis Signaling,Angiopoietin Signaling,Axonal Guidance Signaling,B Cell Receptor Signaling,CD28 Signaling in T Helper Cells,Ceramide Signaling,Chronic Myeloid Leukemia Signaling,Colorectal Cancer Metastasis Signaling,CREB Signaling in Neurons,CTLA4 Signaling in Cytotoxic T Lymphocytes,CXCR4 Signaling,Dendritic Cell Maturation,DNA damage-induced 14-3-3σ Signaling,Docosahexaenoic Acid (DHA) Signaling,EGF Signaling,EIF2 Signaling,Endometrial Cancer Signaling,eNOS Signaling,Ephrin Receptor Signaling,Epithelial Adherens Junction Signaling,Erythropoietin Signaling,Estrogen-Dependent Breast Cancer Signaling,FAK Signaling,Fc Epsilon RI Signaling,Fcγ Receptor-mediated Phagocytosis in Macrophages and Monocytes,FGF Signaling,FLT3 Signaling in Hematopoietic Progenitor Cells,FXR/RXR Activation,G Beta Gamma Signaling,G-Protein Coupled Receptor Signaling,Gap Junction Signaling,Glioblastoma Multiforme Signaling,Glioma Signaling,Glucocorticoid Receptor Signaling,GM-CSF Signaling,Gα12/13 Signaling,Gαq Signaling,HER-2 Signaling in Breast Cancer,Hereditary Breast Cancer Signaling,HGF Signaling,HIF1α Signaling,HMGB1 Signaling,Human Embryonic Stem Cell Pluripotency,Huntington's Disease Signaling,iCOS-iCOSL Signaling in T Helper Cells,IGF-1 Signaling,IL-12 Signaling and Production in Macrophages,IL-15 Signaling,IL-17 Signaling,IL-17A Signaling in Airway Cells,IL-2 Signaling,IL-22 Signaling,IL-3 Signaling,IL-4 Signaling,IL-6 Signaling,IL-8 Signaling,ILK Signaling,Inhibition of Angiogenesis by TSP1,Insulin Receptor Signaling,Integrin Signaling,JAK/Stat Signaling,Leptin Signaling in Obesity,Lymphotoxin β Receptor Signaling,Melanoma Signaling,Molecular Mechanisms of Cancer,Mouse Embryonic Stem Cell Pluripotency,mTOR Signaling,Myc Mediated Apoptosis Signaling,Natural Killer Cell Signaling,Neuregulin Signaling,NF-κB Activation by Viruses,NF-κB Signaling,NGF Signaling,Nitric Oxide Signaling in the Cardiovascular System,Non-Small Cell Lung Cancer Signaling,Ovarian Cancer Signaling,P2Y Purigenic Receptor Signaling Pathway,p53 Signaling,p70S6K Signaling,Pancreatic Adenocarcinoma Signaling,PEDF Signaling,PI3K Signaling in B Lymphocytes,PI3K/AKT Signaling,Production of Nitric Oxide and Reactive Oxygen Species in Macrophages,Prostate Cancer Signaling,PTEN Signaling,PXR/RXR Activation,RANK Signaling in Osteoclasts,RAR Activation,Regulation of eIF4 and p70S6K Signaling,Regulation of the Epithelial-Mesenchymal Transition Pathway,Relaxin Signaling,Renal Cell Carcinoma Signaling,Role of Macrophages, Fibroblasts and Endothelial Cells in |

| | | | | | |
|---|---|---|---|---|---|
| | | | | | Rheumatoid Arthritis,Role of MAPK Signaling in the Pathogenesis of Influenza,Role of NANOG in Mammalian Embryonic Stem Cell Pluripotency,Role of NFAT in Cardiac Hypertrophy,Role of NFAT in Regulation of the Immune Response,Role of Osteoblasts, Osteoclasts and Chondrocytes in Rheumatoid Arthritis,Role of PI3K/AKT Signaling in the Pathogenesis of Influenza,Role of Tissue Factor in Cancer,Sertoli Cell-Sertoli Cell Junction Signaling,Small Cell Lung Cancer Signaling,Sphingosine-1-phosphate Signaling,Systemic Lupus Erythematosus Signaling,Telomerase Signaling,Thrombin Signaling,Tight Junction Signaling,TR/RXR Activation,TREM1 Signaling,Type II Diabetes Mellitus Signaling,VEGF Family Ligand-Receptor Interactions,VEGF Signaling,Wnt/β-catenin Signaling |
| MIMAT0004800 | miR-550a-5p (and other miRNAs w/seed GUGCCUG) | TargetScan Human | High (predicted) | ALDH5A1 | 4-aminobutyrate Degradation I,Aryl Hydrocarbon Receptor Signaling,GABA Receptor Signaling,Glutamate Degradation III (via 4-aminobutyrate),LPS/IL-1 Mediated Inhibition of RXR Function,Xenobiotic Metabolism Signaling |
| MIMAT0004800 | miR-550a-5p (and other miRNAs w/seed GUGCCUG) | TargetScan Human | Moderate (predicted) | ARHGEF10 | Breast Cancer Regulation by Stathmin1,Molecular Mechanisms of Cancer,Phospholipase C Signaling,Reelin Signaling in Neurons,RhoGDI Signaling,Signaling by Rho Family GTPases,Thrombin Signaling |
| MIMAT0004800 | miR-550a-5p (and other miRNAs w/seed GUGCCUG) | TargetScan Human | Moderate (predicted) | ARHGEF12 | Actin Cytoskeleton Signaling,Axonal Guidance Signaling,Breast Cancer Regulation by Stathmin1,Molecular Mechanisms of Cancer,Phospholipase C Signaling,Reelin Signaling in Neurons,RhoA Signaling,RhoGDI Signaling,Semaphorin Signaling in Neurons,Signaling by Rho Family GTPases,Thrombin Signaling |
| MIMAT0004800 | miR-550a-5p (and other miRNAs w/seed GUGCCUG) | TargetScan Human | High (predicted) | ARHGEF15 | Axonal Guidance Signaling,Breast Cancer Regulation by Stathmin1,Ephrin Receptor Signaling,Molecular Mechanisms of Cancer,Phospholipase C Signaling,Reelin Signaling in Neurons,RhoGDI Signaling,Signaling by Rho Family GTPases,Thrombin Signaling |
| MIMAT0004800 | miR-550a-5p (and other miRNAs w/seed GUGCCUG) | TargetScan Human | High (predicted) | ARNT | Aryl Hydrocarbon Receptor Signaling,HIF1α Signaling,Hypoxia Signaling in the Cardiovascular System,Renal Cell Carcinoma Signaling,VEGF Signaling,Xenobiotic Metabolism Signaling |
| MIMAT0004800 | miR-550a-5p (and other miRNAs w/seed | TargetScan Human | Moderate (predicted) | ATP2A2 | Calcium Signaling,Calcium Transport I,Calcium-induced T Lymphocyte Apoptosis,Cardiac β-adrenergic Signaling,Dopamine-DARPP32 Feedback in cAMP Signaling,Nitric Oxide Signaling in the Cardiovascular System |

| | | | | | |
|---|---|---|---|---|---|
| | GUGCCUG) | | | | |
| MIMAT0004800 | miR-550a-5p (and other miRNAs w/seed GUGCCUG) | TargetScan Human | Moderate (predicted) | BMP8A | Axonal Guidance Signaling,Basal Cell Carcinoma Signaling,BMP signaling pathway,Factors Promoting Cardiogenesis in Vertebrates,Human Embryonic Stem Cell Pluripotency,Molecular Mechanisms of Cancer,Role of NANOG in Mammalian Embryonic Stem Cell Pluripotency,Role of Osteoblasts, Osteoclasts and Chondrocytes in Rheumatoid Arthritis |
| MIMAT0004800 | miR-550a-5p (and other miRNAs w/seed GUGCCUG) | TargetScan Human | Moderate (predicted) | C9orf3 | Axonal Guidance Signaling |
| MIMAT0004800 | miR-550a-5p (and other miRNAs w/seed GUGCCUG) | TargetScan Human | Moderate (predicted) | CAB39 | AMPK Signaling |
| MIMAT0004800 | miR-550a-5p (and other miRNAs w/seed GUGCCUG) | TargetScan Human | High (predicted) | CD3G | Calcium-induced T Lymphocyte Apoptosis,CCR5 Signaling in Macrophages,CD28 Signaling in T Helper Cells,Cdc42 Signaling,CTLA4 Signaling in Cytotoxic T Lymphocytes,Cytotoxic T Lymphocyte-mediated Apoptosis of Target Cells,Glucocorticoid Receptor Signaling,Hematopoiesis from Pluripotent Stem Cells,iCOS-iCOSL Signaling in T Helper Cells,Nur77 Signaling in T Lymphocytes,OX40 Signaling Pathway,Phospholipase C Signaling,PKCθ Signaling in T Lymphocytes,Regulation of IL-2 Expression in Activated and Anergic T Lymphocytes,Role of NFAT in Regulation of the Immune Response,Systemic Lupus Erythematosus Signaling,T Cell Receptor Signaling,Type I Diabetes Mellitus Signaling |
| MIMAT0004800 | miR-550a-5p (and other miRNAs w/seed GUGCCUG) | TargetScan Human | High (predicted) | CDH5 | Agranulocyte Adhesion and Diapedesis,Granulocyte Adhesion and Diapedesis,Gα12/13 Signaling,Leukocyte Extravasation Signaling,RhoGDI Signaling,Signaling by Rho Family GTPases,Wnt/β-catenin Signaling |
| MIMAT0004800 | miR-550a-5p (and other miRNAs w/seed GUGCCUG) | TargetScan Human | Moderate (predicted) | CPT1A | AMPK Signaling,LPS/IL-1 Mediated Inhibition of RXR Function,Mitochondrial Dysfunction,Mitochondrial L-carnitine Shuttle Pathway,PXR/RXR Activation |
| MIMAT0004800 | miR-550a-5p (and other miRNAs w/seed | TargetScan Human | High (predicted) | CSNK1A1 | Amyloid Processing,Dopamine-DARPP32 Feedback in cAMP Signaling,Gap Junction Signaling,Pyridoxal 5'-phosphate Salvage Pathway,Role of Macrophages, Fibroblasts and Endothelial Cells in Rheumatoid Arthritis,Role |

| | | | | | |
|---|---|---|---|---|---|
| | GUGCCUG) | | | | of NFAT in Cardiac Hypertrophy,Role of NFAT in Regulation of the Immune Response,Role of Osteoblasts, Osteoclasts and Chondrocytes in Rheumatoid Arthritis,Role of Wnt/GSK-3β Signaling in the Pathogenesis of Influenza,Salvage Pathways of Pyrimidine Ribonucleotides,Wnt/β-catenin Signaling |
| MIMAT0004800 | miR-550a-5p (and other miRNAs w/seed GUGCCUG) | TargetScan Human | High (predicted) | DPYSL5 | Axonal Guidance Signaling,Semaphorin Signaling in Neurons |
| MIMAT0004800 | miR-550a-5p (and other miRNAs w/seed GUGCCUG) | TargetScan Human | Moderate (predicted) | EFNA1 | Axonal Guidance Signaling,Ephrin A Signaling,Ephrin Receptor Signaling |
| MIMAT0004800 | miR-550a-5p (and other miRNAs w/seed GUGCCUG) | TargetScan Human | Moderate (predicted) | EGF | Actin Cytoskeleton Signaling,Axonal Guidance Signaling,Bladder Cancer Signaling,Caveolar-mediated Endocytosis Signaling,Clathrin-mediated Endocytosis Signaling,Colorectal Cancer Metastasis Signaling,EGF Signaling,Ephrin Receptor Signaling,Epithelial Adherens Junction Signaling,ErbB Signaling,ERK5 Signaling,FAK Signaling,Gap Junction Signaling,Glioblastoma Multiforme Signaling,Glioma Signaling,GNRH Signaling,Hepatic Fibrosis / Hepatic Stellate Cell Activation,HER-2 Signaling in Breast Cancer,Huntington's Disease Signaling,IL-8 Signaling,Macropinocytosis Signaling,Neuregulin Signaling,NF-κB Signaling,Non-Small Cell Lung Cancer Signaling,Ovarian Cancer Signaling,Pancreatic Adenocarcinoma Signaling,Regulation of Cellular Mechanics by Calpain Protease,Regulation of the Epithelial-Mesenchymal Transition Pathway,Telomerase Signaling,Thrombin Signaling |
| MIMAT0004800 | miR-550a-5p (and other miRNAs w/seed GUGCCUG) | TargetScan Human | Moderate (predicted) | EIF2S3 | EIF2 Signaling,Regulation of eIF4 and p70S6K Signaling,VEGF Signaling |
| MIMAT0004800 | miR-550a-5p (and other miRNAs w/seed GUGCCUG) | TargetScan Human | Moderate (predicted) | ELAVL1 | AMPK Signaling,VEGF Signaling |
| MIMAT0004800 | miR-550a-5p | TargetScan Human | Moderate (predicted) | EPHA1 | Axonal Guidance Signaling,Ephrin A Signaling,Ephrin Receptor Signaling,RhoA |

| | | | | | |
|---|---|---|---|---|---|
| | (and other miRNAs w/seed GUGCCUG) | | | | Signaling |
| MIMAT0004800 | miR-550a-5p (and other miRNAs w/seed GUGCCUG) | TargetScan Human | Moderate (predicted) | EPHA8 | Axonal Guidance Signaling,Ephrin A Signaling,Ephrin Receptor Signaling |
| MIMAT0004800 | miR-550a-5p (and other miRNAs w/seed GUGCCUG) | TargetScan Human | High (predicted) | ERBB2IP | Neuregulin Signaling |
| MIMAT0004800 | miR-550a-5p (and other miRNAs w/seed GUGCCUG) | TargetScan Human | Moderate (predicted) | ERBB3 | Agrin Interactions at Neuromuscular Junction,ErbB Signaling,ErbB2-ErbB3 Signaling,HER-2 Signaling in Breast Cancer,Neuregulin Signaling |
| MIMAT0004800 | miR-550a-5p (and other miRNAs w/seed GUGCCUG) | TargetScan Human | Moderate (predicted) | FASN | AMPK Signaling,Fatty Acid Biosynthesis Initiation II,FXR/RXR Activation,LXR/RXR Activation,Palmitate Biosynthesis I (Animals),PPARα/RXRα Activation,Stearate Biosynthesis I (Animals),TR/RXR Activation |
| MIMAT0004800 | miR-550a-5p (and other miRNAs w/seed GUGCCUG) | TargetScan Human | Moderate (predicted) | FGFR4 | Adipogenesis pathway,FGF Signaling,FXR/RXR Activation,Hepatic Cholestasis,Human Embryonic Stem Cell Pluripotency,NF-κB Signaling,PTEN Signaling,Regulation of the Epithelial-Mesenchymal Transition Pathway,STAT3 Pathway |
| MIMAT0004800 | miR-550a-5p (and other miRNAs w/seed GUGCCUG) | TargetScan Human | Moderate (predicted) | FLT4 | eNOS Signaling,Hepatic Fibrosis / Hepatic Stellate Cell Activation,IL-8 Signaling,NF-κB Signaling,Nitric Oxide Signaling in the Cardiovascular System,PTEN Signaling,STAT3 Pathway,VEGF Family Ligand-Receptor Interactions,VEGF Signaling |
| MIMAT0004800 | miR-550a-5p (and other miRNAs w/seed GUGCCUG) | TargetScan Human | Moderate (predicted) | FNBP1 | Actin Nucleation by ARP-WASP Complex,Cardiac Hypertrophy Signaling,Cholecystokinin/Gastrin-mediated Signaling,Colorectal Cancer Metastasis Signaling,CXCR4 Signaling,Germ Cell-Sertoli Cell Junction Signaling,Glioblastoma Multiforme Signaling,Glioma Invasiveness Signaling,Gαq Signaling,HMGB1 Signaling,IL-8 Signaling,ILK Signaling,Integrin Signaling,Molecular Mechanisms of Cancer,mTOR Signaling,phagosome formation,Phospholipase C Signaling,Production of Nitric Oxide and Reactive Oxygen Species in Macrophages,Regulation of Actin-based Motility by |

| | | | | | |
|---|---|---|---|---|---|
| | | | | | Rho,RhoGDI Signaling,Semaphorin Signaling in Neurons,Signaling by Rho Family GTPases,Sphingosine-1-phosphate Signaling,Tec Kinase Signaling,Thrombin Signaling |
| MIMAT0004800 | miR-550a-5p (and other miRNAs w/seed GUGCCUG) | TargetScan Human | Moderate (predicted) | FZD6 | Adipogenesis pathway,Axonal Guidance Signaling,Basal Cell Carcinoma Signaling,Colorectal Cancer Metastasis Signaling,Factors Promoting Cardiogenesis in Vertebrates,Glioblastoma Multiforme Signaling,Human Embryonic Stem Cell Pluripotency,Molecular Mechanisms of Cancer,Mouse Embryonic Stem Cell Pluripotency,Ovarian Cancer Signaling,PCP pathway,Regulation of the Epithelial-Mesenchymal Transition Pathway,Role of Macrophages, Fibroblasts and Endothelial Cells in Rheumatoid Arthritis,Role of NANOG in Mammalian Embryonic Stem Cell Pluripotency,Role of Osteoblasts, Osteoclasts and Chondrocytes in Rheumatoid Arthritis,Role of Wnt/GSK-3β Signaling in the Pathogenesis of Influenza,Wnt/Ca+ pathway,Wnt/β-catenin Signaling |
| MIMAT0004800 | miR-550a-5p (and other miRNAs w/seed GUGCCUG) | TargetScan Human | Moderate (predicted) | GABRA3 | GABA Receptor Signaling |
| MIMAT0004800 | miR-550a-5p (and other miRNAs w/seed GUGCCUG) | TargetScan Human | Moderate (predicted) | GABRA4 | GABA Receptor Signaling |
| MIMAT0004800 | miR-550a-5p (and other miRNAs w/seed GUGCCUG) | TargetScan Human | Moderate (predicted) | GABRB3 | GABA Receptor Signaling |
| MIMAT0004800 | miR-550a-5p (and other miRNAs w/seed GUGCCUG) | TargetScan Human | Moderate (predicted) | GHR | Growth Hormone Signaling,NF-κB Signaling,PPARα/RXRα Activation,PTEN Signaling,Role of JAK2 in Hormone-like Cytokine Signaling,STAT3 Pathway |
| MIMAT0004800 | miR-550a-5p (and other miRNAs w/seed GUGCCUG) | TargetScan Human | Moderate (predicted) | GNB4 | Androgen Signaling,Antiproliferative Role of Somatostatin Receptor 2,Axonal Guidance Signaling,Breast Cancer Regulation by Stathmin1,Cardiac Hypertrophy Signaling,Cardiac β-adrenergic Signaling,CCR3 Signaling in Eosinophils,CCR5 Signaling in Macrophages,Colorectal Cancer Metastasis Signaling,CREB Signaling in Neurons,CXCR4 Signaling,Ephrin B Signaling,Ephrin |

| | | | | | |
|---|---|---|---|---|---|
| | | | | | Receptor Signaling,fMLP Signaling in Neutrophils,G Beta Gamma Signaling,G Protein Signaling Mediated by Tubby,Gαi Signaling,Gαq Signaling,Gαs Signaling,Huntington's Disease Signaling,IL-1 Signaling,IL-8 Signaling,P2Y Purigenic Receptor Signaling Pathway,Phospholipase C Signaling,Protein Kinase A Signaling,Relaxin Signaling,RhoGDI Signaling,Role of NFAT in Cardiac Hypertrophy,Role of NFAT in Regulation of the Immune Response,Signaling by Rho Family GTPases,Tec Kinase Signaling,Thrombin Signaling,α-Adrenergic Signaling |
| MIMAT0004800 | miR-550a-5p (and other miRNAs w/seed GUGCCUG) | TargetScan Human | Moderate (predicted) | GNG4 | Androgen Signaling,Antiproliferative Role of Somatostatin Receptor 2,Axonal Guidance Signaling,Breast Cancer Regulation by Stathmin1,Cardiac Hypertrophy Signaling,Cardiac β-adrenergic Signaling,CCR3 Signaling in Eosinophils,CCR5 Signaling in Macrophages,Colorectal Cancer Metastasis Signaling,CREB Signaling in Neurons,CXCR4 Signaling,Ephrin B Signaling,Ephrin Receptor Signaling,fMLP Signaling in Neutrophils,G Beta Gamma Signaling,G Protein Signaling Mediated by Tubby,GPCR-Mediated Nutrient Sensing in Enteroendocrine Cells,Gαi Signaling,Gαq Signaling,Gαs Signaling,Huntington's Disease Signaling,IL-1 Signaling,IL-8 Signaling,P2Y Purigenic Receptor Signaling Pathway,Phospholipase C Signaling,Protein Kinase A Signaling,Relaxin Signaling,RhoGDI Signaling,Role of NFAT in Cardiac Hypertrophy,Role of NFAT in Regulation of the Immune Response,Signaling by Rho Family GTPases,Tec Kinase Signaling,Thrombin Signaling,α-Adrenergic Signaling |
| MIMAT0004800 | miR-550a-5p (and other miRNAs w/seed GUGCCUG) | TargetScan Human | Moderate (predicted) | GRINA | Amyotrophic Lateral Sclerosis Signaling,Calcium Signaling,Circadian Rhythm Signaling,Dopamine-DARPP32 Feedback in cAMP Signaling,Ephrin Receptor Signaling,Glutamate Receptor Signaling,Neuropathic Pain Signaling In Dorsal Horn Neurons,nNOS Signaling in Neurons,Synaptic Long Term Potentiation |
| MIMAT0004800 | miR-550a-5p (and other miRNAs w/seed GUGCCUG) | TargetScan Human | Moderate (predicted) | GSK3B | 14-3-3-mediated Signaling,Amyloid Processing,Axonal Guidance Signaling,B Cell Receptor Signaling,Basal Cell Carcinoma Signaling,Cardiac Hypertrophy Signaling,Cdc42 Signaling,Cell Cycle: G1/S Checkpoint Regulation,Colorectal Cancer Metastasis Signaling,Cyclins and Cell Cycle Regulation,Docosahexaenoic Acid (DHA) Signaling,EIF2 Signaling,Endometrial Cancer Signaling,ErbB Signaling,ErbB2-ErbB3 Signaling,Factors Promoting Cardiogenesis in Vertebrates,Glioblastoma Multiforme Signaling,Gαq Signaling,HER-2 Signaling in Breast Cancer,Human Embryonic Stem Cell Pluripotency,IL-17 Signaling,IL-17A Signaling in Airway Cells,IL-17A Signaling in Fibroblasts,ILK Signaling,Insulin Receptor Signaling,Integrin Signaling,Molecular Mechanisms of Cancer,Mouse Embryonic Stem Cell Pluripotency,NF-κB Signaling,NRF2-mediated Oxidative Stress Response,Ovarian Cancer Signaling,p53 Signaling,PI3K/AKT Signaling,Prostate |

| | | | | | |
|---|---|---|---|---|---|
| | | | | | Cancer Signaling,Protein Kinase A Signaling,PTEN Signaling,Reelin Signaling in Neurons,Regulation of the Epithelial-Mesenchymal Transition Pathway,Role of Macrophages, Fibroblasts and Endothelial Cells in Rheumatoid Arthritis,Role of NANOG in Mammalian Embryonic Stem Cell Pluripotency,Role of NFAT in Cardiac Hypertrophy,Role of NFAT in Regulation of the Immune Response,Role of Osteoblasts, Osteoclasts and Chondrocytes in Rheumatoid Arthritis,Role of PI3K/AKT Signaling in the Pathogenesis of Influenza,Role of Wnt/GSK-3β Signaling in the Pathogenesis of Influenza,Sertoli Cell-Sertoli Cell Junction Signaling,Sonic Hedgehog Signaling,Wnt/Ca+ pathway,Wnt/β-catenin Signaling |
| MIMAT0004800 | miR-550a-5p (and other miRNAs w/seed GUGCCUG) | TargetScan Human | Moderate (predicted) | HDAC11 | Adipogenesis pathway,Calcium Signaling,Cell Cycle: G1/S Checkpoint Regulation,Chronic Myeloid Leukemia Signaling,Cyclins and Cell Cycle Regulation,Hereditary Breast Cancer Signaling,Huntington's Disease Signaling,Phospholipase C Signaling,Role of NFAT in Cardiac Hypertrophy,Telomerase Signaling |
| MIMAT0004800 | miR-550a-5p (and other miRNAs w/seed GUGCCUG) | TargetScan Human | Moderate (predicted) | HNF4A | Acyl-CoA Hydrolysis,AMPK Signaling,FXR/RXR Activation,Hepatic Cholestasis,Maturity Onset Diabetes of Young (MODY) Signaling,PXR/RXR Activation,Stearate Biosynthesis I (Animals),TGF-β Signaling,Transcriptional Regulatory Network in Embryonic Stem Cells |
| MIMAT0004800 | miR-550a-5p (and other miRNAs w/seed GUGCCUG) | TargetScan Human | Moderate (predicted) | HTR2A | G-Protein Coupled Receptor Signaling,Gap Junction Signaling,Gαq Signaling,Serotonin Receptor Signaling |
| MIMAT0004800 | miR-550a-5p (and other miRNAs w/seed GUGCCUG) | TargetScan Human | High (predicted) | HTR5A | cAMP-mediated signaling,G-Protein Coupled Receptor Signaling,Gαs Signaling,Serotonin Receptor Signaling |
| MIMAT0004800 | miR-550a-5p (and other miRNAs w/seed GUGCCUG) | TargetScan Human | Moderate (predicted) | IGF1R | Cardiac Hypertrophy Signaling,Estrogen-Dependent Breast Cancer Signaling,Glioblastoma Multiforme Signaling,Glioma Signaling,Growth Hormone Signaling,Hepatic Fibrosis / Hepatic Stellate Cell Activation,Huntington's Disease Signaling,IGF-1 Signaling,Myc Mediated Apoptosis Signaling,NF-κB Signaling,PTEN Signaling,RhoA Signaling,Role of NFAT in Cardiac Hypertrophy,STAT3 Pathway,Synaptic Long Term Depression |
| MIMAT0004800 | miR-550a-5p (and other | TargetScan Human | Moderate (predicted) | IL13 | Airway Inflammation in Asthma,Differential Regulation of Cytokine Production in Intestinal Epithelial Cells by IL-17A and IL-17F,Differential |

| | | | | | |
|---|---|---|---|---|---|
| | miRNAs w/seed GUGCCUG) | | | | Regulation of Cytokine Production in Macrophages and T Helper Cells by IL-17A and IL-17F,Fc Epsilon RI Signaling,Glucocorticoid Receptor Signaling,Hepatic Cholestasis,HMGB1 Signaling,Role of Cytokines in Mediating Communication between Immune Cells,Role of Pattern Recognition Receptors in Recognition of Bacteria and Viruses,T Helper Cell Differentiation |
| MIMAT0004800 | miR-550a-5p (and other miRNAs w/seed GUGCCUG) | TargetScan Human | High (predicted) | INPP5B | 1D-myo-inositol Hexakisphosphate Biosynthesis II (Mammalian),3-phosphoinositide Degradation,B Cell Receptor Signaling,D-myo-inositol (1,3,4)-trisphosphate Biosynthesis,D-myo-inositol (1,4,5)-trisphosphate Degradation,Fc Epsilon RI Signaling,IL-4 Signaling,Insulin Receptor Signaling,Natural Killer Cell Signaling,PDGF Signaling,PI3K/AKT Signaling,PTEN Signaling,Superpathway of D-myo-inositol (1,4,5)-trisphosphate Metabolism,Superpathway of Inositol Phosphate Compounds |
| MIMAT0004800 | miR-550a-5p (and other miRNAs w/seed GUGCCUG) | TargetScan Human | Moderate (predicted) | INPP5K | 1D-myo-inositol Hexakisphosphate Biosynthesis II (Mammalian),3-phosphoinositide Degradation,B Cell Receptor Signaling,D-myo-inositol (1,3,4)-trisphosphate Biosynthesis,D-myo-inositol (1,4,5)-trisphosphate Degradation,Fc Epsilon RI Signaling,IL-4 Signaling,Insulin Receptor Signaling,Natural Killer Cell Signaling,PDGF Signaling,PI3K/AKT Signaling,PTEN Signaling,Superpathway of D-myo-inositol (1,4,5)-trisphosphate Metabolism,Superpathway of Inositol Phosphate Compounds |
| MIMAT0004800 | miR-550a-5p (and other miRNAs w/seed GUGCCUG) | TargetScan Human | Moderate (predicted) | ITGA2 | Actin Cytoskeleton Signaling,Actin Nucleation by ARP-WASP Complex,Agranulocyte Adhesion and Diapedesis,Agrin Interactions at Neuromuscular Junction,Axonal Guidance Signaling,Caveolar-mediated Endocytosis Signaling,Cdc42 Signaling,CDK5 Signaling,Ephrin Receptor Signaling,ERK/MAPK Signaling,FAK Signaling,Germ Cell-Sertoli Cell Junction Signaling,Granulocyte Adhesion and Diapedesis,HGF Signaling,Integrin Signaling,Leukocyte Extravasation Signaling,Molecular Mechanisms of Cancer,Neuregulin Signaling,NF-κB Activation by Viruses,PAK Signaling,Paxillin Signaling,phagosome formation,Phospholipase C Signaling,PI3K/AKT Signaling,PTEN Signaling,Rac Signaling,Reelin Signaling in Neurons,Regulation of Actin-based Motility by Rho,Regulation of Cellular Mechanics by Calpain Protease,Regulation of eIF4 and p70S6K Signaling,RhoGDI Signaling,Role of Osteoblasts, Osteoclasts and Chondrocytes in Rheumatoid Arthritis,Sertoli Cell-Sertoli Cell Junction Signaling,Signaling by Rho Family GTPases,Tec Kinase Signaling,Virus Entry via Endocytic Pathways |
| MIMAT0004800 | miR-550a-5p (and other miRNAs w/seed | TargetScan Human | Moderate (predicted) | ITGA3 | Actin Cytoskeleton Signaling,Actin Nucleation by ARP-WASP Complex,Agranulocyte Adhesion and Diapedesis,Agrin Interactions at Neuromuscular Junction,Axonal Guidance Signaling,Caveolar-mediated |

| | | | | | |
|---|---|---|---|---|---|
| | GUGCCUG) | | | | Endocytosis Signaling,Cdc42 Signaling,CDK5 Signaling,Ephrin Receptor Signaling,ERK/MAPK Signaling,FAK Signaling,Germ Cell-Sertoli Cell Junction Signaling,Granulocyte Adhesion and Diapedesis,HGF Signaling,Integrin Signaling,Leukocyte Extravasation Signaling,Molecular Mechanisms of Cancer,Neuregulin Signaling,NF-κB Activation by Viruses,PAK Signaling,Paxillin Signaling,phagosome formation,Phospholipase C Signaling,PI3K/AKT Signaling,PTEN Signaling,Rac Signaling,Reelin Signaling in Neurons,Regulation of Actin-based Motility by Rho,Regulation of Cellular Mechanics by Calpain Protease,Regulation of eIF4 and p70S6K Signaling,RhoGDI Signaling,Role of Osteoblasts, Osteoclasts and Chondrocytes in Rheumatoid Arthritis,Role of Tissue Factor in Cancer,Sertoli Cell-Sertoli Cell Junction Signaling,Signaling by Rho Family GTPases,Tec Kinase Signaling,Virus Entry via Endocytic Pathways |
| MIMAT0004800 | miR-550a-5p (and other miRNAs w/seed GUGCCUG) | TargetScan Human | Moderate (predicted) | KCNJ9 | Dopamine-DARPP32 Feedback in cAMP Signaling,G Beta Gamma Signaling |
| MIMAT0004800 | miR-550a-5p (and other miRNAs w/seed GUGCCUG) | TargetScan Human | Moderate (predicted) | KCNN3 | Cellular Effects of Sildenafil (Viagra),GABA Receptor Signaling,Neuropathic Pain Signaling In Dorsal Horn Neurons |
| MIMAT0004800 | miR-550a-5p (and other miRNAs w/seed GUGCCUG) | TargetScan Human | Moderate (predicted) | LPAR2 | eNOS Signaling,Gα12/13 Signaling,RhoA Signaling |
| MIMAT0004800 | miR-550a-5p (and other miRNAs w/seed GUGCCUG) | TargetScan Human | Moderate (predicted) | MAP3K6 | B Cell Receptor Signaling,Cardiac Hypertrophy Signaling,CD27 Signaling in Lymphocytes,Germ Cell-Sertoli Cell Junction Signaling,GNRH Signaling,HGF Signaling,NGF Signaling,PKCθ Signaling in T Lymphocytes,Production of Nitric Oxide and Reactive Oxygen Species in Macrophages,Pyridoxal 5'-phosphate Salvage Pathway,RANK Signaling in Osteoclasts,Salvage Pathways of Pyrimidine Ribonucleotides,Sertoli Cell-Sertoli Cell Junction Signaling,Xenobiotic Metabolism Signaling |
| MIMAT0004800 | miR-550a-5p (and other miRNAs w/seed GUGCCUG) | TargetScan Human | Moderate (predicted) | MAP4K4 | Apoptosis Signaling,Creatine-phosphate Biosynthesis,Death Receptor Signaling,Ephrin Receptor Signaling,IL-10 Signaling,IL-6 Signaling,IL-8 Signaling,NF-κB Signaling,PPAR Signaling,PPARα/RXRα Activation,SAPK/JNK Signaling,Toll-like Receptor Signaling |

| | | | | | |
|---|---|---|---|---|---|
| MIMAT0004800 | miR-550a-5p (and other miRNAs w/seed GUGCCUG) | TargetScan Human | Moderate (predicted) | MAPK10 | 14-3-3-mediated Signaling,4-1BB Signaling in T Lymphocytes,Activation of IRF by Cytosolic Pattern Recognition Receptors,Agrin Interactions at Neuromuscular Junction,Antioxidant Action of Vitamin C,April Mediated Signaling,ATM Signaling,B Cell Activating Factor Signaling,BMP signaling pathway,Cardiac Hypertrophy Signaling,CCR5 Signaling in Macrophages,CD27 Signaling in Lymphocytes,CD28 Signaling in T Helper Cells,CD40 Signaling,Cdc42 Signaling,CDK5 Signaling,Cholecystokinin/Gastrin-mediated Signaling,Colorectal Cancer Metastasis Signaling,CXCR4 Signaling,Dendritic Cell Maturation,Endothelin-1 Signaling,ErbB Signaling,Fc Epsilon RI Signaling,FcγRIIB Signaling in B Lymphocytes,FXR/RXR Activation,GDNF Family Ligand-Receptor Interactions,Germ Cell-Sertoli Cell Junction Signaling,Glucocorticoid Receptor Signaling,GNRH Signaling,Gα12/13 Signaling,Hepatic Cholestasis,HGF Signaling,HIF1α Signaling,HMGB1 Signaling,IL-1 Signaling,IL-12 Signaling and Production in Macrophages,IL-17 Signaling,IL-17A Signaling in Airway Cells,IL-17A Signaling in Gastric Cells,IL-22 Signaling,IL-6 Signaling,IL-8 Signaling,ILK Signaling,Induction of Apoptosis by HIV1,Inhibition of Angiogenesis by TSP1,Leukocyte Extravasation Signaling,LPS-stimulated MAPK Signaling,MIF Regulation of Innate Immunity,Mitochondrial Dysfunction,Molecular Mechanisms of Cancer,Myc Mediated Apoptosis Signaling,NGF Signaling,OX40 Signaling Pathway,PAK Signaling,Pancreatic Adenocarcinoma Signaling,Paxillin Signaling,PCP pathway,Production of Nitric Oxide and Reactive Oxygen Species in Macrophages,RANK Signaling in Osteoclasts,RAR Activation,Reelin Signaling in Neurons,Regulation of IL-2 Expression in Activated and Anergic T Lymphocytes,Renin-Angiotensin Signaling,Role of IL-17A in Arthritis,Role of JAK family kinases in IL-6-type Cytokine Signaling,Role of MAPK Signaling in the Pathogenesis of Influenza,Role of NFAT in Cardiac Hypertrophy,Role of Osteoblasts, Osteoclasts and Chondrocytes in Rheumatoid Arthritis,Role of Pattern Recognition Receptors in Recognition of Bacteria and Viruses,SAPK/JNK Signaling,Sertoli Cell-Sertoli Cell Junction Signaling,Signaling by Rho Family GTPases,STAT3 Pathway,Tec Kinase Signaling,Type I Diabetes Mellitus Signaling,Type II Diabetes Mellitus Signaling,UVA-Induced MAPK Signaling,UVB-Induced MAPK Signaling,UVC-Induced MAPK Signaling |
| MIMAT0004800 | miR-550a-5p (and other miRNAs w/seed GUGCCUG) | TargetScan Human | Moderate (predicted) | MDM2 | Aryl Hydrocarbon Receptor Signaling,ATM Signaling,Bladder Cancer Signaling,Cell Cycle: G1/S Checkpoint Regulation,Cell Cycle: G2/M DNA Damage Checkpoint Regulation,Chronic Myeloid Leukemia Signaling,Clathrin-mediated Endocytosis Signaling,Glioblastoma Multiforme Signaling,Glioma Signaling,HER-2 Signaling in Breast Cancer,HIF1α Signaling,Hypoxia Signaling in the Cardiovascular System,Melanoma Signaling,Molecular Mechanisms of Cancer,p53 Signaling,Pancreatic Adenocarcinoma Signaling,PI3K/AKT |

| | | | | | |
|---|---|---|---|---|---|
| | | | | | Signaling,Prostate Cancer Signaling,Protein Ubiquitination Pathway,Role of p14/p19ARF in Tumor Suppression,TR/RXR Activation,Wnt/β-catenin Signaling |
| MIMAT0004800 | miR-550a-5p (and other miRNAs w/seed GUGCCUG) | TargetScan Human | High (predicted) | MEF2A | Calcium Signaling,Cardiac Hypertrophy Signaling,Cholecystokinin/Gastrin-mediated Signaling,Corticotropin Releasing Hormone Signaling,ERK5 Signaling,Gα12/13 Signaling,p38 MAPK Signaling,Phospholipase C Signaling,Role of NFAT in Cardiac Hypertrophy,Role of NFAT in Regulation of the Immune Response,Role of Oct4 in Mammalian Embryonic Stem Cell Pluripotency |
| MIMAT0004800 | miR-550a-5p (and other miRNAs w/seed GUGCCUG) | TargetScan Human | High (predicted) | MKNK2 | ERK/MAPK Signaling,p38 MAPK Signaling |
| MIMAT0004800 | miR-550a-5p (and other miRNAs w/seed GUGCCUG) | TargetScan Human | High (predicted) | MYH2 | Actin Cytoskeleton Signaling,Agranulocyte Adhesion and Diapedesis,Calcium Signaling,Cellular Effects of Sildenafil (Viagra),Epithelial Adherens Junction Signaling,Hepatic Fibrosis / Hepatic Stellate Cell Activation,ILK Signaling,Protein Kinase A Signaling,Tight Junction Signaling |
| MIMAT0004800 | miR-550a-5p (and other miRNAs w/seed GUGCCUG) | TargetScan Human | High (predicted) | NEUROG1 | Transcriptional Regulatory Network in Embryonic Stem Cells |
| MIMAT0004800 | miR-550a-5p (and other miRNAs w/seed GUGCCUG) | TargetScan Human | Moderate (predicted) | NFATC1 | April Mediated Signaling,Axonal Guidance Signaling,B Cell Activating Factor Signaling,B Cell Receptor Signaling,Calcium Signaling,CD28 Signaling in T Helper Cells,ERK/MAPK Signaling,fMLP Signaling in Neutrophils,Glucocorticoid Receptor Signaling,Gαq Signaling,iCOS-iCOSL Signaling in T Helper Cells,IL-4 Signaling,Netrin Signaling,Nur77 Signaling in T Lymphocytes,Phospholipase C Signaling,PI3K Signaling in B Lymphocytes,PKCθ Signaling in T Lymphocytes,Protein Kinase A Signaling,RANK Signaling in Osteoclasts,Regulation of IL-2 Expression in Activated and Anergic T Lymphocytes,Role of Macrophages, Fibroblasts and Endothelial Cells in Rheumatoid Arthritis,Role of NFAT in Regulation of the Immune Response,Role of Osteoblasts, Osteoclasts and Chondrocytes in Rheumatoid Arthritis,SAPK/JNK Signaling,Systemic Lupus Erythematosus Signaling,T Cell Receptor Signaling,Wnt/Ca+ pathway |
| MIMAT0004800 | miR-550a-5p | TargetScan Human | Moderate (predicted) | NGEF | Axonal Guidance Signaling,Ephrin A Signaling,Ephrin Receptor Signaling,RhoA |

| | | | | | |
|---|---|---|---|---|---|
| | (and other miRNAs w/seed GUGCCUG) | | | | Signaling |
| MIMAT0004800 | miR-550a-5p (and other miRNAs w/seed GUGCCUG) | TargetScan Human | Moderate (predicted) | NPR1 | Antiproliferative Role of Somatostatin Receptor 2,Corticotropin Releasing Hormone Signaling,Gap Junction Signaling,Relaxin Signaling,Sperm Motility,Synaptic Long Term Depression |
| MIMAT0004800 | miR-550a-5p (and other miRNAs w/seed GUGCCUG) | TargetScan Human | Moderate (predicted) | NRIP1 | Aryl Hydrocarbon Receptor Signaling,Estrogen Receptor Signaling,Glucocorticoid Receptor Signaling,PPAR Signaling,RAR Activation,Xenobiotic Metabolism Signaling |
| MIMAT0004800 | miR-550a-5p (and other miRNAs w/seed GUGCCUG) | TargetScan Human | High (predicted) | OPN1MW (includes others) | Phototransduction Pathway |
| MIMAT0004800 | miR-550a-5p (and other miRNAs w/seed GUGCCUG) | TargetScan Human | Moderate (predicted) | P2RX7 | Gustation Pathway |
| MIMAT0004800 | miR-550a-5p (and other miRNAs w/seed GUGCCUG) | TargetScan Human | Moderate (predicted) | P2RY11 | Gustation Pathway,P2Y Purigenic Receptor Signaling Pathway |
| MIMAT0004800 | miR-550a-5p (and other miRNAs w/seed GUGCCUG) | TargetScan Human | Moderate (predicted) | P2RY14 | cAMP-mediated signaling,G-Protein Coupled Receptor Signaling,Gustation Pathway,Gαi Signaling |
| MIMAT0004800 | miR-550a-5p (and other miRNAs w/seed GUGCCUG) | TargetScan Human | Moderate (predicted) | P2RY8 | Gustation Pathway |
| MIMAT0004800 | miR-550a-5p (and other miRNAs w/seed | TargetScan Human | Moderate (predicted) | PBX1 | Glucocorticoid Receptor Signaling |

| | | | | | |
|---|---|---|---|---|---|
| | GUGCCUG) | | | | |
| MIMAT0004800 | miR-550a-5p (and other miRNAs w/seed GUGCCUG) | TargetScan Human | High (predicted) | PDGFRA | Glioblastoma Multiforme Signaling,Glioma Signaling,Hepatic Fibrosis / Hepatic Stellate Cell Activation,Human Embryonic Stem Cell Pluripotency,NF-κB Signaling,PAK Signaling,PDGF Signaling,PPAR Signaling,PTEN Signaling,Sphingosine-1-phosphate Signaling,STAT3 Pathway |
| MIMAT0004800 | miR-550a-5p (and other miRNAs w/seed GUGCCUG) | TargetScan Human | Moderate (predicted) | PGP | Protein Kinase A Signaling |
| MIMAT0004800 | miR-550a-5p (and other miRNAs w/seed GUGCCUG) | TargetScan Human | Moderate (predicted) | PLCD3 | 14-3-3-mediated Signaling,Aldosterone Signaling in Epithelial Cells,Antioxidant Action of Vitamin C,Axonal Guidance Signaling,Cardiac Hypertrophy Signaling,Cellular Effects of Sildenafil (Viagra),CREB Signaling in Neurons,D-myo-inositol (1,4,5)-Trisphosphate Biosynthesis,D-myo-inositol-5-phosphate Metabolism,Dendritic Cell Maturation,Dopamine-DARPP32 Feedback in cAMP Signaling,Endothelin-1 Signaling,Gap Junction Signaling,Glioblastoma Multiforme Signaling,GPCR-Mediated Integration of Enteroendocrine Signaling Exemplified by an L Cell,GPCR-Mediated Nutrient Sensing in Enteroendocrine Cells,Leptin Signaling in Obesity,Melatonin Signaling,Neuropathic Pain Signaling In Dorsal Horn Neurons,P2Y Purigenic Receptor Signaling Pathway,p70S6K Signaling,phagosome formation,Phospholipases,PI3K Signaling in B Lymphocytes,PPARα/RXRα Activation,Protein Kinase A Signaling,Role of Macrophages, Fibroblasts and Endothelial Cells in Rheumatoid Arthritis,Role of NFAT in Cardiac Hypertrophy,Sperm Motility,Sphingosine-1-phosphate Signaling,Superpathway of Inositol Phosphate Compounds,Synaptic Long Term Depression,Synaptic Long Term Potentiation,Thrombin Signaling,UVA-Induced MAPK Signaling,Wnt/Ca+ pathway |
| MIMAT0004800 | miR-550a-5p (and other miRNAs w/seed GUGCCUG) | TargetScan Human | Moderate (predicted) | PLD1 | Antioxidant Action of Vitamin C,Choline Biosynthesis III,Endothelin-1 Signaling,Fcγ Receptor-mediated Phagocytosis in Macrophages and Monocytes,Gαq Signaling,IL-8 Signaling,mTOR Signaling,p70S6K Signaling,Pancreatic Adenocarcinoma Signaling,Phospholipase C Signaling,Phospholipases,Rac Signaling,RhoA Signaling,Signaling by Rho Family GTPases |
| MIMAT0004800 | miR-550a-5p (and other miRNAs w/seed | TargetScan Human | Moderate (predicted) | PLXNC1 | Axonal Guidance Signaling |

| | | | | | |
|---|---|---|---|---|---|
| | GUGCCUG) | | | | |
| MIMAT0004800 | miR-550a-5p (and other miRNAs w/seed GUGCCUG) | TargetScan Human | High (predicted) | PNPLA2 | Docosahexaenoic Acid (DHA) Signaling,PEDF Signaling,Retinol Biosynthesis,Triacylglycerol Degradation |
| MIMAT0004800 | miR-550a-5p (and other miRNAs w/seed GUGCCUG) | TargetScan Human | High (predicted),Moderate (predicted) | POLR2J2/POLR2J3 | Androgen Signaling,Assembly of RNA Polymerase II Complex,CREB Signaling in Neurons,Estrogen Receptor Signaling,Glucocorticoid Receptor Signaling,Hereditary Breast Cancer Signaling,Huntington's Disease Signaling,Nucleotide Excision Repair Pathway |
| MIMAT0004800 | miR-550a-5p (and other miRNAs w/seed GUGCCUG) | TargetScan Human | Moderate (predicted) | POLR3D | Assembly of RNA Polymerase III Complex,Role of p14/p19ARF in Tumor Suppression |
| MIMAT0004800 | miR-550a-5p (and other miRNAs w/seed GUGCCUG) | TargetScan Human | Moderate (predicted) | PPP2R1B | AMPK Signaling,Breast Cancer Regulation by Stathmin1,Cardiac β-adrenergic Signaling,CDK5 Signaling,Cell Cycle Regulation by BTG Family Proteins,Ceramide Signaling,CTLA4 Signaling in Cytotoxic T Lymphocytes,Cyclins and Cell Cycle Regulation,Dopamine Receptor Signaling,Dopamine-DARPP32 Feedback in cAMP Signaling,ERK/MAPK Signaling,HIPPO signaling,ILK Signaling,Mitotic Roles of Polo-Like Kinase,mTOR Signaling,p70S6K Signaling,PI3K/AKT Signaling,Production of Nitric Oxide and Reactive Oxygen Species in Macrophages,Regulation of eIF4 and p70S6K Signaling,Role of CHK Proteins in Cell Cycle Checkpoint Control,Synaptic Long Term Depression,Telomerase Signaling,Tight Junction Signaling,Wnt/β-catenin Signaling,Xenobiotic Metabolism Signaling |
| MIMAT0004800 | miR-550a-5p (and other miRNAs w/seed GUGCCUG) | TargetScan Human | Moderate (predicted) | PRKACG | AMPK Signaling,Amyloid Processing,Androgen Signaling,Axonal Guidance Signaling,BMP signaling pathway,Breast Cancer Regulation by Stathmin1,Calcium Signaling,cAMP-mediated signaling,Cardiac Hypertrophy Signaling,Cardiac β-adrenergic Signaling,CDK5 Signaling,Cellular Effects of Sildenafil (Viagra),Colorectal Cancer Metastasis Signaling,Corticotropin Releasing Hormone Signaling,CREB Signaling in Neurons,Dopamine Receptor Signaling,Dopamine-DARPP32 Feedback in cAMP Signaling,eNOS Signaling,ERK/MAPK Signaling,G Beta Gamma Signaling,G-Protein Coupled Receptor Signaling,Gap Junction Signaling,Glucocorticoid Receptor Signaling,GNRH Signaling,GPCR-Mediated Integration of Enteroendocrine Signaling Exemplified by an L Cell,GPCR-Mediated Nutrient Sensing in Enteroendocrine Cells,Gustation Pathway,Gαi Signaling,Gαs Signaling,Hepatic |

| | | | | | |
|---|---|---|---|---|---|
| | | | | | Cholestasis,IGF-1 Signaling,IL-1 Signaling,Insulin Receptor Signaling,Leptin Signaling in Obesity,Melanocyte Development and Pigmentation Signaling,Melatonin Signaling,Molecular Mechanisms of Cancer,Netrin Signaling,Neuropathic Pain Signaling In Dorsal Horn Neurons,Neuroprotective Role of THOP1 in Alzheimer's Disease,NF-κB Signaling,Nitric Oxide Signaling in the Cardiovascular System,Ovarian Cancer Signaling,P2Y Purigenic Receptor Signaling Pathway,Phototransduction Pathway,PPARα/RXRα Activation,Protein Kinase A Signaling,PXR/RXR Activation,RAR Activation,Relaxin Signaling,Renin-Angiotensin Signaling,Role of NFAT in Cardiac Hypertrophy,Sertoli Cell-Sertoli Cell Junction Signaling,Sonic Hedgehog Signaling,Sperm Motility,Synaptic Long Term Potentiation,Tight Junction Signaling,α-Adrenergic Signaling |
| MIMAT0004800 | miR-550a-5p (and other miRNAs w/seed GUGCCUG) | TargetScan Human | Moderate (predicted) | PTK2B | B Cell Receptor Signaling,CCR5 Signaling in Macrophages,Chemokine Signaling,Cholecystokinin/Gastrin-mediated Signaling,ERK/MAPK Signaling,Fcγ Receptor-mediated Phagocytosis in Macrophages and Monocytes,G-Protein Coupled Receptor Signaling,Gα12/13 Signaling,Gαq Signaling,IL-15 Production,IL-2 Signaling,IL-8 Signaling,Leukocyte Extravasation Signaling,PAK Signaling,Paxillin Signaling,Protein Kinase A Signaling,Rac Signaling,RANK Signaling in Osteoclasts,Renin-Angiotensin Signaling,RhoA Signaling,Role of JAK1 and JAK3 in γc Cytokine Signaling,Role of Osteoblasts, Osteoclasts and Chondrocytes in Rheumatoid Arthritis,Role of Tissue Factor in Cancer,Signaling by Rho Family GTPases,Sperm Motility,Sphingosine-1-phosphate Signaling,Tec Kinase Signaling,VEGF Signaling |
| MIMAT0004800 | miR-550a-5p (and other miRNAs w/seed GUGCCUG) | TargetScan Human | Moderate (predicted) | PTPN3 | Protein Kinase A Signaling |
| MIMAT0004800 | miR-550a-5p (and other miRNAs w/seed GUGCCUG) | TargetScan Human | Moderate (predicted) | RAPGEF1 | Ephrin Receptor Signaling,Epithelial Adherens Junction Signaling,ERK/MAPK Signaling,HGF Signaling,IL-3 Signaling,Insulin Receptor Signaling,Integrin Signaling,Molecular Mechanisms of Cancer,Renal Cell Carcinoma Signaling |
| MIMAT0004800 | miR-550a-5p (and other miRNAs w/seed GUGCCUG) | TargetScan Human | Moderate (predicted) | RGS9BP | Phototransduction Pathway |

| | | | | | |
|---|---|---|---|---|---|
| MIMAT0004800 | miR-550a-5p (and other miRNAs w/seed GUGCCUG) | TargetScan Human | High (predicted) | SCNN1G | Aldosterone Signaling in Epithelial Cells,Gustation Pathway,Insulin Receptor Signaling |
| MIMAT0004800 | miR-550a-5p (and other miRNAs w/seed GUGCCUG) | TargetScan Human | Moderate (predicted) | SEMA4C | Axonal Guidance Signaling |
| MIMAT0004800 | miR-550a-5p (and other miRNAs w/seed GUGCCUG) | TargetScan Human | Moderate (predicted) | SEMA5A | Axonal Guidance Signaling |
| MIMAT0004800 | miR-550a-5p (and other miRNAs w/seed GUGCCUG) | TargetScan Human | Moderate (predicted) | SLC1A2 | Amyotrophic Lateral Sclerosis Signaling,Glutamate Receptor Signaling |
| MIMAT0004800 | miR-550a-5p (and other miRNAs w/seed GUGCCUG) | TargetScan Human | Moderate (predicted) | SLC2A1 | AMPK Signaling,Antioxidant Action of Vitamin C,HIF1α Signaling,Renal Cell Carcinoma Signaling,TR/RXR Activation,Vitamin-C Transport |
| MIMAT0004800 | miR-550a-5p (and other miRNAs w/seed GUGCCUG) | TargetScan Human | Moderate (predicted) | SMPD3 | Ceramide Signaling,NGF Signaling,Sphingomyelin Metabolism,Sphingosine-1-phosphate Signaling,Type II Diabetes Mellitus Signaling,UVA-Induced MAPK Signaling,UVC-Induced MAPK Signaling |
| MIMAT0004800 | miR-550a-5p (and other miRNAs w/seed GUGCCUG) | TargetScan Human | High (predicted) | SOCS4 | Acute Phase Response Signaling,Growth Hormone Signaling,IGF-1 Signaling,JAK/Stat Signaling,Prolactin Signaling,Role of JAK2 in Hormone-like Cytokine Signaling,STAT3 Pathway,Type I Diabetes Mellitus Signaling,Type II Diabetes Mellitus Signaling |
| MIMAT0004800 | miR-550a-5p (and other miRNAs w/seed GUGCCUG) | TargetScan Human | Moderate (predicted) | SPR | Dopamine Receptor Signaling,Serotonin Receptor Signaling,Tetrahydrobiopterin Biosynthesis I,Tetrahydrobiopterin Biosynthesis II |
| MIMAT0004800 | miR-550a-5p (and other | TargetScan Human | Moderate (predicted) | SYNJ2 | 1D-myo-inositol Hexakisphosphate Biosynthesis II (Mammalian),3-phosphoinositide Degradation,B Cell Receptor Signaling,D-myo-inositol |

| | | | | | |
|---|---|---|---|---|---|
| | miRNAs w/seed GUGCCUG) | | | | (1,3,4)-trisphosphate Biosynthesis,D-myo-inositol (1,4,5)-trisphosphate Degradation,Fc Epsilon RI Signaling,IL-4 Signaling,Insulin Receptor Signaling,Natural Killer Cell Signaling,PDGF Signaling,PI3K/AKT Signaling,PTEN Signaling,Superpathway of D-myo-inositol (1,4,5)-trisphosphate Metabolism,Superpathway of Inositol Phosphate Compounds |
| MIMAT0004800 | miR-550a-5p (and other miRNAs w/seed GUGCCUG) | TargetScan Human | Moderate (predicted) | TAF11 | Assembly of RNA Polymerase II Complex,Estrogen Receptor Signaling,Glucocorticoid Receptor Signaling |
| MIMAT0004800 | miR-550a-5p (and other miRNAs w/seed GUGCCUG) | TargetScan Human | Moderate (predicted) | TAF5L | Assembly of RNA Polymerase II Complex,Estrogen Receptor Signaling,Glucocorticoid Receptor Signaling |
| MIMAT0004800 | miR-550a-5p (and other miRNAs w/seed GUGCCUG) | TargetScan Human | Moderate (predicted) | TDP2 | cAMP-mediated signaling,Cardiac β-adrenergic Signaling,G-Protein Coupled Receptor Signaling,Gustation Pathway,NF-κB Signaling,Protein Kinase A Signaling,Relaxin Signaling,tRNA Splicing |
| MIMAT0004800 | miR-550a-5p (and other miRNAs w/seed GUGCCUG) | TargetScan Human | High (predicted) | TEC | Gα12/13 Signaling,Leukocyte Extravasation Signaling,T Cell Receptor Signaling,Tec Kinase Signaling |
| MIMAT0004800 | miR-550a-5p (and other miRNAs w/seed GUGCCUG) | TargetScan Human | Moderate (predicted) | TPM1 | Calcium Signaling |
| MIMAT0004800 | miR-550a-5p (and other miRNAs w/seed GUGCCUG) | TargetScan Human | Moderate (predicted) | TPM3 | Calcium Signaling |
| MIMAT0004800 | miR-550a-5p (and other miRNAs w/seed GUGCCUG) | TargetScan Human | Moderate (predicted) | TRAF2 | 14-3-3-mediated Signaling,4-1BB Signaling in T Lymphocytes,Acute Phase Response Signaling,April Mediated Signaling,B Cell Activating Factor Signaling,CD27 Signaling in Lymphocytes,CD40 Signaling,Death Receptor Signaling,Endoplasmic Reticulum Stress Pathway,Glucocorticoid Receptor Signaling,Hepatic Cholestasis,IL-15 Signaling,IL-6 Signaling,Induction of Apoptosis by HIV1,LPS/IL-1 Mediated Inhibition of RXR Function,Lymphotoxin |

| | | | | | |
|---|---|---|---|---|---|
| | | | | | β Receptor Signaling,NF-κB Activation by Viruses,NF-κB Signaling,OX40 Signaling Pathway,p38 MAPK Signaling,PPAR Signaling,RANK Signaling in Osteoclasts,Role of Macrophages, Fibroblasts and Endothelial Cells in Rheumatoid Arthritis,Role of Osteoblasts, Osteoclasts and Chondrocytes in Rheumatoid Arthritis,Role of PKR in Interferon Induction and Antiviral Response,Role of RIG1-like Receptors in Antiviral Innate Immunity,SAPK/JNK Signaling,Small Cell Lung Cancer Signaling,TNFR1 Signaling,TNFR2 Signaling,TWEAK Signaling,Type I Diabetes Mellitus Signaling,Type II Diabetes Mellitus Signaling,Unfolded protein response |
| MIMAT0004800 | miR-550a-5p (and other miRNAs w/seed GUGCCUG) | TargetScan Human | Moderate (predicted) | TRAF4 | Lymphotoxin β Receptor Signaling,NGF Signaling,Role of Macrophages, Fibroblasts and Endothelial Cells in Rheumatoid Arthritis,Small Cell Lung Cancer Signaling,Toll-like Receptor Signaling |
| MIMAT0004800 | miR-550a-5p (and other miRNAs w/seed GUGCCUG) | TargetScan Human | High (predicted) | TRPM5 | GPCR-Mediated Nutrient Sensing in Enteroendocrine Cells,Gustation Pathway |
| MIMAT0004800 | miR-550a-5p (and other miRNAs w/seed GUGCCUG) | TargetScan Human | Moderate (predicted) | UNC5A | Axonal Guidance Signaling,Netrin Signaling |
| MIMAT0004800 | miR-550a-5p (and other miRNAs w/seed GUGCCUG) | TargetScan Human | Moderate (predicted) | UNC5B | Axonal Guidance Signaling,Netrin Signaling |
| MIMAT0003247 | miR-582-5p (miRNAs w/seed UACAGUU) | TargetScan Human | Moderate (predicted) | ABLIM2 | Axonal Guidance Signaling,Netrin Signaling |
| MIMAT0003247 | miR-582-5p (miRNAs w/seed UACAGUU) | TargetScan Human | Moderate (predicted) | ACACA | AMPK Signaling,Biotin-carboxyl Carrier Protein Assembly,LXR/RXR Activation,TR/RXR Activation |
| MIMAT0003247 | miR-582-5p (miRNAs w/seed UACAGUU) | TargetScan Human | Moderate (predicted) | ACE | Axonal Guidance Signaling,Neuroprotective Role of THOP1 in Alzheimer's Disease,Renin-Angiotensin Signaling |

| | | | | | |
|---|---|---|---|---|---|
| MIMAT0003247 | miR-582-5p (miRNAs w/seed UACAGUU) | TargetScan Human | High (predicted) | ADAMTS5 | Axonal Guidance Signaling,Role of Osteoblasts, Osteoclasts and Chondrocytes in Rheumatoid Arthritis |
| MIMAT0003247 | miR-582-5p (miRNAs w/seed UACAGUU) | TargetScan Human | Moderate (predicted) | AKAP13 | cAMP-mediated signaling,Cardiac β-adrenergic Signaling,Protein Kinase A Signaling |
| MIMAT0003247 | miR-582-5p (miRNAs w/seed UACAGUU) | TargetScan Human | Moderate (predicted) | ANAPC7 | Mitotic Roles of Polo-Like Kinase,Protein Kinase A Signaling |
| MIMAT0003247 | miR-582-5p (miRNAs w/seed UACAGUU) | TargetScan Human | Moderate (predicted) | ATP2B1 | Calcium Signaling,Calcium Transport I |
| MIMAT0003247 | miR-582-5p (miRNAs w/seed UACAGUU) | TargetScan Human | Moderate (predicted) | BMP10 | Axonal Guidance Signaling,Basal Cell Carcinoma Signaling,BMP signaling pathway,Cardiomyocyte Differentiation via BMP Receptors,Factors Promoting Cardiogenesis in Vertebrates,Human Embryonic Stem Cell Pluripotency,Molecular Mechanisms of Cancer,Role of NANOG in Mammalian Embryonic Stem Cell Pluripotency,Role of Osteoblasts, Osteoclasts and Chondrocytes in Rheumatoid Arthritis |
| MIMAT0003247 | miR-582-5p (miRNAs w/seed UACAGUU) | TargetScan Human | Moderate (predicted) | CASP3 | Amyotrophic Lateral Sclerosis Signaling,Apoptosis Signaling,CD27 Signaling in Lymphocytes,Colorectal Cancer Metastasis Signaling,Cytotoxic T Lymphocyte-mediated Apoptosis of Target Cells,Death Receptor Signaling,Docosahexaenoic Acid (DHA) Signaling,Endoplasmic Reticulum Stress Pathway,Endothelin-1 Signaling,eNOS Signaling,Granzyme B Signaling,Huntington's Disease Signaling,ILK Signaling,Induction of Apoptosis by HIV1,Inhibition of Angiogenesis by TSP1,Lymphotoxin β Receptor Signaling,Mitochondrial Dysfunction,Molecular Mechanisms of Cancer,Myc Mediated Apoptosis Signaling,Nur77 Signaling in T Lymphocytes,PAK Signaling,Parkinson's Signaling,PTEN Signaling,Retinoic acid Mediated Apoptosis Signaling,Role of MAPK Signaling in the Pathogenesis of Influenza,Role of PKR in Interferon Induction and Antiviral Response,Role of Tissue Factor in Cancer,Sphingosine-1-phosphate Signaling,TNFR1 Signaling,Tumoricidal Function of Hepatic Natural Killer Cells,TWEAK Signaling,Type I Diabetes Mellitus Signaling,UVA-Induced MAPK Signaling |
| MIMAT0003247 | miR-582-5p (miRNAs w/seed | TargetScan Human | Moderate (predicted) | CCL11 | Agranulocyte Adhesion and Diapedesis,Atherosclerosis Signaling,CCR3 Signaling in Eosinophils,Chemokine Signaling,Glucocorticoid Receptor |

| | | | | | |
|---|---|---|---|---|---|
| | UACAGUU) | | | | Signaling,Granulocyte Adhesion and Diapedesis,IL-17 Signaling,IL-17A Signaling in Airway Cells |
| MIMAT0003247 | miR-582-5p (miRNAs w/seed UACAGUU) | TargetScan Human | Moderate (predicted) | CDYL | Transcriptional Regulatory Network in Embryonic Stem Cells |
| MIMAT0003247 | miR-582-5p (miRNAs w/seed UACAGUU) | TargetScan Human | Moderate (predicted) | CREB1 | AMPK Signaling,ATM Signaling,B Cell Receptor Signaling,BMP signaling pathway,Calcium Signaling,cAMP-mediated signaling,Cardiac Hypertrophy Signaling,Circadian Rhythm Signaling,Corticotropin Releasing Hormone Signaling,CREB Signaling in Neurons,Dendritic Cell Maturation,Dopamine-DARPP32 Feedback in cAMP Signaling,Ephrin Receptor Signaling,ERK/MAPK Signaling,ERK5 Signaling,Estrogen-Dependent Breast Cancer Signaling,FGF Signaling,FLT3 Signaling in Hematopoietic Progenitor Cells,G-Protein Coupled Receptor Signaling,GDNF Family Ligand-Receptor Interactions,Glucocorticoid Receptor Signaling,GNRH Signaling,Gαs Signaling,Huntington's Disease Signaling,Hypoxia Signaling in the Cardiovascular System,ILK Signaling,LPS-stimulated MAPK Signaling,Melanocyte Development and Pigmentation Signaling,Neuropathic Pain Signaling In Dorsal Horn Neurons,Neuroprotective Role of THOP1 in Alzheimer's Disease,Neurotrophin/TRK Signaling,NGF Signaling,P2Y Purigenic Receptor Signaling Pathway,p38 MAPK Signaling,Phospholipase C Signaling,PI3K Signaling in B Lymphocytes,Prostate Cancer Signaling,Protein Kinase A Signaling,Relaxin Signaling,Role of IL-17F in Allergic Inflammatory Airway Diseases,Role of Macrophages, Fibroblasts and Endothelial Cells in Rheumatoid Arthritis,Role of Pattern Recognition Receptors in Recognition of Bacteria and Viruses,Synaptic Long Term Potentiation,Thrombin Signaling,Wnt/Ca+ pathway |
| MIMAT0003247 | miR-582-5p (miRNAs w/seed UACAGUU) | TargetScan Human | Moderate (predicted) | CREM | cAMP-mediated signaling,Cholecystokinin/Gastrin-mediated Signaling,Dopamine-DARPP32 Feedback in cAMP Signaling,Protein Kinase A Signaling,Systemic Lupus Erythematosus Signaling |
| MIMAT0003247 | miR-582-5p (miRNAs w/seed UACAGUU) | TargetScan Human | Moderate (predicted) | EIF2AK3 | EIF2 Signaling,Endoplasmic Reticulum Stress Pathway,NRF2-mediated Oxidative Stress Response,Unfolded protein response,Xenobiotic Metabolism Signaling |
| MIMAT0003247 | miR-582-5p (miRNAs w/seed UACAGUU) | TargetScan Human | Moderate (predicted) | ELF1 | ERK/MAPK Signaling,HGF Signaling,Telomerase Signaling |
| MIMAT0003247 | miR-582-5p | TargetScan Human | Moderate (predicted) | F2R | Actin Cytoskeleton Signaling,Clathrin-mediated Endocytosis |

| | | | | | |
|---|---|---|---|---|---|
| | (miRNAs w/seed UACAGUU) | | | | Signaling,Coagulation System,Glioma Invasiveness Signaling,Gα12/13 Signaling,p70S6K Signaling,Thrombin Signaling |
| MIMAT0003247 | miR-582-5p (miRNAs w/seed UACAGUU) | TargetScan Human | Moderate (predicted) | FAM208A | Role of Oct4 in Mammalian Embryonic Stem Cell Pluripotency |
| MIMAT0003247 | miR-582-5p (miRNAs w/seed UACAGUU) | TargetScan Human | Moderate (predicted) | FOXG1 | AMPK Signaling,PTEN Signaling |
| MIMAT0003247 | miR-582-5p (miRNAs w/seed UACAGUU) | TargetScan Human | Moderate (predicted) | FZD2 | Adipogenesis pathway,Axonal Guidance Signaling,Basal Cell Carcinoma Signaling,Colorectal Cancer Metastasis Signaling,Factors Promoting Cardiogenesis in Vertebrates,Glioblastoma Multiforme Signaling,Human Embryonic Stem Cell Pluripotency,Molecular Mechanisms of Cancer,Mouse Embryonic Stem Cell Pluripotency,Ovarian Cancer Signaling,PCP pathway,Regulation of the Epithelial-Mesenchymal Transition Pathway,Role of Macrophages, Fibroblasts and Endothelial Cells in Rheumatoid Arthritis,Role of NANOG in Mammalian Embryonic Stem Cell Pluripotency,Role of Osteoblasts, Osteoclasts and Chondrocytes in Rheumatoid Arthritis,Role of Wnt/GSK-3β Signaling in the Pathogenesis of Influenza,Wnt/Ca+ pathway,Wnt/β-catenin Signaling |
| MIMAT0003247 | miR-582-5p (miRNAs w/seed UACAGUU) | TargetScan Human | Moderate (predicted) | FZD3 | Adipogenesis pathway,Axonal Guidance Signaling,Basal Cell Carcinoma Signaling,Colorectal Cancer Metastasis Signaling,Factors Promoting Cardiogenesis in Vertebrates,Glioblastoma Multiforme Signaling,Human Embryonic Stem Cell Pluripotency,Molecular Mechanisms of Cancer,Mouse Embryonic Stem Cell Pluripotency,Ovarian Cancer Signaling,PCP pathway,Regulation of the Epithelial-Mesenchymal Transition Pathway,Role of Macrophages, Fibroblasts and Endothelial Cells in Rheumatoid Arthritis,Role of NANOG in Mammalian Embryonic Stem Cell Pluripotency,Role of Osteoblasts, Osteoclasts and Chondrocytes in Rheumatoid Arthritis,Role of Wnt/GSK-3β Signaling in the Pathogenesis of Influenza,Wnt/Ca+ pathway,Wnt/β-catenin Signaling |
| MIMAT0003247 | miR-582-5p (miRNAs w/seed UACAGUU) | TargetScan Human | Moderate (predicted) | GABPB1 | Agrin Interactions at Neuromuscular Junction |
| MIMAT0003247 | miR-582-5p (miRNAs w/seed | TargetScan Human | Moderate (predicted) | GABRR1 | GABA Receptor Signaling |

| | | | | | |
|---|---|---|---|---|---|
| | UACAGUU) | | | | |
| MIMAT0003247 | miR-582-5p (miRNAs w/seed UACAGUU) | TargetScan Human | Moderate (predicted) | GNAO1 | Androgen Signaling,Axonal Guidance Signaling,cAMP-mediated signaling,Cardiac Hypertrophy Signaling,Corticotropin Releasing Hormone Signaling,CREB Signaling in Neurons,CXCR4 Signaling,Endothelin-1 Signaling,Ephrin B Signaling,Ephrin Receptor Signaling,G Beta Gamma Signaling,G-Protein Coupled Receptor Signaling,IL-1 Signaling,Melatonin Signaling,Molecular Mechanisms of Cancer,Relaxin Signaling,RhoGDI Signaling,Role of Macrophages, Fibroblasts and Endothelial Cells in Rheumatoid Arthritis,Role of NFAT in Regulation of the Immune Response,Signaling by Rho Family GTPases,Synaptic Long Term Depression,Tec Kinase Signaling,Thrombin Signaling,Wnt/β-catenin Signaling |
| MIMAT0003247 | miR-582-5p (miRNAs w/seed UACAGUU) | TargetScan Human | Moderate (predicted) | GNAT1 | Androgen Signaling,Axonal Guidance Signaling,Cardiac Hypertrophy Signaling,CREB Signaling in Neurons,CXCR4 Signaling,Endothelin-1 Signaling,Ephrin B Signaling,Ephrin Receptor Signaling,G Beta Gamma Signaling,IL-1 Signaling,Molecular Mechanisms of Cancer,Phototransduction Pathway,Relaxin Signaling,RhoGDI Signaling,Role of NFAT in Regulation of the Immune Response,Signaling by Rho Family GTPases,Synaptic Long Term Depression,Tec Kinase Signaling,Thrombin Signaling |
| MIMAT0003247 | miR-582-5p (miRNAs w/seed UACAGUU) | TargetScan Human | Moderate (predicted) | GRIP1 | Glutamate Receptor Signaling,RhoGDI Signaling,Xenobiotic Metabolism Signaling |
| MIMAT0003247 | miR-582-5p (miRNAs w/seed UACAGUU) | TargetScan Human | Moderate (predicted) | GRM5 | CREB Signaling in Neurons,G-Protein Coupled Receptor Signaling,Glutamate Receptor Signaling,Gαq Signaling,Huntington's Disease Signaling,Neuropathic Pain Signaling In Dorsal Horn Neurons,Synaptic Long Term Depression,Synaptic Long Term Potentiation |
| MIMAT0003247 | miR-582-5p (miRNAs w/seed UACAGUU) | TargetScan Human | Moderate (predicted) | HIF1A | Adipogenesis pathway,HIF1α Signaling,Hypoxia Signaling in the Cardiovascular System,ILK Signaling,Molecular Mechanisms of Cancer,mTOR Signaling,p53 Signaling,Regulation of the Epithelial-Mesenchymal Transition Pathway,Renal Cell Carcinoma Signaling,TR/RXR Activation,VEGF Signaling |
| MIMAT0003247 | miR-582-5p (miRNAs w/seed UACAGUU) | TargetScan Human | Moderate (predicted) | HMGB1 | Glucocorticoid Receptor Signaling,HMGB1 Signaling |
| MIMAT0003247 | miR-582-5p (miRNAs w/seed | TargetScan Human | Moderate (predicted) | HMGCR | AMPK Signaling,LXR/RXR Activation,Mevalonate Pathway I,Superpathway of Cholesterol Biosynthesis,Superpathway of Geranylgeranyldiphosphate |

| | | | | | |
|---|---|---|---|---|---|
| | UACAGUU) | | | | Biosynthesis I (via Mevalonate) |
| MIMAT0003247 | miR-582-5p (miRNAs w/seed UACAGUU) | TargetScan Human | Moderate (predicted) | IL33 | Acute Phase Response Signaling,Agranulocyte Adhesion and Diapedesis,Altered T Cell and B Cell Signaling in Rheumatoid Arthritis,Atherosclerosis Signaling,Cholecystokinin/Gastrin-mediated Signaling,Communication between Innate and Adaptive Immune Cells,Dendritic Cell Maturation,FXR/RXR Activation,Graft-versus-Host Disease Signaling,Granulocyte Adhesion and Diapedesis,Hepatic Cholestasis,IL-10 Signaling,IL-6 Signaling,LPS/IL-1 Mediated Inhibition of RXR Function,LXR/RXR Activation,NF-κB Signaling,p38 MAPK Signaling,PPAR Signaling,Role of Cytokines in Mediating Communication between Immune Cells,Role of Hypercytokinemia/hyperchemokinemia in the Pathogenesis of Influenza,Role of Macrophages, Fibroblasts and Endothelial Cells in Rheumatoid Arthritis,Role of Osteoblasts, Osteoclasts and Chondrocytes in Rheumatoid Arthritis,Systemic Lupus Erythematosus Signaling,Toll-like Receptor Signaling |
| MIMAT0003247 | miR-582-5p (miRNAs w/seed UACAGUU) | TargetScan Human | Moderate (predicted) | INPP5B | 1D-myo-inositol Hexakisphosphate Biosynthesis II (Mammalian),3-phosphoinositide Degradation,B Cell Receptor Signaling,D-myo-inositol (1,3,4)-trisphosphate Biosynthesis,D-myo-inositol (1,4,5)-trisphosphate Degradation,Fc Epsilon RI Signaling,IL-4 Signaling,Insulin Receptor Signaling,Natural Killer Cell Signaling,PDGF Signaling,PI3K/AKT Signaling,PTEN Signaling,Superpathway of D-myo-inositol (1,4,5)-trisphosphate Metabolism,Superpathway of Inositol Phosphate Compounds |
| MIMAT0003247 | miR-582-5p (miRNAs w/seed UACAGUU) | TargetScan Human | Moderate (predicted) | ITGA2 | Actin Cytoskeleton Signaling,Actin Nucleation by ARP-WASP Complex,Agranulocyte Adhesion and Diapedesis,Agrin Interactions at Neuromuscular Junction,Axonal Guidance Signaling,Caveolar-mediated Endocytosis Signaling,Cdc42 Signaling,CDK5 Signaling,Ephrin Receptor Signaling,ERK/MAPK Signaling,FAK Signaling,Germ Cell-Sertoli Cell Junction Signaling,Granulocyte Adhesion and Diapedesis,HGF Signaling,Integrin Signaling,Leukocyte Extravasation Signaling,Molecular Mechanisms of Cancer,Neuregulin Signaling,NF-κB Activation by Viruses,PAK Signaling,Paxillin Signaling,phagosome formation,Phospholipase C Signaling,PI3K/AKT Signaling,PTEN Signaling,Rac Signaling,Reelin Signaling in Neurons,Regulation of Actin-based Motility by Rho,Regulation of Cellular Mechanics by Calpain Protease,Regulation of eIF4 and p70S6K Signaling,RhoGDI Signaling,Role of Osteoblasts, Osteoclasts and Chondrocytes in Rheumatoid Arthritis,Sertoli Cell-Sertoli Cell Junction Signaling,Signaling by Rho Family GTPases,Tec Kinase Signaling,Virus Entry via Endocytic Pathways |

| | | | | | |
|---|---|---|---|---|---|
| MIMAT0003247 | miR-582-5p (miRNAs w/seed UACAGUU) | TargetScan Human | Moderate (predicted) | ITSN1 | Axonal Guidance Signaling,Caveolar-mediated Endocytosis Signaling,Ephrin B Signaling,Ephrin Receptor Signaling,Virus Entry via Endocytic Pathways |
| MIMAT0003247 | miR-582-5p (miRNAs w/seed UACAGUU) | TargetScan Human | Moderate (predicted) | KALRN | Axonal Guidance Signaling,Ephrin B Signaling,Ephrin Receptor Signaling |
| MIMAT0003247 | miR-582-5p (miRNAs w/seed UACAGUU) | TargetScan Human | Moderate (predicted) | KCNQ3 | Cellular Effects of Sildenafil (Viagra),GABA Receptor Signaling,Neuropathic Pain Signaling In Dorsal Horn Neurons |
| MIMAT0003247 | miR-582-5p (miRNAs w/seed UACAGUU) | TargetScan Human | Moderate (predicted) | KLC1 | Axonal Guidance Signaling |
| MIMAT0003247 | miR-582-5p (miRNAs w/seed UACAGUU) | TargetScan Human | Moderate (predicted) | LPAR1 | cAMP-mediated signaling,eNOS Signaling,G-Protein Coupled Receptor Signaling,Gap Junction Signaling,Gα12/13 Signaling,Gαi Signaling,RhoA Signaling |
| MIMAT0003247 | miR-582-5p (miRNAs w/seed UACAGUU) | TargetScan Human | Moderate (predicted) | MAP3K1 | Acute Phase Response Signaling,April Mediated Signaling,B Cell Activating Factor Signaling,B Cell Receptor Signaling,Cardiac Hypertrophy Signaling,CD27 Signaling in Lymphocytes,CD28 Signaling in T Helper Cells,Ceramide Signaling,EGF Signaling,FGF Signaling,Germ Cell-Sertoli Cell Junction Signaling,Glucocorticoid Receptor Signaling,GNRH Signaling,Gα12/13 Signaling,HGF Signaling,IL-1 Signaling,LPS/IL-1 Mediated Inhibition of RXR Function,NF-κB Activation by Viruses,NF-κB Signaling,NGF Signaling,NRF2-mediated Oxidative Stress Response,PDGF Signaling,PKCθ Signaling in T Lymphocytes,Production of Nitric Oxide and Reactive Oxygen Species in Macrophages,Protein Kinase A Signaling,Rac Signaling,RANK Signaling in Osteoclasts,RAR Activation,Regulation of IL-2 Expression in Activated and Anergic T Lymphocytes,Renin-Angiotensin Signaling,Role of NFAT in Cardiac Hypertrophy,SAPK/JNK Signaling,Sertoli Cell-Sertoli Cell Junction Signaling,T Cell Receptor Signaling,TNFR1 Signaling,TNFR2 Signaling,Toll-like Receptor Signaling,Type II Diabetes Mellitus Signaling,Xenobiotic Metabolism Signaling |
| MIMAT0003247 | miR-582-5p (miRNAs w/seed UACAGUU) | TargetScan Human | Moderate (predicted) | MAP3K7 | Acute Phase Response Signaling,AMPK Signaling,B Cell Receptor Signaling,BMP signaling pathway,Cardiac Hypertrophy Signaling,Cardiomyocyte Differentiation via BMP Receptors,CD27 Signaling in Lymphocytes,CD40 Signaling,Factors Promoting Cardiogenesis in Vertebrates,Germ Cell-Sertoli Cell Junction Signaling,Glucocorticoid Receptor |

| | | | | | |
|---|---|---|---|---|---|
| | | | | | Signaling,GNRH Signaling,Hepatic Cholestasis,HGF Signaling,IL-1 Signaling,IL-10 Signaling,IL-17 Signaling,IL-17A Signaling in Airway Cells,IL-17A Signaling in Fibroblasts,IL-6 Signaling,LPS-stimulated MAPK Signaling,LPS/IL-1 Mediated Inhibition of RXR Function,Molecular Mechanisms of Cancer,Mouse Embryonic Stem Cell Pluripotency,NF-κB Signaling,NGF Signaling,NRF2-mediated Oxidative Stress Response,p38 MAPK Signaling,PKCθ Signaling in T Lymphocytes,PPAR Signaling,PPARα/RXRα Activation,Production of Nitric Oxide and Reactive Oxygen Species in Macrophages,RANK Signaling in Osteoclasts,Role of Macrophages, Fibroblasts and Endothelial Cells in Rheumatoid Arthritis,Role of NFAT in Cardiac Hypertrophy,Role of Osteoblasts, Osteoclasts and Chondrocytes in Rheumatoid Arthritis,Role of PKR in Interferon Induction and Antiviral Response,SAPK/JNK Signaling,Sertoli Cell-Sertoli Cell Junction Signaling,TGF-β Signaling,Toll-like Receptor Signaling,Type I Diabetes Mellitus Signaling,Type II Diabetes Mellitus Signaling,Wnt/β-catenin Signaling,Xenobiotic Metabolism Signaling |
| MIMAT0003247 | miR-582-5p (miRNAs w/seed UACAGUU) | TargetScan Human | Moderate (predicted) | MAPK14 | 4-1BB Signaling in T Lymphocytes,Acute Phase Response Signaling,AMPK Signaling,Amyloid Processing,Antioxidant Action of Vitamin C,April Mediated Signaling,ATM Signaling,B Cell Activating Factor Signaling,B Cell Receptor Signaling,BMP signaling pathway,Cardiac Hypertrophy Signaling,CCR3 Signaling in Eosinophils,CCR5 Signaling in Macrophages,CD40 Signaling,Cdc42 Signaling,CDK5 Signaling,Chemokine Signaling,Cholecystokinin/Gastrin-mediated Signaling,Corticotropin Releasing Hormone Signaling,Dendritic Cell Maturation,EGF Signaling,Endothelin-1 Signaling,ErbB Signaling,Factors Promoting Cardiogenesis in Vertebrates,Fc Epsilon RI Signaling,FGF Signaling,FLT3 Signaling in Hematopoietic Progenitor Cells,Germ Cell-Sertoli Cell Junction Signaling,Glucocorticoid Receptor Signaling,GNRH Signaling,HIF1α Signaling,HMGB1 Signaling,IL-1 Signaling,IL-10 Signaling,IL-12 Signaling and Production in Macrophages,IL-15 Signaling,IL-17 Signaling,IL-17A Signaling in Airway Cells,IL-17A Signaling in Fibroblasts,IL-17A Signaling in Gastric Cells,IL-22 Signaling,IL-6 Signaling,Inhibition of Angiogenesis by TSP1,iNOS Signaling,Leukocyte Extravasation Signaling,LPS-stimulated MAPK Signaling,Molecular Mechanisms of Cancer,Mouse Embryonic Stem Cell Pluripotency,NRF2-mediated Oxidative Stress Response,p38 MAPK Signaling,p53 Signaling,Parkinson's Signaling,Paxillin Signaling,PEDF Signaling,PPARα/RXRα Activation,Production of Nitric Oxide and Reactive Oxygen Species in Macrophages,RANK Signaling in Osteoclasts,RAR Activation,Regulation of eIF4 and p70S6K Signaling,Renin-Angiotensin Signaling,Role of IL-17A in Arthritis,Role of JAK family kinases in IL-6-type Cytokine Signaling,Role of Macrophages, Fibroblasts and Endothelial Cells in Rheumatoid Arthritis,Role of MAPK Signaling in the Pathogenesis of |

| | | | | | |
|---|---|---|---|---|---|
| | | | | | Influenza,Role of NFAT in Cardiac Hypertrophy,Role of Osteoblasts, Osteoclasts and Chondrocytes in Rheumatoid Arthritis,Role of PKR in Interferon Induction and Antiviral Response,Role of Tissue Factor in Cancer,Sertoli Cell-Sertoli Cell Junction Signaling,STAT3 Pathway,TGF-β Signaling,Thrombin Signaling,Toll-like Receptor Signaling,Type I Diabetes Mellitus Signaling,UVA-Induced MAPK Signaling,UVB-Induced MAPK Signaling,UVC-Induced MAPK Signaling,Xenobiotic Metabolism Signaling |
| MIMAT0003247 | miR-582-5p (miRNAs w/seed UACAGUU) | TargetScan Human | Moderate (predicted) | NFE2L2 | Aryl Hydrocarbon Receptor Signaling,NRF2-mediated Oxidative Stress Response,Unfolded protein response,Xenobiotic Metabolism Signaling |
| MIMAT0003247 | miR-582-5p (miRNAs w/seed UACAGUU) | TargetScan Human | Moderate (predicted) | NR3C1 | Acute Phase Response Signaling,Estrogen Receptor Signaling,Glucocorticoid Receptor Signaling,IL-4 Signaling,MIF-mediated Glucocorticoid Regulation,Prolactin Signaling,PXR/RXR Activation |
| MIMAT0003247 | miR-582-5p (miRNAs w/seed UACAGUU) | TargetScan Human | Moderate (predicted) | NRP1 | Axonal Guidance Signaling,Semaphorin Signaling in Neurons,VEGF Family Ligand-Receptor Interactions |
| MIMAT0003247 | miR-582-5p (miRNAs w/seed UACAGUU) | TargetScan Human | Moderate (predicted) | NSF | GABA Receptor Signaling,Huntington's Disease Signaling,phagosome maturation,Tight Junction Signaling |
| MIMAT0003247 | miR-582-5p (miRNAs w/seed UACAGUU) | TargetScan Human | Moderate (predicted) | PDE7A | cAMP-mediated signaling,Cardiac β-adrenergic Signaling,G-Protein Coupled Receptor Signaling,Gustation Pathway,Protein Kinase A Signaling,Relaxin Signaling,tRNA Splicing |
| MIMAT0003247 | miR-582-5p (miRNAs w/seed UACAGUU) | TargetScan Human | Moderate (predicted) | PIK3R4 | 14-3-3-mediated Signaling,3-phosphoinositide Biosynthesis,Actin Cytoskeleton Signaling,Acute Myeloid Leukemia Signaling,Aldosterone Signaling in Epithelial Cells,AMPK Signaling,Amyotrophic Lateral Sclerosis Signaling,Angiopoietin Signaling,Antiproliferative Role of Somatostatin Receptor 2,autophagy,Axonal Guidance Signaling,B Cell Receptor Signaling,Breast Cancer Regulation by Stathmin1,Cardiac Hypertrophy Signaling,CCR3 Signaling in Eosinophils,CD28 Signaling in T Helper Cells,CD40 Signaling,Ceramide Signaling,Chronic Myeloid Leukemia Signaling,Clathrin-mediated Endocytosis Signaling,CNTF Signaling,Colorectal Cancer Metastasis Signaling,CREB Signaling in Neurons,CTLA4 Signaling in Cytotoxic T Lymphocytes,CXCR4 Signaling,Dendritic Cell Maturation,Docosahexaenoic Acid (DHA) Signaling,EGF Signaling,EIF2 Signaling,Endometrial Cancer Signaling,Endothelin-1 Signaling,eNOS Signaling,Ephrin A Signaling,ErbB |

| | | | | | |
|---|---|---|---|---|---|
| | | | | | Signaling,ErbB2-ErbB3 Signaling,ErbB4 Signaling,ERK/MAPK Signaling,Erythropoietin Signaling,Estrogen-Dependent Breast Cancer Signaling,FAK Signaling,Fc Epsilon RI Signaling,FcγRIIB Signaling in B Lymphocytes,FGF Signaling,FLT3 Signaling in Hematopoietic Progenitor Cells,fMLP Signaling in Neutrophils,G-Protein Coupled Receptor Signaling,Gap Junction Signaling,GDNF Family Ligand-Receptor Interactions,Germ Cell-Sertoli Cell Junction Signaling,Glioblastoma Multiforme Signaling,Glioma Invasiveness Signaling,Glioma Signaling,Glucocorticoid Receptor Signaling,GM-CSF Signaling,Growth Hormone Signaling,Gα12/13 Signaling,Gαq Signaling,HER-2 Signaling in Breast Cancer,Hereditary Breast Cancer Signaling,HGF Signaling,HIF1α Signaling,HMGB1 Signaling,Human Embryonic Stem Cell Pluripotency,Huntington's Disease Signaling,iCOS-iCOSL Signaling in T Helper Cells,IGF-1 Signaling,IL-12 Signaling and Production in Macrophages,IL-15 Signaling,IL-17 Signaling,IL-17A Signaling in Airway Cells,IL-2 Signaling,IL-3 Signaling,IL-4 Signaling,IL-6 Signaling,IL-8 Signaling,IL-9 Signaling,ILK Signaling,Insulin Receptor Signaling,Integrin Signaling,JAK/Stat Signaling,Leptin Signaling in Obesity,Leukocyte Extravasation Signaling,LPS-stimulated MAPK Signaling,Lymphotoxin β Receptor Signaling,Macropinocytosis Signaling,Melanocyte Development and Pigmentation Signaling,Melanoma Signaling,Molecular Mechanisms of Cancer,Mouse Embryonic Stem Cell Pluripotency,MSP-RON Signaling Pathway,mTOR Signaling,Myc Mediated Apoptosis Signaling,Natural Killer Cell Signaling,Neuropathic Pain Signaling In Dorsal Horn Neurons,Neurotrophin/TRK Signaling,NF-κB Activation by Viruses,NF-κB Signaling,NGF Signaling,Nitric Oxide Signaling in the Cardiovascular System,Non-Small Cell Lung Cancer Signaling,NRF2-mediated Oxidative Stress Response,Ovarian Cancer Signaling,P2Y Purigenic Receptor Signaling Pathway,p53 Signaling,p70S6K Signaling,PAK Signaling,Pancreatic Adenocarcinoma Signaling,Paxillin Signaling,PDGF Signaling,PEDF Signaling,phagosome formation,PKCθ Signaling in T Lymphocytes,Production of Nitric Oxide and Reactive Oxygen Species in Macrophages,Prolactin Signaling,Prostate Cancer Signaling,Rac Signaling,RANK Signaling in Osteoclasts,Reelin Signaling in Neurons,Regulation of eIF4 and p70S6K Signaling,Regulation of the Epithelial-Mesenchymal Transition Pathway,Relaxin Signaling,Renal Cell Carcinoma Signaling,Renin-Angiotensin Signaling,Role of IL-17A in Arthritis,Role of JAK1 and JAK3 in γc Cytokine Signaling,Role of Macrophages, Fibroblasts and Endothelial Cells in Rheumatoid Arthritis,Role of NANOG in Mammalian Embryonic Stem Cell Pluripotency,Role of NFAT in Cardiac Hypertrophy,Role of NFAT in Regulation of the Immune Response,Role of Osteoblasts, Osteoclasts and Chondrocytes in Rheumatoid Arthritis,Role of p14/p19ARF in Tumor Suppression,Role of |

| | | | | | |
|---|---|---|---|---|---|
| | | | | | Pattern Recognition Receptors in Recognition of Bacteria and Viruses,Role of PI3K/AKT Signaling in the Pathogenesis of Influenza,Role of Tissue Factor in Cancer,SAPK/JNK Signaling,Signaling by Rho Family GTPases,Small Cell Lung Cancer Signaling,Sphingosine-1-phosphate Signaling,Superpathway of Inositol Phosphate Compounds,Systemic Lupus Erythematosus Signaling,T Cell Receptor Signaling,Tec Kinase Signaling,Telomerase Signaling,Thrombin Signaling,Thrombopoietin Signaling,TR/RXR Activation,Type II Diabetes Mellitus Signaling,UVA-Induced MAPK Signaling,UVB-Induced MAPK Signaling,VEGF Family Ligand-Receptor Interactions,VEGF Signaling,Virus Entry via Endocytic Pathways,Xenobiotic Metabolism Signaling |
| MIMAT0003247 | miR-582-5p (miRNAs w/seed UACAGUU) | TargetScan Human | Moderate (predicted) | PLA2G12A | Antioxidant Action of Vitamin C,Atherosclerosis Signaling,CCR3 Signaling in Eosinophils,Eicosanoid Signaling,Endothelin-1 Signaling,ERK/MAPK Signaling,Fc Epsilon RI Signaling,MIF Regulation of Innate Immunity,MIF-mediated Glucocorticoid Regulation,p38 MAPK Signaling,Phospholipase C Signaling,Phospholipases,Role of MAPK Signaling in the Pathogenesis of Influenza,Sperm Motility,Synaptic Long Term Depression,VEGF Family Ligand-Receptor Interactions |
| MIMAT0003247 | miR-582-5p (miRNAs w/seed UACAGUU) | TargetScan Human | Moderate (predicted) | PLXNA2 | Axonal Guidance Signaling |
| MIMAT0003247 | miR-582-5p (miRNAs w/seed UACAGUU) | TargetScan Human | Moderate (predicted) | POU2F1 | Glucocorticoid Receptor Signaling,PKCθ Signaling in T Lymphocytes,Role of BRCA1 in DNA Damage Response |
| MIMAT0003247 | miR-582-5p (miRNAs w/seed UACAGUU) | TargetScan Human | Moderate (predicted) | PPP2R5A | 3-phosphoinositide Biosynthesis,3-phosphoinositide Degradation,AMPK Signaling,Breast Cancer Regulation by Stathmin1,Cardiac β-adrenergic Signaling,CDK5 Signaling,Cell Cycle Regulation by BTG Family Proteins,Ceramide Signaling,CTLA4 Signaling in Cytotoxic T Lymphocytes,Cyclins and Cell Cycle Regulation,D-myo-inositol (1,4,5,6)-Tetrakisphosphate Biosynthesis,D-myo-inositol (3,4,5,6)-tetrakisphosphate Biosynthesis,D-myo-inositol-5-phosphate Metabolism,Dopamine Receptor Signaling,Dopamine-DARPP32 Feedback in cAMP Signaling,ERK/MAPK Signaling,HIPPO signaling,ILK Signaling,Mitotic Roles of Polo-Like Kinase,mTOR Signaling,p70S6K Signaling,PI3K/AKT Signaling,Production of Nitric Oxide and Reactive Oxygen Species in Macrophages,Regulation of eIF4 and p70S6K Signaling,Role of CHK Proteins in Cell Cycle Checkpoint Control,Superpathway of Inositol Phosphate Compounds,Synaptic Long Term Depression,Telomerase Signaling,Tight Junction Signaling,Wnt/β-catenin |

| | | | | | |
|---|---|---|---|---|---|
| | | | | | Signaling,Xenobiotic Metabolism Signaling |
| MIMAT0003247 | miR-582-5p (miRNAs w/seed UACAGUU) | TargetScan Human | Moderate (predicted) | PPP3R1 | Axonal Guidance Signaling,B Cell Receptor Signaling,Calcium Signaling,Calcium-induced T Lymphocyte Apoptosis,cAMP-mediated signaling,Cardiac Hypertrophy Signaling,CD28 Signaling in T Helper Cells,Clathrin-mediated Endocytosis Signaling,Dopamine-DARPP32 Feedback in cAMP Signaling,fMLP Signaling in Neutrophils,Gap Junction Signaling,Glucocorticoid Receptor Signaling,GM-CSF Signaling,Gαq Signaling,iCOS-iCOSL Signaling in T Helper Cells,IL-3 Signaling,Netrin Signaling,nNOS Signaling in Neurons,Nur77 Signaling in T Lymphocytes,Phospholipase C Signaling,PI3K Signaling in B Lymphocytes,PKCθ Signaling in T Lymphocytes,Protein Kinase A Signaling,RANK Signaling in Osteoclasts,Regulation of IL-2 Expression in Activated and Anergic T Lymphocytes,Role of Macrophages, Fibroblasts and Endothelial Cells in Rheumatoid Arthritis,Role of NFAT in Cardiac Hypertrophy,Role of NFAT in Regulation of the Immune Response,Role of Osteoblasts, Osteoclasts and Chondrocytes in Rheumatoid Arthritis,Synaptic Long Term Potentiation,T Cell Receptor Signaling |
| MIMAT0003247 | miR-582-5p (miRNAs w/seed UACAGUU) | TargetScan Human | Moderate (predicted) | PRKAA1 | AMPK Signaling,eNOS Signaling,Glucocorticoid Receptor Signaling,mTOR Signaling,Nitric Oxide Signaling in the Cardiovascular System,PPARα/RXRα Activation,Pyridoxal 5'-phosphate Salvage Pathway,Salvage Pathways of Pyrimidine Ribonucleotides,Type II Diabetes Mellitus Signaling |
| MIMAT0003247 | miR-582-5p (miRNAs w/seed UACAGUU) | TargetScan Human | Moderate (predicted) | PRKACB | AMPK Signaling,Amyloid Processing,Androgen Signaling,Axonal Guidance Signaling,BMP signaling pathway,Breast Cancer Regulation by Stathmin1,Calcium Signaling,cAMP-mediated signaling,Cardiac Hypertrophy Signaling,Cardiac β-adrenergic Signaling,CDK5 Signaling,Cellular Effects of Sildenafil (Viagra),Colorectal Cancer Metastasis Signaling,Corticotropin Releasing Hormone Signaling,CREB Signaling in Neurons,Dopamine Receptor Signaling,Dopamine-DARPP32 Feedback in cAMP Signaling,eNOS Signaling,ERK/MAPK Signaling,G Beta Gamma Signaling,G-Protein Coupled Receptor Signaling,Gap Junction Signaling,Glucocorticoid Receptor Signaling,GNRH Signaling,GPCR-Mediated Integration of Enteroendocrine Signaling Exemplified by an L Cell,GPCR-Mediated Nutrient Sensing in Enteroendocrine Cells,Gustation Pathway,Gαi Signaling,Gαs Signaling,Hepatic Cholestasis,IGF-1 Signaling,IL-1 Signaling,Insulin Receptor Signaling,Leptin Signaling in Obesity,Melanocyte Development and Pigmentation Signaling,Melatonin Signaling,Molecular Mechanisms of Cancer,Netrin Signaling,Neuropathic Pain Signaling In Dorsal Horn Neurons,Neuroprotective Role of THOP1 in Alzheimer's Disease,NF-κB Signaling,Nitric Oxide Signaling in |

| | | | | | |
|---|---|---|---|---|---|
| | | | | | the Cardiovascular System,Ovarian Cancer Signaling,P2Y Purigenic Receptor Signaling Pathway,Phototransduction Pathway,PPARα/RXRα Activation,Protein Kinase A Signaling,PXR/RXR Activation,RAR Activation,Relaxin Signaling,Renin-Angiotensin Signaling,Role of NFAT in Cardiac Hypertrophy,Sertoli Cell-Sertoli Cell Junction Signaling,Sonic Hedgehog Signaling,Sperm Motility,Synaptic Long Term Potentiation,Tight Junction Signaling,α-Adrenergic Signaling |
| MIMAT0003247 | miR-582-5p (miRNAs w/seed UACAGUU) | TargetScan Human | Moderate (predicted) | PRKCE | 14-3-3-mediated Signaling,Aldosterone Signaling in Epithelial Cells,Amyloid Processing,Androgen Signaling,Apoptosis Signaling,Axonal Guidance Signaling,Breast Cancer Regulation by Stathmin1,Calcium-induced T Lymphocyte Apoptosis,CCR3 Signaling in Eosinophils,CCR5 Signaling in Macrophages,Cholecystokinin/Gastrin-mediated Signaling,Corticotropin Releasing Hormone Signaling,CREB Signaling in Neurons,CXCR4 Signaling,Dopamine-DARPP32 Feedback in cAMP Signaling,Endothelin-1 Signaling,eNOS Signaling,ErbB Signaling,ErbB4 Signaling,ERK/MAPK Signaling,Erythropoietin Signaling,Factors Promoting Cardiogenesis in Vertebrates,Fc Epsilon RI Signaling,Fcγ Receptor-mediated Phagocytosis in Macrophages and Monocytes,fMLP Signaling in Neutrophils,G Beta Gamma Signaling,G-Protein Coupled Receptor Signaling,Gap Junction Signaling,Glioma Signaling,GNRH Signaling,GPCR-Mediated Nutrient Sensing in Enteroendocrine Cells,Growth Hormone Signaling,Gαq Signaling,Hepatic Cholestasis,HER-2 Signaling in Breast Cancer,HGF Signaling,Huntington's Disease Signaling,IL-12 Signaling and Production in Macrophages,IL-3 Signaling,IL-8 Signaling,Leukocyte Extravasation Signaling,LPS-stimulated MAPK Signaling,Macropinocytosis Signaling,Mechanisms of Viral Exit from Host Cells,Melatonin Signaling,Molecular Mechanisms of Cancer,mTOR Signaling,Natural Killer Cell Signaling,Neuregulin Signaling,Neuropathic Pain Signaling In Dorsal Horn Neurons,NF-κB Activation by Viruses,Nitric Oxide Signaling in the Cardiovascular System,nNOS Signaling in Neurons,NRF2-mediated Oxidative Stress Response,P2Y Purigenic Receptor Signaling Pathway,p70S6K Signaling,phagosome formation,Phospholipase C Signaling,Production of Nitric Oxide and Reactive Oxygen Species in Macrophages,Prolactin Signaling,Protein Kinase A Signaling,Pyridoxal 5'-phosphate Salvage Pathway,RAR Activation,Renin-Angiotensin Signaling,Role of Macrophages, Fibroblasts and Endothelial Cells in Rheumatoid Arthritis,Role of NFAT in Cardiac Hypertrophy,Role of Pattern Recognition Receptors in Recognition of Bacteria and Viruses,Salvage Pathways of Pyrimidine Ribonucleotides,Sperm Motility,Synaptic Long Term Depression,Synaptic Long Term Potentiation,Tec Kinase Signaling,Thrombin Signaling,Thrombopoietin Signaling,Type II Diabetes Mellitus Signaling,UVB- |

| | | | | | |
|---|---|---|---|---|---|
| | | | | | Induced MAPK Signaling,UVC-Induced MAPK Signaling,VDR/RXR Activation,VEGF Family Ligand-Receptor Interactions,Virus Entry via Endocytic Pathways,Xenobiotic Metabolism Signaling,α-Adrenergic Signaling |
| MIMAT0003247 | miR-582-5p (miRNAs w/seed UACAGUU) | TargetScan Human | High (predicted) | PTPN2 | 3-phosphoinositide Biosynthesis,3-phosphoinositide Degradation,D-myo-inositol (1,4,5,6)-Tetrakisphosphate Biosynthesis,D-myo-inositol (3,4,5,6)-tetrakisphosphate Biosynthesis,D-myo-inositol-5-phosphate Metabolism,Interferon Signaling,Protein Kinase A Signaling,Role of JAK1, JAK2 and TYK2 in Interferon Signaling,STAT3 Pathway,Superpathway of Inositol Phosphate Compounds |
| MIMAT0003247 | miR-582-5p (miRNAs w/seed UACAGUU) | TargetScan Human | Moderate (predicted) | PTPRJ | 3-phosphoinositide Biosynthesis,3-phosphoinositide Degradation,D-myo-inositol (1,4,5,6)-Tetrakisphosphate Biosynthesis,D-myo-inositol (3,4,5,6)-tetrakisphosphate Biosynthesis,D-myo-inositol-5-phosphate Metabolism,Protein Kinase A Signaling,Superpathway of Inositol Phosphate Compounds |
| MIMAT0003247 | miR-582-5p (miRNAs w/seed UACAGUU) | TargetScan Human | Moderate (predicted) | PTPRK | Protein Kinase A Signaling |
| MIMAT0003247 | miR-582-5p (miRNAs w/seed UACAGUU) | TargetScan Human | Moderate (predicted) | RASA1 | 3-phosphoinositide Biosynthesis,3-phosphoinositide Degradation,Angiopoietin Signaling,Axonal Guidance Signaling,Cdc42 Signaling,D-myo-inositol (1,4,5,6)-Tetrakisphosphate Biosynthesis,D-myo-inositol (3,4,5,6)-tetrakisphosphate Biosynthesis,D-myo-inositol-5-phosphate Metabolism,EGF Signaling,Ephrin Receptor Signaling,G-Protein Coupled Receptor Signaling,GDNF Family Ligand-Receptor Interactions,Gα12/13 Signaling,Huntington's Disease Signaling,IGF-1 Signaling,Molecular Mechanisms of Cancer,PDGF Signaling,Superpathway of Inositol Phosphate Compounds,T Cell Receptor Signaling |
| MIMAT0003247 | miR-582-5p (miRNAs w/seed UACAGUU) | TargetScan Human | Moderate (predicted) | RASGRP1 | G-Protein Coupled Receptor Signaling,Leukocyte Extravasation Signaling,Molecular Mechanisms of Cancer,T Cell Receptor Signaling |
| MIMAT0003247 | miR-582-5p (miRNAs w/seed UACAGUU) | TargetScan Human | Moderate (predicted) | RB1 | Antiproliferative Role of TOB in T Cell Signaling,Aryl Hydrocarbon Receptor Signaling,Bladder Cancer Signaling,Cell Cycle Regulation by BTG Family Proteins,Cell Cycle: G1/S Checkpoint Regulation,Chronic Myeloid Leukemia Signaling,Cyclins and Cell Cycle Regulation,Estrogen-mediated S-phase Entry,Glioblastoma Multiforme Signaling,Glioma Signaling,Hereditary Breast Cancer Signaling,Melanoma Signaling,Molecular Mechanisms of Cancer,Non- |

| | | | | | |
|---|---|---|---|---|---|
| | | | | | Small Cell Lung Cancer Signaling,Ovarian Cancer Signaling,p53 Signaling,Pancreatic Adenocarcinoma Signaling,Prostate Cancer Signaling,Regulation of Cellular Mechanics by Calpain Protease,Role of BRCA1 in DNA Damage Response,Role of Oct4 in Mammalian Embryonic Stem Cell Pluripotency,Role of p14/p19ARF in Tumor Suppression,Small Cell Lung Cancer Signaling,Telomerase Signaling |
| MIMAT0003247 | miR-582-5p (miRNAs w/seed UACAGUU) | TargetScan Human | Moderate (predicted) | RGS7 | cAMP-mediated signaling,G-Protein Coupled Receptor Signaling,Gαi Signaling,Gαq Signaling |
| MIMAT0003247 | miR-582-5p (miRNAs w/seed UACAGUU) | TargetScan Human | Moderate (predicted) | RHOD | Actin Nucleation by ARP-WASP Complex,Axonal Guidance Signaling,Cardiac Hypertrophy Signaling,Cholecystokinin/Gastrin-mediated Signaling,Colorectal Cancer Metastasis Signaling,CXCR4 Signaling,Germ Cell-Sertoli Cell Junction Signaling,Glioblastoma Multiforme Signaling,Glioma Invasiveness Signaling,Gαq Signaling,HMGB1 Signaling,IL-8 Signaling,ILK Signaling,Integrin Signaling,Molecular Mechanisms of Cancer,mTOR Signaling,phagosome formation,Phospholipase C Signaling,Production of Nitric Oxide and Reactive Oxygen Species in Macrophages,Regulation of Actin-based Motility by Rho,RhoGDI Signaling,Semaphorin Signaling in Neurons,Signaling by Rho Family GTPases,Sphingosine-1-phosphate Signaling,Tec Kinase Signaling,Thrombin Signaling |
| MIMAT0003247 | miR-582-5p (miRNAs w/seed UACAGUU) | TargetScan Human | High (predicted) | ROBO2 | Axonal Guidance Signaling |
| MIMAT0003247 | miR-582-5p (miRNAs w/seed UACAGUU) | TargetScan Human | Moderate (predicted) | RTN4 | Axonal Guidance Signaling |
| MIMAT0003247 | miR-582-5p (miRNAs w/seed UACAGUU) | TargetScan Human | Moderate (predicted) | RYR2 | Calcium Signaling,Cardiac β-adrenergic Signaling,Gαs Signaling,Netrin Signaling,Nitric Oxide Signaling in the Cardiovascular System,nNOS Signaling in Skeletal Muscle Cells,Protein Kinase A Signaling,Synaptic Long Term Depression |
| MIMAT0003247 | miR-582-5p (miRNAs w/seed UACAGUU) | TargetScan Human | Moderate (predicted) | SMARCA4 | AMPK Signaling,Aryl Hydrocarbon Receptor Signaling,Estrogen Receptor Signaling,Glucocorticoid Receptor Signaling,Hereditary Breast Cancer Signaling,RAR Activation,Role of BRCA1 in DNA Damage Response |
| MIMAT0003247 | miR-582-5p (miRNAs w/seed | TargetScan Human | Moderate (predicted) | SST | Antiproliferative Role of Somatostatin Receptor 2,Cholecystokinin/Gastrin-mediated Signaling,GPCR-Mediated Integration of Enteroendocrine Signaling |

| | | | | | |
|---|---|---|---|---|---|
| | UACAGUU) | | | | Exemplified by an L Cell,Neuroprotective Role of THOP1 in Alzheimer's Disease |
| MIMAT0003247 | miR-582-5p (miRNAs w/seed UACAGUU) | TargetScan Human | Moderate (predicted) | TAF1A | Assembly of RNA Polymerase I Complex |
| MIMAT0003247 | miR-582-5p (miRNAs w/seed UACAGUU) | TargetScan Human | Moderate (predicted) | TTN | Actin Cytoskeleton Signaling,Integrin Signaling,Protein Kinase A Signaling,RhoA Signaling |
| MIMAT0003247 | miR-582-5p (miRNAs w/seed UACAGUU) | TargetScan Human | Moderate (predicted) | YWHAZ | 14-3-3-mediated Signaling,Cell Cycle: G2/M DNA Damage Checkpoint Regulation,ERK/MAPK Signaling,ERK5 Signaling,HIPPO signaling,IGF-1 Signaling,Myc Mediated Apoptosis Signaling,p70S6K Signaling,PI3K/AKT Signaling,Protein Kinase A Signaling |
| MIMAT0004912 | miR-890 (miRNAs w/seed ACUUGGA) | TargetScan Human | Moderate (predicted) | ADRB1 | AMPK Signaling,cAMP-mediated signaling,Cardiac Hypertrophy Signaling,Cardiac β-adrenergic Signaling,G-Protein Coupled Receptor Signaling,Gap Junction Signaling,GPCR-Mediated Integration of Enteroendocrine Signaling Exemplified by an L Cell,Gαs Signaling,Nitric Oxide Signaling in the Cardiovascular System,TR/RXR Activation |
| MIMAT0004912 | miR-890 (miRNAs w/seed ACUUGGA) | TargetScan Human | Moderate (predicted) | AK2 | AMPK Signaling |
| MIMAT0004912 | miR-890 (miRNAs w/seed ACUUGGA) | TargetScan Human | Moderate (predicted) | AK4 | AMPK Signaling,Pyrimidine Deoxyribonucleotides De Novo Biosynthesis I,Pyrimidine Ribonucleotides De Novo Biosynthesis,Pyrimidine Ribonucleotides Interconversion,Salvage Pathways of Pyrimidine Ribonucleotides |
| MIMAT0004912 | miR-890 (miRNAs w/seed ACUUGGA) | TargetScan Human | High (predicted) | AKAP13 | cAMP-mediated signaling,Cardiac β-adrenergic Signaling,Protein Kinase A Signaling |
| MIMAT0004912 | miR-890 (miRNAs w/seed ACUUGGA) | TargetScan Human | Moderate (predicted) | CALM1 (includes others) | Androgen Signaling,B Cell Receptor Signaling,Breast Cancer Regulation by Stathmin1,Calcium Signaling,Calcium-induced T Lymphocyte Apoptosis,cAMP-mediated signaling,Cardiac Hypertrophy Signaling,CCR3 Signaling in Eosinophils,CCR5 Signaling in Macrophages,CD28 Signaling in T Helper Cells,Cellular Effects of Sildenafil (Viagra),Chemokine Signaling,Corticotropin Releasing Hormone Signaling,CREB Signaling in Neurons,Dopamine-DARPP32 Feedback in cAMP Signaling,eNOS Signaling,fMLP Signaling in |

| | | | | | |
|---|---|---|---|---|---|
| | | | | | Neutrophils,Glioma Signaling,Glutamate Receptor Signaling,Gαq Signaling,iCOS-iCOSL Signaling in T Helper Cells,iNOS Signaling,Melatonin Signaling,Nitric Oxide Signaling in the Cardiovascular System,nNOS Signaling in Neurons,nNOS Signaling in Skeletal Muscle Cells,Nur77 Signaling in T Lymphocytes,Phospholipase C Signaling,PI3K Signaling in B Lymphocytes,Protein Kinase A Signaling,RANK Signaling in Osteoclasts,Regulation of IL-2 Expression in Activated and Anergic T Lymphocytes,Role of Macrophages, Fibroblasts and Endothelial Cells in Rheumatoid Arthritis,Role of NFAT in Cardiac Hypertrophy,Role of NFAT in Regulation of the Immune Response,Role of Osteoblasts, Osteoclasts and Chondrocytes in Rheumatoid Arthritis,Sperm Motility,Synaptic Long Term Potentiation,T Cell Receptor Signaling,α-Adrenergic Signaling |
| MIMAT0004912 | miR-890 (miRNAs w/seed ACUUGGA) | TargetScan Human | Moderate (predicted) | CAMK2D | B Cell Receptor Signaling,Breast Cancer Regulation by Stathmin1,Calcium Signaling,cAMP-mediated signaling,Chemokine Signaling,CREB Signaling in Neurons,Crosstalk between Dendritic Cells and Natural Killer Cells,G-Protein Coupled Receptor Signaling,Glioma Signaling,GM-CSF Signaling,GNRH Signaling,iCOS-iCOSL Signaling in T Helper Cells,Melatonin Signaling,Molecular Mechanisms of Cancer,Neuropathic Pain Signaling In Dorsal Horn Neurons,PI3K Signaling in B Lymphocytes,PKCθ Signaling in T Lymphocytes,Protein Kinase A Signaling,Role of Macrophages, Fibroblasts and Endothelial Cells in Rheumatoid Arthritis,Role of NFAT in Cardiac Hypertrophy,Synaptic Long Term Potentiation,Thrombin Signaling,Xenobiotic Metabolism Signaling |
| MIMAT0004912 | miR-890 (miRNAs w/seed ACUUGGA) | TargetScan Human | Moderate (predicted) | CAPN6 | Amyloid Processing,Amyotrophic Lateral Sclerosis Signaling,Apoptosis Signaling,FAK Signaling,Huntington's Disease Signaling,Integrin Signaling,nNOS Signaling in Neurons,Regulation of Cellular Mechanics by Calpain Protease |
| MIMAT0004912 | miR-890 (miRNAs w/seed ACUUGGA) | TargetScan Human | Moderate (predicted) | CAV1 | Caveolar-mediated Endocytosis Signaling,eNOS Signaling,G Beta Gamma Signaling,Gap Junction Signaling,Gαi Signaling,Integrin Signaling,Nitric Oxide Signaling in the Cardiovascular System,PDGF Signaling,Virus Entry via Endocytic Pathways |
| MIMAT0004912 | miR-890 (miRNAs w/seed ACUUGGA) | TargetScan Human | Moderate (predicted) | CDH11 | Gα12/13 Signaling,RhoGDI Signaling,Signaling by Rho Family GTPases |
| MIMAT0004912 | miR-890 (miRNAs w/seed | TargetScan Human | Moderate (predicted) | CDH6 | Gα12/13 Signaling,RhoGDI Signaling,Signaling by Rho Family GTPases |

| | | | | | |
|---|---|---|---|---|---|
| | ACUUGGA) | | | | |
| MIMAT0004912 | miR-890 (miRNAs w/seed ACUUGGA) | TargetScan Human | Moderate (predicted) | CNGA3 | cAMP-mediated signaling,eNOS Signaling,Gαs Signaling,Phototransduction Pathway,Protein Kinase A Signaling,Regulation of Cellular Mechanics by Calpain Protease,Sperm Motility |
| MIMAT0004912 | miR-890 (miRNAs w/seed ACUUGGA) | TargetScan Human | Moderate (predicted) | CNGB3 | cAMP-mediated signaling,eNOS Signaling,Gαs Signaling,Phototransduction Pathway,Protein Kinase A Signaling,Regulation of Cellular Mechanics by Calpain Protease,Sperm Motility |
| MIMAT0004912 | miR-890 (miRNAs w/seed ACUUGGA) | TargetScan Human | Moderate (predicted) | EGF | Actin Cytoskeleton Signaling,Axonal Guidance Signaling,Bladder Cancer Signaling,Caveolar-mediated Endocytosis Signaling,Clathrin-mediated Endocytosis Signaling,Colorectal Cancer Metastasis Signaling,EGF Signaling,Ephrin Receptor Signaling,Epithelial Adherens Junction Signaling,ErbB Signaling,ERK5 Signaling,FAK Signaling,Gap Junction Signaling,Glioblastoma Multiforme Signaling,Glioma Signaling,GNRH Signaling,Hepatic Fibrosis / Hepatic Stellate Cell Activation,HER-2 Signaling in Breast Cancer,Huntington's Disease Signaling,IL-8 Signaling,Macropinocytosis Signaling,Neuregulin Signaling,NF-κB Signaling,Non-Small Cell Lung Cancer Signaling,Ovarian Cancer Signaling,Pancreatic Adenocarcinoma Signaling,Regulation of Cellular Mechanics by Calpain Protease,Regulation of the Epithelial-Mesenchymal Transition Pathway,Telomerase Signaling,Thrombin Signaling |
| MIMAT0004912 | miR-890 (miRNAs w/seed ACUUGGA) | TargetScan Human | High (predicted) | ELK3 | ERK/MAPK Signaling,HGF Signaling,Telomerase Signaling |
| MIMAT0004912 | miR-890 (miRNAs w/seed ACUUGGA) | TargetScan Human | High (predicted) | GAD2 | GABA Receptor Signaling,Glutamate Degradation III (via 4-aminobutyrate),Glutamate Dependent Acid Resistance,Type I Diabetes Mellitus Signaling |
| MIMAT0004912 | miR-890 (miRNAs w/seed ACUUGGA) | TargetScan Human | Moderate (predicted) | GLI2 | Axonal Guidance Signaling,Basal Cell Carcinoma Signaling,Corticotropin Releasing Hormone Signaling,Sonic Hedgehog Signaling |
| MIMAT0004912 | miR-890 (miRNAs w/seed ACUUGGA) | TargetScan Human | Moderate (predicted) | GNG12 | Actin Cytoskeleton Signaling,Androgen Signaling,Antiproliferative Role of Somatostatin Receptor 2,Axonal Guidance Signaling,Breast Cancer Regulation by Stathmin1,Cardiac Hypertrophy Signaling,Cardiac β-adrenergic Signaling,CCR3 Signaling in Eosinophils,CCR5 Signaling in Macrophages,Colorectal Cancer Metastasis Signaling,CREB Signaling in Neurons,CXCR4 Signaling,Ephrin B Signaling,Ephrin Receptor Signaling,fMLP |

| | | | | | |
|---|---|---|---|---|---|
| | | | | | Signaling in Neutrophils,G Beta Gamma Signaling,G Protein Signaling Mediated by Tubby,GPCR-Mediated Nutrient Sensing in Enteroendocrine Cells,Gαi Signaling,Gαq Signaling,Gαs Signaling,Huntington's Disease Signaling,IL-1 Signaling,IL-8 Signaling,P2Y Purigenic Receptor Signaling Pathway,Phospholipase C Signaling,Protein Kinase A Signaling,Relaxin Signaling,RhoGDI Signaling,Role of NFAT in Cardiac Hypertrophy,Role of NFAT in Regulation of the Immune Response,Signaling by Rho Family GTPases,Tec Kinase Signaling,Thrombin Signaling,α-Adrenergic Signaling |
| MIMAT0004912 | miR-890 (miRNAs w/seed ACUUGGA) | TargetScan Human | Moderate (predicted) | GNG4 | Androgen Signaling,Antiproliferative Role of Somatostatin Receptor 2,Axonal Guidance Signaling,Breast Cancer Regulation by Stathmin1,Cardiac Hypertrophy Signaling,Cardiac β-adrenergic Signaling,CCR3 Signaling in Eosinophils,CCR5 Signaling in Macrophages,Colorectal Cancer Metastasis Signaling,CREB Signaling in Neurons,CXCR4 Signaling,Ephrin B Signaling,Ephrin Receptor Signaling,fMLP Signaling in Neutrophils,G Beta Gamma Signaling,G Protein Signaling Mediated by Tubby,GPCR-Mediated Nutrient Sensing in Enteroendocrine Cells,Gαi Signaling,Gαq Signaling,Gαs Signaling,Huntington's Disease Signaling,IL-1 Signaling,IL-8 Signaling,P2Y Purigenic Receptor Signaling Pathway,Phospholipase C Signaling,Protein Kinase A Signaling,Relaxin Signaling,RhoGDI Signaling,Role of NFAT in Cardiac Hypertrophy,Role of NFAT in Regulation of the Immune Response,Signaling by Rho Family GTPases,Tec Kinase Signaling,Thrombin Signaling,α-Adrenergic Signaling |
| MIMAT0004912 | miR-890 (miRNAs w/seed ACUUGGA) | TargetScan Human | High (predicted) | GYS2 | AMPK Signaling,Glycogen Biosynthesis II (from UDP-D-Glucose),Gαq Signaling,Insulin Receptor Signaling,PI3K/AKT Signaling,Protein Kinase A Signaling,α-Adrenergic Signaling |
| MIMAT0004912 | miR-890 (miRNAs w/seed ACUUGGA) | TargetScan Human | Moderate (predicted) | H3F3A/H3F3B | ERK/MAPK Signaling,Estrogen Receptor Signaling,p38 MAPK Signaling,Protein Kinase A Signaling,UVB-Induced MAPK Signaling |
| MIMAT0004912 | miR-890 (miRNAs w/seed ACUUGGA) | TargetScan Human | Moderate (predicted) | HDAC8 | Adipogenesis pathway,Calcium Signaling,Cell Cycle: G1/S Checkpoint Regulation,Chronic Myeloid Leukemia Signaling,Cyclins and Cell Cycle Regulation,Hereditary Breast Cancer Signaling,Huntington's Disease Signaling,Phospholipase C Signaling,Role of NFAT in Cardiac Hypertrophy,Telomerase Signaling |
| MIMAT0004912 | miR-890 (miRNAs w/seed ACUUGGA) | TargetScan Human | Moderate (predicted) | HHAT | Molecular Mechanisms of Cancer,Protein Kinase A Signaling |

| | | | | | |
|---|---|---|---|---|---|
| MIMAT0004912 | miR-890 (miRNAs w/seed ACUUGGA) | TargetScan Human | Moderate (predicted) | HLTF | AMPK Signaling,Glucocorticoid Receptor Signaling,Hereditary Breast Cancer Signaling,RAR Activation,Role of BRCA1 in DNA Damage Response,Role of JAK2 in Hormone-like Cytokine Signaling |
| MIMAT0004912 | miR-890 (miRNAs w/seed ACUUGGA) | TargetScan Human | Moderate (predicted) | HSPA9 | Aldosterone Signaling in Epithelial Cells,eNOS Signaling,Glucocorticoid Receptor Signaling,Huntington's Disease Signaling,Protein Ubiquitination Pathway,Unfolded protein response |
| MIMAT0004912 | miR-890 (miRNAs w/seed ACUUGGA) | TargetScan Human | Moderate (predicted) | IRS1 | AMPK Signaling,Cardiac Hypertrophy Signaling,GDNF Family Ligand-Receptor Interactions,Growth Hormone Signaling,IGF-1 Signaling,IL-4 Signaling,IL-9 Signaling,ILK Signaling,Insulin Receptor Signaling,Molecular Mechanisms of Cancer,mTOR Signaling,p70S6K Signaling,PI3K Signaling in B Lymphocytes,PPARα/RXRα Activation,Prolactin Signaling,Regulation of eIF4 and p70S6K Signaling,Role of JAK1 and JAK3 in γc Cytokine Signaling,Role of JAK2 in Hormone-like Cytokine Signaling,SAPK/JNK Signaling,Type II Diabetes Mellitus Signaling |
| MIMAT0004912 | miR-890 (miRNAs w/seed ACUUGGA) | TargetScan Human | High (predicted) | ITGA2 | Actin Cytoskeleton Signaling,Actin Nucleation by ARP-WASP Complex,Agranulocyte Adhesion and Diapedesis,Agrin Interactions at Neuromuscular Junction,Axonal Guidance Signaling,Caveolar-mediated Endocytosis Signaling,Cdc42 Signaling,CDK5 Signaling,Ephrin Receptor Signaling,ERK/MAPK Signaling,FAK Signaling,Germ Cell-Sertoli Cell Junction Signaling,Granulocyte Adhesion and Diapedesis,HGF Signaling,Integrin Signaling,Leukocyte Extravasation Signaling,Molecular Mechanisms of Cancer,Neuregulin Signaling,NF-κB Activation by Viruses,PAK Signaling,Paxillin Signaling,phagosome formation,Phospholipase C Signaling,PI3K/AKT Signaling,PTEN Signaling,Rac Signaling,Reelin Signaling in Neurons,Regulation of Actin-based Motility by Rho,Regulation of Cellular Mechanics by Calpain Protease,Regulation of eIF4 and p70S6K Signaling,RhoGDI Signaling,Role of Osteoblasts, Osteoclasts and Chondrocytes in Rheumatoid Arthritis,Sertoli Cell-Sertoli Cell Junction Signaling,Signaling by Rho Family GTPases,Tec Kinase Signaling,Virus Entry via Endocytic Pathways |
| MIMAT0004912 | miR-890 (miRNAs w/seed ACUUGGA) | TargetScan Human | Moderate (predicted) | KSR1 | Ceramide Signaling,ERK/MAPK Signaling |
| MIMAT0004912 | miR-890 (miRNAs w/seed ACUUGGA) | TargetScan Human | Moderate (predicted) | LIFR | CNTF Signaling,Mouse Embryonic Stem Cell Pluripotency,Role of NANOG in Mammalian Embryonic Stem Cell Pluripotency |

| | | | | | |
|---|---|---|---|---|---|
| MIMAT0004912 | miR-890 (miRNAs w/seed ACUUGGA) | TargetScan Human | Moderate (predicted) | MAP2K1 | 14-3-3-mediated Signaling,4-1BB Signaling in T Lymphocytes,Actin Cytoskeleton Signaling,Acute Myeloid Leukemia Signaling,Acute Phase Response Signaling,Aldosterone Signaling in Epithelial Cells,Antiproliferative Role of Somatostatin Receptor 2,Apoptosis Signaling,Axonal Guidance Signaling,B Cell Receptor Signaling,Bladder Cancer Signaling,BMP signaling pathway,Breast Cancer Regulation by Stathmin1,cAMP-mediated signaling,Cardiac Hypertrophy Signaling,CCR3 Signaling in Eosinophils,CD27 Signaling in Lymphocytes,CD28 Signaling in T Helper Cells,CD40 Signaling,CDK5 Signaling,Ceramide Signaling,Chemokine Signaling,Cholecystokinin/Gastrin-mediated Signaling,Chronic Myeloid Leukemia Signaling,CNTF Signaling,Colorectal Cancer Metastasis Signaling,Corticotropin Releasing Hormone Signaling,CREB Signaling in Neurons,CXCR4 Signaling,EGF Signaling,EIF2 Signaling,Endometrial Cancer Signaling,Ephrin Receptor Signaling,ErbB Signaling,ErbB2-ErbB3 Signaling,ErbB4 Signaling,ERK/MAPK Signaling,Erythropoietin Signaling,Estrogen Receptor Signaling,FAK Signaling,Fc Epsilon RI Signaling,FGF Signaling,FLT3 Signaling in Hematopoietic Progenitor Cells,fMLP Signaling in Neutrophils,G-Protein Coupled Receptor Signaling,Gap Junction Signaling,GDNF Family Ligand-Receptor Interactions,Germ Cell-Sertoli Cell Junction Signaling,Glioblastoma Multiforme Signaling,Glioma Signaling,Glucocorticoid Receptor Signaling,GM-CSF Signaling,GNRH Signaling,Gα12/13 Signaling,Gαq Signaling,Gαs Signaling,HGF Signaling,HMGB1 Signaling,IGF-1 Signaling,IL-12 Signaling and Production in Macrophages,IL-15 Signaling,IL-17 Signaling,IL-17A Signaling in Airway Cells,IL-2 Signaling,IL-3 Signaling,IL-6 Signaling,IL-8 Signaling,Insulin Receptor Signaling,Integrin Signaling,JAK/Stat Signaling,Leptin Signaling in Obesity,LPS-stimulated MAPK Signaling,Melanocyte Development and Pigmentation Signaling,Melanoma Signaling,Melatonin Signaling,Molecular Mechanisms of Cancer,Mouse Embryonic Stem Cell Pluripotency,Natural Killer Cell Signaling,Neuregulin Signaling,Neurotrophin/TRK Signaling,NGF Signaling,Nitric Oxide Signaling in the Cardiovascular System,Non-Small Cell Lung Cancer Signaling,NRF2-mediated Oxidative Stress Response,Oncostatin M Signaling,Ovarian Cancer Signaling,P2Y Purigenic Receptor Signaling Pathway,p70S6K Signaling,PAK Signaling,Pancreatic Adenocarcinoma Signaling,PDGF Signaling,Phospholipase C Signaling,PI3K Signaling in B Lymphocytes,PI3K/AKT Signaling,PPAR Signaling,PPARα/RXRα Activation,Production of Nitric Oxide and Reactive Oxygen Species in Macrophages,Prolactin Signaling,Prostate Cancer Signaling,Protein Kinase A Signaling,PTEN Signaling,Pyridoxal 5'-phosphate Salvage Pathway,Rac Signaling,RANK Signaling in Osteoclasts,RAR Activation,Regulation of eIF4 and p70S6K Signaling,Regulation of IL-2 Expression in Activated and Anergic T |

| | | | | | |
|---|---|---|---|---|---|
| | | | | | Lymphocytes,Regulation of the Epithelial-Mesenchymal Transition Pathway,Relaxin Signaling,Renal Cell Carcinoma Signaling,Renin-Angiotensin Signaling,Role of IL-17A in Arthritis,Role of IL-17F in Allergic Inflammatory Airway Diseases,Role of Macrophages, Fibroblasts and Endothelial Cells in Rheumatoid Arthritis,Role of MAPK Signaling in the Pathogenesis of Influenza,Role of NANOG in Mammalian Embryonic Stem Cell Pluripotency,Role of NFAT in Cardiac Hypertrophy,Role of NFAT in Regulation of the Immune Response,Role of PI3K/AKT Signaling in the Pathogenesis of Influenza,Salvage Pathways of Pyrimidine Ribonucleotides,Sertoli Cell-Sertoli Cell Junction Signaling,Signaling by Rho Family GTPases,STAT3 Pathway,Synaptic Long Term Depression,Synaptic Long Term Potentiation,T Cell Receptor Signaling,Telomerase Signaling,TGF-β Signaling,Thrombin Signaling,Thrombopoietin Signaling,Thyroid Cancer Signaling,UVB-Induced MAPK Signaling,UVC-Induced MAPK Signaling,VEGF Family Ligand-Receptor Interactions,VEGF Signaling,Xenobiotic Metabolism Signaling,α-Adrenergic Signaling |
| MIMAT0004912 | miR-890 (miRNAs w/seed ACUUGGA) | TargetScan Human | Moderate (predicted) | MAPT | 14-3-3-mediated Signaling,Amyloid Processing,CDK5 Signaling,Neuroprotective Role of THOP1 in Alzheimer's Disease,p38 MAPK Signaling,p70S6K Signaling,Reelin Signaling in Neurons |
| MIMAT0004912 | miR-890 (miRNAs w/seed ACUUGGA) | TargetScan Human | Moderate (predicted) | MARCKS | phagosome formation,Phospholipase C Signaling |
| MIMAT0004912 | miR-890 (miRNAs w/seed ACUUGGA) | TargetScan Human | Moderate (predicted) | MPRIP | Actin Cytoskeleton Signaling,CCR3 Signaling in Eosinophils,Cdc42 Signaling,Cellular Effects of Sildenafil (Viagra),Chemokine Signaling,Integrin Signaling,Phospholipase C Signaling,Regulation of Actin-based Motility by Rho,RhoA Signaling,Thrombin Signaling |
| MIMAT0004912 | miR-890 (miRNAs w/seed ACUUGGA) | TargetScan Human | Moderate (predicted) | NCOA1 | Androgen Signaling,Estrogen Receptor Signaling,Glucocorticoid Receptor Signaling,HIF1α Signaling,IL-12 Signaling and Production in Macrophages,LPS/IL-1 Mediated Inhibition of RXR Function,PPAR Signaling,PXR/RXR Activation,RAR Activation,Role of Wnt/GSK-3β Signaling in the Pathogenesis of Influenza,TR/RXR Activation,VDR/RXR Activation,Xenobiotic Metabolism Signaling |
| MIMAT0004912 | miR-890 (miRNAs w/seed ACUUGGA) | TargetScan Human | Moderate (predicted) | NFAT5 | April Mediated Signaling,Axonal Guidance Signaling,B Cell Activating Factor Signaling,B Cell Receptor Signaling,Calcium Signaling,CD28 Signaling in T Helper Cells,fMLP Signaling in Neutrophils,Glucocorticoid Receptor Signaling,iCOS-iCOSL Signaling in T Helper Cells,IL-4 Signaling,Netrin |

| | | | | | |
|---|---|---|---|---|---|
| | | | | | Signaling,Phospholipase C Signaling,PI3K Signaling in B Lymphocytes,PKCθ Signaling in T Lymphocytes,Protein Kinase A Signaling,Regulation of IL-2 Expression in Activated and Anergic T Lymphocytes,Role of Macrophages, Fibroblasts and Endothelial Cells in Rheumatoid Arthritis,Role of NFAT in Regulation of the Immune Response,Role of Osteoblasts, Osteoclasts and Chondrocytes in Rheumatoid Arthritis,Systemic Lupus Erythematosus Signaling,T Cell Receptor Signaling,Wnt/Ca+ pathway |
| MIMAT0004912 | miR-890 (miRNAs w/seed ACUUGGA) | TargetScan Human | Moderate (predicted) | NFKBIE | 4-1BB Signaling in T Lymphocytes,Activation of IRF by Cytosolic Pattern Recognition Receptors,Acute Phase Response Signaling,Angiopoietin Signaling,Antioxidant Action of Vitamin C,Apoptosis Signaling,April Mediated Signaling,B Cell Activating Factor Signaling,B Cell Receptor Signaling,CD27 Signaling in Lymphocytes,CD28 Signaling in T Helper Cells,CD40 Signaling,Death Receptor Signaling,Dendritic Cell Maturation,Erythropoietin Signaling,fMLP Signaling in Neutrophils,G-Protein Coupled Receptor Signaling,Glucocorticoid Receptor Signaling,Gα12/13 Signaling,Gαq Signaling,Hepatic Cholestasis,Hypoxia Signaling in the Cardiovascular System,iCOS-iCOSL Signaling in T Helper Cells,IL-1 Signaling,IL-10 Signaling,IL-17A Signaling in Airway Cells,IL-17A Signaling in Fibroblasts,IL-6 Signaling,Induction of Apoptosis by HIV1,iNOS Signaling,LPS-stimulated MAPK Signaling,MIF Regulation of Innate Immunity,MIF-mediated Glucocorticoid Regulation,Molecular Mechanisms of Cancer,NF-κB Activation by Viruses,NF-κB Signaling,OX40 Signaling Pathway,PEDF Signaling,PI3K Signaling in B Lymphocytes,PI3K/AKT Signaling,PKCθ Signaling in T Lymphocytes,PPAR Signaling,PPARα/RXRα Activation,Production of Nitric Oxide and Reactive Oxygen Species in Macrophages,Prostate Cancer Signaling,Protein Kinase A Signaling,RANK Signaling in Osteoclasts,Regulation of IL-2 Expression in Activated and Anergic T Lymphocytes,Relaxin Signaling,Role of IL-17A in Arthritis,Role of Macrophages, Fibroblasts and Endothelial Cells in Rheumatoid Arthritis,Role of NFAT in Regulation of the Immune Response,Role of Osteoblasts, Osteoclasts and Chondrocytes in Rheumatoid Arthritis,Role of PI3K/AKT Signaling in the Pathogenesis of Influenza,Role of PKR in Interferon Induction and Antiviral Response,Role of RIG1-like Receptors in Antiviral Innate Immunity,Small Cell Lung Cancer Signaling,TNFR1 Signaling,TNFR2 Signaling,TWEAK Signaling,Type I Diabetes Mellitus Signaling,Type II Diabetes Mellitus Signaling |
| MIMAT0004912 | miR-890 (miRNAs w/seed ACUUGGA) | TargetScan Human | High (predicted) | NR5A2 | FXR/RXR Activation,Hepatic Cholestasis,LPS/IL-1 Mediated Inhibition of RXR Function,Role of Oct4 in Mammalian Embryonic Stem Cell Pluripotency,Wnt/β-catenin Signaling |

| | | | | | |
|---|---|---|---|---|---|
| MIMAT0004912 | miR-890 (miRNAs w/seed ACUUGGA) | TargetScan Human | Moderate (predicted) | OCRL | 1D-myo-inositol Hexakisphosphate Biosynthesis II (Mammalian),3-phosphoinositide Degradation,B Cell Receptor Signaling,D-myo-inositol (1,3,4)-trisphosphate Biosynthesis,D-myo-inositol (1,4,5)-trisphosphate Degradation,Fc Epsilon RI Signaling,IL-4 Signaling,Insulin Receptor Signaling,Natural Killer Cell Signaling,PDGF Signaling,PI3K/AKT Signaling,PTEN Signaling,Superpathway of D-myo-inositol (1,4,5)-trisphosphate Metabolism,Superpathway of Inositol Phosphate Compounds |
| MIMAT0004912 | miR-890 (miRNAs w/seed ACUUGGA) | TargetScan Human | Moderate (predicted) | OPN5 | Phototransduction Pathway |
| MIMAT0004912 | miR-890 (miRNAs w/seed ACUUGGA) | TargetScan Human | Moderate (predicted) | P2RX5 | Gustation Pathway |
| MIMAT0004912 | miR-890 (miRNAs w/seed ACUUGGA) | TargetScan Human | High (predicted) | P2RY1 | Gustation Pathway,P2Y Purigenic Receptor Signaling Pathway |
| MIMAT0004912 | miR-890 (miRNAs w/seed ACUUGGA) | TargetScan Human | Moderate (predicted) | PAFAH1B1 | Reelin Signaling in Neurons |
| MIMAT0004912 | miR-890 (miRNAs w/seed ACUUGGA) | TargetScan Human | Moderate (predicted) | PAX6 | Transcriptional Regulatory Network in Embryonic Stem Cells |
| MIMAT0004912 | miR-890 (miRNAs w/seed ACUUGGA) | TargetScan Human | Moderate (predicted) | POU2F1 | Glucocorticoid Receptor Signaling,PKCθ Signaling in T Lymphocytes,Role of BRCA1 in DNA Damage Response |
| MIMAT0004912 | miR-890 (miRNAs w/seed ACUUGGA) | TargetScan Human | Moderate (predicted) | PPP2R3A | 3-phosphoinositide Biosynthesis,3-phosphoinositide Degradation,AMPK Signaling,Breast Cancer Regulation by Stathmin1,Cardiac β-adrenergic Signaling,CDK5 Signaling,Cell Cycle Regulation by BTG Family Proteins,Ceramide Signaling,CTLA4 Signaling in Cytotoxic T Lymphocytes,Cyclins and Cell Cycle Regulation,D-myo-inositol (1,4,5,6)-Tetrakisphosphate Biosynthesis,D-myo-inositol (3,4,5,6)-tetrakisphosphate Biosynthesis,D-myo-inositol-5-phosphate Metabolism,Dopamine Receptor Signaling,Dopamine-DARPP32 Feedback in cAMP Signaling,ERK/MAPK Signaling,HIPPO signaling,ILK Signaling,Mitotic Roles of Polo-Like Kinase,mTOR Signaling,p70S6K Signaling,PI3K/AKT Signaling,Production of |

| | | | | | |
|---|---|---|---|---|---|
| | | | | | Nitric Oxide and Reactive Oxygen Species in Macrophages,Regulation of eIF4 and p70S6K Signaling,Role of CHK Proteins in Cell Cycle Checkpoint Control,Superpathway of Inositol Phosphate Compounds,Synaptic Long Term Depression,Telomerase Signaling,Tight Junction Signaling,Wnt/β-catenin Signaling,Xenobiotic Metabolism Signaling |
| MIMAT0004912 | miR-890 (miRNAs w/seed ACUUGGA) | TargetScan Human | Moderate (predicted) | PPP2R5C | AMPK Signaling,Breast Cancer Regulation by Stathmin1,Cardiac β-adrenergic Signaling,CDK5 Signaling,Cell Cycle Regulation by BTG Family Proteins,Ceramide Signaling,CTLA4 Signaling in Cytotoxic T Lymphocytes,Cyclins and Cell Cycle Regulation,Dopamine Receptor Signaling,Dopamine-DARPP32 Feedback in cAMP Signaling,ERK/MAPK Signaling,HIPPO signaling,ILK Signaling,Mitotic Roles of Polo-Like Kinase,mTOR Signaling,p70S6K Signaling,PI3K/AKT Signaling,Production of Nitric Oxide and Reactive Oxygen Species in Macrophages,Regulation of eIF4 and p70S6K Signaling,Role of CHK Proteins in Cell Cycle Checkpoint Control,Synaptic Long Term Depression,Telomerase Signaling,Tight Junction Signaling,Wnt/β-catenin Signaling,Xenobiotic Metabolism Signaling |
| MIMAT0004912 | miR-890 (miRNAs w/seed ACUUGGA) | TargetScan Human | Moderate (predicted) | PRDX6 | Antioxidant Action of Vitamin C,Atherosclerosis Signaling,Eicosanoid Signaling,Endothelin-1 Signaling,Glutathione Redox Reactions I,Heparan Sulfate Biosynthesis,Heparan Sulfate Biosynthesis (Late Stages),phagosome maturation,Phospholipases,Role of MAPK Signaling in the Pathogenesis of Influenza,Sperm Motility,Synaptic Long Term Depression,Triacylglycerol Degradation |
| MIMAT0004912 | miR-890 (miRNAs w/seed ACUUGGA) | TargetScan Human | Moderate (predicted) | PTH | Dopamine Receptor Signaling,Role of Osteoblasts, Osteoclasts and Chondrocytes in Rheumatoid Arthritis,VDR/RXR Activation |
| MIMAT0004912 | miR-890 (miRNAs w/seed ACUUGGA) | TargetScan Human | Moderate (predicted) | PTPN18 | Protein Kinase A Signaling |
| MIMAT0004912 | miR-890 (miRNAs w/seed ACUUGGA) | TargetScan Human | Moderate (predicted) | PTPRT | Oleate Biosynthesis II (Animals),Protein Kinase A Signaling |
| MIMAT0004912 | miR-890 (miRNAs w/seed ACUUGGA) | TargetScan Human | Moderate (predicted) | RAP2B | B Cell Receptor Signaling,Calcium Signaling,Integrin Signaling,Molecular Mechanisms of Cancer |

| | | | | | |
|---|---|---|---|---|---|
| MIMAT0004912 | miR-890 (miRNAs w/seed ACUUGGA) | TargetScan Human | Moderate (predicted) | RCAN1 | Calcium Signaling,Role of NFAT in Cardiac Hypertrophy,Role of NFAT in Regulation of the Immune Response |
| MIMAT0004912 | miR-890 (miRNAs w/seed ACUUGGA) | TargetScan Human | Moderate (predicted) | REST | Huntington's Disease Signaling,Role of Oct4 in Mammalian Embryonic Stem Cell Pluripotency,Transcriptional Regulatory Network in Embryonic Stem Cells |
| MIMAT0004912 | miR-890 (miRNAs w/seed ACUUGGA) | TargetScan Human | Moderate (predicted) | SET | 3-phosphoinositide Biosynthesis,3-phosphoinositide Degradation,D-myo-inositol (1,4,5,6)-Tetrakisphosphate Biosynthesis,D-myo-inositol (3,4,5,6)-tetrakisphosphate Biosynthesis,D-myo-inositol-5-phosphate Metabolism,Granzyme A Signaling,Superpathway of Inositol Phosphate Compounds,Transcriptional Regulatory Network in Embryonic Stem Cells |
| MIMAT0004912 | miR-890 (miRNAs w/seed ACUUGGA) | TargetScan Human | Moderate (predicted) | SOCS2 | Acute Phase Response Signaling,Growth Hormone Signaling,IGF-1 Signaling,IL-9 Signaling,JAK/Stat Signaling,Prolactin Signaling,Role of JAK2 in Hormone-like Cytokine Signaling,STAT3 Pathway,Type I Diabetes Mellitus Signaling,Type II Diabetes Mellitus Signaling |
| MIMAT0004912 | miR-890 (miRNAs w/seed ACUUGGA) | TargetScan Human | Moderate (predicted) | SOCS3 | 3-phosphoinositide Biosynthesis,3-phosphoinositide Degradation,Acute Phase Response Signaling,D-myo-inositol (1,4,5,6)-Tetrakisphosphate Biosynthesis,D-myo-inositol (3,4,5,6)-tetrakisphosphate Biosynthesis,D-myo-inositol-5-phosphate Metabolism,Erythropoietin Signaling,Growth Hormone Signaling,IGF-1 Signaling,IL-10 Signaling,IL-22 Signaling,IL-6 Signaling,IL-9 Signaling,Insulin Receptor Signaling,JAK/Stat Signaling,Leptin Signaling in Obesity,Prolactin Signaling,Role of JAK family kinases in IL-6-type Cytokine Signaling,Role of JAK1 and JAK3 in γc Cytokine Signaling,Role of JAK2 in Hormone-like Cytokine Signaling,Role of Macrophages, Fibroblasts and Endothelial Cells in Rheumatoid Arthritis,STAT3 Pathway,Superpathway of Inositol Phosphate Compounds,Type I Diabetes Mellitus Signaling,Type II Diabetes Mellitus Signaling |
| MIMAT0004912 | miR-890 (miRNAs w/seed ACUUGGA) | TargetScan Human | Moderate (predicted) | SOCS4 | Acute Phase Response Signaling,Growth Hormone Signaling,IGF-1 Signaling,JAK/Stat Signaling,Prolactin Signaling,Role of JAK2 in Hormone-like Cytokine Signaling,STAT3 Pathway,Type I Diabetes Mellitus Signaling,Type II Diabetes Mellitus Signaling |
| MIMAT0004912 | miR-890 (miRNAs w/seed ACUUGGA) | TargetScan Human | Moderate (predicted) | TCF7L2 | Acute Myeloid Leukemia Signaling,Basal Cell Carcinoma Signaling,Colorectal Cancer Metastasis Signaling,Epithelial Adherens Junction Signaling,Factors Promoting Cardiogenesis in Vertebrates,Human Embryonic Stem Cell Pluripotency,Mouse Embryonic Stem Cell Pluripotency,Ovarian Cancer |

| | | | | | |
|---|---|---|---|---|---|
| | | | | | Signaling,Protein Kinase A Signaling,Regulation of the Epithelial-Mesenchymal Transition Pathway,Role of Macrophages, Fibroblasts and Endothelial Cells in Rheumatoid Arthritis,Role of Osteoblasts, Osteoclasts and Chondrocytes in Rheumatoid Arthritis,Role of Wnt/GSK-3β Signaling in the Pathogenesis of Influenza,Thyroid Cancer Signaling,Wnt/β-catenin Signaling |
| MIMAT0004912 | miR-890 (miRNAs w/seed ACUUGGA) | TargetScan Human | Moderate (predicted) | TNFRSF11A | NF-κB Signaling,PTEN Signaling,RANK Signaling in Osteoclasts,Role of Osteoblasts, Osteoclasts and Chondrocytes in Rheumatoid Arthritis,STAT3 Pathway |
| MIMAT0004912 | miR-890 (miRNAs w/seed ACUUGGA) | TargetScan Human | Moderate (predicted) | TUBA3C/TUBA3D | 14-3-3-mediated Signaling,Axonal Guidance Signaling,Breast Cancer Regulation by Stathmin1,Epithelial Adherens Junction Signaling,Gap Junction Signaling,Germ Cell-Sertoli Cell Junction Signaling,phagosome maturation,Remodeling of Epithelial Adherens Junctions,Sertoli Cell-Sertoli Cell Junction Signaling |
| MIMAT0004912 | miR-890 (miRNAs w/seed ACUUGGA) | TargetScan Human | Moderate (predicted) | UTRN | Agrin Interactions at Neuromuscular Junction |

**Supplementary Table 4: Pathways predicted by KEGG**

| KEGG pathway | p-value | Genes | miRNAs |
|---|---|---|---|
| Fatty acid biosynthesis | 0 | 4 | 2 |
| Prion diseases | 0 | 11 | 4 |
| Morphine addiction | 1.20E-11 | 54 | 5 |
| TGF-beta signaling pathway | 1.62E-09 | 42 | 2 |
| Proteoglycans in cancer | 4.40E-06 | 91 | 2 |
| Thyroid hormone synthesis | 5.89E-06 | 10 | 3 |
| GABAergic synapse | 6.24E-06 | 50 | 5 |
| Amphetamine addiction | 1.06E-05 | 34 | 3 |
| Mucin type O-Glycan biosynthesis | 1.55E-05 | 15 | 3 |
| Lysine degradation | 8.57E-05 | 25 | 3 |
| Nicotine addiction | 0.000699639 | 10 | 1 |
| ECM-receptor interaction | 0.000787444 | 15 | 1 |
| Estrogen signaling pathway | 0.000947203 | 31 | 2 |
| Transcriptional misregulation in cancer | 0.001109214 | 44 | 2 |
| Signaling pathways regulating pluripotency of stem cells | 0.001344512 | 64 | 2 |
| Axon guidance | 0.001350358 | 64 | 2 |
| Wnt signaling pathway | 0.001641238 | 66 | 2 |
| Hippo signaling pathway | 0.002468741 | 61 | 2 |
| Thyroid hormone signaling pathway | 0.002811897 | 67 | 5 |
| ErbB signaling pathway | 0.004443176 | 50 | 3 |
| Pathways in cancer | 0.005934716 | 110 | 1 |
| Glioma | 0.006459832 | 31 | 2 |
| Hepatitis B | 0.01114283 | 48 | 3 |
| Dopaminergic synapse | 0.01530467 | 31 | 1 |
| Fatty acid metabolism | 0.01747381 | 14 | 2 |
| FoxO signaling pathway | 0.01993118 | 52 | 1 |
| Retrograde endocannabinoid signaling | 0.02897847 | 53 | 4 |
| Prostate cancer | 0.02916435 | 34 | 1 |
| Glycosphingolipid biosynthesis - ganglio series | 0.05152128 | 8 | 2 |
| AMPK signaling pathway | 0.05679455 | 48 | 2 |
| Renal cell carcinoma | 0.05728152 | 38 | 2 |
| Adrenergic signaling in cardiomyocytes | 0.06367646 | 41 | 2 |
| Choline metabolism in cancer | 0.0667138 | 13 | 1 |
| Endocytosis | 0.1080233 | 86 | 2 |
| Chronic myeloid leukemia | 0.1130318 | 37 | 2 |
| N-Glycan biosynthesis | 0.1176902 | 16 | 2 |
| Neurotrophin signaling pathway | 0.1308701 | 41 | 1 |
| Oocyte meiosis | 0.1438274 | 29 | 1 |
| Arrhythmogenic right ventricular cardiomyopathy (ARVC) | 0.1606276 | 28 | 2 |

| | | | |
|---|---|---|---|
| Circadian entrainment | 0.189665 | 33 | 2 |
| Ubiquitin mediated proteolysis | 0.1942163 | 50 | 2 |
| Melanogenesis | 0.2082728 | 27 | 1 |
| Colorectal cancer | 0.2196179 | 30 | 2 |
| cGMP-PKG signaling pathway | 0.2296742 | 36 | 1 |
| Cocaine addiction | 0.2583916 | 14 | 1 |
| Gap junction | 0.2713185 | 44 | 3 |
| Long-term potentiation | 0.2893186 | 32 | 2 |
| MAPK signaling pathway | 0.3007868 | 74 | 1 |
| Circadian rhythm | 0.3429932 | 17 | 1 |
| mRNA surveillance pathway | 0.3641713 | 28 | 1 |
| Apoptosis | 0.3797438 | 34 | 1 |
| Melanoma | 0.388184 | 34 | 2 |
| Biotin metabolism | 0.4531548 | 1 | 1 |
| Tight junction | 0.461465 | 12 | 1 |
| Regulation of actin cytoskeleton | 0.4648892 | 13 | 1 |
| Endometrial cancer | 0.469465 | 27 | 2 |
| Non-small cell lung cancer | 0.4741262 | 28 | 2 |
| mTOR signaling pathway | 0.4750969 | 19 | 1 |
| Protein processing in endoplasmic reticulum | 0.4871043 | 37 | 1 |
| Glycosphingolipid biosynthesis - lacto and neolacto series | 0.5811645 | 4 | 1 |
| Glutamatergic synapse | 0.5835907 | 25 | 1 |
| Rap1 signaling pathway | 0.5862067 | 44 | 1 |
| Adherens junction | 0.6094392 | 37 | 2 |
| Bacterial invasion of epithelial cells | 0.6335391 | 21 | 1 |
| HIF-1 signaling pathway | 0.6611199 | 26 | 1 |
| Ras signaling pathway | 0.7022114 | 60 | 1 |
| Thyroid cancer | 0.7162268 | 11 | 1 |
| Amyotrophic lateral sclerosis (ALS) | 0.7430115 | 14 | 1 |
| Pantothenate and CoA biosynthesis | 0.7506027 | 6 | 1 |
| Long-term depression | 0.7514546 | 16 | 1 |
| Dorso-ventral axis formation | 0.758026 | 12 | 1 |
| Cell adhesion molecules (CAMs) | 0.7797345 | 11 | 1 |
| Endocrine and other factor-regulated calcium reabsorption | 0.8933441 | 13 | 1 |
| Glycosaminoglycan biosynthesis - heparan sulfate / heparin | 0.9082822 | 5 | 1 |
| Galactose metabolism | 0.9484585 | 2 | 1 |
| 2-Oxocarboxylic acid metabolism | 0.9566452 | 5 | 1 |
| Tyrosine metabolism | 0.9623393 | 5 | 1 |
| Valine, leucine and isoleucine biosynthesis | 0.9749657 | 1 | 1 |
| Alanine, aspartate and glutamate metabolism | 0.9853395 | 7 | 1 |

| | | | |
|---|---|---|---|
| Lysine biosynthesis | 0.9902207 | 2 | 1 |
| Steroid biosynthesis | 0.9930727 | 1 | 1 |

**Supplementary Table 5: Predicted targets from Glutamatergic Pathway**

| Sr no. | Genes | Ensmbl ID |
|---|---|---|
| 1 | ADCY1 | ENSG00000164742 |
| 2 | GNG11 | ENSG00000127920 |
| 3 | ADRBK2 | ENSG00000100077 |
| 4 | GRM5 | ENSG00000168959 |
| 5 | GNG12 | ENSG00000172380 |
| 6 | PPP3R1 | ENSG00000221823 |
| 7 | GNAI3 | ENSG00000065135 |
| 8 | HOMER2 | ENSG00000103942 |
| 9 | PPP3CA | ENSG00000138814 |
| 10 | PLCB1 | ENSG00000182621 |
| 11 | PPP3CB | ENSG00000107758 |
| 12 | SLC38A2 | ENSG00000134294 |
| 13 | GRM7 | ENSG00000196277 |
| 14 | GRIA4 | ENSG00000152578 |
| 15 | HOMER1 | ENSG00000152413 |
| 16 | GNG4 | ENSG00000168243 |
| 17 | GRM4 | ENSG00000124493 |
| 18 | CACNA1D | ENSG00000157388 |
| 19 | GNG5 | ENSG00000174021 |
| 20 | GRIN2A | ENSG00000183454 |
| 21 | SLC1A2 | ENSG00000110436 |
| 22 | GNAI1 | ENSG00000127955 |
| 23 | PRKACB | ENSG00000142875 |
| 24 | GRIA3 | ENSG00000125675 |
| 25 | GRIK3 | ENSG00000163873 |
| 26 | GRIN2B | ENSG00000273079 |

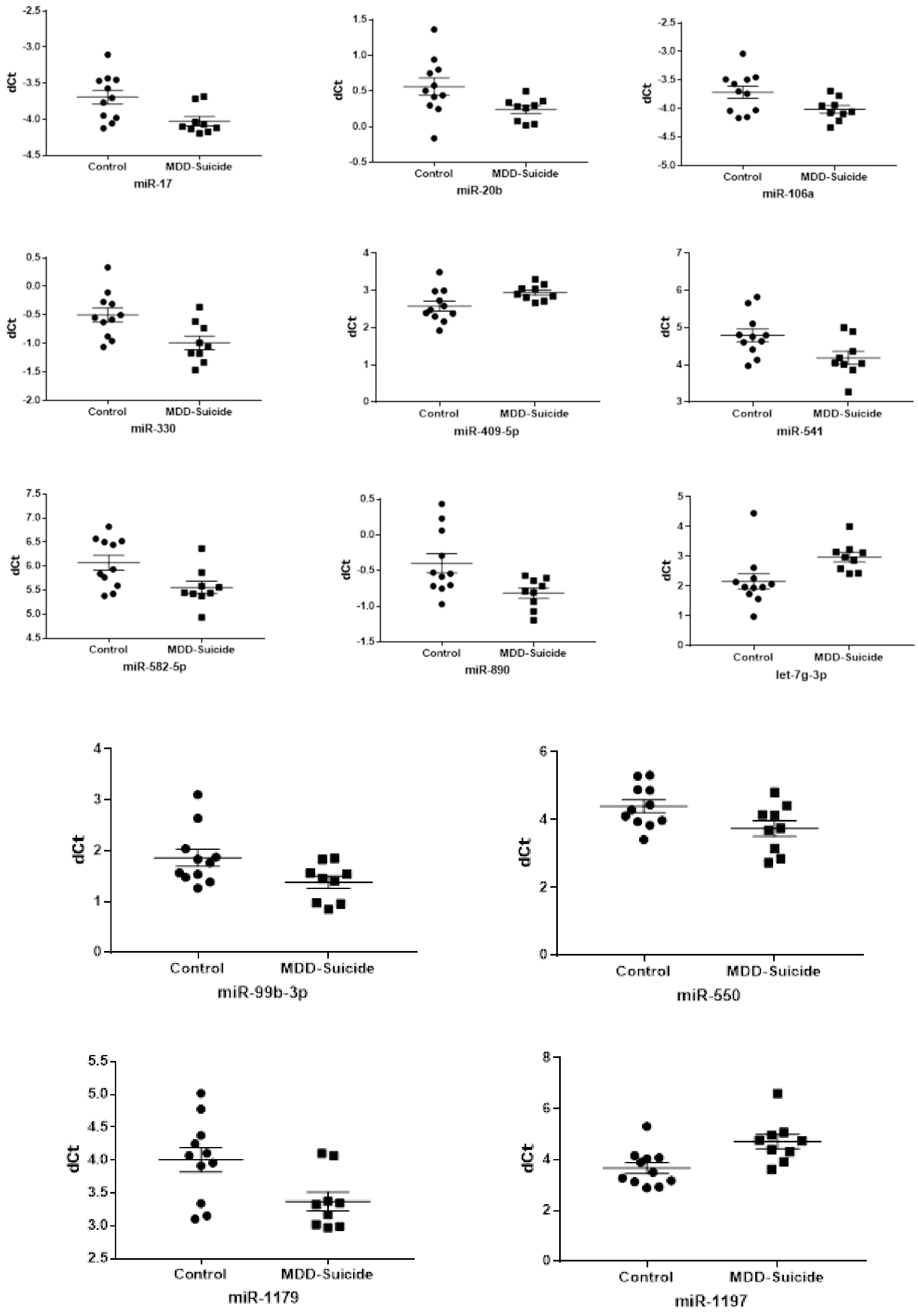


**Supplementary Figure 1.** Scatter plots of significantly altered miRNAs in locus coeruleus of MDD-suicide subjects (n = 9) and healthy controls (n = 11). Data represented as ± SEM.

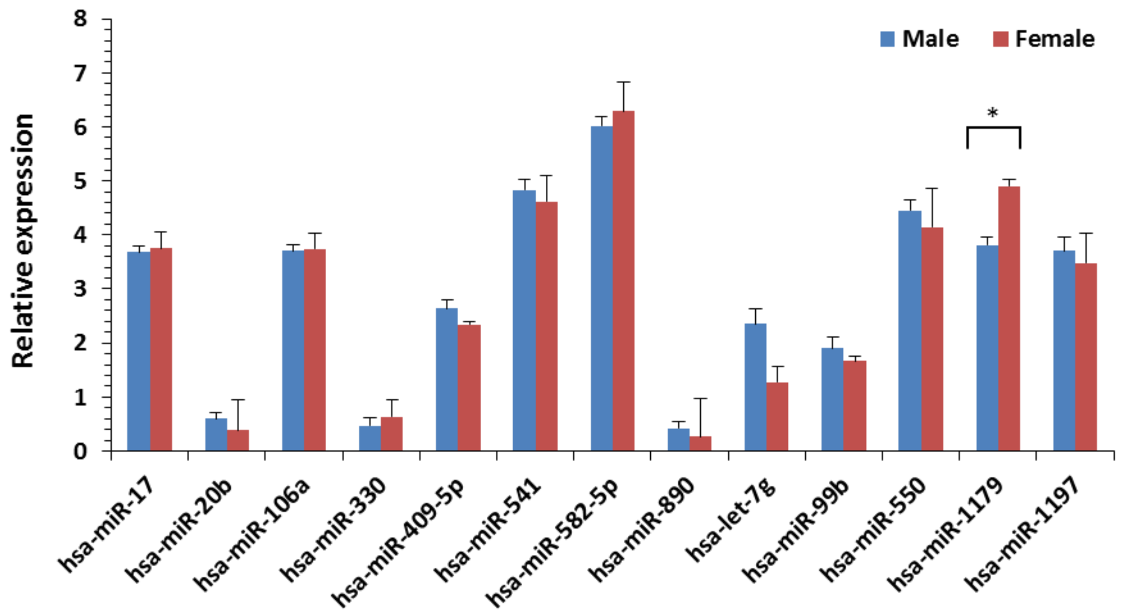


**Supplementary Figure 2.** Effect of gender on miRNA expression levels in locus coeruleus of healthy control subjects. The bar diagram showing the effect of gender on expression of hsa-miR-17-5p, hsa-miR-20b-5p, hsa-miR-106a-5p, hsa-miR-330-3p, hsa-miR-409-5p, hsa-miR-541-3p, hsa-miR-582-5p, hsa-miR-890, hsa-let-7g-3p, hsa-miR-99b-3p, hsa-miR-550-5p, hsa-miR-1179 and hsa-miR-1197. There were 9 males and 2 females in the control group. The levels of significance were determined with Student's t-test for each miRNA. Significant effect of gender was observed for miR-1179 ($p = 0.014$).

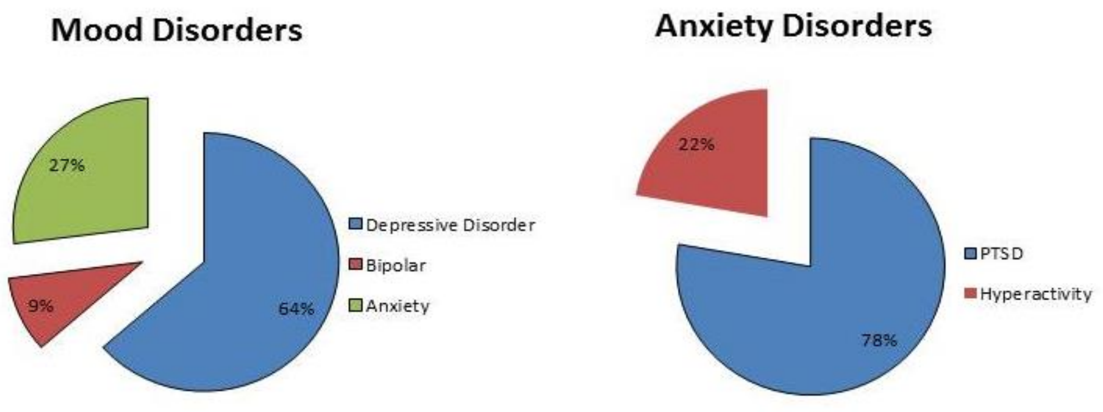


**Supplementary Figure 3.** Relative distribution of target genes of altered miRNAs under various sub categories of mood and anxiety disorders. Ingenuity pathway based in silico analysis (IPA) identified the disease associated pathways based on relative involvement of protein coding genes found to be the targets of altered miRNAs from MDD-suicide group.

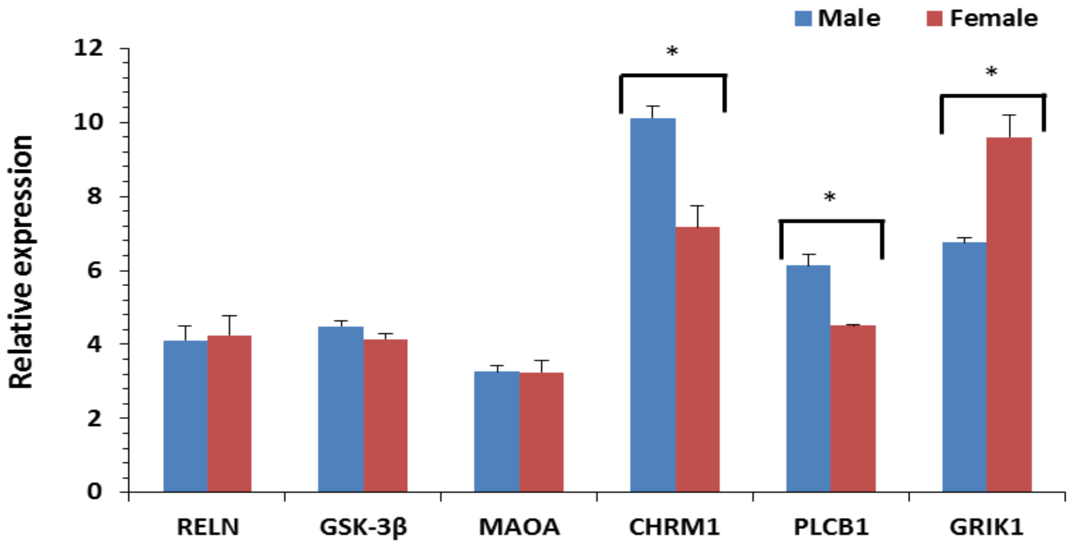


**Supplementary Figure 4.** Effect of gender on mRNA expression levels in locus coeruleus of healthy control subjects. The bar diagram demonstrates the gender specific effect on the expression of RELN (data represented as n = 9 males & n = 2 females for respective gene), GSK-3β (data represented as n = 8 males & n = 2 females for respective gene), MAOA (data represented as n = 7 males & n = 2 females for respective gene), CHRM1 (data represented as n = 9 males & n = 2 females for respective gene) and PLCB1 (data represented as n = 9 males & n = 2 females) and GRIK1(data represented as n = 8 males & n = 2 females) transcripts in post-mortem brain samples of healthy control. Significant effect of gender was observed on CHRM1 (p = 0.003), PLCB1 (p = 0.033) and GRIK (p = 0.0005) gene.
